# Financial Incentives for Linkage to Care and Viral Suppression Among HIV-Positive Patients: A Randomized Clinical Trial (HPTN 065)

**DOI:** 10.1001/jamainternmed.2017.2158

**Published:** 2017-08-07

**Authors:** Wafaa M. El-Sadr, Deborah Donnell, Geetha Beauchamp, H. Irene Hall, Lucia V. Torian, Barry Zingman, Garret Lum, Michael Kharfen, Richard Elion, Jason Leider, Fred M. Gordin, Vanessa Elharrar, David Burns, Allison Zerbe, Theresa Gamble, Bernard Branson

**Affiliations:** 1 ICAP, Mailman School of Public Health, Columbia University, New York, New York; 2 Vaccine and Infectious Disease Division, Fred Hutchinson Cancer Research Center, Seattle, Washington; 3 Division of HIV/AIDS Prevention, National Center for HIV/AIDS, Viral Hepatitis, STD, and TB Prevention, Centers for Disease Control and Prevention, Atlanta, Georgia; 4 New York City Department of Health and Mental Hygiene, New York; 5 Montefiore Medical Center, Albert Einstein College of Medicine, New York, New York; 6 District of Columbia Department of Health, HIV/AIDS, Hepatitis, STD and TB Administration, Washington, DC; 7 George Washington University, School of Medicine, Washington, DC; 8 Albert Einstein College of Medicine, New York, New York; 9 Washington DC VA Medical Center, Washington, DC; 10 Office of AIDS Research, National Institutes of Health, Bethesda, Maryland; 11 Division of AIDS, National Institute of Allergy and Infectious Diseases, Bethesda, Maryland; 12 HPTN Leadership and Operations Center, FHI360, Durham, North Carolina; 13 Scientific Affairs LLC, Atlanta, Georgia

## Abstract

**Importance:**

Achieving linkage to care and viral suppression in human immunodeficiency virus (HIV)-positive patients improves their well-being and prevents new infections. Current gaps in the HIV care continuum substantially limit such benefits.

**Objective:**

To evaluate the effectiveness of financial incentives on linkage to care and viral suppression in HIV-positive patients.

**Design, Setting, and Participants:**

A large community-based clinical trial that randomized 37 HIV test and 39 HIV care sites in the Bronx, New York, and Washington, DC, to financial incentives or standard of care.

**Interventions:**

Participants at financial incentive test sites who had positive test results for HIV received coupons redeemable for $125 cash-equivalent gift cards upon linkage to care. HIV-positive patients receiving antiretroviral therapy at financial incentive care sites received $70 gift cards quarterly, if virally suppressed.

**Main Outcomes and Measures:**

Linkage to care: proportion of HIV-positive persons at the test site who linked to care within 3 months, as indicated by CD4^+^ and/or viral load test results done at a care site. Viral suppression: proportion of established patients at HIV care sites with suppressed viral load (<400 copies/mL), assessed at each calendar quarter. Outcomes assessed through laboratory test results reported to the National HIV Surveillance System.

**Results:**

A total of 1061 coupons were dispensed for linkage to care at 18 financial incentive test sites and 39 359 gift cards were dispensed to 9641 HIV-positive patients eligible for gift cards at 17 financial incentive care sites. Financial incentives did not increase linkage to care (adjusted odds ratio, 1.10; 95% CI, 0.73-1.67; *P* = .65). However, financial incentives significantly increased viral suppression. The overall proportion of patients with viral suppression was 3.8% higher (95% CI, 0.7%-6.8%; *P* = .01) at financial incentive sites compared with standard of care sites. Among patients not previously consistently virally suppressed, the proportion virally suppressed was 4.9% higher (95% CI, 1.4%-8.5%; *P* = .007) at financial incentive sites. In addition, continuity in care was 8.7% higher (95% CI, 4.2%-13.2%; *P* < .001) at financial incentive sites.

**Conclusions and Relevance:**

Financial incentives, as used in this study (HPTN 065), significantly increased viral suppression and regular clinic attendance among HIV-positive patients in care. No effect was noted on linkage to care. Financial incentives offer promise for improving adherence to treatment and viral suppression among HIV-positive patients.

**Trial Registration:**

clinicaltrials.gov Identifier: NCT01152918

## Key Points

**Question:**

Can the use of financial incentives improve linkage to care and viral suppression for human immunodeficiency virus (HIV)-positive patients?

**Findings:**

In this community-based clinical trial, 37 HIV test and 39 HIV care sites in the Bronx, New York, and Washington, DC, were site-randomized to financial incentives or standard of care. Financial incentives significantly increased viral suppression and regular clinic attendance among HIV-positive patients; however, financial incentives did not have a significant effect on linking HIV-positive individuals to care.

**Meaning:**

Use of financial incentives can lead to increased viral suppression and regular clinic attendance among HIV-positive patients in care.

## Teaser

This randomized clinical trial examines the effectiveness of financial incentives on linkage to care and viral suppression in HIV-positive patients.

## Introduction

Antiretroviral therapy resulting in viral suppression dramatically reduces human immunodeficiency virus (HIV)-related morbidity and risk of HIV transmission.^1^ To realize these benefits, gaps in the HIV care continuum must be minimized.^5^ In the United States, approximately 13% of HIV-positive individuals are unaware of their HIV infection and only 55% of patients diagnosed with HIV have achieved viral suppression.^9^

Financial incentives (FI) can assist in achieving desirable health behaviors.^10^ In a meta-analysis^13^ of clinical trials that used cash transfers for health behaviors, 10 of 11 studies demonstrated a positive effect, including 3 for medication adherence. Other studies have shown their effectiveness in decreasing risk for acquisition of HIV and other sexually transmitted infections.^14^

The potential for FI to close the gaps in the HIV care continuum motivated the HIV Prevention Trials Network (HPTN) study, HPTN 065, which evaluated the effectiveness of FI for improving linkage to care and viral suppression in the Bronx, New York, and Washington, DC, 2 communities severely affected by HIV.^18^

## Methods

### Study Setting

**Figure 1.**
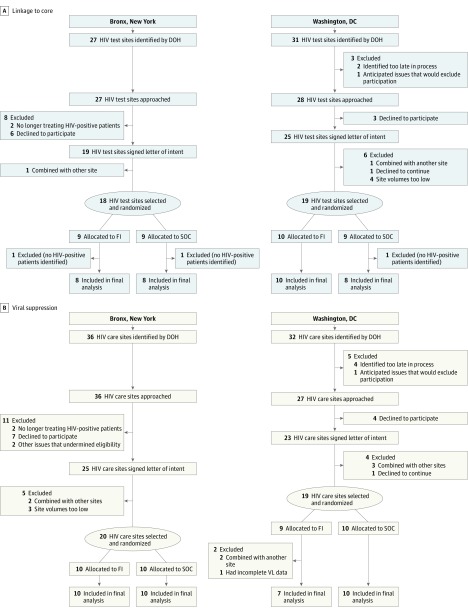
CONSORT Flow Diagram of Site Inclusion DOH indicates Department of Health; FI, financial incentives; HIV, human immunodeficiency virus; SOC, standard of care; VL, viral load.

Bronx, New York, and Washington, DC, HIV test sites with the highest number of newly HIV diagnosed individuals and HIV care sites treating the largest number of HIV-positive patients, determined based on 2008 to 2009 HIV surveillance data, were approached for potential study participation.^18^ The trial protocol is available in Supplement 1. The CONSORT diagram in Figure 1 shows the cascade of site selection and randomization. HIV test and care sites were separately randomized to FI or standard of care (SOC). Test sites provided FI from April 2011 until December 2012, and care sites from February 2011 through January 2013. Data from US surveillance system for 2010 were considered as baseline data.

### Financial Incentive Interventions

Individuals who tested positive for HIV at an FI HIV test site received a coupon redeemable within 3 months for 2 cash-equivalent gift cards: $25 on getting blood drawn for HIV-related tests and $100 on meeting with a clinician and developing a care plan. Few HIV test sites offered coupons to known HIV-positive persons who had not been in care for at least 12 months.

HIV-positive patients receiving antiretroviral therapy at FI care sites were eligible for incentives only if they were already engaged in care at that site (ie, had at least 1 prior viral load measurement there within the last 3 to 9 months). Such patients could qualify for a $70 gift card if their plasma viral load was suppressed (HIV RNA <400 copies/mL), for a maximum of once every 3 months for the duration of the FI component of the study (from February 2011 through January 2013). Clinicians received information on prevailing HIV treatment guidelines.^19^

### Study Outcomes

The outcomes for linkage to care, viral suppression, and continuity in care were assessed using data routinely reported to each jurisdiction’s HIV surveillance system.^18^ All confirmed diagnoses of HIV from HIV test sites were reported by providers and specific HIV-related test results (positive findings on Western blot, HIV RNA viral load, and CD4^+^ cell count) were reported by laboratories to the local surveillance system. Site-aggregated data per calendar quarter from 2010 through 2013 were transmitted to the HPTN statistical and data management center for analysis.

For linkage to care, the primary outcome was the proportion of individuals testing positive at each HIV test site who linked to care within 3 months, as indicated by those individuals’ record of CD4^+^ cell count or viral load test result in the surveillance system. HIV-positive patients were eligible if they were either newly diagnosed (based on no previous report in the surveillance system of a positive Western blot finding) or previously diagnosed but out of care (evidenced by no reported CD4^+^ cell count or viral load test result in the prior year).

For viral suppression, the primary outcome was the proportion of established patients at each HIV care site with suppressed viral load, assessed at each calendar quarter. For each quarter, patients were considered established in care if HIV laboratory test results were reported from that site in 2 different calendar quarters over the prior 15 months. A patient was considered virally suppressed if a viral load had been measured within 6 months and the most recent viral load was less than 400 copies/mL. Patients with no viral load test results in the past 6 months were assumed not suppressed. Viral suppression was evaluated for each site based on all viral load test results, regardless of whether these were from patients on antiretroviral therapy (treatment status is not captured in the surveillance system). Consistent viral suppression at baseline was defined as having a viral load of less than 400 copies/mL in every quarter in 2010.

For continuity in care (among established patients), the outcome was defined as the proportion of patients with CD4^+^ cell count or HIV viral load test results in the surveillance system during at least 4 of the prior 5 calendar quarters.

### Statistical Methods and Analyses

Site randomization aimed to achieve balance between arms by site size and preintervention endpoints, as measured by prerandomization (2008-2009) surveillance data: for test sites, balance in both the number of HIV-positive individuals identified in the previous year and the proportion of those linked to care within 3 months of HIV diagnosis; for care sites, balance in both the number of HIV-positive patients in care in the previous year and the proportion with viral suppression.^18^

#### Sample Size

For the linkage to care intervention, a sample size of 40 HIV test sites (20 per arm) was estimated to provide 80% power to detect a 13% increase in linkage to care, assuming an average of 54 HIV diagnoses per site and an intraclass correlation of 0.18. For the viral suppression intervention, a sample size of 40 HIV care sites (20 per arm) was estimated to provide 90% power to detect a 6% increase in viral suppression, assuming an average of 220 patients per site and an intraclass correlation of 0.10.

#### Statistical Analyses

The linkage to care intervention effect was estimated using logistic regression for a binary outcome of linkage to care adjusted for baseline site proportion linked to care and arm, using a generalized estimating equation (GEE) with exchangeable correlation structure.

The intervention effect for viral suppression was estimated using linear regression for the site-aggregated proportion of virally suppressed patients in each of the 5 quarters with arm as the primary covariate, adjusting for sites’ baseline proportion virally suppressed, weighted by the average number of patients at each site during the assessment period, and using a GEE approach with exchangeable correlation structure to account for 5 quarterly measures for each site. The same modeling approach was used for continuity in care, but adjusted for site’s baseline proportion of continuity in care. Thus, the model estimates an intervention effect corresponding to the increase in probability of viral suppression for a patient in care at a site implementing FI compared with SOC. Analysis of the peak intervention quarter (fourth quarter of 2012) for viral suppression, a prespecified sensitivity analysis, used simple weighted linear regression.

Four prespecified subgroup site analyses were planned: study community (Bronx vs Washington, DC), smaller vs larger sites (≤/> median number of patients), hospital vs community-based sites and lower vs higher percent with viral suppression at baseline (≤/> median percent). In addition, the effect of FI on patients not consistently virally suppressed prior to the intervention was assessed by excluding from the analysis all patients who achieved viral suppression in every quarter of 2010.

### Ethical Review

The study involved minimal risk. Because outcomes were reported using only quarterly site-aggregated data already reported to surveillance, no additional individual data were collected from sites. Each site’s affiliated institutional review board approved the study with a waiver of patient informed consent granted under 45 CFR 46.116 (d). The protocol is available in Supplement 1.

## Results

### Study Sites

Study site selection is shown in Figure 1 and baseline site characteristics are shown in eTable 1 in Supplement 2. A total of 37 test sites (18 in the Bronx and 19 in Washington, DC) participated in the linkage to care component and 34 were included in the analysis (3 were excluded because no HIV-positive patients were identified). During the baseline year, the mean (SD) number of HIV-positive cases was 35 (45), and sites’ mean (SD) linkage to care rate was 74% (29%). Characteristics of HIV-positive patients at baseline by arm were not statistically different (eTable 2 in Supplement 2).

A total of 39 HIV care sites participated in the viral suppression component (20 in the Bronx and 19 in Washington, DC), with 37 included in the analysis: 1 site was excluded owing to viral load test results not reported electronically to the surveillance system, and 2 sites within the same facility randomized to the same arm were combined owing to the inability to disaggregate their data in the surveillance system. At baseline, the mean (SD) number of patients in care was 374 (478), with a mean (SD) of 62% (16%) of patients virally suppressed. Characteristics of patients at HIV care sites by arm were not statistically different (eTable 3 in Supplement 2).

### Linkage to Care

A total of 1159 HIV-positive individuals were identified from test sites during the assessment; 389 in the Bronx and 770 in Washington, DC. The mean number of HIV-positive individuals diagnosed per site was 34. Among 914 patients identified in 2012 (303 in the Bronx and 611 in Washington, DC), 641 (70%) were men, 384 (42%) were men who have sex with men (99 [33%] in the Bronx and 285 [47%] in Washington, DC), 630 (69%) were black (151 [50%] in the Bronx and 479 [78%] in Washington, DC), 198 (22%) were Hispanic (140 [46%] in the Bronx and 58 [9%] in Washington, DC), and 150 (16%) were younger than 25 years (43 [14%] in the Bronx and 107 [17%] in Washington, DC). Characteristics were similar by study arm.

A total of 1061 coupons were dispensed, 238 in the Bronx and 823 in Washington, DC, and 79% were redeemed for both $25 and $100 gift cards (Table 1).

**Table 1.** Intervention Delivery Characteristics at HIV Test and Care Sites Randomized to Financial Incentives

Characteristic	Bronx, New York	Washington, DC	Overall
HIV test sites^a^	8	10	18
HIV-positive patients eligible for coupon	238	823	1061
Redeemed first visit incentive [$25], No. (%)	217 (91)	713 (87)	930 (88)
Redeemed both visit incentives [$125], No. (%)	194 (82)	646 (78)	840 (79)
Test site size (No. of persons testing HIV-positive), cases			
1-10	4	0	4
11-50	3	6	9
>50	1	4	5
HIV care sites	10	7	17
No. of HIV-positive persons eligible for FI per site, mean (SD)	528 (651)	572 (672)	546 (639)
Eligible care visits, mean (SD)	2930 (3917)	2661 (3166)	2819 (3522)
Proportion of eligible care visits qualified and received gift card (No.)	97% (7)	95% (6)	96% (6)
Care site size (No. of HIV-positive patients in care), patients			
51-200	5	3	8
201-400	2	1	3
401-1000	1	2	3
>1000	2	1	3

Abbreviations: FI, financial incentives; HIV, human immunodeficiency virus; SD, standard deviation.

^a^ HIV-positive individuals who received the second $100 financial incentive but whose data were missing for having received the first $25 financial incentive are counted as having received the first financial incentive.

Financial incentives did not significantly increase linkage to care compared with SOC with adjusted odds ratio 1.10 (95% CI, 0.73-1.67; *P* = .65), with no effects noted in predefined subgroups (Table 2).

**Table 2.** Effectiveness of Linkage to Care by Study Arm and Type of HIV Test Site

Variable	Sites, Mean (SD), %			Effect of FI, OR of Linkage (95% CI)^c^	*P* Value for Effect of FI
	Proportion of Patients Linked to Care at Baseline (p_baseline_)^a^	Proportion of Patients Linked to Care During Intervention (p_follow-up_)^b^	Change in Proportion of Patients Linked to Care From Baseline (p_follow-up—_p_baseline_)		
**Overall**					.65
FI (n = 18)	75 (30)	89 (11)	14 (34)	1.10 (0.73-1.67)	
SOC (n = 16)	73 (27)	83 (17)	11 (19)		
**Subgroups**					
Bronx, New York					.32
FI (n = 8)	82 (34)	93 (12)	11 (39)	1.58 (0.64-3.89)	
SOC (n = 8)	75 (28)	83 (20)	8 (20)		
Washington, DC					.65
FI (n = 10)	70 (28)	86 (10)	17 (32)	0.88 (0.52 to 1.50)	
SOC (n = 8)	70 (29)	84 (14)	14 (17)		
Community-based					.51
FI (n = 10)	65 (37)	92 (9)	28 (40)	1.33 (0.57 to 3.14)	
SOC (n = 9)	65 (34)	81 (22)	16 (23)		
Hospital-based					.77
FI (n = 8)	88 (12)	85 (13)	−3 (14)	0.94 (0.63 to 1.41)	
SOC (n = 7)	82 (12)	86 (7)	4 (7)		

Abbreviations: FI, financial incentives; HIV, human immunodeficiency virus; OR, odds ratio; SOC, standard of care.

^a^ Baseline period for linkage to care was from April 2010 to March 2011, before financial incentives.

^b^ Endpoint assessment period was from first quarter. All financial incentive sites were operating until financial incentives ended (September 1, 2011, to December 31, 2012).

^c^ OR from logistic model adjusted for sites' rate of linkage to care at baseline.

### Viral Suppression

At baseline, 16 208 patients were established in care at the sites (9703 in the Bronx and 6505 in Washington, DC). At baseline in 2010, most (10 201, 63%) were men (5485 [57%] in the Bronx and 4716 [72%] in Washington, DC), 4518 (28%) were men who have sex with men (1631 [17%] in the Bronx and 2887 [44%] in Washington, DC), 9355 (58%) were black (4434 [46%] in the Bronx and 4921 [76%] in Washington, DC), 5231 (32%) were Hispanic (4826 [50%] in the Bronx and 405 [6%] in Washington, DC), and 945 (6%) were younger than 25 years (575 in the Bronx and 370 in Washington, DC).

A total of 9641 patients (5275 in the Bronx and 4366 in Washington, DC) were eligible for gift cards at FI care sites, with 41 530 visits that potentially qualified for a gift card and 39 359 (95%) gift cards dispensed.

**Figure 2.**
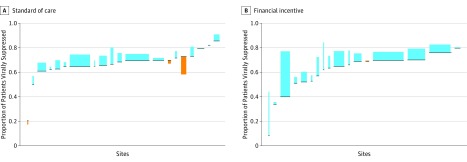
Change in Proportion of Patients With Viral Suppression by Arm and Site HIV care sites randomized to the 2 study arms are ordered by baseline viral suppression. Dark blue lines indicate baseline proportion of patients virally suppressed at baseline. Bars for each site indicate mean change in proportion of patients virally suppressed and the width of the bar is relative to the number of patients in care at the site. Bars in blue indicate increase and bars in orange indicate decrease in proportion of patients virally suppressed.

At baseline, mean (SD) overall viral suppression was 62% (16%) (eTable 1 and 4 in Supplement 2) and increased during the study at both FI and SOC sites (Table 3). The proportions of patients with viral suppression over time by site and study arm are shown in eFigure 1 in Supplement 2. Financial incentives had a statistically significant overall effect on viral suppression with a 3.8% (95% CI, 0.7%-6.8%; *P* = .01) higher proportion of virally suppressed patients at FI compared with SOC (Table 3) (Figure 2). The effect of FI was statistically significant at sites in Washington, DC (6.6% higher; 95% CI, 1.9%-11.3%; *P* = .006), at hospital-based sites (4.9% higher; 95% CI, 1.4%-8.5%; *P* = .007), as well as at sites with lower and higher baseline viral suppression (5.6% higher; 95% CI, 0.0%-11.3%; *P* = .05 and 3.6% higher; 95% CI, 0.3%-7.0%; *P* = .03, respectively), but not in the Bronx community-based sites or by size of the site. At the peak of the intervention, overall viral suppression was significantly higher by 4.6% (95% CI, 0.4%-8.8%; *P* = .031) at FI sites (eTable 5 in Supplement 2). Among the subpopulation of patients with viral load not consistently suppressed at baseline, viral suppression was significantly higher by 4.9% (95% CI, 1.4%-8.5%; *P* = .007) at FI sites (Table 3).

**Table 3.** Effect of Financial Incentives on Viral Suppression, on Viral Suppression in Patients Not Consistently Suppressed at Baseline, and on Continuity in Care Compared With Standard of Care, Bronx, New York, and Washington, DC

Variable	Viral Suppression			Viral Suppression in Patients Not Consistently Suppressed at Baseline			Continuity in Care		
	Change in Proportion With VS Between Baseline and Intervention, Mean (SD)^a^	Increase in Proportion With VS, % (95% CI)^b^	*P* Value	Change in Proportion With VS Between Baseline and Intervention, Mean (SD)^a^	Increase in Proportion With VS, % (95% CI)^b^	*P* Value	Change in Proportion With CC Between Baseline and Intervention, Mean (SD)^c^	Increase in Proportion of CC, % (95% CI)^b^	*P* Value
**Overall**			.01			.007			<.001
FI (n = 17)	11.5 (11.1)	3.8 (0.7 to 6.8)		22.3 (10.0)	4.9 (1.4 to 8.5)		16.5 (13.9)	8.7 (4.2 to 13.2)	
SOC (n = 20)	3.7 (5.9)			16.1 (8.4)			−1.8 (11.4)		
**Subgroups**									
Bronx, NY			.14			.48			<.001
FI (n = 10)	7.8 (6.7)	1.6 (−0.6 to 3.9)		20.5 (8.4)	0.9 (−1.7 to 3.5)		9.8 (9.0)	8.0 (4.1 to 11.9)	
SOC (n = 10)	5.2 (4.5)			18.3 (8.5)			7.0 (12.0)		
Washington, DC			.006			<.001			.03
FI (n = 7)	16.7 (14.4)	6.6 (1.9 to 11.3)		24.8 (12.2)	8.7 (3.9 to 13.4)		26.0 (14.6)	10.1 (1.2 to 19)	
SOC (n = 10)	2.2 (7.0)			14.0 (8.1)			3.4 (8.5)		
Hospital-based			.007			.01			.001
FI (n = 7)	14.1 (16.0)	4.9 (1.4 to 8.5)		23.1 (12.4)	5.9 (1.3 to 10.5)		14.6 (15.2)	8.7 (3.4 to 14)	
SOC (n = 7)	2.1 (7.8)			14.7 (6.9)			−7.7 (14.5)		
Community-based			.47			.11			.02
FI (n = 10)	9.6 (6.2)	1.2 (−2.0 to 4.3)		21.7 (8.6)	3.6 (−0.9 to 8.1)		17.8 (13.6)	9.4 (1.7 to 17.1)	
SOC (n = 13)	4.6 (4.8)			16.9 (9.3)			1.4 (8.4)		
Smaller (≤196 at baseline)			.05			.23			.02
FI (n = 9)	16.1 (13.3)	11.8 (−0.1 to 23.7)		23.9 (12.8)	9.2 (−5.7 to 24.1)		24.4 (13.7)	10.3 (1.5 to 19.2)	
SOC (n = 10)	3.8 (5.1)			15.7 (10.5)			−3.2 (14.9)		
Larger (>196 at baseline)			.08			.008			.005
FI (n = 8)	6.2 (4.3)	2.7 (−0.3 to 5.7)		20.4 (5.7)	4.1 (1.1 to 7.0)		7.5 (7.4)	8.0 (2.4 to 13.6)	
SOC (n = 10)	3.6 (6.9)			16.5 (6.3)			−0.4 (7.1)		
Lower base VS (baseline ≤66%)			.05			.06			.27
FI (n = 11)	15.1 (12.2)	5.6 (0.0 to 11.3)		23.6 (11.9)	7.7 (−0.5 to 15.9)		22.3 (13.7)	5.7 (−4.4 to 15.8)	
SOC (n = 9)	4.9 (3.9)			16.0 (7.1)			−4.9 (14.5)		
Higher base VS (baseline >66%)			.03			.02			<.001
FI (n = 6)	4.8 (3.9)	3.6 (0.3 to 7.0)		19.7 (4.9)	4.6 (0.8 to 8.4)		5.8 (5.8)	8.7 (3.6 to 13.8)	
SOC (n = 11)	2.7 (7.2)			16.3 (9.7)			0.7 (8.2)		

Abbreviations: CC, continuity in care; FI, financial incentives; GEE, generalized estimating equation; SOC, standard of care; VS, viral suppression.

^a^ Baseline period for VS was January 1, 2010, to March 31, 2011. Intervention assessment period began after financial incentives were in operation at sites for at least 6 months until financial incentives ended (January 1, 2012, to March 31, 2013). Columns report the mean and standard deviation for the average site change in VS from baseline over the sites in each group.

^b^ Columns report intervention effect based on a weighted GEE model that estimates the increase in probability of VS (CC) for FI compared with SOC adjusted for baseline VS (CC).

^c^ Baseline period for CC was January 1, 2010 to March 31, 2011. Intervention assessment period began after financial incentives were in operation at sites for at least 15 months until financial incentives ended (July 1, 2012 to March 31, 2013). Columns report the mean and standard deviation for the average site change in CC from baseline over the sites in each group.

### Continuity in Care

The proportion of patients with continuity in care was higher by 8.7% (95% CI, 4.2%-13.2%; *P* < .001) at FI compared with SOC sites. This finding was consistent in both cities; at hospital and community-based sites, at smaller as well as larger sites; and sites with higher viral suppression at baseline (Table 3).

## Discussion

This site-randomized, community-based study included a large number of sites that provide HIV testing and HIV care in the Bronx and Washington, DC, and for a substantial proportion of the HIV-positive patients in both communities. This study, the largest to evaluate the effectiveness of FI on HIV care-related behaviors, demonstrated that FI significantly increased by 3.8% the proportion of patients with viral suppression at FI compared with SOC sites. The effects observed were stronger in patients not consistently suppressed prior to the intervention. Financial incentives also substantially increased the proportion of patients reporting regularly for quarterly clinic visits. However, FI did not have a significant effect on linking HIV-positive individuals to care when compared with linkage at SOC sites.

Few studies have assessed the efficacy of FI on HIV care-related behaviors.^22^ In 1 study^22^ conducted among people who inject drugs (PWID) in India, the use of vouchers for food or household goods was associated with enhanced linkage to care. In 2 US studies^23^ of PWID, contingency management combined with voucher prize drawings increased adherence to antiretroviral therapy. In 2 other US studies, 1 found that cue training with monetary reinforcement was associated with a transient increase in adherence but had no effect on viral suppression, whereas the other did not demonstrate benefit of FI on viral suppression among substance users.^25^

In this study, while FI had a statistically significant overall effect on the proportion of patients virally suppressed, this effect was larger in Washington, DC. This may be owing to the fact that New York State was an earlier adopter of initiating antiretroviral therapy, regardless of CD4^+^ cell count, which may have made it more difficult to detect the effect of FI at the Bronx sites. In addition, FI may have had a more pronounced influence in getting patients to make regular clinic visits and achieve viral suppression in Washington, DC, because a smaller proportion of patients made regular clinic visits at baseline compared with the Bronx. We also found a significant effect of FI at hospital-based care sites, possibly because they provided care for less financially secure patients for whom FI may be more meaningful.

It should also be noted that, in this study, all patients on treatment at a site randomized to FI were eligible to receive them if virally suppressed, including those already virally suppressed. Thus, the larger effect of FI observed in the subset of patients not consistently suppressed at baseline, albeit an outcome not defined a priori, may reflect the potential added value of FI among such patients.^29^ In a study that assessed the effect of FI on adherence to warfarin, a significant effect was only noted in individuals with characteristics associated with nonadherence rather than in all patients.^30^

In this study, FI also significantly increased the proportion of patients regularly attending clinic visits. Poor retention in care has been associated with higher mortality.^31^ Regular clinical and laboratory monitoring is important to enable provision of adherence support, prevention counseling, or screening for other health conditions.^32^ However, care should be taken to safeguard against overutilization of clinic services motivated by FI.

Use of FI to motivate behaviors remains controversial.^13^ We took measures to prevent untoward consequences. Prior to study initiation, we consulted with the study’s community advisory group to determine the appropriate value of the FI. In addition, we used a site- rather than individual-randomized design to avoid the potential disruption of services and perceived inequities if only some patients within a site received FI. Last, to discourage patients from transferring care to FI sites, we required that patients be in care at a site for at least 3 months before receiving a gift card.

### Strengths and Limitations

The study had several important strengths. It was a community-wide effort implemented at hospital and community-based sites with a wide range of patient volumes. The outcome measure was laboratory-reported viral suppression, a biological indicator rather than self-reported adherence.^33^ A unique feature was the use of data from the US HIV Surveillance System to measure outcomes, an approach particularly well-suited for assessing a community-level intervention and one that avoided the selection bias inherent in recruitment and follow-up of individual participants.

The study also had several limitations. The effect of FI may have been diluted because, while only patients receiving antiretroviral therapy were eligible for FI, all patients in care at an HIV care site (including those not on treatment) were included in the assessment of the viral suppression outcome because treatment status is not available in the surveillance system. Overall data completeness was superior in the Bronx compared with Washington, DC; moreover, for some nonresident patients receiving care at sites in Washington, DC, CD4^+^ and viral load data were reported to their jurisdiction of residence rather than to Washington DC, possibly hindering complete capture of data. We also attempted to mitigate the potential of assessment bias caused by increased frequency of viral load assessment owing to FI by defining the viral load suppression outcome over a 6-month period consistent with recommendations in prevailing guidelines^32^; however, we are unable to assess whether this approach was fully successful. Finally, the study had limited power to asses changes in linkage to care because linkage was high at baseline, and the number of HIV-positive individuals identified per site was substantially lower than anticipated.

## Conclusions

HPTN 065, the largest study to date to our knowledge to evaluate the effect of FI on HIV-related care outcomes, demonstrated the overall effectiveness of FI for achieving viral suppression and regular clinic attendance. While seemingly modest, an increase of 4% in viral suppression with FI may potentially have considerable clinical and preventive implications on a population level, particularly in settings and among patients with less robust viral suppression. A recent study indicated that one-third of HIV-positive patients in care in the United States had detectable viral load for substantial durations of time, limiting their individual benefit and increasing their risk for transmitting HIV to others.^35^ Our study also demonstrated both the feasibility of using FI in a large community-wide effort and the successful linkage of a large-scale research endeavor with the established HIV surveillance system.^36^ Further analyses are ongoing to determine the cost-effectiveness of FI on viral suppression based on our study findings. In our study, we found that FI had no significant effect on linkage to care, an area ripe for further research.

In conclusion, while our findings offer an innovative intervention for achieving the treatment and prevention potential of antiretroviral therapy, a strategy that offers great promise for control of HIV in the United States and globally, more research is needed to determine how such an intervention can be implemented in programs and at scale.

## Notes

Supplement 1. Trial Protocol

Supplement 2. **eTable 1.** Baseline Characteristics of HIV Test and Care Sites, Bronx, NY and Washington, DC, 2010 and 2011 **eTable 2.** Baseline Characteristics of HIV-positive Patients at HIV Test Sites by Study Arm and Overall **eTable 3.** Baseline Characteristics of Patients at HIV Care Sites by Study Arm and Overall **eTable 4.** Baseline HIV Care Site Level Characteristics for Viral Suppression (VS) and Continuity in Care (CC) for Patients in Care **eTable 5.** Effect of Financial Incentives (FI) and Standard-of-Care (SOC) on Viral Load Suppression (VS) for Patients at Peak Intervention Period **eFigure 1.** Change in Proportion Virally Suppressed over Time by Study Arm
