# Supplementary material for: Financial Incentives for Linkage to Care and Viral Suppression Among HIV-Positive Patients: A Randomized Clinical Trial (HPTN 065)
Source: JAMA Intern Med. 2017 Aug 7;177(8):1083–92. doi: 10.1001/jamainternmed.2017.2158 (PMC5604092; doi:10.1001/jamainternmed.2017.2158)
Supplement: Supplement 1. — Trial Protocol [file jamainternmed-177-1083-s001.pdf]

This supplement contains the following items:

1. Original protocol, final protocol, summary of changes.

2. Original statistical analysis plan, final statistical analysis plan, summary of changes

TABLE OF CONTENTS

1. HPTN 065 Protocol, version 1.0 (01 March 2010)..pg. 3
2. HPTN 065 Protocol, version 3.0 (14 January 2014)..pg. 140
3. HPTN 065, Summary of Changes, Version 1.0 to Version 2.0...............................pg. 281
4. HPTN 065, Summary of Changes, Version 2.0 to Version 3.0...............................pg. 284
5. HPTN 065, Statistical Analysis Plan, Version 1.0 (13 June 2011)...........................pg. 288
6. HPTN 065, Statistical Analysis Plan, Version 2.0 (09 September 2014).................pg. 316
   * Includes summary of changes (Version 1.0 to Version 2.0) in Section 8

HPTN 065

TLC-Plus: A Study to Evaluate the Feasibility of an Enhanced Test, Link to Care, Plus Treat Approach for HIV Prevention in the United States

A Study of the HIV Prevention Trials Network

Sponsored by:

Division of AIDS (DAIDS), U.S. National Institute of Allergy and Infectious Diseases (NIAID)
U.S. National Institutes of Health (NIH)

Protocol Chair(s):
Wafaa El-Sadr, M.D., MPH
Columbia University and Harlem Hospital
New York, NY, USA

Bernard Branson, M.D.
Centers for Disease Control and Prevention (CDC)
Atlanta, GA, USA

Version 1.0
FINAL
01 March 2010

DAIDS Protocol #: 11685

Non-IND Study

HPTN 065

TLC-Plus: A Study to Evaluate the Feasibility of an Enhanced Test, Link to Care, Plus Treat Approach for HIV Prevention in the United States

TABLE OF CONTENTS

LIST OF ABBREVIATIONS AND ACRONYMS V

PROTOCOL TEAM ROSTER VIII

INVESTIGATOR SIGNATURE PAGE XIII

SCHEMA XIV

OVERVIEW OF STUDY DESIGN AND RANDOMIZATION SCHEME XVII

STUDY TIMELINE XVIII

1.0 INTRODUCTION 1

- 1.1 Background and Prior Research 1
- 1.2 Purpose 17
- 1.3 Rationale 18
- 1.4 Protocol Structure 23

2.0 EXPANDED HIV TESTING 23

- 2.1 Study Objectives for Expanded HIV Testing 23
- 2.2 Design for Expanded HIV Testing 24
- 2.3 Study Population for Expanded HIV Testing 24
- 2.4 Study Sites for Expanded HIV Testing 25
- 2.5 Interventions for Enhanced HIV Testing Activities 25
- 2.6 Study Procedures for Expanded HIV Testing 27
- 2.7 Study Duration for Expanded HIV Testing 32
- 2.8 Statistics and Data Analysis for Expanded HIV Testing 33
- 2.9 Human Subjects/Ethical Considerations 35

3.0 LINKAGE-TO-CARE 36

- 3.1 Study Objectives for Linkage-to-Care 36
- 3.2 Design for Linkage-to-Care 36
- 3.3 Study Population for Linkage-to-Care 37
- 3.4 Study Sites for Linkage-to-Care 37
- 3.5 Intervention for Linkage-to-Care 37
- 3.6 Study Procedures for Linkage-to-Care 38
- 3.7 Study Duration for Linkage-to-Care 40
- 3.8 Statistics and Data Analysis for Linkage-to-Care 41
- 3.9 Human Subjects/Ethical Considerations 45
- 3.10 Safety Monitoring and Adverse Event Reporting 46

4.0 VIRAL SUPPRESSION 46

- 4.1 Study Objectives for Viral Suppression 46
- 4.2 Design for the Viral Suppression 47
- 4.3 Study Population for Viral Suppression 47

4.4 STUDY SITES FOR THE VIRAL SUPPRESSION 47
4.5 INTERVENTION FOR THE VIRAL SUPPRESSION 48
4.6 STUDY PROCEDURES FOR THE VIRAL SUPPRESSION 48
4.7 STUDY DURATION FOR THE VIRAL SUPPRESSION 49
4.8 STATISTICS AND DATA ANALYSIS FOR THE VIRAL SUPPRESSION 50
4.9 HUMAN SUBJECTS/ETHICAL CONSIDERATIONS 55
4.10 SAFETY MONITORING AND ADVERSE EVENT REPORTING 55
5.0 PREVENTION FOR POSITIVES 56
5.1 STUDY OBJECTIVES FOR PREVENTION FOR POSITIVES 56
5.2 DESIGN FOR PREVENTION FOR POSITIVES 56
5.3 STUDY POPULATION FOR PREVENTION FOR POSITIVES 56
5.4 STUDY SITES FOR PREVENTION FOR POSITIVES 57
5.5 INTERVENTION FOR PREVENTION FOR POSITIVES 57
5.6 STUDY PROCEDURES FOR PREVENTION FOR POSITIVES 58
5.7 STUDY DURATION FOR PREVENTION FOR POSITIVES 59
5.8 SAFETY MONITORING AND ADVERSE EVENT REPORTING 59
5.9 STATISTICS AND DATA ANALYSIS FOR PREVENTION FOR POSITIVES 60
5.10 HUMAN SUBJECTS/ETHICAL CONSIDERATIONS 63
6.0 SURVEY OF PATIENTS AND PROVIDERS 64
6.1 STUDY OBJECTIVES FOR SURVEY OF PATIENTS AND PROVIDERS 64
6.2 DESIGN FOR THE SURVEY OF PATIENTS AND PROVIDERS 64
6.3 STUDY POPULATION FOR THE SURVEY OF PATIENTS AND PROVIDERS 65
6.4 STUDY SITES FOR THE SURVEY OF PATIENTS AND PROVIDERS 65
6.5 STUDY PROCEDURES FOR THE SURVEY OF PATIENTS 65
6.6 STUDY PROCEDURES FOR THE SURVEY OF PROVIDERS 66
6.7 STUDY DURATION FOR THE SURVEY OF PATIENTS AND PROVIDERS 66
6.8 STATISTICS AND DATA ANALYSIS FOR SURVEYS OF PATIENTS AND PROVIDERS 66
6.9 HUMAN SUBJECTS/ETHICAL CONSIDERATIONS 67
6.10 SAFETY MONITORING AND ADVERSE EVENT REPORTING 68
7.0 HIV SURVEILLANCE, ROUTINELY-COLLECTED AND OTHER SURVEY DATA 68
7.1 HIV TESTING DATA 68
7.2 HIV SURVEILLANCE DATA 71
7.3 BEHAVIORAL DATA 74
8.0 ADMINISTRATIVE PROCEDURES AND OPERATIONAL CONSIDERATIONS 76
8.1 STUDY ACTIVATION 77
8.2 STUDY COORDINATION 77
8.3 STUDY MONITORING 78
8.4 PROTOCOL COMPLIANCE 78
8.5 HUMAN SUBJECTS/ETHICAL CONSIDERATIONS 78
8.6 USE OF INFORMATION AND PUBLICATIONS 79
8.7 STUDY DISCONTINUATION 79
9.0 REFERENCES 81
APPENDICES 89

APPENDIX I: SCHEDULE OF STUDY VISITS AND PROCEDURES 90
APPENDIX IIA: PATIENT COMPUTER-DELIVERED INTERVENTION AND SURVEY INFORMED CONSENT FORM.....92
APPENDIX IIB: PROVIDER SURVEY ONLINE INFORMED CONSENT TEXT 97
APPENDIX IIIA: ADULT HIV/AIDS CONFIDENTIAL CASE REPORT 99
APPENDIX IIIB: BEHAVIORAL RISK FACTOR SURVEILLANCE SYSTEM (BRFSS).....102
APPENDIX IIIC: 2008 PEMS VARIABLE REQUIREMENTS 104
APPENDIX IIID: NYC HIV SURVEILLANCE PERFORMANCE INDICATORS.....111
APPENDIX IIIE: WASHINGTON, D.C. CORE HIV SURVEILLANCE SYSTEM.....116

HPTN 065
TLC-Plus: A Study to Evaluate the Feasibility of an Enhanced Test, Link to Care, Plus Treat Approach for HIV Prevention in the United States

LIST OF ABBREVIATIONS AND ACRONYMS

AA	African American
ACASI	Audio Computer-Assisted Self Interview
ACTG	AIDS Clinical Trials Group
AIDS	Acquired Immunodeficiency Syndrome
ADAP	AIDS Drug Assistance Program
ALIVE	AIDS Link to Intravenous Experience
AMPATH	Academic Model for the Prevention and Treatment of HIV/AIDS
ART	Antiretroviral Therapy
ARTAS	Antiretroviral Treatment Access Study
ASD	Adult/Adolescent Spectrum of HIV Disease
ASO	AIDS Service Organization
BRFSS	Behavioral Risk Factor Surveillance System
CARE+	Computer Assessment and Risk Reduction Education for HIV-positives
CATI	Computer-Assisted Telephone Interviewing
CBO	Community-Based Organization
CD4	surface glycoprotein that denotes helper T cells
CDC	Centers for Disease Control and Prevention
CHS	Community Health Survey
CLIA	Clinical Laboratory Improvement Amendments
CORE	Coordinating and Operations Center
CRF	Case Report Form
CSTE	Counsel of State and Territorial Epidemiologists
CTRS	Counseling, Testing and Referral Services
DAIDS	Division of AIDS
DC	District of Columbia
DHHS	Department of Health and Human Services
DOB	Date of Birth
DOC	Department of Corrections
DOH	Department of Health
DOHMH	Department of Health and Mental Hygiene
ED	Emergency Department
eHARS	enhanced HIV/AIDS Reporting System
EIA	Enzyme Immunoassay
EMR	Electronic Medical Record
FI	Financial Incentive
FSU	Field Services Unit
FY	Fiscal Year
GE	General Electric
GEE	Generalized Estimating Equations
HbA1c	Hemoglobin A1c

LIST OF ABBREVIATIONS AND ACRONYMS (continued)

HAART	Highly Active Antiretroviral Therapy
HAHSTA	HIV/AIDS, Hepatitis, STD and TB Administration
HARS	HIV/AIDS Reporting System
HCPI	Health Communications/Public Information
HCSUS	HIV Cost and Services Utilization Study
HHS	Health and Human Services
HIPAA	Health Insurance Portability and Accountability Act
HIV	Human Immunodeficiency Virus
HOPS	HIV Outpatient Study
HOPWA	Housing Opportunities for People with AIDS
HPTN	HIV Prevention Trials Network
HRSA	Health Resources and Services Administration
ICC	Intra-class Correlation
ICT	Information and Communication Technologies
ID	Identification
IDSA	Infectious Disease Society of America
IDU	Injecting Drug User
INR	International Normalized Ratio
IPV	Intimate Partner Violence
IRB	Institutional Review Board
JAIDS	Journal of Acquired Immune Deficiency Syndromes
MEMS	Medication Event Monitoring System
MCO	Managed Care Organization
MMP	Medical Monitoring Project
MMP	Morbidity Monitoring Project
MMWR	Morbidity and Mortality Weekly Report
MSM	Men who have Sex with Men
MTRH	Moi Teaching and Referral Hospital
NAAT	Nucleic Acid Amplification Testing
NHANES	National Health and Nutrition Examination Survey
NHBS	National HIV Behavioral Surveillance
NHBS-HET	National HIV Behavioral Surveillance – At Risk Heterosexuals
NHBS-IDU	National HIV Behavioral Surveillance – Injection Drug Users
NHBS-MSM	National HIV Behavioral Surveillance – Men who have Sex with Men
NHIS	National Health Interview Survey
NIAID	National Institute of Allergy and Infectious Diseases
NIH	National Institutes of Health
NIMH	National Institutes of Mental Health
NL	Network Laboratory
NY	New York
NYC	New York City
OARAC	Office of AIDS Research Advisory Committee

LIST OF ABBREVIATIONS AND ACRONYMS (continued)

OHRP	Office for Human Research Protections
PEMS	Program Evaluation and Monitoring System
PHMC	Public Health Management Corporation
PLWHA	People Living With HIV/AIDS
RCC	Regulatory Compliance Center
RCT	Randomized Controlled Trial
RFA	Request for Applications
RNA	Ribonucleic Acid
SCHARP	Statistical Center for HIV/AIDS Research and Prevention
SDMC	Statistics and Data Management Center
SES	Socio-economic Status
SHAS	Supplement to HIV/AIDS Surveillance
SMART	Strategies for Management of Antiretroviral Therapy
SMC	Study Monitoring Committee
SOC	Standard of Care
SQL	Structured Query Language
SSP	Study-Specific Procedures Manual
STD	Sexually Transmitted Disease
TA	Technical Assistance
TB	Tuberculosis
TLC	Test and Link to Care
UODT	Universal Offer Development Team
U.S.	United States
USPHS	United States Public Health Service
USA	United States of America
VL	Viral Load
WB	Western Blot
WHO	World Health Organization

HPTN 065
TLC-Plus: A Study to Evaluate the Feasibility of an Enhanced Test, Link to Care, Plus Treat Approach for HIV Prevention in the United States

PROTOCOL TEAM ROSTER

Carlos Allende, MHSA
VIP Community Services
1910 Arthur Ave., 5th floor
Bronx New York, NY 10457
Phone: (718) 583-5150 Ex 8065
Fax: (718) 902-4875
Email: callende@vipservices.org

Geetha Beauchamp, MS
SCHARP, Vaccine and Infectious Disease Institute
Fred Hutchinson Cancer Research Center
Seattle, Washington
Phone (206) 667-6167
Fax: (206) 667-4812
Email: geetha@scharp.org

Constance Benson, M.D.
Professor of Medicine
Division of Infectious Diseases
Principal Investigator/Chair, AIDS Clinical Trial Groups
I.D. Fellowship Program Director
Director, Antiviral Research Center
University of California, San Diego
U.S. Mail: 200 W. Arbor Drive
Mail Code 8208
San Diego, CA 92103-8208
FedEx, UPS, or Overnight Mail:
220 Dickinson Street, Suite A
San Diego, CA 92103
Phone: (619) 543-8080
Fax: (619) 543-5066
Email: cbenson@ucsd.edu

Judith Berger, M.D.
Chief, Division of Infectious Diseases and HIV Care: Medical Director Pathways Center for Comprehensive Care
St. Barnabas Hospital
4422 Third Ave
Bronx, NY 10457
Phone: (718) 960-6205
Fax: (718) 960-3218
Email: judyberger@pol.net
Email: jberger@sbhny.org

Bernard Branson, M.D
Center for Disease Control and Prevention
Division of HIV/AIDS Prevention
1600 Clifton Road MS D-21
Atlanta Georgia 30333
Phone: (404) 639-6166
Fax: (404) 639-0897
Email: BBranson@cdc.gov

Kate Buchacz, Ph.D.
Centers for Disease Control and Prevention
Divisions of HIV/AIDS Prevention
1600 Clifton Road, MS E-45
Atlanta, GA 30333
Phone: (404) 639-5167
Fax: (404)-639-6127
E-mail: acu7@cdc.gov

David Burns, M.D., M.P.H.
Division of AIDS, Prevention Research Branch, National Institute of Allergy and Infectious Diseases
6700 B Rockledge Drive
Bethesda, MD, 20892
Phone: (301) 435-8896
Fax: (301) 496-8530
Email: burnsda@niaid.nih.gov

HPTN 065
TLC-Plus: A Study to Evaluate the Feasibility of an Enhanced Test, Link to Care, Plus Treat Approach for HIV Prevention in the United States

PROTOCOL TEAM ROSTER (continued)

Robert George Chin, M.D.
Associate Chair, Department of Emergency Medicine
Lincoln Medical and Mental Health Center
234 East 149th Street
Bronx, NY 10451
Phone : (718) 579-6010
Fax: (718) 579-4822
Email: robert.chin@nychhc.org

Blayne Cutler, M.D., Ph.D.
Director, HIV Prevention
Bureau of HIV/AIDS Prevention and Control
New York City Department of Health and Mental Hygiene
40 Worth Street, CN-A1
New York, NY 10013
Phone: (212) 788-4484
Email: bcutler@health.nyc.gov

Deborah Donnell, Ph.D.
SCHARP, Vaccine and Infectious Disease Institute
Fred Hutchinson Cancer Research Center
1100 Fairview Ave. N.,
Mailstop: M2-C200
P.O. Box 19024
Seattle, WA 98109-1024
Phone: (206) 667-5661
Email: deborah@scharp.org

Rick Elion, M.D.
Whitman Walker Clinic
1701 14th St., NW
Washington, DC 20009
Phone: (202) 745-6142
Fax: (202) 745-0238
Email: Rickelion@gmail.com

Wafaa El-Sadr, M.D., M.P.H.
Columbia University and Harlem Hospital
Mailman School of Public Health
722 West 168th Street
Room 715
New York, NY 10032
Phone: (212) 342-0532 or (212) 939-2936
Fax: (212) 342-1824 or (212) 939-2968
Email: wme1@mail.cumc.columbia.edu

Lisa Fitzpatrick. M.D., M.P.H.
Associate Professor of Medicine
Howard University School of Medicine
Division of Infectious Diseases
2041 Georgia Avenue, NW
Room 5-C-16
Washington, DC 20060
Phone: (202) 865-1877
Fax : (202) 865-4607
Email: lfitzpatrick@howard.edu

Donna Futterman, M.D.
Director, Adolescent AIDS Program
Professor of Clinical Pediatrics
Montefiore Medical Center
Albert Einstein College of Medicine
111 East 210th Street
Bronx, NY 10467
Phone: (718) 882-0322
Email: dfutterman@adolescentAIDS.org

Theresa Gamble, Ph.D.
Family Health International
2224 E. NC 54
Durham, NC 27713
Phone: (919) 544-7040, Ex 11350
Fax: (919) 544-7261
Email: tgamble@fhi.org

HPTN 065
TLC-Plus: A Study to Evaluate the Feasibility of an Enhanced Test, Link to Care, Plus Treat Approach for HIV Prevention in the United States

PROTOCOL TEAM ROSTER (continued)

Cynthia Grossman, Ph.D.
National Institute of Mental Health
6001 Executive Boulevard
Room 6201, MSC 9619
Bethesda, MD, 20892
Phone: (301) 443-8962
Email: grossmanc@mail.nih.gov

Shannon Hader, M.D., M.P.H.
District of Columbia Department of Health
64 New York Avenue, NE
Washington DC 20002
Phone: (202) 671-4900
Fax: (202) 671-4860
Email: shannon.hader@dc.gov

H. Irene Hall, Ph.D.
CDC - Division of HIV/AIDS Prevention
National Center for HIV/AIDS, Viral Hepatitis, STD, and TB Prevention
1600 Clifton Road, MS E 47
Atlanta, Georgia 30333
Phone: (404) 639-2050
Fax: (404) 639-2980
Email: ixh1@cdc.gov

Sally Hodder, M.D.
UMDNJ- Medical School
185 South Orange Ave., MSB I-510
Newark, NJ 07103
Phone: (973) 972-3846
Fax: (973) 972-2122
Email: hoddersa@umdnj.edu

Jessica Justman, M.D.
Columbia University
Mailman School of Public Health
722 West 168th Street, Room #714
New York, NY 10032
Phone: (212) 342-0537
Fax: (212) 342-1824
E-mail: jj2158@columbia.edu

Ann Kurth, CNM, Ph.D.
Professor, New York University
College of Nursing
726 Broadway, Rm. 1006
New York, NY 10003
Phone: (212) 998-5316
Fax: (212) 995-3413
Cell: (206) 795-3616
Email: akurth@nyu.edu

Fabienne Laraque, M.D., M.P.H.
Director, Care, Treatment and Housing Program, Bureau of HIV Prevention and Control
New York City Department of Health and Mental Hygiene
40 Worth Street, Room 1502
New York, NY 10013
Phone: (212) 788-5070
Fax: (212) 788-2273
Email: flaraque@health.nyc.gov

Jason Leider, M.D., Ph.D.
North Bronx Health Care Network
1400 Pelham Parkway South
Building #5, Rm 622
Bronx, NY 10461
Phone: (718) 918-3669
Fax: (718) 918-7686
Email: Jason.Leider@nbhn.net

HPTN 065
TLC-Plus: A Study to Evaluate the Feasibility of an Enhanced Test, Link to Care, Plus Treat Approach for HIV Prevention in the United States

PROTOCOL TEAM ROSTER (continued)

Kenneth Mayer, M.D.
Brown University, School of Medicine
Miriam Hospital of Rhode Island
Division of Infectious Diseases
164 Summit Avenue
Providence, R. I. 02906
Phone: (401) 793-4710
Fax: (401) 793-4709
Email: Kenneth_Mayer@brown.edu

Candia Richards-Clarke, M.P.H.
Chief Operating Officer
Bronx AIDS Services, Inc.
540 East Fordham Rd
Bronx, NY 10458
Phone: (718) 295-5605
Fax: (718) 733-3429
Email: ctrichards@basnyc.org

Paul Richardson, M.Sc.
Johns Hopkins University
600 North Wolfe Street
Pathology 313
Baltimore, MD 21287
Phone: (410) 502-0435
Fax: (410) 614-0430
Email: pricha18@jhmi.edu

Nirupama Sista, Ph.D.
Family Health International
2224 E. NC 54
Durham, NC 27713
Phone: (919) 544-7040, Ext. 11590
Fax: (919) 544-7261
Email: nsista@fhi.org

Margo A. Smith, M.D.
Director, Section of Infectious Diseases
Washington Hospital Center INF
110 Irving Street, NW
Suite 2A-56
Washington, DC 20010
Phone: (202) 877-7164
Email: Margo.A.Smith@Medstar.net

Edward E. Telzak, M.D.
Chief, Division of Infectious Diseases
Director, AIDS Program
Bronx-Lebanon Hospital Center
1650 Grand Concourse
Bronx, NY 10456
Phone: (718) 960-1212
Fax: (718) 960-2054
Email: etelzak@bronxleb.org

Lucia V. Torian, Ph.D.
Deputy Director, HIV Epidemiology and Field Services Program
New York City Department of Health and Mental Hygiene
125 Worth St., CN#44
New York, NY 10013
Phone: (212) 442-3461
Fax: (212) 442-3482
Email: ltorian@health.nyc.gov

Melissa Turner, M.S.W., LICSW
Veteran Affairs Medical Center
Department of Infectious Diseases
50 Irving Street NW
Room 2C-211A
Washington, D.C. 20422
Phone: (202) 745-8695
Phone: (202) 745-8000, Ext. 5667
Fax: (202) 745-8432
Email: melissa.turner@va.gov

HPTN 065
TLC-Plus: A Study to Evaluate the Feasibility of an Enhanced Test, Link to Care, Plus Treat Approach for HIV Prevention in the United States

PROTOCOL TEAM ROSTER (continued)

Kevin Volpp, M.D., Ph.D.
Staff Physician, CHERP,
Philadelphia VA Medical Center
Director, Center for Health Incentives,
Leonard Davis Institute of Health Economics
Associate Professor, University of Pennsylvania School of Medicine and the Wharton School
1232 Blockley Hall, 423 Guardian Drive
Philadelphia, PA 19104-6021
Phone: (215) 573-0270
Fax: (215) 573-8778

Douglas J. Ward, M.D.
DuPont Circle Physicians Group
1737 20th Street, North West
Washington, DC 20009
Phone: (202) 745-0201
Fax: (202) 332-2794
Email: dward@dupontdocs.com

Angela Fulwood Wood, M.S.W.
Chief Operations Officer
Family and Medical Counseling Service, Inc.
2041 Martin Luther King Jr., Ave SE
Washington, DC 20020
Phone: (202) 889-7900
Fax: (202) 610-3095
Email: afulwood@fmcsinc.org

HPTN 065
TLC-Plus: A Study to Evaluate the Feasibility of an Enhanced Test, Link to Care, Plus Treat Approach for HIV Prevention in the United States
Version 1.0 / 01 March 2010

INVESTIGATOR SIGNATURE PAGE

A Study of the HIV Prevention Trials Network (HPTN)

Sponsored by:
Division of AIDS (DAIDS), U.S. National Institute of Allergy and Infectious Diseases (NIAID)
U.S. National Institutes of Health (NIH)

I, the Investigator of Record, agree to conduct this study in full accordance with the provisions of this protocol. I agree to maintain all study documentation for a minimum of three years after submission of the site's final Financial Status Report to the Division of AIDS (DAIDS), unless otherwise specified by DAIDS or the HIV Prevention Trials Network (HPTN) Coordinating and Operations Center. Publication of the results of this study will be governed by HPTN policies. Any presentation, abstract or manuscript will be made available by the investigators to the HPTN Manuscript Review Committee and DAIDS for review prior to submission.

I have read and understand the information in this protocol and will ensure that all associates, colleagues and employees assisting in the conduct of the study are informed about the obligations incurred by their contribution to the study.

____
Name of Investigator of Record

_________________________________ _________________________________
Signature of Investigator of Record Date

HPTN 065

TLC-Plus: A Study to Evaluate the Feasibility of an Enhanced Test, Link to Care, Plus Treat Approach for HIV Prevention in the United States

SCHEMA

Purpose: The main purpose of this study is to evaluate the feasibility of an enhanced community-level test, link to care, plus treat strategy in the United States. The study includes the following components:

- Expanded Human Immunodeficiency Virus (HIV) Testing
- Linkage-to-Care
- Viral Suppression
- Prevention for Positives
- Patient and Provider Surveys

Design: Each component of the study involves an independent design but is interrelated to the other components.

- The Expanded HIV Testing component involves social mobilization, with targeted messaging to promote testing, and implementation of the universal offer of HIV testing in emergency departments (EDs) and hospital inpatient admissions.
- The Linkage-to-Care and Viral Suppression components involve site randomization to test the effectiveness of a financial incentive (FI) intervention compared with the standard of care (SOC).
- The Prevention for Positives component uses individual randomization to compare the SOC plus a computer-delivered intervention with the SOC.
- The Patient and Provider Surveys will be administered at specific time points during the study to assess knowledge, attitudes and practices regarding early initiation of antiretroviral therapy (ART) and the FI interventions.

Study Sites: The study will be conducted in two intervention communities (the Bronx, New York and Washington, D.C.) and surveillance data from these communities will be compared with that from four non-intervention communities (Chicago, Illinois; Houston, Texas; Miami, Florida; and Philadelphia, Pennsylvania).

Study Population: This study will primarily target individuals age 18 years and older, and will also include younger individuals who are legally able to consent for HIV testing and care according to the state or local law in the two study communities. All of the study components will include HIV-positive individuals, with the exception of the provider survey sub-component.

Study Objectives: The main objective of the study is to determine the feasibility of a community focused enhanced test and link-to-care strategy in the United States. The study includes feasibility objectives for the Expanded HIV Testing, Linkage-to-Care and Viral Suppression components, and effectiveness objectives for the Linkage-to-Care, Viral Suppression and Prevention for Positive components.

Study Duration: The study will take place over 36 months.

HPTN 065

TLC-Plus: A Study to Evaluate the Feasibility of an Enhanced Test, Link to Care, Plus Treat Approach for HIV Prevention in the United States

SCHEMA (Continued)

Study Components:

The study includes the following five components:

Expanded HIV Testing: The expanded HIV testing intervention will supplement ongoing social mobilization and HIV testing efforts already in place in the two intervention communities. Refined messages will be added to ongoing social marketing and social networking efforts, targeted to increase HIV testing and testing frequency among men who have sex with men (MSM) and other subpopulations disproportionately affected by HIV. The intervention for EDs and hospital admissions includes encouraging hospital leadership and staff to institute standing orders for universal HIV testing. It also includes providing financial support for an increased number of HIV tests, and use of novel mechanisms to deliver information about HIV testing to patients.

Linkage-to-Care: HIV test sites will be randomized to either the FI intervention or to the SOC for linkage of HIV-positive patients identified at the testing sites to HIV care sites. At HIV test sites assigned to FIs, HIV-positive patients will be provided with a coupon to redeem at participating HIV care sites in the community. Upon completion of confirmatory HIV laboratory testing, patients with coupons will be given an FI ($25 gift card) at an HIV care site. A $100 gift card will be provided to patients upon completion of a care visit that includes interaction with a healthcare provider and discussion of HIV laboratory test results (*e.g.* CD4 cell count and viral load (VL) measurements).

Viral Suppression: HIV care sites will be randomized to either the FI intervention or the SOC for the achievement and maintenance of viral suppression. HIV care sites assigned to the FI intervention will provide an FI ($70 gift card) to HIV-positive patients on antiretroviral therapy (ART) demonstrating a suppressed VL (as defined by <400 copies/mL) at quarterly care visits.

Prevention for Positives: In a subset of patients enrolled from select HIV care sites in the two intervention communities, patients will be randomized either to an intervention arm (receiving SOC prevention activities plus a computer-delivered intervention for sexual and behavioral risk reduction) or to the control arm (receiving only the SOC prevention activities at the care site). In the intervention arm a modified version of the computerized counseling platform called Computer Assessment and Risk Reduction Education for HIV-positives (CARE+) will be used with an Audio Computer Assisted Self-Interviewing technique (ACASI). The CARE+/ACASI intervention session will ascertain behavioral risk, assess self-efficacy/motivation, and provide tailored feedback on specific risk behaviors. The computer-delivered intervention session will be administered every three months for one year. Participants in the control arm will also have CARE+/ACASI sessions every three months. However, participants in the control arm will only be administered behavior assessments via CARE+/ACASI and will not receive prevention messaging.

HPTN 065

TLC-Plus: A Study to Evaluate the Feasibility of an Enhanced Test, Link to Care, Plus Treat Approach for HIV Prevention in the United States

SCHEMA (Continued)

Study Components (continued):

Patient and Provider Surveys: The same computer system used for the Prevention for Positives intervention will be used to administer a survey to patients enrolled in that study component. The survey will assess their knowledge and attitudes towards ART use for treatment and prevention, ART adherence and FIs. Providers from these sites will be invited to complete a Web-based survey regarding their knowledge, attitudes and practices concerning ART for treatment and prevention as well as use of FIs. Both surveys will also collect some key sociodemographic data on surveyed populations to allow more in depth characterization of these populations.

Study Size: Each of the five study components has a target sample size.

- The universal offer of HIV testing will be made in EDs and during hospital admission at ~seven facilities in the Bronx, NY and ~seven in Washington, D.C. Additional focused messages promoting testing will be targeted to the entire population of each intervention community: the Bronx (population 1.4 million) and Washington, D.C. (population 600,000).
- The Linkage-to-Care component includes 40 HIV test sites (20 in each intervention community) and 40 HIV care sites (20 in each intervention community). We project that, by the end of the study, approximately 3000 new individuals in the two intervention communities will have tested positive for HIV.
- The Viral Suppression component includes the 40 HIV care sites (20 in each intervention community). Throughout the study duration, approximately 30,000 HIV-positive individuals will be in care, with an estimated 75% (22,500) eligible for ART in the two intervention communities.
- The Prevention for Positives component will be conducted at a total of twelve sites (six in each intervention community) with a total of 1320 patients participating.
- The 1320 patients at the twelve sites participating in the Prevention for Positives component will be surveyed. Providers at all participating HIV care sites will be invited to complete the provider survey.

HPTN 065
TLC-Plus: A Study to Evaluate the Feasibility of an Enhanced Test, Link to Care, Plus Treat Approach for HIV Prevention in the United States

OVERVIEW OF STUDY DESIGN AND RANDOMIZATION SCHEME

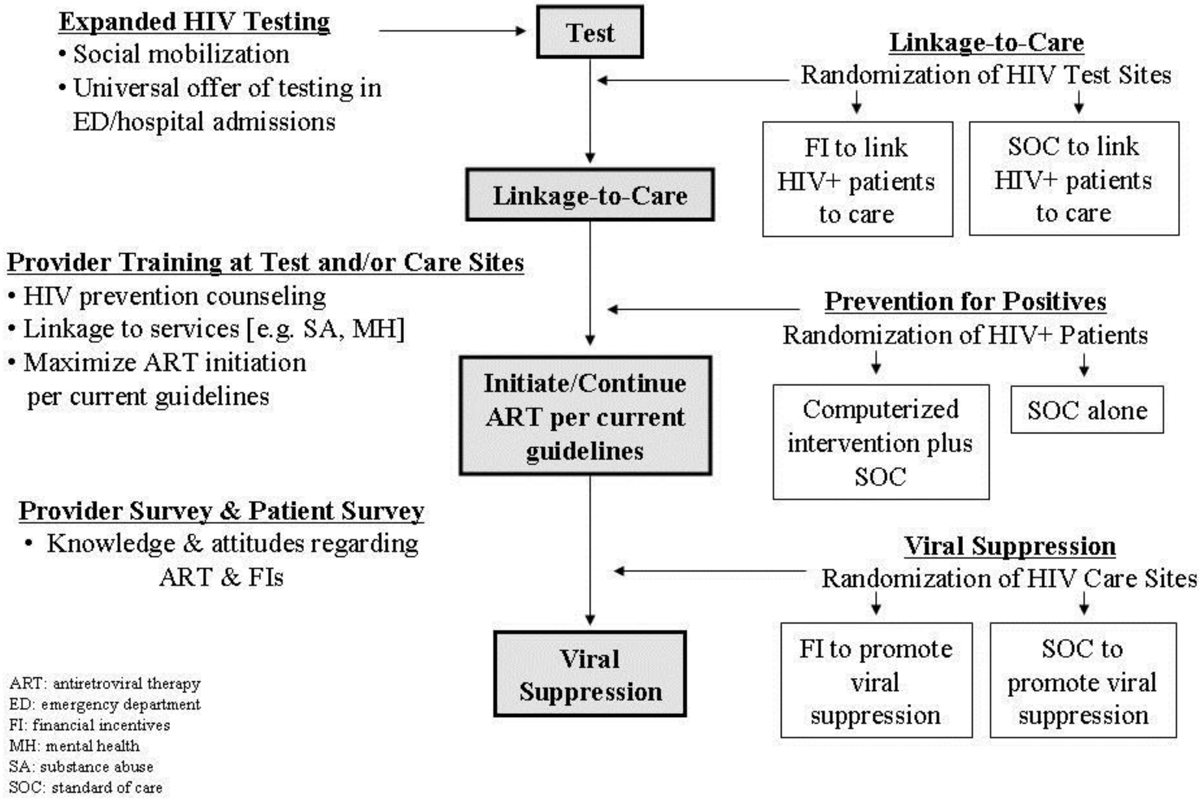

Summary of Study Components

Study Component	Design	Outcomes
Expanded HIV Testing	Descriptive, ecologic study	Feasibility
Linkage-to-Care	Two-arm, site-randomized, prospective, effectiveness study	Feasibility and effectiveness
Viral Suppression	Two-arm, site-randomized, prospective, effectiveness study	Feasibility and effectiveness
Prevention for Positives	Two-arm, individually-randomized, prospective, effectiveness study	Effectiveness
Patient and Provider Surveys	Quantitative	Survey

HPTN 065
TLC-Plus: A Study to Evaluate the Feasibility of an Enhanced Test, Link to Care, Plus Treat Approach for HIV Prevention in the United States

STUDY TIMELINE

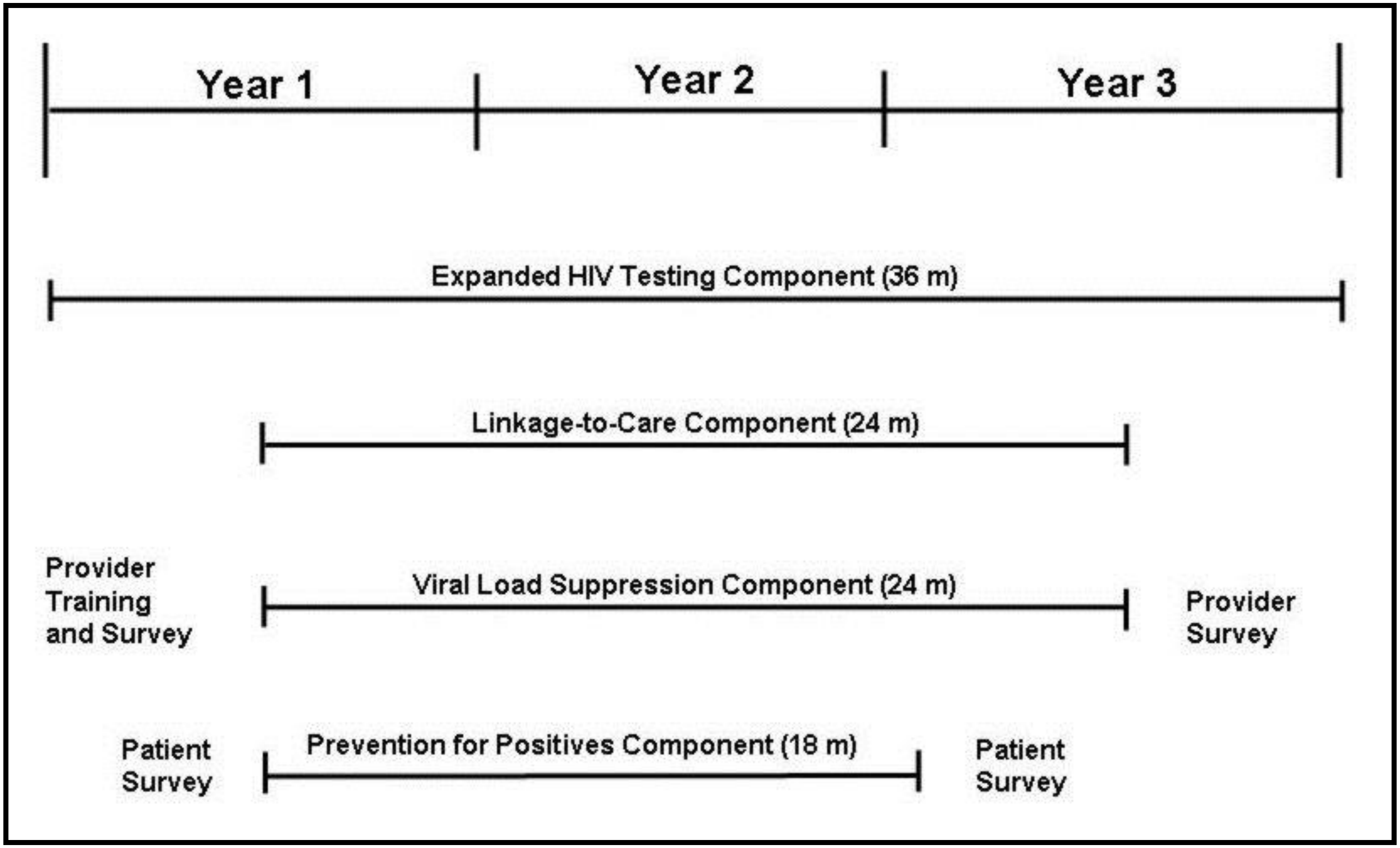

1.0 INTRODUCTION

1.1 Background and Prior Research

1.1.1 The Test-and-Treat Approach

The test-and-treat (TNT) strategy to prevent human immunodeficiency virus (HIV) transmission is based on expanded HIV testing to identify undiagnosed HIV infection, combined with prompt and effective initiation of ART to lower HIV viral load (VL) levels. While the potential effect of ART on HIV transmission has been previously studied by several investigators (Cohen, Gay et al. 2007), it received renewed attention in 2009. Granich and colleagues at the World Health Organization (WHO) published results of a modeling exercise that assessed the potential effect of such an approach on the HIV epidemic in South Africa (Granich, Gilks et al. 2009). The assumptions in the model included annual HIV testing of all adults older than 15 years of age, prompt initiation of ART in all those infected irrespective of disease stage, and a 99% decrease in infectiousness with highly effective first-line ART. The model indicated a dramatic drop in HIV incidence within 10 years and reduction of HIV prevalence to 1 percent in 50 years. The results of this modeling exercise have generated interest in the evaluation of such an approach (Assefa and Lera 2009; Cohen, Mastro et al. 2009; Dieffenbach and Fauci 2009; Epstein 2009; Granich, Gilks et al. 2009; Hsieh and de Arazoza 2009; Jurgens, Cohen et al. 2009; Wagner and Blower 2009).

The TNT strategy is hypothesized to achieve its effect on HIV transmission through the following two pathways:

- HIV testing identifies HIV-positive persons who, after learning their status, adopt safer behaviors, which decreases HIV transmission.
- HIV-positive individuals who initiate ART, and then maintain high levels of adherence and achieve viral suppression, are less infectious, which decreases HIV transmission.

Figure 1: Test and Treat Concept

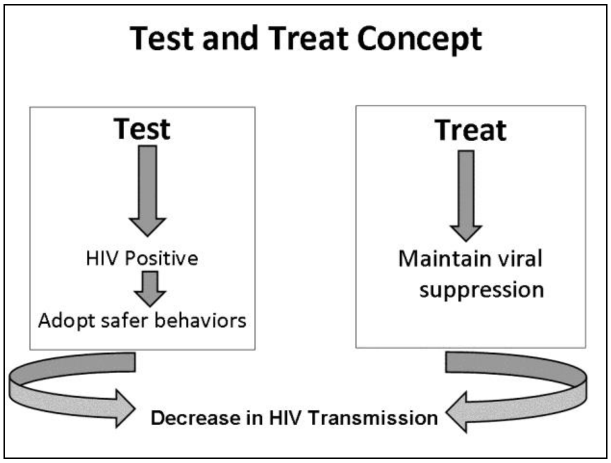

HIV screening has been found to be as cost-effective as other routine health interventions (Paltiel, Weinstein et al. 2005; Sanders, Bayoumi et al. 2005) and initiatives to expand HIV screening are underway in the United States. However, to optimize health outcomes, expanded testing efforts must be coupled with initiatives that ensure that both the newly diagnosed and those already known to have HIV infection are effectively linked to HIV care (Walensky, Weinstein et al. 2005) and receive ART as indicated with optimal adherence and suppression of viral replication.

The use of ART for prevention is supported by observational data from discordant couples that suggest that use of ART by the HIV sero-positive partner is associated with lower HIV incidence in the sero-negative partner (Quinn, Wawer et al. 2000; Sullivan, Kayitenkore et al. 2009). Other data also indicate a decrease in rates of HIV infection with use of ART (Bunnell, Ekwaru et al. 2006). However, definitive data for the effect of ART on transmission awaits the outcome of HIV Prevention Trials Network (HPTN) 052/AIDS Clinical Trials Group (ACTG) 5245. In addition, if ART is to be used widely for prevention purposes, its risk/benefit needs to be defined for a population not eligible for ART based on current therapeutic guidelines. Definitive data are lacking for the optimal timing of ART initiation for clinical benefit (Wilkin and Gulick 2008). Two ongoing randomized clinical trials (RCTs), the INSIGHT START study and HPTN 052/ACTG 5245, should yield such data on the efficacy and safety of initiation of ART at higher CD4 cell counts than the currently recommended threshold (Panel on Antiretroviral Guidelines for Adults and Adolescents 2009). In HPTN 052, HIV-positive individuals with CD4 count between 350 and 550 cells/mm^3 and who have HIV-discordant partners are randomized to initiate ART at CD4 cell count between 350 and 550 cells/mm^3 or at CD4 cell count between 200 and 250 cells/mm^3. The purpose of the latter study is to determine the effectiveness of ART on the sexual transmission of HIV to the uninfected partner as well as to determine the long-term effectiveness and safety of use of ART. In the INSIGHT START Study, HIV -positive patients with CD4 cell count of >500 cells/mm^3 are randomized to immediate ART initiation versus deferral of ART to when the CD4 cell count falls below 350 cells/mm^3.

1.1.2 HIV Testing in the United States, Washington, D.C., and the Bronx

1.1.2.1 Current HIV Testing in the United States

Based on data from the 2006 National Health Interview Survey (NHIS), the Centers for Disease Control and Prevention (CDC) estimates that 40% of all non-elderly adults (18 - 64 years) in the United States have been tested for HIV. A much smaller percentage of this population (10%) reported testing for HIV within the year prior to the survey. These levels of HIV testing, for both having ever been tested and for those tested in the past year, have remained constant from 2001 to 2006, suggesting that some individuals are tested repeatedly, while the majority of the U.S. population (60%) remains untested.

Approximately 21% of HIV-positive patients in the United States are not aware of their HIV infection (Campsmith, Rhodes et al. 2008) and not only may miss the benefits of HIV care and timely ART but also may continue to transmit HIV to their partners.

To increase the level of HIV testing in the United States and reduce the frequency of late HIV diagnoses, the CDC revised its recommendations for HIV testing in healthcare

settings and several federal agencies have new initiatives to increase testing in specific subpopulations (Duran, Beltrami et al. 2008). In 2006, the CDC recommended opt-out HIV screening as part of routine clinical care for adults and adolescents, including pregnant women, in all healthcare settings (Branson, Handsfield et al. 2006). In order to lower some of the barriers to testing, CDC recommendations require neither separate written consent nor HIV-prevention counseling as mandatory elements of HIV testing.

In September 2007, CDC funded an Expanded Testing Initiative in the 26 U.S. jurisdictions with the largest number of Acquired Immunodeficiency Syndrome (AIDS) cases. Through June 2009, 1.2 million persons have been tested under the Expanded Testing Initiative, with identification of nearly 14,400 new HIV diagnoses, 63% of which were among persons previously unaware they were infected. These tests were performed in Emergency Departments EDs (34%), sexually transmitted disease (STD) clinics (26%), community health centers (18%), corrections facilities (13%), and hospital inpatient settings (1.4%).

1.1.2.2 Current HIV Testing Activities in New York City and the Bronx

In the past three years, the New York City (NYC) Department of Health and Mental Hygiene (DOHMH) has scaled up its efforts to support routine HIV testing throughout its jurisdiction. As part of that effort, “The Bronx Knows” project was conceptualized. This program was initiated in early 2007 with the intention of scaling up HIV testing throughout the borough of the Bronx. After one year of capacity building, “The Bronx Knows” initiative was launched publicly on June 27, 2008 (National HIV Testing Day). The goals of the initiative are three-fold: to ensure that all Bronx residents who have never had an HIV test are screened for HIV over three years, to identify Bronx residents with undiagnosed HIV, and to link these HIV-positive individuals to high quality care and supportive services. While these are the stated goals of the initiative, residents with ongoing risk for HIV acquisition are also encouraged to be tested at least annually, per CDC recommendations.

As part of “The Bronx Knows,” the NYC DOHMH provides free test kits to organizations that offer HIV testing to uninsured individuals and to Community-Based Organizations (CBOs) that do not have dedicated funds for HIV testing. The DOHMH also provides technical assistance (TA) in the following areas: logistical/infrastructure change to support routine HIV screening (including on-site TA), obtaining clinical laboratory improvement amendment (CLIA) waivers, billing/reimbursement, rapid HIV testing technologies and data collection. In addition, the NYC DOHMH conducts sector-based workshops and Webinars in which participants review best practices and barriers experienced in their own sector (hospital, clinic or CBO). Finally, DOHMH provides ongoing social marketing and media campaigns targeting both Bronx residents (promoting routine HIV testing) and providers (promoting routine screening of patients for HIV).

By January 2009, more than 70 organizations had joined “The Bronx Knows.” These include seven of eight Bronx hospitals, 39 community health clinics and 20 of the borough's largest CBOs. Other participants include colleges/universities, faith-based institutions, local commercial establishments and community boards. Preliminary

aggregate data show that HIV testing among reporting organizations has increased by 28% since the launch of the initiative, despite New York State's requirement of separate signed informed consent for HIV testing.

1.1.2.3 Current HIV Testing Activities in Washington, D.C.

The Washington, D.C. Department of Health (DOH) has made HIV its number one health priority. Efforts have focused on aggressive diagnosis and treatment of HIV throughout Washington, D.C. Washington, D.C. does not require separate signed consent for HIV testing and was the first jurisdiction in the country to commit to and implement a policy of routine, opt-out HIV testing for residents. In June 2006, Washington, D.C. launched its, "Come Together D.C. - Get Screened for HIV" campaign. This campaign promoted testing among residents and recruited new testing organizations and testing sites. Starting in fiscal year 2007 (FY07) and continuing through FY08, testing expansion focused on scaling up routine, opt-out testing in the Department of Corrections (DOC) jail settings as well as in medical settings with special emphasis on hospital EDs, primary medical settings, managed care organizations (MCOs) and CBOs. In 2008, Washington, D.C. began implementation of a six-pronged strategy to scale up routine HIV screening citywide. The goals of this strategy are to: 1) increase the scope and scale of routine screening for HIV in clinical settings; 2) provide HIV testing and referral services, with an emphasis on identifying newly infected persons and ensuring that test results are received; 3) establish models to more efficiently link HIV-positive individuals to care; 4) collaborate with DOCs, medical care entities and CBOs to encourage and support Counseling, Testing and Referral Services (CTRS); 5) collect and analyze data to determine the scope and reach of CTRS programs; and 6) develop and disseminate tools to address myths and barriers related to routine HIV screening.

Despite efforts to increase provider awareness and practice of routinely offering HIV testing, behavioral survey and testing data suggest missed opportunities for routine testing in medical settings are still quite frequent, with nearly 75% of newly diagnosed HIV-positive persons reporting having seen a healthcare provider in the past twelve months without having been diagnosed (NHBS-Het Survey 2009). As a complement to efforts to increase provider participation in routine HIV testing, Washington, D.C.'s expansion efforts also include a direct-to-consumer marketing campaign called "Ask for the Test," which seeks to drive consumer demand by encouraging clients to ask for HIV testing if it is not routinely offered. It is anticipated that the approaches described above could result in an additional 85,000 tests per year.

1.1.3 HIV Care and ART Utilization in the United States, Washington, D.C. and the Bronx

The most recent population-based estimates on the proportion of HIV-positive Americans who are in HIV care and receiving ART are from 2003. These estimates depend on: 1) the fraction of HIV-positive persons who are undiagnosed and unaware of their HIV infection; 2) the fraction who have been diagnosed with HIV but are not in care; and 3) the changing recommendations with respect to timing of initiation of ART. Using data from the CDC's national HIV surveillance system and its 10-city Adult/Adolescent Spectrum of HIV Disease (ASD) project, the CDC estimated that, as of 2003, there were

480,000 Americans between the ages of 15 and 49 living with HIV or AIDS who were eligible for ART at a threshold of CD4 cell count < 350 cells/mm^3 (Teshale, Kamimoto et al. 2005). Of all eligible persons, approximately 340,000 (71%) were diagnosed with HIV and in care. Only 268,000 (55% of all eligible) were receiving ART. Based on these findings, the CDC recommended that three critical components be incorporated into the national HIV-prevention strategy: 1) increasing the number of HIV-positive individuals who are aware of their status; 2) linking them to HIV-prevention and care services; and 3) increasing the number receiving ART per federal guidelines (Branson, Handsfield et al. 2006). Given the growing number of HIV-infected persons living in the United States, estimated at 1.1 million as of 2006 (Campsmith, Rhodes et al. 2008), and the trend for initiation of therapy at CD4 cell count > 350 cells/mm^3 for some subsets of patients (Panel on Antiretroviral Guidelines for Adults and Adolescents 2009), it is likely that a greater number of persons (than the 0.5 million estimated in 2003 by Teshale et al.) are eligible for and in need of ART nationwide today.

In 2007, the CDC launched the Medical Monitoring Project (MMP) based on a national probability sample of HIV-positive persons receiving care in the United States to obtain data on patterns of use and quality of HIV care and prevention services among such persons, and their clinical and virologic status (McNaghten, Wolfe et al. 2007). Preliminary data from the MMP project are expected in 2010. Available data on current patterns of HIV care, ART treatment and ART adherence come primarily from a few large U.S. HIV cohorts (Palella, Delaney et al. 1998; Lazo, Gange et al. 2007; Mugavero, Lin et al. 2009). For instance, the CDC-funded HIV Outpatient Study (HOPS) has shown persistent reductions in mortality and hospitalizations due to increasing use of highly active antiretroviral therapy (HAART) over time (Palella, Delaney et al. 1998; Buchacz, Baker et al. 2008). Data from the HIV Cost and Services Utilization Study (HCSUS) (Cunningham, Markson et al. 2000) and the HIV Research Network (Gebo, JAIDS 2005) point to disparities in use of ART by sociodemographic characteristics (*e.g.*, race, gender and insurance status), some of which appear to persist to the present (Palella, Armon et al. 2008).

Linkage-to-care is required for HIV-positive persons to realize the benefits of both HIV care and prompt initiation of ART. Recently updated Infectious Disease Society of America (IDSA) guidelines for the management of persons with HIV emphasized the importance of linkage and retention in primary care (Aberg, Kaplan et al. 2009). Poor engagement in care has been found to be a predictor of higher mortality. Specifically, HIV-positive persons with poor retention in care have been found to have 50% higher mortality rates (Giordano, Gifford et al. 2007). Appropriate care includes determining stage of HIV disease through clinical evaluation and measurement of CD4 cell count and HIV ribonucleic acid (RNA) levels, other health maintenance interventions such as Pap smears for women, tuberculin skin testing, provision of drugs for prevention of opportunistic infections, health education, risk reduction, supportive counseling and ART for patients eligible for such treatment (Panel on Antiretroviral Guidelines for Adults and Adolescents 2009). However, data from multiple studies indicate both failure and delay in linkage-to-care (Shapiro, Morton et al. 1999; Giordano, Visnegarwala et al. 2005; Tobias, Cunningham et al. 2007). Nationally, various studies estimate that only 60-75% of persons are linked to HIV care within three to six months of receipt of HIV diagnosis (Torian, Wiewel et al. 2008; Reed, Hanson et al. 2009; Zetola, Bernstein et al. 2009).

In the supplement to HIV/AIDS surveillance (SHAS) project, analyses of interview data collected during 2000-2004 from over 20 U.S. cities, counties, and states, indicated that 72% of persons reported entering HIV care within three months of testing HIV-positive (Reed, Hanson et al. 2009). Barriers associated with failure or delayed entry into HIV care include structural, financial and personal/cultural factors. Such factors include first-time HIV testing and anonymous HIV testing (Reed, Hanson et al. 2009), longer waiting time for initial care appointment (Mugavero, Lin et al. 2007) or being diagnosed at earlier stage of HIV disease when patients report "feeling well" (Tobias, Cunningham et al. 2007). In a NYC study using 2003 HIV surveillance data, Torian et al. found that 64% of patients initiated HIV care within three months of HIV diagnosis, 19% initiated care more than three months after diagnosis, and 17% never initiated care. Delay in initiation of care was associated with HIV testing in a community site, in the correctional system, at a sexually transmitted infection or tuberculosis (TB) clinic, non-white race, injection drug use and foreign birth (Torian, Wiewel et al. 2008).

The national five-year multisite Outreach Initiative funded by Health Resources and Services Administration (HRSA) in 2001 identified a number of effective programmatic interventions for engaging and retaining HIV-positive persons in care, including education and outreach, strengthening of referrals, provision of linkage coordination and navigation services, and case management (Rajabiun, Mallinson et al. 2007; Tobias, Cunningham et al. 2007). For instance, an "HIV system navigation" approach was successful in reducing barriers to establishing care and improved health outcomes in a multisite study over 12-month period (Bradford, Coleman et al. 2007). The CDC-funded Antiretroviral Treatment Access Study (ARTAS) strengths-based case management intervention, delivered at CBOs and health departments in 10 sites across the United States during 2005-2006, resulted in 79% of recently diagnosed HIV-positive persons receiving HIV medical care within six months of enrolling in the study (Gardner, Metsch et al. 2005; Craw, Gardner et al. 2008).

1.1.3.1 Expected Rates of Viral Suppression

The epidemiology of HIV infection in the United States reveals a disproportionate percentage of African American (AA) people (41%) living with HIV compared to the general population (12%). Outcomes of virologic efficacy in all populations impacted by HIV in the United States have been assessed in studies to determine rates of success of such treatment and its effect on mortality and morbidity. An analysis from 1995 -2001 revealed that mortality rates for HIV-positive white men in the United States declined by 85% compared to 50% and 65% for HIV-positive AA women and men respectively (Prevention 2002). The latter finding may relate to factors such as late diagnosis of HIV, access to ART and challenges in achieving high rates of adherence with treatment.

A number of studies to evaluate these differences between race and virologic efficacy have been published. Weintrob et al. (Weintrob, Grandits et al. 2009) demonstrated significantly lower odds of obtaining virologic suppression in AA compared to Caucasians at six and 12 months in a cohort of military personnel. This group was studied because such individuals are likely to have similar access to medications and care. However, several factors may have still influenced this finding including adherence with ART, provider bias, or differences in education status impacting selection bias

regarding provider selection of initial therapy, or how long patients stay on a given regimen. Anastos et al. (Anastos, Schneider et al. 2005) reported similar findings in terms of rates of viral suppression in 961 HIV-infected women enrolled in the Women's Interagency HIV Study (WIHS). AA women were 30% less likely to achieve viral suppression and were also 30% more likely to rebound after achieving viral suppression. The reasons for these differences remains unclear, as subsequent adjustment for multiple variables including biologic and behavioral variables did not eliminate the differences noted by race. There have been other studies showing genetically determined factors that influence drug levels, but studies that link these same factors to virologic success or toxicity have been inconclusive.

Thus, evidence suggests that, in addition to the disproportionate impact of HIV on racial/ethnic minorities in the United States, there are racial disparities in terms of outcomes of HIV disease and these persist in HIV treatment. It is unclear if this disparity in outcomes points toward race as a biologic determinant or as an underlying complex sociologic marker. Nonetheless, the selection of measure of virologic success at an HIV RNA level of < 400 copies/mL is a reasonable measurement that should reflect success of ART in individuals irrespective of racial group.

1.1.3.2 Access to Support Services for Mental Health, Substance Use and Homelessness

Linkage to HIV care is critical for those infected with HIV; however, underlying conditions, such as substance use (Robison, Westfall et al. 2008; Wood, Kerr et al. 2008; Applebaum, Reilly et al. 2009; Norman, Basso et al. 2009), mental illness (Berg, Cooperman et al. 2009; Kapetanovic, Christensen et al. 2009; Roux, Carrieri et al. 2009), and homelessness (Royal, Kidder et al. 2009) can have significant impact on a person's ability to become successfully linked to HIV care, as well as to adhere to ART. Depression and anxiety are the most common psychiatric diagnoses in HIV-positive persons and are 5-10 times more common in this population than in the general population (Pence 2009). A limited number of randomized clinical trials have demonstrated the beneficial effect of various psychotherapy-based interventions on ART adherence (Weber, Christen et al. 2004; Wyatt, Longshore et al. 2004; Safren, Knauz et al. 2006). In addition, substance use may impact side effects of ART. For example, Cheng et al. noted that subjects with high alcohol use had higher odds of experiencing lipodystrophy (odds = 2.07, adjusted odds ratio = 0.90, 95% confidence interval = 4.73) compared to those with lower alcohol use (Cheng, Libman et al. 2009). It is estimated that at least 13% of HIV patients have mental health and substance use disorders (Weaver, Conover et al. 2009).

Access to supportive services such as substance use management, mental health, homelessness prevention and adherence support are some of the key components in comprehensive HIV programs. Such supportive services are available in both of the intervention cities. Study test sites will maintain the ability to refer clients with urgent needs (e.g., mental health) for necessary services. However, test sites will not routinely link patients to support services. The DOHs in both intervention communities actively encourage a *single* referral of HIV-positive patients from test sites to care sites. Multiple referrals can be overwhelming to newly HIV-diagnosed patients and may give the wrong impression that an individual must be drug-free in order to engage in HIV care, and may

paradoxically delay entry into care. Additionally, testing sites usually do not have ongoing relationships with persons in need of support services, and are thus not well suited for evaluating sustained linkages to these resources or adequacy of services to meet patient needs.

Participating HIV care sites will serve as the comprehensive ‘medical home’ for coordination of all HIV-positive patient care needs, including substance use, mental health, and other support services. As newly diagnosed HIV-positive patients present for care, care sites will evaluate CD4 cell count and VL. Care sites will then create an appropriate medical plan for each patient, consistent with current HIV care and treatment guidelines. Prior to study implementation, all HIV care site staff will be trained on the importance of linking HIV-positive patients to appropriate support services.

1.1.3.3 HIV Care and ART Utilization in Washington, D.C.

Historically, Washington, D.C., like several other jurisdictions, has had limited success in ensuring that clients newly diagnosed with HIV are routinely and rapidly linked to a primary HIV “medical home.” In 2006, before the HIV testing scale up, only approximately 50% of persons newly diagnosed with HIV were linked to care, as evidenced by a baseline CD4 cell count within six months of diagnosis reported to HIV surveillance. In contrast, in 2007, approximately 67% of the newly diagnosed HIV cases had laboratory evidence (CD4 cell count or VL) of a first HIV-related medical care visit within six months of the initial diagnosis.

In 2008, Washington, D.C. began to look beyond initial linkage-to-care to continuum-of-care after diagnosis. The number of HIV/AIDS cases reported between 2004 and 2007 increased 20.3% from 1,239 cases to 1,490 cases; data from 2008 are still preliminary but include 1,198 reports to date. Sixty-seven percent (67%) of cases had a first CD4 cell count, percentage or VL reported within three months of HIV diagnosis in 2008. The median CD4 cell count among newly diagnosed cases increased 57% from 216 cells/µl in 2004 to 340 cells/µl in 2008. Among newly diagnosed AIDS cases, the proportion of late testers decreased from 66% in 2004 to 57% in 2008.

Washington, D.C. supports full access to HIV care and treatment for all HIV-positive residents through a combination of regular Medicaid/Medicare, the Medicaid 1115 waiver, and Ryan White-funded services, including the AIDS Drug Assistance Program (ADAP). However, despite availability of services, use and outcomes of HIV care in Washington, D.C. remain sub-optimal. Preliminary review of ADAP data suggests irregular utilization at client level, with approximately 60% of enrollees picking up medications in a given month. Moreover, a preliminary review of Ryan White-funded, primary HIV-care providers suggests that only between 25-60% of clients in care are on ART, with approximately 50% of those being virally suppressed (unpublished data from ADAP and grantee reports).

Washington, D.C. is starting to address sub-optimal utilization and outcomes through a number of initiatives. First, a mass media information campaign promoting the availability of treatment was instrumental in increasing ADAP enrollment over 50% within 18 months. Since 2007, the HIV/AIDS, Hepatitis, STD and TB administration (HAHSTA) has also conducted treatment promotion through the “It’s Free to Treat Your

HIV" campaign, which is currently being retooled with patient components (emphasizing treatment enrollment and retention) and a provider toolkit (improving patient linkages and outcomes). Second, Washington, D.C. is collaborating with CDC to identify ways to better utilize routine ADAP pharmacy data to identify irregular utilization and build rapid feedback loops to providers. Third, the Ryan White Planning Council and HAHSTA have adopted a "health first" approach, making navigation (for linkage-to-care from positive test), re-capture (bringing persons lost to follow-up back into HIV care), and retention core priorities for all new funding opportunities for 2009-2012.

HIV care in Washington, D.C. is supported by a range of funding sources, to ensure that every individual in need of HIV treatment is provided both primary care and the supportive services necessary to remain in care. HAHSTA directly supports primary medical care programs at nine organizations in Washington, D.C. These primary medical care programs serve as a hub for services, with the responsibility of coordinating the services needed by their clients. In 2008 HAHSTA began implementing a best practice, four Rs (recruitment, recapture, retaining and results) approach to improving the continuum and continuity of HIV care in Washington, D.C. Recruitment or rapid entry to care links individuals who receive a positive HIV test immediately into primary care facilities, and includes special programs, such as:1) Red Carpet Entry to Care services; 2) adult and adolescent healthcare navigation services; and 3) Rapid Entry to Care initiatives. To recapture patients into care, HAHSTA partnered with several funded care providers over three months to bring back into care nearly 900 people known to be living with HIV but found to not have had a CD4 cell count or VL test in the previous six months. For example, Family Medical Counseling Services re-established care with approximately 70% of 450 clients identified as lost to care, often after more than 10 contacts. Finally, retaining people living with HIV into care and ensuring quality results is a critical component of ensuring the continuity and continuum of care. HAHSTA has supported best-practice, outcome-based management of HIV-positive clients in care with affiliated primary care providers. HAHSTA has partnered with funded providers to use E-clinical Works and other electronic medical record (EMR) systems to institute evidence-based best practices such as deploying coordinated care teams, using focused, comprehensive health messaging, and identifying outcomes at every clinical encounter, with the goals of reducing missed medical appointments, increasing efficiency, increasing prophylaxis and increasing viral suppression.

Another specific strategy to increase linkage-to-care in Washington, D.C. is the requirement that all individuals with preliminary positive rapid HIV tests be linked immediately into care. When confirmatory testing is needed, it is performed at the initial medical visit, along with CD4 cell count, VL and other routine tests for HIV-positive individuals.

Most recently, HAHSTA funded Navigator Services, consisting of an Adult Navigator and an Adolescent Navigator, to facilitate the linkage of HIV-positive individuals into care and treatment. This new citywide resource supports the efforts of independent clinicians who have historically diagnosed up to 40% of our new HIV-positive persons. Navigator services provide individualized support to link newly diagnosed and previously identified positives not in care to a medical home for ongoing specialized HIV care and treatment, as well as evaluate these clients for any other social service needs.

1.1.3.4 HIV Care and ART Utilization in the Bronx, New York

Recent data evaluating linkage-to-care in NYC generally, and the Bronx specifically, have shown slightly more robust linkage rates than in Washington, D.C. However, retention in care is comparable. Most recent surveillance data for NYC show that approximately 70% of newly diagnosed persons have laboratory evidence of a first HIV-related medical care visit (demonstrating linkage-to-care) within three months of their initial diagnosis of HIV. The linkage rate in the Bronx is equivalent to that citywide. Continuity of care, defined by evidence of a medical visit (CD4 cell count or VL reported in the HIV/AID Registry) at least every six months, was 54.4% for NYC overall and 49.3% in the Bronx. Among persons initiating care within three months of HIV (non-AIDS) diagnosis, median CD4 cell count was 439 cells/mm^3 citywide and 451 cells/mm^3 in the Bronx. Finally, 69% of persons eligible under Department of Health and Human Services (DHHS) guidelines to receive ART citywide (CD4<350), and 70% of eligible persons in the Bronx, had achieved an undetectable VL within a median of six months after initiation of care.

HIV-related treatment and economic services are widely available for all HIV-positive New Yorkers, regardless of means. A combination of Medicaid/Medicare services, Ryan White-funded services, including the ADAP, Housing Opportunities for People with AIDS (HOPWA), and benefits available through the HIV/AIDS Services Administration of NYC's Human Resources Administration ensure broad coverage to meet treatment and social service needs. Despite this network of support, outcomes of HIV care for New Yorkers including Bronx residents, as described above, indicate some potential for gain.

NYC has undertaken at least two major initiatives to attempt to make headway in these areas. Beginning in December 2009, the NYC DOH will begin funding 27 agencies throughout NYC, including six agencies in the Bronx, for comprehensive coordination of care using Ryan White funds. Coordination of care will include health system navigation, medical case management with treatment adherence, and educational coaching with goals of viral suppression and self-sufficiency for individual patients. Additionally, an expanding Field Services Unit (FSU), created in 2006, stations public health advisors on site at ten tertiary hospitals in the highest prevalence neighborhoods of NYC, as well as at Rikers Island jail, a large correctional facility. FSU staff assists index patients and physicians with partner elicitation, partner notification and testing of partners. FSU staff also work to recapture patients who have fallen out of medical care.

1.1.4 Adherence to ART and Viral Suppression in the United States, Washington, D.C. and the Bronx

Non-adherence to ART is common among patients in the United States, with the percentage of prescribed doses taken estimated between 60% to 70% (Simoni, Pearson et al. 2006). Somewhat higher adherence levels are reported over shorter recall periods (Mugavero, Ostermann et al. 2006; Lazo, Gange et al. 2007). However, levels of adherence to ART as high as 80% to 95%, depending on the type of regimen, may be necessary to achieve and maintain maximal viral suppression and optimize clinical outcomes (Bangsberg 2008). Incomplete adherence has been among the most important factors related to virologic failure, the emergence of drug resistance, and ultimately

progression of HIV disease to AIDS and death (Lazo, Gange et al. 2007; Lima, Geller et al. 2007; Horberg, Silverberg et al. 2008). Non-adherence also contributes to the transmission of drug-resistant HIV strains (Sethi, Celentano et al. 2003).

Recently introduced ART regimens are less toxic, more tolerable and simpler, particularly if available in fixed-dose combinations (ARV treatment guidelines). These include ART regimens with new agents from established classes and new classes of ART that have been recently shown to be effective in suppression of resistant viral strains (Steigbigel, Cooper et al. 2008; Markowitz, Nguyen et al. 2009). Multiple clinical trials have shown the ability to achieve suppressed viral replication with the use of such regimens in a large proportion of patients. However, suboptimal adherence remains common, and young age, active drug and alcohol use, and depression are associated with poor adherence to ART (Levine, Hinkin et al. 2005; Lazo, Gange et al. 2007; Horberg, Silverberg et al. 2008). In clinical practice, adherence is typically assessed by self-report (Simoni, Kurth et al. 2006) and can be corroborated by the use of MEMS caps, pill counts, biologic markers (*e.g.*, plasma drug levels), pharmacy refill data, (Bangsberg 2008) and ultimately by the measurement of HIV VL.

In addition to optimizing ART regimens, effective approaches to improving ART adherence include ART-readiness training, adherence-related case management, various forms of counseling, pharmacist-based support, telephone support, reminder devices, and directly observed therapy (Bartlett 2002; Bangsberg 2008). A recent meta-analysis of randomized behavioral interventions conducted mostly in the United States found that participants receiving adherence interventions were 1.5 times as likely to report 95% adherence and 1.25 times as likely to achieve undetectable VL than controls (Simoni, Pearson et al. 2006). Improving ART adherence requires a combination of methods appropriate to the patient and clinical setting. Among the most important predictors of ART adherence are engagement in medical care and continuous adherence with medical visits (Aberg, Kaplan et al. 2009). Alterable factors known to impact adherence, such as mental health problems, active substance use, homelessness, inadequate level of social/economic support (Magnus, Kuo et al. 2009), as well as the therapeutic relationship between patient and provider should be addressed proactively (Bakken, Holzemer et al. 2000; Bangsberg 2008).

Both in the Bronx, NY and Washington, D.C., the health departments consider adherence support services to be an essential component of a comprehensive HIV-care package. These health departments monitor trends in adherence by reviewing HIV VLs and CD4 cell count data reported to HIV surveillance systems. For example, HAHSTA approaches the promotion of treatment adherence as a responsibility shared by all providers or services including physicians, physician assistants, nurses, pharmacists and other clinical care providers. Upon enrollment, and periodically thereafter, each client's records are reviewed for evidence of viral suppression as well as assessed for the need for specialized services to improve the ability of the client to take HIV medications effectively and remain consistently in care. A wide range of services is available for those in need, including linkages to mental health and substance use programs, education on HIV, programs designed to assist with the necessities of daily living, and ongoing individual or group psychosocial and peer support.

1.1.5 Prevention for Positives

Meta-analyses show that prevention efforts in HIV-positive individuals (Prevention for Positives) (Crepaz, Lyles et al. 2006; Johnson, Carey et al. 2006), can be effective in decreasing risky behaviors. However, most Prevention for Positives interventions have been assessed only in research contexts and require substantial investments of staff and resources to deliver with fidelity, suggesting that population-level uptake may not likely follow. Sexual risk assessments and risk-reduction counseling are not routinely performed in many HIV clinical settings, and even when they are, quality and consistency are variable (Metsch, Pereyra et al. 2004). Information and communication technologies (ICT) may facilitate scale-up of prevention interventions, as they utilize the client's time for self-monitoring, deliver content with fidelity, can include multiple languages, and, once programmed, can be used for multiple computers with marginal cost beyond cost of the hardware. A recent meta-analysis found that these tools are efficacious for reducing HIV transmission risk (Noar, Black et al. 2009). A recent study by Gerbert et al. found that computer-delivered "video doctor" counseling to 960 HIV-positive patients was associated with reduced transmission risk behavior (Gilbert, Ciccarone et al. 2008). CARE+ Prevention is one example of an intervention that may be effective in decreasing sexual behaviors most likely to transmit HIV. Other studies have shown that Prevention for Positives interventions are less effective with patients with ongoing substance use and mental health conditions.

In Washington, D.C. HAHSTA is in the process of developing a Prevention for Positives initiative that includes consistent and repeated delivery of prevention interventions by clinicians to people living with HIV/AIDS (PLWHA) in care and treatment settings. The strengths of this clinic-based approach include linking the prevention of HIV transmission to the treatment of HIV, offering repeated opportunities to intervene in high-risk behavior, and serving as a source of information, support and linkage to needed services for the HIV-positive individual. The five areas of focus for this initiative will be: 1) social support for disclosure; 2) treatment adherence for viral suppression; 3) mental health; 4) substance use; and 5) general prevention interventions, such as distribution of condoms.

The NYC DOHMH has begun a concerted effort to prioritize prevention activities with HIV-positive individuals. Beginning in 2007, the department began funding 19 agencies to conduct 16 different behavioral interventions that have clear evidence of achieving behavior change. While many of these interventions work with HIV-positive and high-risk HIV-negative individuals, six agencies were funded to conduct interventions that focus primarily on HIV-positive individuals, such as "Healthy Relationships." Partner services activities as described above, operating primarily through the FSU and the Contact Notification Assistance Program, also focus directly on HIV-positive individuals as well as soliciting, notifying and testing partners of index patients. As part of their routine work, FSU staff members also assist in linkage and health navigation activities, discuss risk reduction, and provide free condoms, along with condom education as needed.

In 2009, the NYC DOHMH introduced a CDC-developed training course for NYC healthcare providers focusing exclusively on HIV prevention for positive patients called

“Ask, Screen, Intervene.” The course was offered quarterly and will continue in 2010. The Department further developed a comprehensive prevention-for-positives protocol for clinicians and non-clinician CBO staff, which will be made available in 2010. Finally, both the Ryan White comprehensive coordination of care program and a planned new funding stream will increase dedicated prevention activities for HIV-positive persons beginning in 2010.

1.1.6 Provider and Patient Attitudes Towards ART Use

Mathematical models that have addressed the issue of use of ART for prevention of HIV transmission have largely concluded that ART would be most effective for HIV prevention if used in the largest proportion of HIV-positive populations (Granich, Gilks et al. 2009). This implies that such treatment would be provided to HIV-positive individuals irrespective of their eligibility for ART initiation, as detailed in the available national or international guidelines. To date, such guidelines (Health and Human Services (HHS) guidelines, WHO ART guidelines) have been largely based on evidence supporting benefits versus risks of the use of ART for the individual with HIV infection, rather than based on prevention considerations.

Providers have largely utilized such recommendations to guide their decisions for when to initiate or use ART in HIV-positive individuals, aiming to provide patients in their care with the opportunity to achieve optimal outcomes for their individual health and well-being. Use of ART in HIV-positive individuals to benefit their partners and the community is a concept that goes beyond this framework and places providers in a dilemma, should the well-being of their patients be affected adversely by therapy whose aim is to reduce potential transmission to others in the community.

Similarly, from the patient’s perspective, ART use has been largely perceived to provide individual benefits. Thus, in making decisions regarding initiation of ART, patients make their assessment based on information provided to them regarding benefits and risks to their own health rather than based on potential effect on transmission of HIV to others. Adding reduction in infectiousness as a consideration will need to be weighed and patient knowledge and attitudes regarding such use carefully evaluated.

1.1.7 Financial Incentives

The use of financial incentives (FIs) to modify behaviors has become increasingly common in settings both within and outside the healthcare sector. Outside the healthcare setting, experiments have ranged from conditional cash transfers to alleviate poverty among low socio-economic status (SES) individuals in NYC to efforts to improve school performance within NYC, Chicago and Washington, D.C. Within the healthcare setting, in recent years, there has been increasingly widespread use of FIs among insurers and employers who see this as an approach with great promise to help individuals make better tradeoffs between unhealthy behaviors (that have immediate gratification) in favor of healthier behaviors (that have delayed benefits). For example, NYC is evaluating the effectiveness of FIs in achieving normal HbA1c levels in individuals with diabetes. In fact, such efforts have now been shown to be effective in changing health behavior and improving health outcomes in a variety of clinical contexts.

The interest in interventions aimed at improving patient behaviors is in part due to recognition that unhealthy behaviors may be a bigger contributor to poor health and premature mortality than inadequate healthcare delivery. Experts have estimated that unhealthy behaviors, such as medication non-adherence, smoking, poor diet and sedentary lifestyles account for as much as 40% of premature mortality in the United States, whereas deficiencies in healthcare delivery account for only 10% of premature mortality (Schroeder 2007). Many factors such as the social and structural environment, public policies, genetics and provider access and quality affect the rate of such behaviors. However, individuals' behavioral choices are clearly a central driver and are potentially more amenable to incentives.

Early evidence on incentives suggests that incentive-based approaches can be highly effective in two areas in particular: (1) changing short-term health behaviors related to preventive services that involve a limited number of visits, and (2) reducing the use of addictive substances. Examples of effectiveness of incentives in increasing use of preventive services include studies that have shown increases in rates of follow-up for abnormal pap smears (Marcus, Kaplan et al. 1998), postpartum visits by adolescents (Stevens-Simon, O'Connor et al. 1994), TB test reading (Malotte, Rhodes et al. 1998), and the rate at which IV drug users received all three doses of hepatitis B vaccine (Seal, Kral et al. 2003). The evidence that such approaches are effective in reducing the rate of use of addictive substances, such as cocaine (Higgins and Silverman 1999; Lussier, Heil et al. 2006) and nicotine in the short term, (Donatelle, Hudson et al. 2004; Volpp, Gurmankin Levy et al. 2006), as well as for short-term weight loss (Jeffery, Thompson et al. 1978; Jeffery, Gerber et al. 1983; Finkelstein, Linnan et al. 2007), suggests that financial rewards designed to incent long-term changes in behavior could be applicable to a wide range of other health behaviors in which frequent reinforcement and longitudinal follow-up are necessary.

However, evidence of effectiveness of incentives is not limited to the areas cited above. A review of 11 randomized trials of FIs found that in 10 studies, FIs promoted adherence better than any tested alternative, leading to better blood pressure control, better appointment attendance and higher immunization rates (Giuffrida and Torgerson 1997). More recent reviews of economic incentives found that a wide range of incentive mechanisms are effective in changing behavior (Kane, Johnson et al. 2004; Sutherland, Christianson et al. 2008).

Another reason to consider further testing of incentive-based approaches is that many highly efficacious medical tests, treatments and medications have limited effectiveness due to patient behaviors. For example, by one year after having a myocardial infarction, nearly half of patients prescribed cholesterol medications have stopped taking them (Jackevicius, Mamdani et al. 2002). Similarly, the effectiveness of HIV medications would be much higher if rates of adherence increased to the point where benefits demonstrated in clinical trials could also be seen in high rates of effectiveness in communities across the United States.

More recent research has found that FIs double the rate of attendance at and completion of a smoking-cessation program, triple long-term smoking cessation rates and substantially increase medication adherence among patients on warfarin. For example, in

a two-arm RCT of 878 employees at General Electric (GE), (Volpp, Troxel et al. 2009) incentives of up to $750 resulted in quit rates at nine-12 months triple those in the control group (p<.001). Other studies have examined the use of incentives for weight loss (Volpp, John et al. 2008) and medication adherence (Volpp, Loewenstein et al. 2008).

Two studies have been published in which FIs were used for ART adherence. In both cases, the payments made a significant improvement in ART adherence (Rigsby, Rosen et al. 2000; Rosen, Dieckhaus et al. 2007). The findings that FIs can help modify difficult-to-change behaviors whose cessation requires ongoing reinforcement, such as quitting alcohol or cocaine use, are particularly striking and suggest that a well-designed incentive program can succeed in changing behaviors in clinical contexts in which many other approaches have been unsuccessful (Bigelow and Silverman 1999; Donatelle, Prows et al. 2000; Higgins, Wong et al. 2000).

The amounts we selected for use as FI in this study are based on the following principles: 1) review of relevant literature and experiences, 2) input from investigators with knowledge of target communities and populations, 3) input from community groups/advisors from the study communities and elsewhere, and 4) input from key staff in departments of health and public health entities in the relevant communities. Based on these factors, the amount of $125 for successful linkage-to-care, and a maximum of $630 for viral suppression throughout the study period were thought to be appropriate amounts for evaluation in this study.

With regard to the sustainability of any effect caused by the FI after it is removed, a recent study on incentives for smoking cessation (Volpp, Troxel et al. 2009) demonstrated that the ratio of tobacco cessation among incentive to control group participants at nine-12 months (2.9) remained significant six months after cessation of incentive payments (ratio of quit rates was 2.6 at 15-18 months). In other work, shorter durations of incentives have been associated with higher relapse rates. It is of interest that based on the findings of the latter study on smoking cessation, GE announced plans to implement a program for FIs for smoking cessation nationally for all its 152,000 employees.

Studies involving FIs have focused on both process measures (the behavior in question, such as medication adherence) and outcomes (smoking cessation). There are several precedents for the use of outcomes (*e.g.,* VL) to assess the effect of FIs on medication adherence. Several studies that used FIs to improve adherence with medication (*e.g.,* warfarin or insulin) have measured biologic outcomes to evaluate adherence (*e.g.,* INR, HbA1c) (Volpp, Loewenstein et al. 2008). It is essential that FIs be provided in such a way that they yield a verifiable outcome. Because lack of VL suppression most often results from lack of adherence, the use of FIs to encourage ART adherence is plausible from both a medical and a behavioral-economics perspective. The relative effectiveness of focusing incentives on process versus outcomes is generally unknown and is ultimately an empirical question. In Washington, D.C., a demonstration project indicated that a focus on outcomes (rather than process) has been associated with improvements in the areas of HIV prevention and care (Hadar, personal communications). Specifically, rewarding HIV-care organizations for every three patients they successfully re-linked to care, regardless of the effort expended, yielded positive results. In this study, FIs will

focus on outcomes – the completion of care visits for the Linkage-to-Care component and in the Viral Suppression component.

In contingency management studies, non-monetary rewards have often been used in place of monetary rewards because of concerns that cash might increase the likelihood of substance use. However, studies have not supported such concern. In the AIDS Link to Intravenous Experience (ALIVE) study, ethnographers trailed the drug users after they received the money to assess where they went. They found that participants mostly went to food establishments after they received the money. There was no difference in the rates of death caused by overdose in participants when analyzed by days after study visit or amount of reimbursement (Vlahov, Tang et al. 2000).

1.1.8 HIV Surveillance Data

Since 1982, all 50 U.S. states and Washington, D.C. have reported AIDS cases to the CDC using a standardized case report form (CRF) (Schneider, Whitmore et al. 2008). In 1994, the CDC integrated national reporting of HIV with AIDS case reporting, at which time 25 states with confidential, name-based HIV reporting started submitting case reports to the CDC. Over time, additional states implemented name-based HIV reporting and started reporting these cases to CDC. By April 2008, all states and Washington, D.C. had implemented name-based HIV reporting and were reporting cases to the CDC.

HIV case surveillance data are collected as part of routine HIV surveillance, as mandated by state or local laws or regulations. Named reporting is required for all diagnoses of HIV and AIDS and all HIV-related illness according to the case definition. Information is collected on demographic characteristics of persons diagnosed with HIV, transmission risk factors, facility of diagnosis, diagnostic tests (*e.g.*, positive Western Blot (WB) tests for HIV antibody) and death. All areas require laboratory reporting of VL and CD4 cell count values. While not all areas require reporting of all values of VL or CD4 cell count (some limit reporting to detectable VL and/or CD4 <200 or <14%), all areas collect the first results of such tests after HIV diagnosis through chart review or laboratory reporting, where all values are reported through voluntary laboratory reporting. Both intervention communities require reporting of all laboratory results. Some areas such as NY also require reporting of all HIV genotypes.

These population-based registries are continuously updated with new, de-duplicated diagnoses and laboratory results. Incoming diagnostic WB and VL reports from providers and laboratories that cannot be matched to an existing registry record initiate a field investigation to confirm the case, date and disposition of diagnosis and collect other data required for surveillance. Data are also obtained through matches with other disease registries, the state and local death registries, the National Death Index and the Social Security Death Master File. Assessments of duplicate cases occur both on the state and national level (potential duplicates are identified based on soundex code [a phonetic algorithm for indexing names by sound, as pronounced in English] and selected demographic characteristics), while elimination of such cases occurs at the state level.

HIV reporting and laboratory reporting requirements allow virtually complete surveillance of diagnoses of HIV, stage of disease at HIV diagnosis, and number of

people diagnosed and presumed to be living with HIV. Using CD4 cell count and VL test ordering as proxy measures for initiating HIV primary care, after the first positive WB test, allows for calculation of the time between diagnosis and initiation of care. Frequency of visits, regularity of U.S. DHHS-recommended laboratory monitoring, and estimates of the proportion of cases eligible for ART are now possible with CD4 cell count and VL result reports. These laboratory indicators also allow estimates of the number and characteristics of cases not in care. Clusters of highly resistant HIV will be detectable when state genotype reporting systems become operational.

The CDC has developed a data entry and reporting system, the Program Evaluation and Monitoring System (PEMS), to strengthen monitoring and evaluation of HIV-prevention programs. PEMS is used by health departments and CBOs funded through CDC HIV-prevention cooperative agreements. PEMS allows grantees to collect agency data, community planning data, program plan data, and client-level data. This assures a comprehensive set of standardized variables are available.

Some areas have implemented additional surveillance activities, such as behavioral risk factor surveys and supplemental surveillance activities supported by the CDC (*e.g.*, incidence surveillance). The National HIV Behavioral Surveillance (NHBS), which takes place in 25 cities, can provide information on testing among various population groups including among MSM, a special emphasis group in this project. Where available, the Morbidity Monitoring Project (MMP, a 26-site study) may provide information on treatment among those in care. In addition, data are collected on testing conducted in all sites receiving funding from the CDC and in additional sites as required by state or local laws or regulations.

1.2 Purpose

The main purpose of this study is to assess the feasibility of a community-level test, link to care, plus treat strategy in the United States. The study will involve evaluation of the feasibility of some components and the effectiveness of others.

The study will assess the following:

- The feasibility of expanding HIV testing via social mobilization and universal offer of HIV testing in EDs and inpatient hospital admissions
- The feasibility and effectiveness of facilitating the linkage of HIV-positive patients to HIV care sites
- The feasibility and effectiveness of different strategies for assuring maximum initiation of ART for clinically eligible patients according to current guidelines
- The feasibility and effectiveness of different strategies for promoting high adherence to ART and maintenance of VL suppression
- The effectiveness of a computer-assisted program for Prevention for Positives
- Patient and provider attitudes towards the initiation of ART in early HIV disease

The primary outcomes of this study's package of interventions will be determined through measurement of change from baseline (at the initiation of the study) over the duration of the study (see study timeline) in key parameters in two intervention communities in the United States. Observations in four non-intervention communities will help assess the influence of current trends in HIV testing and care expansion in the United States.

This study will serve as a proof-of-concept, formative study. It will provide key information to guide the design and anticipate the costs of a future large, randomized, community-level clinical trial of full implementation of a test-and-treat strategy in the United States. This study uses innovative approaches including: 1) a community focus; 2) multi-component strategies that include behavioral and biomedical interventions; 3) the use of routinely reported HIV surveillance data to determine key outcomes; and 4) partnership with both local DOHs and the CDC. Findings from this study could also inform test-and-treat efforts in other developed countries with epidemics similar to that in the United States.

HPTN 065 will help generate estimates for some of the parameters that, taken together, will describe an "index of participation." Such an index of participation (upon which the test-and-treat strategy would ultimately depend) is based on a cascade: the percentage of HIV-infected persons tested and identified within a community, the percentage of such individuals linked-to-care, and the percentage who initiate and remain adherent to ART and maintain ongoing viral suppression. For example, even with optimistic assumptions (80% of infected persons tested; 80% linked to care, and 80% adherence to ART) the index of participation would be 51%. These parameters have, to date, been treated as assumptions in test-and-treat models. The mathematical model described by Granich et al. assumed that all adults would be tested annually with a 100% sensitive, 100% specific test; that all HIV-infected persons would enter care and start ART as soon as they were diagnosed irrespective of HIV disease stage; that there would be a decrease of 99% in their infectiousness and that only 1.5% of subjects would discontinue ART each year, for an unrealistic index of participation of 98.5% (Granich, Gilks et al. 2009).

HPTN 065 will examine the feasibility and relative effectiveness of various interventions that individually aim at optimizing each component related to the index of participation. These insights together with data on the magnitude of the effect of ART on infectiousness (to be obtained from other studies), could be incorporated into future models that aim to determine the potential effect of a test and treat strategy on incidence in various communities in the United States.

1.3 Rationale

1.3.1 Rationale for a Test-and-Treat Approach

More than one million persons in the United States are currently living with HIV and about 21% are unaware of their HIV-positive status. The HIV epidemic in the United States affects specific subpopulations and localized "hot spots." Individuals may be at risk for HIV because of their own risky behaviors or the high prevalence of HIV among

persons whom they encounter, or a combination of both. Risk may be compounded by high rates of undiagnosed HIV infection within these same communities.

In 2006, there were an estimated 56,000 new HIV infections in the United States; a number that has been approximately stable for the past decade (Hall, Song et al. 2008). Certain municipalities and communities in the United States bear the brunt of the HIV epidemic. This includes certain geographic areas in the United States and specific subsets of individuals defined by behavior and/or by race/ethnicity. The data from 2006 indicate that MSM and AAs are most severely affected. For example, in Washington, D.C., three percent of the population is HIV-positive. National Health and Nutrition Examination Survey (NHANES) data indicate that HIV prevalence among AAs age 40-49 is nearly 3% among women and 4.5% among men (McQuillan, Kruszon-Moran et al. 2006).

The central factors that drive the HIV epidemic in the United States are: (1) the number of individuals unaware of their HIV infection who continue behaviors likely to transmit HIV; (2) the frequency of late diagnosis of HIV infection; and (3) delay in access to care, delay in initiation of ART and suboptimal adherence, leading to failure of sustained viral suppression. Approximately 40% of persons are diagnosed with AIDS within a year of their first HIV-positive test (Valdiserri, Holtgrave et al. 1999; Castilla, Sobrino et al. 2002; (CDC) 2009), too late to realize the full benefit of advances in HIV management. Persons with undiagnosed HIV infection may also unwittingly transmit HIV to partners. While they represent less than one quarter of the entire HIV-infected population, persons with undiagnosed HIV infection account for more than 50-70% of new sexually transmitted infections in the United States and are 2.5 times more likely to transmit HIV than persons who are aware they are infected (Marks, Crepaz et al. 2006). Evidence also indicates that many diagnosed individuals delay or fail to engage in HIV care (Torian, Wiewel et al. 2008), and for those who have initiated ART, retention in care and adherence to ART is suboptimal (Lazo, Gange et al. 2007; Mugavero, Lin et al. 2009).

Since the beginning of this decade, evidence has accumulated that higher plasma HIV RNA is associated with increased risk of HIV transmission (Quinn, Wawer et al. 2000; Fideli, Allen et al. 2001). More recent data suggest that use of ART is associated with a substantial decrease in rate of HIV transmission from the HIV-infected to uninfected sexual partner (Bunnell, Ekwaru et al. 2006; Sullivan, Kayitenkore et al. 2009). The availability of various ART regimens capable of suppressing viral replication in various subsets of HIV-positive individuals offers the opportunity to determine the potential effectiveness of use of ART on HIV incidence in a community. While various models suggest that such a strategy may be associated with a successful impact on the trajectory of the HIV epidemic, empiric data are needed to confirm this hypothesis.

It is widely anticipated that if a TNT approach is to achieve its desired impact, it would require the following ambitious effort: universal HIV testing efforts; prompt and effective linkage of all individuals with HIV infection to HIV care; timely initiation of ART; and sustained suppression of HIV replication in all HIV-positive individuals. It is evident that assessment of such an intervention requires a deeper understanding of each of its components.

Thus, we will examine five study components highly relevant to the TNT approach. Four study components are interventions and one is a survey. The four study interventions to be assessed include: expanded HIV testing, linkage from HIV testing sites to HIV care sites, viral suppression with use of ART, and a computer-delivered intervention to achieve safer behaviors among HIV-positive individuals. In addition, surveys for both HIV-positive persons and HIV-care providers will be done to determine knowledge and attitudes regarding early use of ART and financial incentives.

1.3.2 Rationale for Choice of Intervention Communities

This study, Test, Link-to-Care, Plus Treat, will assess five components in two communities in the United States, the Bronx, NY and Washington, D.C. These communities were selected due to: (1) the severe impact of the epidemic on these communities (NYC has the largest number of cases of HIV of all cities in the United States and Washington, D.C. has the highest HIV seroprevalence), and (2) the substantial efforts these cities have already made to improve HIV diagnosis, facilitate linkage-to-care, and support adherence.

NYC has the oldest, largest and most heterogeneous epidemic in the Western World. By June 30, 2008, a cumulative total of 207,687 persons in NYC had been diagnosed and reported with HIV. 43,344 (20.8%) of these persons were residents of the Bronx at the time of diagnosis. A cumulative total of 100,378 persons with HIV have died over the course of the epidemic (48.3% of cumulative diagnoses). There have been 20,052 deaths (19.9% of cumulative deaths citywide) among Bronx residents. In 2007 HIV was the third leading cause of death in persons under age 65.

As of June 30, 2008, there were 104,234 persons diagnosed, reported and living with HIV in NYC. 25,622 (24.5%) were residents of the Bronx. Citywide, the rate of new HIV diagnoses was 46.8/100,000 population. The diagnosis rate in the Bronx was 62.4/100,000 population. The Bronx rate was second to that of Manhattan, which reported a diagnosis rate of 70.5/100,000 population. Citywide, 26.1% of newly diagnosed persons have already progressed to AIDS at the time they first learn they are infected with HIV (concurrent diagnosis of HIV and AIDS). The borough with the highest proportion of concurrent diagnoses (27.3%) is the Bronx.

Washington, D.C. is in the midst of a generalized HIV epidemic. At the end of December, 2007, there were 15,120 persons diagnosed and reported living with HIV in Washington, D.C. Between 2003 and 2007, there were 7,432 new HIV/AIDS cases reported, bringing to more than 3% the proportion of Washington, D.C.'s adult population diagnosed and living with HIV. It is estimated that one-third to one-half of infected persons may be unaware of their HIV infection (DC NHBS data). All wards in Washington, D.C. (with the exception of Ward 3) have prevalence rates above the Joint United Nations Programme on HIV/AIDS (UNAIDS)/WHO threshold for a generalized HIV epidemic. More than two-thirds of new HIV diagnoses are in people over age 30 years. More than 7% of Washington, D.C. residents age 40-49 and 5.2% of D.C. residents age 50-59 years are diagnosed and living with HIV. The rate of HIV is highest among black males (6.5%), but 3.0% of Hispanic males and 2.5% of white males and black females are estimated to be living with HIV. In examining the most recent trend

data on HIV (not AIDS) and newly reported AIDS cases, mode of transmission trends are changing. Heterosexuals account for over 37% of newly reported HIV cases and 31.5% of newly reported AIDS cases, followed by MSM (25.8% and 29.0%, respectively) and injection drug users (13.2% and 21.7% respectively). With the advent of ART, deaths due to AIDS have significantly decreased over the last 15 years. HIV/AIDS, however, is still the leading cause of death in Washington, D.C. residents age 25-44 (Washington 2009). In 2007, there were 138 deaths from AIDS or AIDS-related complications.

1.3.3 Rationale for Choice of Interventions

The study will focus on expansion of HIV testing in hospital emergency departments and inpatient units. The rationale for expanding the offering of HIV screening in EDs emanates from numerous studies in urban EDs that indicate a 0.7% - 1% yield of new HIV diagnoses among those tested. Many patients with HIV infection visit EDs, but remain undiagnosed. For example, of all patients with new HIV diagnoses in South Carolina from 2001-2005, 73% had previously visited healthcare facilities, but were not tested for HIV; 79% of these visits were to an ED. Although many EDs in both the intervention and non-intervention cities have initiated HIV screening, most ED programs are unable to offer screening 24/7, and many are unable to test all patients who agree to be tested.

A similar yield of new HIV diagnoses might be expected from testing hospital admissions. Data from the NY Health and Hospitals Corporation (the organization that operates 11 public hospitals in NYC where expanded HIV screening began in 2006), and from Boston Medical Center's HIV Inpatient Testing Service, indicated an HIV positivity rate of 2.5% - 5% among inpatients who agreed to be tested (selectively thus which may or may not reflect the prevalence of HIV infection among all inpatients, including those who refused HIV testing or were not offered testing). No data exist on the yield of routine HIV screening of all hospital admissions. This approach was traditionally a significant component of syphilis screening efforts. Similarly, this approach may be a cost-effective way to expand HIV screening and to ensure that no HIV-positive patient who encounters the healthcare system remains undiagnosed. This component of the HPTN 065 study (*i.e.* supporting expanded ED- and hospital-based offering of testing) will be a supplement to ongoing testing efforts in these communities.

The study will also aim to use social mobilization to increase HIV testing volume and frequency. Because of the bimodal nature of the HIV epidemic in the Bronx and in Washington, D.C. (generalized, especially among AAs, and concentrated among MSM) the study will also include another strategy in the testing component. Through social mobilization, focused messages will be created for the intervention communities on the importance of getting tested for HIV and knowing one's status (tested in past year). In addition, specific messages will be created to promote more frequent retesting for sexually active MSM—preferably twice a year, but at a minimum, annually.

In this study, we will not aim to identify acute infections. Unpublished data from the CDC's Acute HIV Infection Study demonstrate that HIV retesting as described above is considerably more cost-effective than Nucleic Acid Amplification Testing (NAAT) screening for acute infection.

The study will utilize FIs to promote linkage of HIV-positive individuals from testing to care sites and to achieve and maintain viral suppression. As described in Section 1.1.7 FIs hold great promise for achieving the target behaviors. The study will use a site-randomized approach to compare the effectiveness of FIs to the standard of care (SOC) in achieving these two goals.

Some TNT models assume that all HIV-positive individuals will initiate ART irrespective of their CD4 cell count. Evidence from observational studies supports earlier initiation of ART, with the goal of delaying HIV disease progression and reducing mortality rates (Kitahata, Gange et al. 2009; Sterne, May et al. 2009; When to Start Consortium 2009). The availability of safer, simpler, more tolerable and potent ART regimens has reduced, but not eliminated, some concerns about the development of long-term toxicities and complications among HIV-positive patients (Justice 2006; Friis-Moller, Reiss et al. 2007) and reduced concerns about development of antiretroviral resistance (Phillips, Leen et al. 2007; von Wyl, Yerly et al. 2009). However, in this study, the provision of ART will be guided by the prevailing DHHS Guidelines for the Use of ART in HIV-Positive Adults and Adolescents (Panel on Antiretroviral Guidelines for Adults and Adolescents 2009). Providers at participating sites will receive educational trainings on best practices in HIV management, per current guidelines, and will be encouraged to promptly and appropriately evaluate their patients for eligibility for ART, maximize ART initiation when indicated per current guidelines, and switch regimens when indicated.

Lastly, while TNT models have not directly addressed the contribution of efforts to promote safer behaviors to reduce transmission from HIV-positive persons, unsafe sex behaviors by HIV-positive persons are a major factor contributing to ongoing HIV transmission. Therefore, in this study, we will evaluate an individually randomized computer-delivered prevention-for-positives component to reduce unsafe sexual and injection-drug using behaviors. In this manner, we will gather feasibility and effectiveness data to inform about the value of such an intervention for future studies.

1.3.4 Rationale for Use of Surveillance and Routinely-collected Data

The study will utilize routinely reported HIV surveillance data collected in the United States to determine the effects of the various interventions. The United States is fortunate to have a robust HIV surveillance system that was established at the advent of the epidemic in the early 1980’s. Over the ensuing years, this system has been expanded and refined. At present, in many communities, it includes data on all positive HIV tests, demographic and risk behavior data on all those found to be HIV-positive, collection of all CD4 cell count and HIV RNA results, and information on AIDS-related deaths, among other data elements. Thus, this system offers a remarkable opportunity to assess various site-level or community-level interventions.

The evaluation of each of the present study’s components, and the feasibility of use of existing HIV surveillance data, will be critical to the design, implementation and evaluation of a large, future, community-focused test-and-treat study, with HIV incidence as the endpoint of interest. The collaborative process utilized in the development of this study that includes partnership among the National Institutes of Health (NIH)-funded

HPTN, the CDC, and departments of health is noteworthy. In addition, the engagement of diverse settings, and organizations, including HIV testing sites, HIV care sites, health facilities, providers, CBOs, advocates and leaders is critical to its success and necessary for future large studies of TNT strategies.

1.4 Protocol Structure

This protocol describes the five study components: 1) expansion of HIV testing, 2) enhancing the linkage of HIV-positive individuals to HIV care sites, 3) maintenance of VL suppression, 4) use of a computer-delivered intervention to decrease high-risk sex behaviors (Prevention for Positives), and 5) provider and patient surveys to gather data on knowledge, attitudes and practices regarding ART and ART adherence (potentially starting at higher CD4 cells counts) and FIs. Sections 2-6 are devoted to the objectives; study design, interventions, and procedures; data and statistical analysis; and ethical issues of each study component. Sections 7-8 contain information applicable to all study components (HIV surveillance data, data sources and administrative/operational issues).

2.0 EXPANDED HIV TESTING

2.1 Study Objectives for Expanded HIV Testing

The intervention communities have committed substantial programmatic funds to expanded testing. The study will aim for expansion of HIV testing efforts in the intervention communities building on ongoing efforts in these communities. The study will focus on two elements: 1) social mobilization and 2) universal offer of HIV testing and counseling in hospital EDs and inpatient units.

True evaluation of the efficacy of increased testing and its impact on detecting undiagnosed infections could only be achieved through a high-quality random sample, representative of the general population, to obtain information about HIV status and knowledge. The expense and scale of such an effort is beyond the scope of a feasibility study. Thus, we have adopted the planned ecological approach of monitoring process and outcome measures to capture increase in HIV testing and change in number and characteristics of newly detected infections throughout the course of the study.

The **primary feasibility objective**s for expanded HIV testing, through a combination of focused and enhanced social mobilization efforts, will be measured by assessing the following trends in the intervention cities:

- Volume of testing in publicly funded testing sites
- Number of newly identified HIV-positive individuals
- Site of diagnosis for newly identified individuals
- Initial CD4 cell count results after first positive HIV test (to assess proportion eligible for ART at different initiation thresholds)
- Proportion of newly identified individuals with HIV concurrently diagnosed with AIDS
- Proportion of population tested for HIV in the last year (local behavioral surveys)

Because expanded testing initiatives are already underway in numerous jurisdictions, the magnitude and trends of changes in the intervention cities will be compared to similar measures in the non-intervention cities, in an attempt to account for secular trends independent of the study components.

- The following variables in the intervention cities will be assessed:
  - Costs of enhanced testing activities in EDs and for hospital admissions
  - Costs of social mobilization
  - Intensity of social mobilization activities (content, number, timing of activities, and type of activity, such as TV, radio, brochures, posters, community leaders, events, *etc*.)

The protocol team will track all of the expenses listed above. Funds for these items will be held centrally and only disbursed when line item bills are presented. Funds provided by HPTN 065 will be a small supplement to DOH program efforts in Washington, D.C and the Bronx, NY. The protocol team will request costs of existing activities from the DOHs.

The **primary feasibility objectives** for expanded HIV testing in EDs and for hospital admissions will be measured by determining the following:

- Proportion of visits to EDs and admissions to hospitals who receive an HIV test (including all individuals permitted by local or state law to consent for HIV testing in the study communities)
- Number of patients tested in EDs newly identified as HIV-positive and their demographic characteristics
- Number of patients tested during hospital admissions newly identified as HIV-positive and their demographic characteristics
- Number of tested patients identified with previously diagnosed HIV who are not in care
- Cost of support for additional staff and HIV tests

2.2 Design for Expanded HIV Testing

This study component will aim to substantially expand HIV testing activities in the two intervention communities. The Expanded HIV Testing component is a descriptive ecologic study. For this component of the study, outcomes will be evaluated through routinely reported surveillance and process data. Because extensive interventions to expand testing have already been undertaken, the study team will catalogue the existing activities in the intervention communities as they relate to social mobilization, enhanced testing and linkage activities, in collaboration with departments of health in the two communities.

2.3 Study Population for Expanded HIV Testing

The target age population for the social mobilization element of expanded HIV testing will be all persons age 13 and older in the Bronx, NY and Washington, D.C., with special emphasis on MSM.

The target population for the implementation of universal offer of testing in EDs and during hospital admissions will be individuals permitted to consent for HIV testing according to New York State or Washington, D.C. law.

2.3.1 Inclusion Criteria

The inclusion criteria for the universal offer of HIV testing are the following:

- Individuals who are permitted to consent for HIV testing according to New York State or Washington, D.C. law
- Capacity to understand and provide consent for HIV testing
- Admission to a Bronx, NY or Washington, D.C. ED and/or a Bronx, NY or Washington, D.C. hospital

2.3.2 Exclusion Criteria

The exclusion criteria for the universal offer of HIV testing are:

- Lacks the capacity to provide consent for HIV testing
- Acute or urgent medical condition that might be adversely affected by process of obtaining consent or performing HIV test

2.4 Study Sites for Expanded HIV Testing

2.4.1 Hospital/ED Study Sites in the Bronx, NY

Efforts to expand HIV testing will be undertaken at approximately seven Bronx hospitals with at least a Level I trauma center/ED and with daily hospital admissions. Overall, total ED visits at these hospitals in 2006 ranged from 49,635 to 134,969 per hospital.

2.4.2 Hospital/ED Study Sites in Washington, D.C.

Efforts to expand HIV testing will be undertaken at approximately seven Washington, D.C. hospitals. Routine HIV testing through the ED at George Washington University Hospital and throughout Howard University Hospital, begun in 2006, accounted for nearly 25% of all publicly supported tests in Washington, D.C. and 20% of reported positives in the HIV testing data in 2008.

2.5 Interventions for Enhanced HIV Testing Activities

The goal of the enhanced HIV testing activities to be implemented in the intervention communities is to increase the proportion of individuals from the community who have been tested within the prior year, with the intent of achieving earlier detection of HIV infection. Two populations are being targeted: those with ongoing risk through their own or partner risk behaviors and those with prevalent HIV-infection who are unaware of

their HIV infection. The former are targeted for regular HIV testing, at least annually, the latter are targeted for one-time "capture" testing.

At least three factors hamper the existing HIV testing promotion programs: limitations on the ability to influence hospital infrastructure; the inability to collect enhanced data from unfunded agencies; and limited resources to sustain social marketing campaigns. Within each institution, multiple stakeholders (including physicians, administrators and Department of Health staff) have identified necessary logistical changes that would be required to bring HIV screening to scale. Because staff have many other duties that often preclude their ability to focus on these key logistical or infrastructure change, the necessary coordination and follow-through between departments has not taken place. The ability to hire dedicated staff solely on overcoming key logistical barriers to routine HIV screening within each institution should significantly address this first limitation.

Programs such as "The Bronx Knows" have been able to collect aggregate data on HIV screening from all of its testing partners; however, because there has been no direct funding to each organization to support this data collection, the complexity and quantity of process data available to evaluate the initiatives are limited. The study will fund enhanced data collection directly at each site and allow creation of a more robust data set to evaluate various aspects of the testing promotion.

Although funds provided by the CDC in 2008 and 2009 have allowed for the development and dissemination of initial phases of consumer campaigns encouraging HIV testing, resource limitations hampered the sustainability of social messaging campaigns. Supplemental funding from the study will improve both sustainability of ongoing messaging and the creative development of additional components, including a module geared to medical providers. In both jurisdictions, the messaging to the general adolescent/adult population has been to get tested – either annually (Washington, D.C. message) or at least once (Bronx message). However, in both jurisdictions, some sub-populations, such as men who have sex with men, would be best served by more frequent repeated testing. Delivery of targeted enhanced testing messages alongside general testing messages will be supported by the TLC-Plus study.

In addition, the earliest models of routine HIV testing in medical settings were often stand-alone projects working in parallel with the existing medical services, necessary in order to prove feasibility in an environment where routine testing was felt to be burdensome or risky. These models have often relied extensively on the availability of rapid point-of-care HIV tests. However, as routine testing expands new models of integrated testing and lab-based large platform testing may be more appropriate to sustainably meet volume, workload, and cost-efficiency needs. The "early adopter paradigm" applies here – once a program has initial success in routine testing, support is needed for continued evolution or shift of that model. The TLC-Plus study will supplement CDC funding for expanded testing to support this evolution.

The study will build on current social mobilization efforts and communications plans in the intervention communities by adding two refined messages:

- All sexually active individuals age 13 and older should have had an HIV test within the prior year

- Sexually active MSM should seek HIV testing, at least annually, but ideally every six months

This study component will include social marketing to the general population in the two intervention communities, with outreach to the MSM community, healthcare providers, CBOs and social networking systems.

The study aims to achieve the goal of universal offer of HIV testing in EDs and during hospital admissions through the combination of the following approaches, tailored to the needs of each facility:

- Outreach to facility directors and key leaders at these institutions

- Establishment of HIV-testing goals for each facility

- Establishment of HIV testing as part of routine admission orders

- Provision of financial support for HIV-testing kits needed for increased testing

- Development and implementation of computer-delivered information on HIV testing

- Provision of computer terminals

2.6 Study Procedures for Expanded HIV Testing

2.6.1 Study Procedures for Social Mobilization

Both the Bronx, NY and Washington, D.C. have ongoing social mobilization activities as part of their HIV campaigns ("The Bronx Knows" and "DC Takes on HIV"). The HPTN 065 study will provide additional resources to craft and fine tune key messages aimed at increased testing and testing frequency as well as increase testing frequency in specific populations. In addition, the study will also support the development of messages that highlight importance of linkage to HIV care and HIV treatment. Focus groups will assist in developing these messages and in evaluating tools to determine their acceptability and effectiveness. The details of the social mobilization interventions will be determined at the beginning of the study; however, they may include, but are not limited to: flyers/brochures in clubs frequented by MSM; radio commercials; Internet ads/links to study information on sites commonly visited by MSM; and utilizing CBO staff to disseminate "word-of-mouth" messaging in the community.

2.6.1.1 Study Procedures for Social Mobilization in the Bronx

The study will supplement current efforts by the NYC DOHMH for the creative development and placement of social marketing materials throughout the Bronx, NY. Materials produced will both reinforce and supplement current social marketing messaging regarding the importance of routine HIV screening in healthcare settings and will further refine the messaging to the two target populations as indicated above.

The NYC DOHMH will develop social marketing materials using its standard procedures, including (but not limited to) contracting with local advertising agencies to create and produce materials, per NYC contracting practices.

All messaging will be subject to approval by the NYC materials review board, as is the practice for other CDC-funded social marketing materials developed by the NYC DOHMH. Sample materials produced also will be provided to the NYC DOHMH Institutional Review Board (IRB) for review.

Decisions regarding dissemination of social marketing materials will be made by the Bureau of HIV/AIDS Prevention and Control in the NYC DOHMH.

In general, placement of social marketing materials developed for MSM will focus on locations frequently visited by MSM including the Bronx Community Pride Center and local MSM-friendly Bronx clubs, such as Mi Gente. Where possible and appropriate, social marketing materials will be placed on the Internet (*e.g.* on social networking sites frequented by MSM in the Bronx).

2.6.1.2 Study Procedures for Social Mobilization in Washington, D.C.

Financial support will be provided to supplement the comprehensive social marketing program the Washington, D.C. DOH has already developed. This multi-phased program seeks to scale up routine HIV testing, promote treatment and promote behaviors to reduce risk of infection. The program is branded with the umbrella message "DC Takes On HIV" and promotes routine HIV testing in medical settings. Other aspects of the social marketing campaign include a consumer component entitled "Ask for the Test" and a provider component, "We Offer the Test." Using principles successfully implemented by the pharmaceutical industry, the campaign aims to drive consumers to ask for HIV testing when they visit their doctor. The message for providers is that patients will expect HIV testing as part of their standard healthcare. The consumer program features both traditional media (public transit, newspaper, radio and television advertising) and new media (Internet and text messaging). The DOH has developed an umbrella Web site www.DCTakesOnHIV.com with links to resources about HIV testing and services. The DOH has also established a text messaging service where residents can text "DCTEST" to 365247, receive a health fact, and then search by zip code for the nearest free HIV-testing location.

The provider component, "We Offer The Test," includes a toolkit (handbook, pocket card, poster, information cards for patients and appointment cards for HIV specialists) to make it easier for practitioners to implement routine HIV testing. The provider materials

include practical steps to make HIV testing routine, including sample scripts for use with patients, information on billing codes, information cards outlining what patients should know about both negative and positive results, and a “refusal” card that informs patients of the health risk from not getting an HIV test. DOH has formed a partnership with the Global Business Coalition and Pfizer to implement a pilot program under which Pfizer sales representatives will promote routine testing and provide the toolkit during their regular visits to medical practitioners. The pilot will start with 200 physicians and practices in Washington, D.C.

Washington, D.C. will also use social networking media (social Web sites such as Facebook and MySpace) to increase access to Washington, D.C. residents, and will use Twitter as a vehicle to communicate relevant information on HIV. Currently, the DOH has established these sites and a Twitter account for its free condom distribution program. DOH has entered into a contract with a public relations firm to advance the social marketing materials.

2.6.2 Study Procedures for Universal Offer of HIV Testing in EDs and Hospital Admissions in the Bronx

2.6.2.1 Emergency Departments

New York State has requirements for specific information that must be provided to patients before they sign separate, informed consent for HIV testing. HIV testing is already being offered in some EDs in the Bronx. Under this protocol, testing will be expanded at the EDs where it is currently offered and introduced in those that are not testing. Given the complexity and unique exigencies of each hospital's own logistics, this study will work within each facility to establish a procedure for universal offer of HIV testing. A universal offer development team (UODT), hired at the beginning of the study period for each intervention community, will conduct a series of meetings with staff at multiple levels of seniority within each hospital who have some stake in ED care. The UODT will work with these staff to identify and overcome current barriers to universal offer of HIV testing in their ED. Key staff may include the ED Director, the chief of nursing, laboratory director, chief ED residents and chief administrators. The UODT will also work with the ED leadership to set goals for testing and will provide quarterly results to each hospital to create and implement strategies for improvement.

Where necessary to improve logistics, laptop computers attached to rotating stands that can be easily moved from bay to bay will be provided by the study team to the ED site. Videos that meet New York State requirements for pre-test counseling will be loaded onto the laptops. Patients can view the videos and indicate whether they agree to test. Where possible, the video will be set up so that when a patient indicates interactively that he or she agrees to an HIV test, a tester (a nurse, physician or dedicated tester) will be notified and the test will be performed. While the patient waits for results, post-test, HIV-prevention messaging will also be viewable, if the patient chooses to watch it.

All EDs will be provided with free HIV test kits for individuals who are uninsured and cannot bill an insurance carrier (including Medicaid) for HIV screening. Free test kits

will be provided in proportion to the percentage of uninsured individuals seen in that ED who receive an offer of HIV testing at baseline.

The study will also provide EDs with a downloadable computer widget application on routine HIV testing that can be placed on the desktops of all ED computer terminals (either an updated version of the tool created by St. Vincent's Medical Center, with funding from the New York State AIDS Institute, or a similar tool). This widget will not only provide the full CDC Guidelines for routine HIV screening, but will include brief checklists that walk providers through the process of obtaining written consent (currently still required in NY), conducting an HIV test, providing results and handling a positive HIV diagnosis.

In working with the complex logistics in EDs, the study team will also operate within existing hospital admission processes as much as possible.

2.6.2.2 Hospital Admissions

Although some NYC hospitals have initiated programs to offer HIV testing for inpatients, none has yet implemented universal offer of testing at the time of hospital admission. At the beginning of the study period, the UODT will conduct meetings at hospitals with staff at multiple levels of seniority who have some stake in the inpatient admissions process and inpatient medical care. The UODT will work with these staff to identify and overcome current barriers to a universal offer of HIV testing in the hospital admissions process. Key staff will include the medical director, the chief of nursing, the laboratory director, internal medicine and surgery chief residents and chief administrators. The UODT will also work with the medical director and/or other designated hospital leadership to set goals for testing and will provide quarterly results to each hospital to create and implement strategies for improvement.

For any hospital that has set standing (basic, preset) admitting orders, the UODT will work with the Medical Director (or her/his designate) and the hospital's information technology department to have HIV testing added to these standing admitting orders, in accordance with hospital policies. The UODT will also work with the hospital's laboratory to overcome any barriers to placing HIV testing on hospital standing orders. All HIV testing completed as part of this protocol, whether in the ED or during hospital admission, will be conducted according to the laws of New York State. The UODT will work to ensure that all necessary paperwork is streamlined and available on all floors for the admitting teams and for admitting nurses.

All hospital admitting teams will ascertain if the patient has already been tested in the ED during his/her current visit, so that duplicate HIV testing is not performed. If possible, prior tests offered in the ED will be entered electronically, so that the admitting team will know whether the patient had been offered the test.

Admitting teams will be instructed to review the key pre-test counseling points that are required by New York State, obtain consent and, if possible, order the test with routine admitting phlebotomy. If ordering the test with routine phlebotomy is not possible, an HIV rapid test will be performed.

Alternatively (if desired) hospital admitting teams can be provided with laptop computers attached to rotating stands that can be easily moved from patient to patient. Videos that meet New York State requirements for pre-test counseling will be loaded onto the laptops. Patients can view the videos and indicate whether they agree to testing. Where possible, the video will be set up so that when patients indicate interactively that they agree to an HIV test, a tester (a nurse, physician or dedicated tester) will be notified and the test will be performed.

As with EDs, free HIV tests will be provided to the facilities for uninsured persons offered testing on admission. The percentage of tests offered will be derived in similar fashion to the percentage offered for ED testing. Where possible, the same downloadable widget provided to EDs will be provided to inpatient admitting teams for use on their computer terminals.

2.6.3 Study Procedures for Universal Offer of HIV Testing in EDs and Hospital Admissions in Washington, D.C.

2.6.3.1 Emergency Departments

Washington, D.C. EDs have been focal points for the implementation of routine HIV screening because many Washington, D.C. residents only access healthcare through the city's EDs. HIV testing in EDs can be integrated as a routine, opt-out procedure because Washington, D.C. does not require a separate written consent form for HIV testing. Patients are provided with written or verbal information about HIV testing, advised that a test is recommended, and (depending on available staff) a point-of-care rapid test is performed, unless the patient declines. In addition to the two institutions currently conducting routine testing in EDs, the Washington, D.C. DOH has begun to engage six additional EDs to undertake routine, opt-out HIV testing. HAHSTA recently completed review of a Request for Applications (RFA) to support expansion of routine HIV screening in EDs and hospital centers by providing support for a part-time coordinator at each hospital to promote implementation of routine testing. A coordinator at each facility will work with hospital leadership and department heads (CEOs and medical, lab and data staff) to develop plans for routine HIV screening in the ED and in other departments in the hospital system. In Washington, D.C., health insurers are required to reimburse for a voluntary HIV test performed during an insured's visit, regardless of the reason for that ED visit. Study funds will be used to support the cost of tests for patients who are uninsured. In addition, the study will support a part-time coordinator at each institution, working with management and ED and laboratory staff toward implementation of routine screening. The Washington, D.C. DOH will provide TA, including staff training, to all partnering hospitals.

Washington, D.C. has developed a provider toolkit for routine, opt-out HIV testing that includes an introductory brochure, pocket card, result cards and opt-out card. These tools define routine, opt-out HIV testing and provide checklists for the screening process, from test introduction to the provision of follow-up appointments for HIV-positive individuals. These materials will be available for wide distribution to the staff of partnering EDs participating in routine, opt-out HIV testing and to clinical providers throughout Washington, D.C.

The Washington, D.C. DOH will also provide test kits to partner EDs for use with uninsured patients. Although Washington, D.C. legislation mandates reimbursement for HIV testing performed in EDs, none of our partner hospitals has yet developed a protocol for billing for HIV tests. Free tests kits will be made available to hospitals while they develop systems to achieve sustainability through billing and reimbursement procedures. As those systems mature, the hospitals will assume responsibility for procuring test kits independently.

The study will provide financial support to participating EDs in order to increase staffing for HIV testing activities.

2.6.3.2 Hospital Admissions

Washington, D.C. hospitals are already at varying stages in the process of offering HIV testing to inpatients, but none conduct routine screening at admission. Seven of Washington, D.C.'s eight hospitals are performing rapid HIV testing in labor and delivery units for women without a documented HIV test result. Howard University Hospital currently makes rapid HIV testing available during regular business hours, upon physician or patient request. United Medical Center's long-range goal is to implement routine, opt-out HIV testing throughout the hospital.

Intensive TA will be provided to hospitals for admission testing, as with ED HIV testing. In an effort to promote the most cost-effective method of HIV testing, the DOH and study-supported coordinator at each institution will work with hospitals to add routine, opt-out HIV testing to standing orders for admission blood work for all hospital admissions. Washington, D.C.'s lack of a requirement for separate signed consent for HIV testing makes this a feasible option. Hospitals will also be encouraged to offer point-of-care tests for persons who are not tested at the time of admission.

Hospital admissions staff will have access to the routine, opt-out HIV testing reference materials originally developed for EDs. The Washington, D.C. DOH will also provide HIV test kits to hospitals for inpatient testing that must be performed at point-of care (when not done as part of admitting blood work) until they achieve sustainability through billing and reimbursements.

The study will provide financial support to participating hospitals in order to increase staffing for HIV testing activities.

2.7 Study Duration for Expanded HIV Testing

The expanded HIV testing component of this protocol will continue for the duration of the feasibility study, currently projected to be 36 months. It is expected—by virtue of emphasizing stakeholder buy-in, providing training and logistical support, and improving staff and systems capacity— that several key activities undertaken as part of this protocol, such as establishing HIV testing as part of routine admission orders, will be sustained after the study is completed.

2.8 Statistics and Data Analysis for Expanded HIV Testing

2.8.1 Endpoints

2.8.1.1 Endpoints for feasibility of enhancing HIV testing through a combination of focused and enhanced social mobilization efforts

- Number and results of HIV tests per month in publicly funded testing sites (local health department data)
- Number, transmission category and testing source of newly identified cases in HIV surveillance data
- Initial CD4 cell count of newly identified HIV cases in surveillance data
- Number of newly identified HIV cases concomitantly diagnosed with AIDS in surveillance data
- Proportion of persons in the community tested for HIV in the last year (local population-based behavioral surveys)

2.8.1.2 Endpoints for feasibility of routine offer of HIV testing at emergency departments and inpatient units

- Proportion and number of total ED visits and admissions to hospital where patients receive HIV testing
- Number of HIV tests in EDs where HIV infection is newly identified
- Number of HIV tests in hospital admissions where patients receive HIV testing
- Proportion of hospital admissions who have newly identified HIV infection
- Number of tested patients identified with previously diagnosed HIV who are not in care
- Cost of support for additional staff and HIV tests

2.8.2 HIV Testing Intervention Goals

More than 72,000 publicly supported HIV tests were conducted in Washington, D.C. in 2008, of which approximately 1000 were new positive diagnoses. Likewise, between April 2008 and March 2009, 161,619 HIV tests were performed as part of “The Bronx Knows” initiative, of which approximately 700 were new, positive diagnoses. This study will evaluate the increase in overall testing in Washington, D.C. and the Bronx. In addition to the volume of testing and number of new HIV diagnoses, the study will evaluate trends in CD4 cell count at first diagnosis.

Enhanced access to HIV testing in both ED and inpatient settings will occur throughout the entire 36-month study period. With regard to specific targets for the testing effort, the study will aim to offer HIV testing to 80% of eligible individuals during 80% of visits

with a 60% acceptance rate in the EDs. Likewise, the study will aim to offer HIV testing to 80% of eligible individuals with a 70% acceptance for inpatient admissions. The absolute numbers that these percentages represent will vary from hospital to hospital. The study will evaluate the ED and inpatient HIV data for increases in volume of testers, increases in number of HIV-positive tests and increased volume and percentage of new HIV infections detected.

2.8.3 HIV Testing Baseline Data

Table 1 contains the current levels of testing volume and newly identified cases from Health Departments and Surveillance data in each of the six cities (intervention and non-intervention) for 2007 and 2008.

Table 1. Testing Volume and Results in the Six Intervention and Non-Intervention Communities

Cities	2007				2008			
	No. of HIV Tests N	No. of Sites N	HIV+ Results % (n)	Newly Identified HIV+ % (n)	No. of HIV Tests N	No. of Sites N	HIV+ Results % (n)	Newly Identified HIV+ % (n)
Bronx[a]	127,947	21	1.3% (1,600)	727[b]	161,619[c]	21	0.9% (1,513)[c]	0.4% (691)[c]
Washington, D.C.	43,271	47	2.8% (1,192)	1.6% (702)	72,864	55	2.5 % (1,828)	0.8% (555)
Chicago	Not Available				77,616	42	1.0% (789)	0.5% (363)
Houston	38,612	16	1.05% (404)	1.70% (128)[d]	38,763	16	1.50% (580)	1.29% (109)[d]
Philadelphia	44,504	315	1.8% (780)	1.6% (710)	66,246	315	1.0% (678)	0.7% (489)
Miami	48,696	100	3.1% (1509)	1.8% (878)	59,510	117	2.7% (1611)	1.6% (912)

a) Data from *The Bronx Knows* Initiative
b) Data on linkage-to-care and newly diagnosed are only available for 19 agencies participating in *The Bronx Knows* for 2007.
c) *The Bronx Knows* agencies began reporting data in April of 2008; data reported here therefore covers the period of April 2008-March 2009 to provide one year's worth of data.
d) Data on newly identified HIV+ in Houston included only a subset of testing facilities. Numerators and denominators for these data reflect only that subset of tests/facilities.

2.8.4 Data Analysis

Data to be analyzed for this study component will be obtained from routine HIV surveillance sources and from utilization data collected by participating hospitals.

Endpoint measures of the potential feasibility of large-scale implementation of an expanded HIV testing strategy will be obtained by two analytic strategies: 1) comparison within the intervention communities before vs. after the intervention, and 2) comparison of change between communities with vs. without the intervention. For the within-community comparison, we will monitor the change from pre- to post-

intervention, with the primary interest in the change achieved at the end of the intervention period. For the between-community comparison, we will compare the change in communities with vs. without the intervention. Thus, we will test two indicators of change as a result of the intervention:

- **Within**: Comparison of outcomes of interest before vs. after intervention in an intervention community

- **Difference of differences**: Comparison of the change in a measure during the study period in communities with vs. without the intervention cities

Measurement of change or temporal trends in any of the outcome measures of the intervention implies that both of the above indicators of change will be computed.

2.9 Human Subjects/Ethical Considerations

The expanded HIV testing study component is a public health practice. Two geographic areas will participate in this study component: Washington, D.C. and the Bronx, NYC. Social mobilization and emergency department testing is already taking place in the two intervention communities (Bronx and Washington, D.C.) and is intended to be specific to the needs of those populations. While some observations from the overall TLC Plus project with regard to social mobilization and expanded testing may be applicable elsewhere in the United States, these activities were initiated originally for the specific benefit of the respective communities, and do not constitute research (CDC 1999).

The protocol will be submitted to appropriate IRBs (a central and/or local site IRBs) for ethical review prior to study initiation. Any subsequent modifications to the protocol will be submitted to appropriate IRBs, and, at a minimum, the protocol will be submitted annually for continuing review and approval by these same ethics boards. Because the expanded HIV testing component is public health practice applied in the two study communities, the study team will request that IRBs reviewing the protocol as a whole consider only the expanded testing component to be a non-research component, and therefore not subject to the requirements of 45CFR46. The other three components of this protocol constitute research and will be addressed in separate sections.

In Washington, D.C., written informed consent is not required for routine HIV testing; only oral consent, as part of standard voluntary opt-out HIV testing, is required. In contrast, New York State requires written informed consent for individuals undergoing HIV testing. For the expanded HIV testing component of this study, participants will undergo HIV testing according to the SOC and legal requirements of their jurisdiction.

To assess the impact of the study on HIV testing in the communities of Washington, D.C. and the Bronx, surveillance data routinely collected by the DOHs will be analyzed. No individual data, other than what exists in the surveillance databases, will be collected from study participants in the HIV-testing component of this study.

No study-specific laboratory testing will be conducted under this protocol. Therefore, no additional study-related test results will be reported to authorities. HIV-testing data for

tests performed in the EDs, hospitals and community test sites in Washington, D.C. and the Bronx, NY will be reported per local HIV reporting requirements.

The study team will neither collect nor report Adverse Events because there is no biomedical intervention. Again, due to the nature of this study component, the team will not collect or report Social Harms.

3.0 LINKAGE-TO-CARE

3.1 Study Objectives for Linkage-to-Care

The **primary feasibility objective** for using FIs to facilitate linkage of HIV-positive patients to HIV care sites will be measured by determining the following:

- Overall cost of the program, including staffing, program materials and incentives
- Extent of other available linkage-to-care activities (case managers, peer navigation, *etc*.)

The **primary effectiveness objective** for using FIs to facilitate linkage of patients from HIV testing to HIV care sites is the following:

- To increase the proportion of newly HIV-diagnosed and out-of-care patients linking to care within three months of identification

The protocol team will track all of the expenses listed above. Funds for these items will be held centrally and only disbursed when line-item bills are presented.

In order to monitor the implementation of the FI program, certain parameters will be gathered from a subset of participating sites at various time points to ensure program quality. These parameters will include: the proportion and number of patients eligible for incentives (the number of HIV–positives at a participating site), the proportion and number of patients receiving incentives, and the amount received compared with the total number who are eligible.

3.2 Design for Linkage-to-Care

The Linkage-to-Care component of the study is a two-arm, site-randomized, prospective, effectiveness clinical trial conducted within each intervention community. This study component compares the effectiveness of an FI intervention to link HIV-positive individuals from HIV test sites to HIV care sites with the usual SOC procedures. Each HIV test site will be randomized to either the intervention or SOC arm of the study. For this component of the study, study outcomes will be evaluated through routinely reported HIV surveillance data.

In order to identify the SOC for linkage, against which the incentives intervention will be assessed, an appropriate facility administrator will complete a brief form that will collect key attributes of HIV testing sites, including types of navigation and support services already available to patients to facilitate linkage-to-care.

For the purposes of this protocol, we consider an HIV-positive individual linked to care when that person has a VL or CD4 cell count assessment at an HIV care site within 3 months of confirmatory WB testing.

3.3 Study Population for Linkage-to-Care

The Linkage-to-Care component of the study will include all individuals who are permitted to consent for HIV care according to New York State or Washington, D.C. law newly found to be HIV-positive at HIV test sites participating in the study. This study component will also include individuals who have been previously diagnosed with HIV but have been out of care for at least a year and are reconfirmed for HIV infection by standard laboratory tests.

3.4 Study Sites for Linkage-to-Care

Twenty HIV test sites will be selected from Washington, D.C. and 20 HIV test sites will be selected from the Bronx to participate in the Linkage-to-Care component of this study.

HIV test sites will be selected based on two criteria: 1) site agreement to participate in this component of the study, and 2) sites with the highest volume of HIV-positive individuals identified in the previous year.

In each community, these 20 sites will be randomized such that 10 will use the FI intervention, described in Section 3.5, to link HIV-patients to care and 10 will use the SOC only.

The site randomization will be balanced by the following two baseline characteristics:

- The number of HIV-positive individuals identified in the previous year
- The rate of linkage-to-care within three months of HIV diagnosis over the course of the calendar year prior to study initiation

In addition, 20 care sites will be selected in Washington, D.C. and 20 care sites will be selected in the Bronx, NY. These care sites will redeem the coupons provided to patients by the test sites selected for the FI arm.

3.5 Intervention for Linkage-to-Care

At HIV test sites assigned to FIs, individuals permitted to consent for HIV care according to New York State or Washington, D.C. law who test HIV-positive will be provided with a coupon that is redeemable at participating HIV care sites. These coupons will be designed so they will not breach patient confidentiality (for example, there will be no patient names, no indication of HIV status, and no clinic names on these coupons). Patients presenting coupons to participating HIV care sites will be given an FI upon completion of a blood draw/lab visit ($25), which is usually the first visit, and another FI ($100) upon an interaction with their healthcare provider, which is usually at a second visit. Alternatively, for those patients who complete a comprehensive visit at

participating care sites, a visit that includes both the lab and provider components for Linkage-to-Care, a $125 FI gift card will be provided.

The coupons distributed at the HIV test sites must be redeemed at the HIV care sites within three months of the date that a participant receives them.

The proportion of persons successfully linked to care within three months of their HIV-positive test will be compared, through the use of routinely collected HIV surveillance data, between sites implementing the incentives intervention and those with SOC procedures for linkage-to-care.

3.6 Study Procedures for Linkage-to-Care

The specific procedures for HIV test sites to obtain and distribute the FI coupons for linkage-to-care, as well as the procedures for the redemption of these coupons at HIV care sites, are outlined in the HPTN 065 Study-Specific Procedures Manual (SSP).

3.6.1 Procedures at Test Sites

Participating test sites will link all HIV-positive patients to an HIV care site. Each test site will be provided with a listing of participating HIV care sites (with contact information) to give to HIV-positive patients. In addition, test sites will give HIV-positive patients coupons redeemable for FIs at HIV care sites.

Prior to study implementation, all HIV test site staff will be trained on the procedures for the FI intervention. Providers will also be trained on HIV prevention counseling, and the importance of linking HIV-positive patients to care.

Study test sites will maintain the ability to refer clients with urgent needs (e.g., mental health) for necessary services. However, test sites will not routinely link patients to support services. The DOHs in both intervention communities actively encourage a *single* referral of HIV-positive patients from test sites to care sites. Multiple referrals can be overwhelming to newly HIV-diagnosed patients and may give the wrong impression that an individual must be drug-free in order to engage in HIV care, and may paradoxically delay entry into care. Additionally, testing sites usually do not have ongoing relationships with persons in need of support services, and are thus not well suited for evaluating sustained linkages to these resources or adequacy of services to meet patient needs.

3.6.2 Procedures at Care Sites

Participating HIV care sites will serve as the comprehensive ‘medical home’ for coordination of all HIV-positive patient care needs, including substance use, mental health, and other support services.

As newly diagnosed HIV-positive patients present for care, care sites will evaluate CD4 cell count and VL. Care sites will then create an appropriate medical plan for each patient, consistent with current HIV care and treatment guidelines. Simultaneously, care sites will also assess patients and link them as appropriate to support services. Finally, valid coupons distributed by HIV test sites will be redeemed with FI gift cards.

Prior to study implementation, all HIV care site staff will be trained on the procedures for the FI intervention, ART initiation according to current guidelines, and the importance of linking HIV-positive patients to appropriate support services. In addition, as part of study orientation and training for HIV care providers, existing support service linkages will be reviewed.

To collect clinic-level information on services available to patients in the two jurisdictions, HIV care sites will be surveyed at baseline and annually thereafter to ascertain support services available on- and off-site (e.g., social services, substance use treatment, support groups, mental health resources, etc). The study team will develop and maintain a comprehensive list of support services in the two intervention communities. In addition, the study will regularly update lists for care sites of contact persons and referral processes at support service agencies.

Because the study will use surveillance data to measure outcomes and will not collect data on individual patients, the study will not be able to document or track linkages to ancillary services in the Linkage-to-care and Viral Suppression study components. Information on referrals made and completed by patients is not part of the surveillance data that will be analyzed for study outcomes. Likewise, offering FIs for completion of linkages to drug treatment or mental health services is not possible, as it would also require following individual patients with explicit data collection. However, for the subset of patients completing Patient Survey, we will collect data on the frequency of use, ease of access, and patient experiences with support services (including mental health, drug abuse, case management, etc).

3.6.3 Monitoring HIV Test Sites

It is possible that members of the intervention communities will learn of the FI to strengthen linkage-to-care and seek out HIV test sites offering these incentives. In all likelihood, the majority of individuals who seek testing will not know their HIV status yet and, thus, will be unaware that incentives are being offered for linkage-to-care. In addition, many of the HIV test sites in the study (~seven out of the 20 participating HIV test sites in each city) will be hospitals in which HIV testing will take place in emergency departments and inpatient units. Individuals seen in these settings are usually not there for the purpose of obtaining an HIV test. Thus, it is unlikely that these individuals will seek medical services based on the availability of FIs for linkage to HIV care.

However, it is possible that some individuals, who are aware of their HIV infection, may "recycle" through the HIV test sites due to the availability of FIs. There are some natural barriers to this phenomenon, as it is likely that the staff at these HIV test sites will recognize individuals who are testing with undue frequency. In these cases, staff may withhold the FI coupons from those who have already received them for a prior HIV-positive test. Another natural barrier is that individuals are known at HIV care sites, so individuals will be unable to re-link repeatedly to the same HIV care site. In addition, the FI intervention is designed such that multiple activities (lab-work and provider interaction, both of which take time and effort) are required for the full redemption, which may discourage some from repeated linkage.

It should also be noted that based on the HIV prevalence in the intervention cities, relatively few people test positive out of all of those who test. Thus, the number of

people who could “recycle” through the HIV test sites is relatively small compared to the total testing done in these venues.

Despite all of the barriers to HIV-test-site migration and linkage recycling, FI may encourage these phenomena; thus, the study team will use surveillance data to monitor these potential situations. Specifically, the team will monitor the number of duplicate linkage-to-care events in the name/ID-based surveillance data system over time. So, for example, in the case where a person re-tests at five unique HIV test sites (all offering linkage-to-care incentives) and links to five unique participating HIV care sites over the course of six months, HIV surveillance data will capture this event.

In addition, the team will monitor whether the number of positive HIV tests becomes severely disproportionately distributed among the participating HIV test sites (a sign that HIV testing is much reduced or increased at some sites). This information will be examined in an on-going fashion, and if site migration or linkage recycling becomes highly extensive so that it becomes a burden on HIV test or care sites, or threatens the validity of the evaluation, this component of the study will be re-evaluated. Such re-evaluation may include discussions with the community advisory group to design other mechanisms to minimize such migration, or, if needed, a redesign of this component of the study may be embarked on by the study team.

3.7 Study Duration for Linkage-to-Care

The duration of the FI intervention for the Linkage-to-Care component of the study is depicted in Figure 2. Coupons will be available at the HIV test sites randomized to the FI arm for 21 months. These coupons can be redeemed after the initial visit with an HIV provider at HIV care sites for a total of 24 months (for the entire time coupons are distributed at certain HIV test sites plus an additional three months).

Figure 2: Duration of FI Intervention for Linkage-to-Care

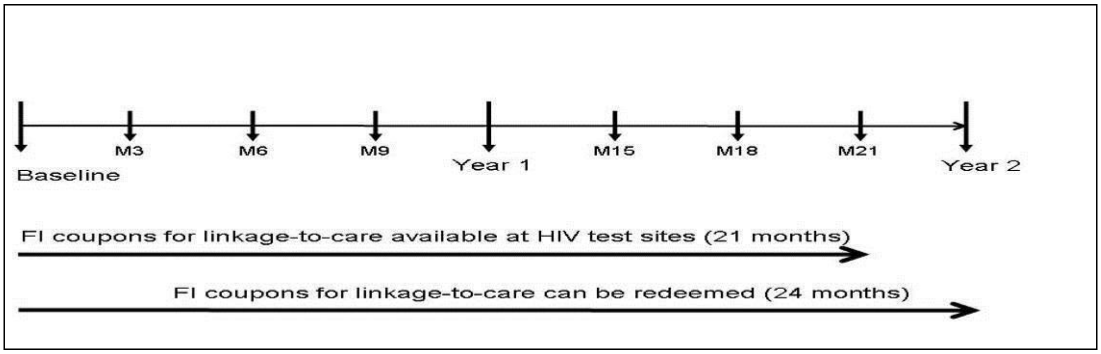

3.8 Statistics and Data Analysis for Linkage-to-Care

3.8.1 Endpoints

3.8.1.1 Endpoints for the feasibility of using financial incentives in the HIV-positive population for Linkage-to-Care

The feasibility endpoints for the Linkage-to-Care component of the study are the following:

- Number of individuals eligible for incentives (see section 3.3) and number of individuals receiving incentives (upon linkage to HIV care) at participating sites
- Cost of the program including staffing, infrastructure and incentives

3.8.1.2 Endpoints for the Effectiveness of Linkage-to-Care

In this site-randomized design, Linkage-to-Care effectiveness endpoints will be evaluated for each site and intervention effectiveness assessed by comparing site outcomes for intervention vs. SOC sites.

The effectiveness endpoints for the Linkage-to-Care component of the study will be ascertained based on HIV surveillance data and will include the following:

- Proportion of HIV-positive individuals at each testing site, who either have a newly detected HIV infection (based on new surveillance case with confirmed WB) or who were previously diagnosed but were out of care (based on no VL or CD4 ascertainment in the year prior to a repeat WB test in an existing surveillance case), and who presently are linked to care as evidenced by having a CD4 cell count and/or VL measurement at a separate visit within three months of WB confirmation

- Mean time interval at each testing site from HIV diagnosis (WB confirmation) to first CD4 cell count or VL for those with newly detected HIV infection and those who were previously diagnosed but were out of care

- Proportion of HIV-positive individuals at a testing site (overall, and separately for those with newly detected HIV infection and those previously diagnosed who were out of care) with at least two CD4 cell count and VL measurements in the prior year

3.8.2 Sample Size and Power

The primary endpoint of this study component is proportion of cases (newly diagnosed and previously diagnosed but not in care) with a CD4 cell count and/or VL measurement within three months of the diagnostic WB.

Randomization is by testing site, with balance achieved for baseline volume of testing and baseline linkage-to-care rates. Accrual of participants into the Linkage-to-Care

component of the study will occur over the first 24 months of the study, when eligible persons receive the FI coupon at an HIV test site randomized to the FI arm of the study. There will be no follow-up for this component of the study, other than the two visits in care (or a combined lab/provider intake visit).

In 2007, in the 20 testing sites with the highest volume in the Bronx, NY, 611 newly diagnosed HIV-infected people were identified. Of those, 77% were liked to care within three months, with linkage at different testing sites ranging from 30% to 100%. The mean number of newly diagnosed cases per provider in 2008 was 31, and ranged from five to 93. The intra-class correlation (ICC) of proportion linked to care in the Bronx was 0.27.

In 2008, in the 20 testing sites in Washington, D.C, the jurisdiction with the highest volume of people testing HIV-positive, 783 newly infected cases were identified. Of these, 67% were linked to care, ranging across different testing sites from 5% to 100%. The mean number of newly diagnosed cases per provider in 2008 was 40, and ranged from eight to 143. The ICC of proportion linked was 0.31.

These estimates do not include the population of those re-linking to care. It is anticipated that linkage success in SOC for this population will be lower because of the history of loss to care. We could reasonably expect that: 1) the number of people eligible for linkage-to-care will be higher because of longer study duration and addition of re-linked to care cases; 2) the proportion linked to care will be lower because of the re-linked to care cases; and, therefore, 3) the ICC will be smaller. It is assumed that the number of cases to be linked to care during the 24-month study duration will be similar to these past case loads on an annual basis. We will randomize 20 testing sites in each intervention city, and we expect to identify between 1200 and 2400 individuals for linkage-to-care, a mean of 60-120 people per testing site.

Table 2 below gives the mean number of linkage cases needed per testing site to detect an increase in linkage-to-care given 20 sites per arm (*i.e.* 40 in all). With a mean ranging from 80 to 100 linkages per site during the 24 months of the study, we would have 80% power, even with a high ICC, to detect a 13% change in proportion linked to care. If higher numbers are eligible for intervention due to linkage-to-care (*e.g.* 140), we would attain 90% power to detect this magnitude of difference. Note that the ICC coefficient estimated from the linkage-to-care of newly detected cases is high in large part because of sites with a small number of newly detected cases and 100% success in linking to care. It is probable that the ICC observed in the trial will be lower.

This sample size calculation for clustered designs uses the approach described by Thomson, Hayes and Cousens (Hayes and Bennett 1999; Thomson, Hayes et al. 2009).

Table 2. Mean number at each site required to achieve sufficient power to detect differences in proportion of newly detected or reconnected to care within three months of testing. Number of clusters fixed at 20 per arm

Mean percentage linked to care at a site		Standard deviation of percent linked to care		Intra-class correlation	Mean number of potential cases for linkage-to-care per testing site	
**Standard of care**	**Intervention**	**Standard of care**	**Intervention**		**80% power**	**90% power**
67%	80%	17%	12%	0.127	37	49
67%	80%	20%	15%	0.183	54	72
67%	80%	26%	19%	0.309	102	137
67%	75%	17%	14%	0.127	105	140
67%	75%	20%	17%	0.183	152	203
67%	75%	26%	22%	0.309	287	384

3.8.3 Randomization Scheme

In the Linkage-to-Care study component, HIV testing sites will be randomized to use FIs or the SOC.

HIV test sites will be assigned to one of the following two arms:

- An arm offering FIs: Test sites assigned to this arm will provide coupons to all individuals testing HIV-positive, who are not already linked to HIV care. The coupons can be redeemed for the FI at a participating HIV care site.

- An arm continuing with SOC: Each person who tests HIV-positive, and is not currently in care, will be directed to HIV care sites using the site's SOC procedures.

The site randomization will be balanced by the following two baseline characteristics:

- The number of HIV-positive individuals identified in the previous year

- The rate of linkage-to-care within three months of HIV diagnosis over the course of the calendar year prior to study initiation

3.8.4 Data Analysis

Data to be analyzed for this study component will be obtained from routine HIV surveillance data as well as from the participating sites. Participating sites will maintain tracking logs, both for coupons provided to HIV-positive individuals at the test sites and for FI disbursement at the care sites. Information from the tracking logs, with all participant-identifying information removed, will be provided to the study team.

The analysis approach for testing the effectiveness of an FI intervention to link HIV-positive individuals from HIV test sites to HIV care sites compared to the SOC is described below. Analysis of secondary endpoints will be detailed in the statistical analysis plan.

3.8.4.1 Primary Effectiveness Analysis

The population for assessment of Linkage-to-Care is the following:

1. All individuals with newly detected HIV infections reported to the HIV surveillance system for the DOH jurisdiction of Washington, D.C. and the Bronx, NY who were tested at any of the 40 testing sites in the study

2. All participants eligible for re-linkage to care, defined as a WB recorded in the enhanced HIV/AIDS Reporting System (eHARs), at any of the 40 testing sites in the study after the implementation of FIs, who had no VL or CD4 assessment by a provider in the previous 12 months

The endpoint is the percentage of participants with a VL or CD4 cell count assessment within three months of the WB confirmation (separate from clinical assessment done at the time of the confirmation visit). Participants in the FI arm are attributed to the linkage-to-care strategy in place at the site where the first HIV-positive result was detected.

The estimation of intervention effect will test for a change in odds ratio of linkage-to-care between the SOC and FI test sites, assuming a correlation within participants tested at the same site, using Generalized Estimating Equations (GEE) methods.

3.8.4.2 Feasibility Endpoints

Analysis of feasibility endpoints will use descriptive statistics to characterize the variability of uptake of FIs across types of testing venues.

3.9 Human Subjects/Ethical Considerations

The Linkage-to-Care component of the study involves a randomized intervention using FIs to link HIV-positive individuals to HIV care after diagnosis. This component is public health research. In this component, HIV test sites will be randomized, not individuals, and no de novo individual-level data will be collected. Instead, (de-identified) HIV surveillance data routinely collected by the Departments of Health (DOH) in the two communities will be analyzed. Because this research involves minimal risk and would be impracticable with informed consent, a waiver of patient informed consent will be requested under 45 CFR 46.116 (c) or (d).

The protocol will be submitted to appropriate IRBs (a central and/or local site IRBs) for ethical review prior to study initiation. Any subsequent modifications to the protocol will be submitted to appropriate IRBs, and, at a minimum, the protocol will be submitted annually for continuing review and approval by these same ethics boards.

This component of the study involves an intervention using FIs to link HIV-positive individuals to HIV care after diagnosis. These incentives are described in detail in the intervention section for this study component. No additional incentives (for example for transportation) will be given to individuals who participate in this component of the study.

No individual data, other than what exists in the surveillance databases, will be collected from study participants in the Linkage-to-Care component of this study. To assess the impact of the study on Linkage-to-Care in the communities of Washington, D.C. and the Bronx, HIV surveillance data routinely collected by the DOH will be analyzed. Data from the DOHs will be provided to the study team in a de-identified fashion, as per usual HIV surveillance procedures.

No study-specific laboratory testing will be conducted under this protocol. Therefore, no additional study-related test results will need to be reported to authorities. HIV testing and care data collected during routine clinical care will be reported per local HIV and AIDS reporting requirements.

3.10 Safety Monitoring and Adverse Event Reporting

The study team will not collect or report Adverse Events because there is no biomedical intervention. However, the team will collect and report all social harms that are brought to the attention of study staff members, using a study-specific incident report form. This form will be anonymous and will query common social harms such as altered personal relationships, forced change in housing, and physical violence. The form will also include space for a written narrative to document additional details of any social harm experienced. All research staff will be trained to properly complete the form. As a part of study training, research staff will also be trained on the provision of referrals to counseling and social service support. Reports of social harms will be reviewed quarterly or more often, if indicated, and reported to the medical officer together with any actions that are taken. Social harms will be summarized and reported to appropriate IRB(s) on an annual basis.

4.0 VIRAL SUPPRESSION

4.1 Study Objectives for Viral Suppression

The **primary feasibility objectives** for using FIs to incentivize HIV-positive patients to achieve and maintain viral suppression (HIV RNA < 400 copies/mL) will be measured by determining the following:

- Proportion and number of patients in care, on ART (eligible for incentives), and receiving incentives upon achieving VL suppression
- Number of incentives disbursed compared with the total number of incentives available to eligible patients over the study duration [measurement of duration of viral suppression]
- Overall cost of program including staffing, infrastructure and incentives
- Number of patients previously adherent to ART who change from non-intervention to intervention care sites

In order to monitor the implementation of the FI program, certain parameters will be gathered from a subset of participating sites at various time points to ensure program quality. These parameters will include: the proportion and number of patients in care, on ART (eligible for incentives) and receiving incentives, as well as the incentive amount received compared with the total number on ART who would be eligible.

The **primary effectiveness objective** is to compare an FI intervention with the SOC for achieving and maintaining suppressed VL in HIV-positive patients in care, managed under the prevailing guidelines for the initiation of ART. For sites in each intervention arm, this objective will be measured by the following:

- Comparing the mean proportion of patients in care at a site who have suppressed VL (HIV RNA <400 copies/mL) during the fixed 12-month calendar evaluation period beginning 12 months after initiation of the FIs at that site
- Comparing the trend in mean proportion of patients in care at a site who have suppressed VL over time since initiation of FIs

- Comparing the mean proportion of patients who have suppressed VL (HIV RNA <400 copies/mL) after cessation of incentives

4.2 Design for the Viral Suppression

The viral suppression component of the study is a two-arm, site-randomized, prospective, effectiveness clinical trial. It will be conducted within each intervention community to assess the effectiveness of a FI intervention for achieving and maintaining suppressed VL (HIV RNA < 400 copies/mL) in HIV-positive individuals (who have initiated ART under prevailing guidelines) compared to SOC for supporting patients in achieving adherence and virologic suppression. Each HIV care site will be randomized to either the intervention or SOC arm of the study. For this component of the trial, study outcomes will be evaluated through routinely reported HIV surveillance data.

In order to identify the SOC for ART adherence/viral suppression support, against which the incentives intervention will be assessed, we will develop a brief form, to be completed by an appropriate facility administrator, which will collect key attributes of HIV care sites, including types of ART adherence support, case management services, and other support services already available to patients.

4.3 Study Population for Viral Suppression

The study population for the viral suppression component of the study will include all individuals permitted to consent for HIV care according to New York State or Washington, D.C. law, who have initiated care at participating HIV care sites.

4.4 Study Sites for the Viral Suppression

Twenty HIV care sites will be selected from Washington, D.C. and 20 HIV care sites will be selected from the Bronx to participate in this component of the study. Sites will be selected based on two criteria: 1) site agreement to participate in this component of the study, and 2) sites with the highest number of HIV-positive patients in care in the previous year.

HIV care sites will be randomized 1:1 to the FI vs. SOC, stratified by city and balanced by size of patient population in a clinic and baseline levels of proportion of VL showing suppression. In each city, these 20 sites will be randomized such that 10 will use the FI intervention, described in Section 4.5, to promote viral suppression in HIV-patients on ART and 10 will continue the existing SOC.

The site randomization will be balanced by the following two baseline characteristics:

- The size of the site's HIV-positive patient case load

- The proportion of HIV-positive patients with VL suppression at each site

4.5 Intervention for the Viral Suppression

HIV-positive individuals at HIV care sites assigned to the FI intervention will receive FIs (gift cards worth $70) to reinforce adherence to ART as measured by confirmation of each suppressed VL measurement (<400 copies/mL). The gift cards will be distributed at the HIV Care sites during each routine, quarterly clinic visit that a study participant demonstrates a suppressed VL.

In the event participants report full adherence to their providers but have not succeeded in achieving VL suppression, providers will be expected to obtain resistance testing, as recommended by current treatment guidelines, and to alter their patients' regimens accordingly. Once patients with resistant HIV are on appropriate regimens, they will again be eligible for FIs for viral suppression. The specific procedures for HIV care sites to distribute the FIs for viral suppression will be outlined in the HPTN 065 SSP.

This intervention will be compared to SOC support for ART adherence through the use of routinely collected HIV surveillance data.

4.6 Study Procedures for the Viral Suppression

At the initiation of the study, the study team, along with experts in treatment and management of HIV disease, will provide training to providers in the intervention communities on current HIV-treatment guidelines. Training will encourage providers to maximize initiation of ART per current guidelines and will emphasize the following: eligibility criteria for ART as defined in the guidelines; assessment of patient readiness to start ART (if eligible); prompt initiation of ART (if eligible); use of recommended regimens; the provision of supportive services to ensure adherence; regular clinical and laboratory monitoring; the importance of assessment for evidence of treatment failure; reinforcement of adherence; obtaining HIV resistance testing (if appropriate); and modification of failing regimen with new effective regimen. All HIV care site staff will also be trained on the procedures for the FI intervention prior to implementation. Along with this training, the importance of linkage to supportive services will be emphasized, including income assistance, housing support, substance use management and mental health services. Each HIV care site, regardless of the study arm it is randomized to, will be provided with a listing of available resources in the community with contact names and numbers. Providers will also be trained on HIV prevention counseling.

A baseline survey will be conducted to determine the availability of supportive services at all participating HIV care sites (*e.g.* social services, adherence support, substance use treatment, support groups, mental health resources, *etc.*) and to determine whether these services are available on site or by referral. In addition, an effort will be made to monitor the number of new referrals and waiting times for appointments to assess program capacity.

The specific procedures for HIV care sites to distribute the FIs for viral suppression will be outlined in the HPTN 065 SSP.

4.6.1 Monitoring HIV Care Sites

It is possible that members of the intervention communities, particularly those who are HIV-positive, will learn of the FI intervention to promote viral suppression and seek out HIV care sites offering these incentives. In an effort to minimize significant migration of patients from HIV care sites without FIs to ones that offer them, the following rules have been put into place:

- A patient must have at least one VL measurement at the HIV care site between three and nine months prior to the first suppressed VL for which he or she may receive an incentive. Both of these VL measurements must be done at the same HIV care site.

- All subsequent VL measurements for which a patient may receive FIs must be performed at the same HIV care site.

If a patient changes HIV care sites during the course of the study, he or she will be required to "establish care" at the new HIV care site prior to receiving any FI for a suppressed VL as indicated above. Specifically, to receive an FI again, the individual must have a VL measurement at the new HIV care site and return in three to nine months for a repeat VL measurement. The results of the second test will determine whether the person receives the FI or not.

Despite this barrier to HIV-care-site migration, the FI intervention may encourage this phenomenon; thus, the study team will use surveillance data to monitor the case load at each site at frequent intervals. By monitoring HIV care site case load over time, the team will be able to determine if significant migration is taking place. This will motivate discussions with the community advisory group to design other mechanisms to minimize such migration or if needed a redesign of this component of the study may be embarked on by the study team.

4.7 Study Duration for the Viral Suppression

The duration for the FI intervention for the viral suppression component of the study is depicted in Figure 3. Participants will be eligible to receive FIs for suppressed (< 400 copies/mL) VL measurements once every three months throughout the entire 24-month study period. Participants who miss a quarterly visit(s) remain eligible for incentives as long as they present back to care with a suppressed VL measurement.

Patients with HIV diagnoses returning to care after a hiatus (of at least a year) and individuals who switch HIV care sites during the study are all eligible for the FIs. However they must have at least one VL measurement at three months (or more) at the same HIV care site as the qualifying VL measurement for which they can receive an FI.

Accrual of participants into the Viral Suppression component of the study will occur over the entire 24 months of the study. Some participants may receive up to nine FI payments if they are seen at the same HIV care site throughout the entire study, had been receiving care at that the same site before the study began, and maintain viral suppression throughout the 24 months.

Figure 3: Duration of FI Intervention for Viral Suppression

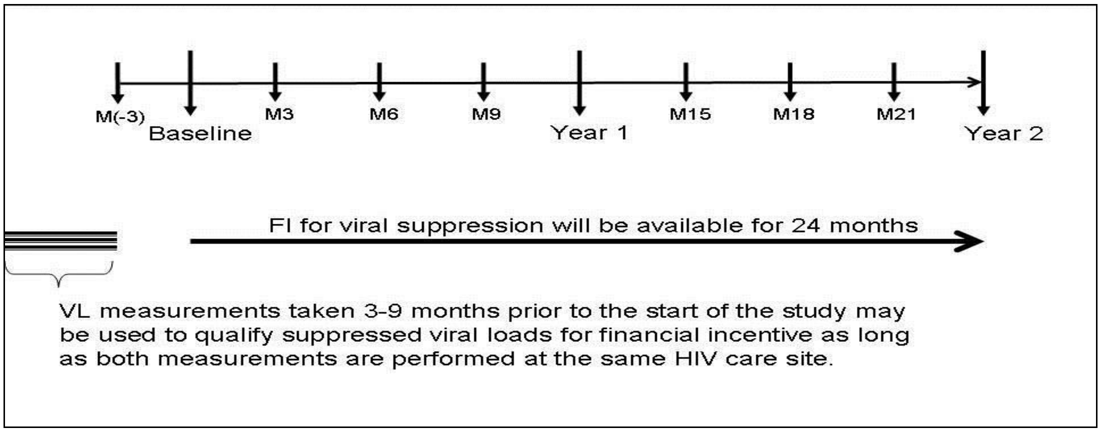

4.8 Statistics and Data Analysis for the Viral Suppression

4.8.1 Endpoints

4.8.1.1 Endpoints for the feasibility of using financial incentives in the HIV-positive population for viral suppression

The feasibility endpoints for the viral suppression component of the study will include the following:

- Number of individuals eligible for incentives and receiving incentives at a select subset of sites for select time points
- Cost of program including staffing, infrastructure and incentives

4.8.1.2 Endpoints for the Effectiveness of Viral Suppression

The effectiveness endpoints for the viral suppression component of the study will include comparing between sites in the intervention and SOC arms:

- Probability of an HIV-positive patient in care at a site having a suppressed VL (<400 copies/mL) in the 12-month calendar assessment period beginning 12 months after initiation of the assessment period
- Number of identified HIV-positive patients in care who have sustained viral suppression (see below)

The primary endpoint of the intervention is evaluated using all VL ordered at each site in the 12-month period beginning 12 months after initiation of FIs. An individual will be defined as in care at a site if two VL or CD4 assessments were ordered at that site in the year prior to initiation of this study, or if care (defined by two VL/CD4 assessments) is initiated at the site during the study. PLWHA will be assigned to sites (and thus arms) based on the site where they are in care.

To avoid ascertainment bias that would arise from missing VL assessments in the in-care cohort, we will impute whether VL is suppressed in each quarter of the 12-month assessment period a patient is in care (this is based on an SOC where VL assessments are ordered approximately every three months). For example, a patient with a VL assessment in each quarter will have an endpoint assessment for each quarter. A patient who has a suppressed VL assessment in the first, third and fifth quarters, but no assessment in the second and fourth, will be assumed to be suppressed in the second and fourth. However a patient with a suppressed VL in the first quarter and no subsequent assessment throughout the study will be assumed not suppressed in all subsequent quarters. The algorithm for imputing VL suppression (incorporating the sequence of VL assessments and including management of the reporting lag in surveillance data), inclusion of assessments in the quarters preceding and following the assessment period, handling of cases in treatment at more that one site, and censoring because of moving outside the surveillance area, death, *etc*. will be detailed in the statistical analysis plan. Contamination between arms will be tracked by detection of patients with VL assessments at multiple providers in the year.

It should be noted that the existing surveillance procedures include extensive efforts to link cases across jurisdictions. Individuals moving outside the jurisdiction will be censored at last measure prior to relocation.

4.8.2 Sample Size and Power

The top 20 provider sites in Washington, D.C. and the Bronx account for approximately 5,000 HIV patients in Washington, D.C. and 16,000 in the Bronx (exclusive of incarcerated patients in HIV care). It is assumed that the number of cases in care during the 24-month study duration will be similar to these past case loads on an annual basis. In the Bronx, VL was suppressed in 57% of 8,316 patients with VL assessed in 2008. The mean number of patients per clinic with a VL assessment in 2008 in the top 20 care sites was 400, ranging from 50 to 3,300. The ICC of proportion virally suppressed estimated from the Bronx 2008 data was 0.07. In Washington, D.C., 67% of 2,926 patients with VL assessments in 2008 achieved viral suppression at their most recent assessment. The mean number of patients per clinic with VL assessments was 245, ranging from 35 to 1,400 in the top 20 clinics. The ICC of proportion virally suppressed estimated from the Washington, D.C. 2008 data was 0.104.

Table 3 shows the mean number of patients with VL assessments needed per clinic, assuming 20 clinics in each arm (40 clinics in total), to detect a change in proportion of virally suppressed for a range of effect, power and ICC. If the clinics had a mean of 219 patients with VL assessments in the 12 months of evaluation, we would have 90% power

to detect an increase in viral suppression from 60% to 66% between the SOC and intervention clinics.

Sample size calculation for clustered designs use the approach described by Thomson, Hayes and Cousens (Thomson, Hayes et al. 2009).

Table 3. Mean number of patients in care at a site required to achieve sufficient power to detect differences in proportion with suppressed VL. Number of clusters fixed at 20 per arm.

Percent in care virally suppressed		Standard Deviance of percent virally suppressed		Intra-class correlation	Mean number of patients in care	
Standard of care	Intervention	Standard of care	Intervention		80% power	90% power
60%	70%	15%	13%	0.094	57	76
60%	70%	16%	14%	0.105	62	83
60%	70%	18%	16%	0.135	78	104
60%	66%	15%	14%	0.094	163	217
60%	66%	16%	15%	0.105	179	219
60%	66%	18%	13%	0.135	199	299

4.8.3 Randomization Scheme

In the viral suppression study component, facilities that provide HIV care services (HIV care sites) will be randomized to use FIs or the SOC.

HIV care sites will be assigned to one of the following two arms:

- Sites offering FIs for VL suppression: FIs offered to all those who are on ART, upon the confirmation of each suppressed VL measurement (<400 copies/mL)

- Sites continuing with SOC to encourage VL suppression: Each person on ART will be offered support via the site's SOC procedures to attend HIV care site visits and remain adherent to their ART regimen in order to achieve and maintain VL suppression

The site randomization will be balanced by the following two baseline characteristics:

- The size of the site's HIV-positive patient case load

- The proportion of HIV-positive patients with VL suppression at each site

4.8.4 Data Analysis

Data to be analyzed for this study component will be obtained from routine HIV surveillance data as well as from the participating sites. Participating sites will maintain tracking logs for FIs provided to participants maintaining suppressed VLs. Information from the tracking logs, with all participant-identifying information removed, will be provided to the study team.

The data for the effectiveness of an FI intervention for high ART adherence to achieve viral suppression compared to the SOC will be analyzed as described below. Analysis of secondary endpoints will be detailed in the statistical analysis plan.

4.8.4.1 Primary Effectiveness Analysis

The assessment cohort includes all HIV cases identified from HIV surveillance in the intervention city DOH jurisdiction that have a VL assessment in the 24 months of trial duration. The assessment period for each site begins 12 months after the onset of FIs in each city. An intention-to-treat approach will be used to assign patients to the care site assignment existing at the trial onset. For the 12-month assessment period (*i.e.* from 12 through 24 months), all VLs in the surveillance data assessed by the 40 clinics in the study are used to assess differences in percent achieving viral suppression by study arm. Details about each site's contribution of patients who enter into care during the study will be provided in the statistical analysis plan. As described in 4.8.1.2, an imputation scheme for assessing VL suppression during the 12 months of the assessment that avoids the bias of missing assessments will be applied to all patients in care.

The estimate of intervention effect is the odds ratio of VL suppression in the FI intervention compared to SOC arms, assuming both a within-person and within-clinic correlation, stratified by city, using GEE methods. Sensitivity analysis will be conducted to assess the impact of patients who switch providers or establish care at multiple providers during the trial.

4.8.4.2 Feasibility Endpoints

Descriptive statistics will be used to summarize the mean uptake and overall cost of FIs and its variation between sites by site and population characteristics.

4.9 Human Subjects/Ethical Considerations

The Viral Suppression component of the study involves an intervention using FIs to encourage HIV-positive individuals on ART to be adherent to their medications and to maintain HIV VL suppression. This component is public health research. In this component, HIV care sites will be randomized, not individuals, and no de novo individual-level data will be collected. Instead, (de-identified) HIV surveillance data routinely collected by the DOH in the two communities will be analyzed. Because this research involves minimal risk and would be impracticable with informed consent, a waiver of patient informed consent will be requested under 45 CFR 46.116 (c) or (d).

The protocol will be submitted to appropriate IRBs (a central and/or local site IRBs) for ethical review prior to study initiation. Any subsequent modifications to the protocol will be submitted to appropriate IRBs, and, at a minimum, the protocol will be submitted annually for continuing review and approval by these same ethics boards.

No individual data, other than what exists in the surveillance databases, will be collected from study participants in the viral suppression component of this study. To assess the impact of the study on viral suppression in the communities of Washington, D.C. and the Bronx, surveillance data routinely collected by the DOHs will be analyzed. Data from the DOHs will be provided to the study team in a de-identified fashion, as per usual HIV surveillance procedures.

No study-specific laboratory testing will be conducted under this protocol. Therefore, no additional study-related test results will need to be reported to authorities. HIV testing and care data collected during routine clinical care will be reported per local HIV and AIDS reporting requirements.

4.10 Safety Monitoring and Adverse Event Reporting

The study team will not collect or report Adverse Events because there is no biomedical intervention. However, the team will collect and report all social harms that are brought to the attention of study staff members, using a study-specific incident report form. This form will be anonymous and will query common social harms such as altered personal relationships, forced change in housing, and physical violence. The form will also include space for a written narrative to document additional details of any social harm experienced. All research staff will be trained to properly complete the form. As a part of study training, research staff will also be trained on the provision of referrals to counseling and social service support. Reports of social

harms will be reviewed quarterly or more often, if indicated, and reported to the medical officer together with any actions that are taken. Social harms will be summarized and reported to appropriate IRB(s) on an annual basis.

5.0 PREVENTION FOR POSITIVES

5.1 Study Objectives for Prevention for Positives

The primary objective of the Prevention for Positives study component is to evaluate the effectiveness of a computer-delivered counseling intervention (“CARE+ Prevention”) containing Prevention for Positives messages in addition to SOC compared to SOC alone. Both intervention and control groups will have access to available SOC support and counseling services available to HIV-positive patients at the participating HIV care sites. All participants will have regular assessment of risk behaviors. The objective for this study component will be measured by comparing key self-reported HIV-transmission sexual risk behaviors and condom use errors.

5.2 Design for Prevention for Positives

The Prevention for Positives component of the study is a two-arm, individually randomized, prospective, effectiveness trial to be conducted at 12 HIV care sites (6 within each intervention community). This component will compare CARE+ containing Prevention for Positives messages in addition to SOC with SOC alone. All consenting individuals will be randomly assigned to one of the two arms of the study. In this component of the study, participant informed consent will be obtained for all participants in both of the study arms. Limited medical records data pertaining to HIV disease and individual data will be abstracted and analyzed in this study component.

5.3 Study Population for Prevention for Positives

The Prevention for Positives component of the study will include HIV-positive patients at a select number of participating HIV care sites. These patients will include individuals newly diagnosed with HIV and linked to care, and those established in care. ART use is not required for participation.

5.3.1 Inclusion Criteria

The inclusion criteria for this study component are as follows:

- All individuals who are permitted to consent for HIV care according to New York State or Washington, D.C. law
- Receiving care at the selected HIV care sites in the Bronx or Washington, D.C.
- Have attended the clinic one or more times in the last four months
- Able to understand either spoken English or Spanish

- Able and willing to provide informed consent

Subjects enrolled into the Prevention for Positives component of the study will participate in the patient survey (see Section 6.0).

5.3.2 Exclusion Criteria

The exclusion criteria for this study component are the following:

- Not seen in the clinic in the last four months
- History or evidence of altered mentation, inebriation or substance use that would interfere with participation in the study
- Unable or unwilling to provide informed consent
- Participation in another study focusing on HIV prevention for positives

5.4 Study Sites for Prevention for Positives

Participants will be recruited for the Prevention for Positives component of the study from six HIV care sites (three FI sites and three SOC of care sites) in each intervention city.

The three sites among the FI sites and the three sites in the SOC sites in each city will be selected based on the following criteria:

- The highest volume of HIV-positive patients in care
- Site agreement to participate in this component of the study

5.5 Intervention for Prevention for Positives

A modified version of CARE+ for HIV-positives will be used with Audio Computer Assisted Self-Interviewing (ACASI) to measure behavioral risk and to compare a full computer counseling intervention (assesses self-efficacy and motivation, provides tailored feedback on specific risk behaviors, shows skill-building videos, and helps the user make a risk-reduction plan) to a control session consisting only of ACASI ascertainment of risk behavior.

An additional component will be the utilization of the computer-based system described above to measure factors that impact both the acceptability of the interventions utilized in this study and the feasibility of future TNT strategies. Other data, beyond those that can be obtained from routine HIV surveillance data, will be captured including the following:

- HIV testing history and prior experience with linkage/drop out from care (if applicable)

- CD4 cell count at ART initiation (via chart abstraction, not routinely available from HIV surveillance), at study enrollment, and then every 3 months up to and including month 18

- VL at ART initiation (via chart abstraction, not routinely available from HIV surveillance), at study enrollment, and then every 3 months up to and including month 18

CD4 cell counts and HIV RNA levels obtained from month 0 through month 18, as well as CD4 cell count and HIV RNA levels at time of ART initiation, will also be extracted from participant's medical records to enable individual-level subgroup analyses of adherence and virologic responses among CARE+ participants who are receiving versus not receiving FIs for virologic suppression, as well as other subgroup analyses

- Behaviors likely to transmit HIV (sexual and parenteral)

- Receipt of and experience with services to promote retention and adherence

- Knowledge of, attitudes towards, and the feasibility of early initiation of ART

- Knowledge of and attitudes towards use of ART for prevention in partners

5.6 Study Procedures for Prevention for Positives

5.6.1 Recruitment Process

Study staff at the HIV care sites will be trained for the Prevention for Positives study procedures and on human subject requirements. Using a randomized approach algorithm, study staff will sequentially approach potential subjects in the waiting room and inform them of the study using a brief, standardized recruitment script. Those individuals refusing study participation will be asked if they will provide brief, anonymous demographic data, to assess comparability to study participants. Persons who are interested in the study will be assessed for eligibility and consented in a private area of the clinic. The study staff will provide information to the participant on the use of the tablet computer and headphones, and assist the participant with the anonymous ID log-in and beginning of the session, after which the staff person will remain nearby only to provide assistance if the participant requests it.

5.6.2 Co-Enrollment Guidelines

Prevention for Positives study participants will be allowed to be enrolled in other studies except those focused on reducing the risk of HIV transmission.

5.6.3 Participant Retention

Once a participant enrolls in the Prevention for Positives component of this study, the research staff will make every effort to retain him/her for 18 months of follow-up in order to minimize possible bias associated with loss to follow-up.

5.6.4 Participant Withdrawal

Participants may voluntarily withdraw from the study for any reason at any time. Participants also may be withdrawn by the study sponsor, site IRBs, government or regulatory authorities, the Investigator of Record (after consultation with the Protocol Chair), or Division of AIDS (DAIDS) Medical Officer.

Participants who elect to withdraw from the study prior to month 12 will be asked to complete one last computer session, however, participants will not be required to do so. Study staff will record the reason(s) for all withdrawals from the study in participants' study records.

5.7 Study Duration for Prevention for Positives

Duration of the Prevention for Positives study component is 18 months.

Patients enrolled in the Prevention for Positives component of the study will complete the CARE+/ACASI tool at baseline (month 0) and every three months thereafter for one year (at months 3, 6, 9, and 12). Patients will also be assessed again at 18 months (six months after the 12 month time point) to ascertain durability of effect.

5.8 Safety Monitoring and Adverse Event Reporting

Physical and psychological risks of this study are expected to be minimal, as no medical/surgical or pharmacologic procedures are planned. The Prevention for Positives study component addresses sensitive behaviors around sex and social interactions. Questions regarding sex may cause embarrassment or discomfort. Participants will be informed in writing and verbally that they may skip any questions and/or drop out of the study at any time without repercussion. Mental stress identified in the course of the study, such as depression, suicidality and intimate partner violence (IPV) will be followed-up appropriately, with counseling services available through the clinics. Counseling services for any other issues may be requested by participants during their routine appointment at the HIV care site.

Researchers will do everything possible to emphasize and maintain the confidentiality of participants, and safeguards for protecting confidentiality of data will be strictly enforced.

The study team will not collect or report Adverse Events because there is no biomedical intervention. However, the team will collect and report all social harms that are brought to the attention of study staff members, using a study-specific incident report form. This form will be anonymous and will query common social harms such as altered personal relationships, forced change in housing, and physical violence. The form will also include space for a written narrative to document additional details of any social harm experienced. All research staff will be trained to properly complete the form. As a part of study training, research staff will also be trained on the provision of referrals to counseling and social service support. Reports of social harms will be reviewed quarterly or more often, if indicated, and reported to the medical officer together with any actions

that are taken. Social harms will be summarized and reported to appropriate IRB(s) on an annual basis.

5.9 Statistics and Data Analysis for Prevention for Positives

5.9.1 Endpoints

The primary endpoint for the effectiveness of the Prevention for Positives component of the study is the following:

- The proportion of participants reporting any unprotected vaginal or anal sex in the previous three months

The secondary endpoints for the effectiveness of CARE+ Positive Prevention component of the study are the following:

- The number of sex partners of discordant or unknown HIV status with whom participant had unprotected vaginal or anal sex in the previous three months

- The number of sex partners with whom the participant had unprotected sex in the previous three months

- The number of persons with whom the participant shared needles after self use in the previous three months

At month 18, 6 months after the intervention ends, the above endpoints will be analyzed to determine the durability of any observed effects.

5.9.2 Accrual, Follow-up, and Sample Size

The primary behavioral endpoint of this study component is the proportion of patients reporting any unprotected vaginal or anal sex in the previous three months. In the Strategies for Management of Antiretroviral Therapy (SMART) study, at 11.2% of visits participants reported any unprotected anal or vaginal sex in the previous two months. Using post-enrollment data; 5.4% of patient visits included a report of unprotected anal or vaginal sex with an HIV-uninfected partner in the previous two months.

Individual randomization will occur, potentially stratified by gender.

Using a 2-sided alpha of 5%, and calculating the sample size required to achieve 90% power to detect differences in percentages of participant visits where high-risk behaviors are reported in each arm, Table 4 presents a number of assumptions. A within-person, ICC of 0.3-0.4 is assumed, based on data from the SMART trial behavioral endpoints.

Assuming at least one follow-up visit for 90% of enrollees, and an average number of visits per participant of 3.75 amongst retained participants, the study will enroll 660 per arm (1320 in total) to allow for loss due to attrition and loss to follow-up. It is estimated that around 110 participants will be recruited per HIV care site, with a target enrollment

of two subjects/day per site. Target enrollment should be reached within eight-12 months of initiation.

It is anticipated that a total of 1320 individuals will be randomized. Data will be collected from each person an average of 4 times during the study (out of four possible visits following enrollment at month 0, *i.e.* months 3, 6, 9, and 12). Allowing for loss to follow-up, with 522 people available for assessment per arm, the study has 90% power to detect a decrease from 11% to 8% in proportion of patients reporting any unprotected vaginal or anal sex at a given visit during the study.

Table 4: Per-Arm Sample Size Required for 90% Power to Detect Difference in Proportion Reporting High Risk Behavior at a Visit, with Two-sided Alpha of 5%, Assuming Four Visits per Participant

Control (%)	Intervention (%)	Mean Number of Visits	Intra-class Correlation	Number of People per Arm	Intra-class Correlation	Number of People per Arm
5.4%	3.0%	4	0.4	378	0.3	373
5.4%	3.5%	4	0.4	638	0.3	630
5.4%	4.0%	4	0.4	1240	0.3	1223
7.5%	4.0%	4	0.4	243	0.3	238
7.5%	4.5%	4	0.4	344	0.3	337
7.5%	5.0%	4	0.4	515	0.3	505
11%	7.5%	4	0.4	385	0.3	374
11%	8.0%	4	0.4	537	0.3	522
11%	8.5%	4	0.4	792	0.3	769

5.9.3 Randomization Scheme

Randomization to intervention (SOC and full CARE+ Prevention counseling session) or control arm (SOC alone) is done automatically within the software application using a pseudo-random number generator.

5.9.4 Data Analysis

Data to be analyzed for this study component will be obtained from the CARE+ electronic storage system and abstracted from participant's medical records. As participants proceed through the CARE+ intervention, their answers will automatically be stored electronically in a secure system. Access to the storage system will be limited to essential personnel. CD4 cell counts and HIV RNA levels obtained from month 0 through month 18, as well as CD4 cell count and HIV RNA levels at time of ART initiation, will be extracted from participant's medical records to enable individual-level subgroup analyses of adherence and virologic responses among CARE+ participants who are receiving versus not receiving FIs for virologic suppression, as well as other subgroup analyses. CD4 cell count and HIV RNA levels at ART initiation cannot be ascertained from routine surveillance data.

The primary behavioral endpoint of this study component is the proportion reporting any unprotected vaginal or anal sex in the previous three months.

Effectiveness of the CARE+ Prevention for Positives component will assess differences in proportion reporting self-reported risk behavior since the last visit for participants in the CARE+ Prevention arm compared to the control arm. GEE methods for repeated measures will be used to assess the difference in proportion reporting high-risk behavior between the two study arms for participants. Major subgroup analyses for those on ART and not on ART will be conducted. Similarly, GEE methods for repeated measures will be used to compare self-reported correctness and consistency of condom use data from individuals enrolled in the CARE+ Prevention arm with the control arm.

5.10 Human Subjects/Ethical Considerations

In the Prevention for Positives component of the study, a subset of patients enrolled at select HIV care sites in the two intervention communities will be randomized either to an intervention arm (receiving SOC prevention activities plus a computer-delivered intervention) or to the control arm (receiving only the SOC prevention activities at the care site). Individual-level data will be collected and analyzed. This component is a public health research requiring informed consent. Written informed consent will be obtained from participants.

The protocol and supporting study materials (the informed consent form and any advertising materials) will be submitted to appropriate IRBs (a central and/or local site IRBs) for ethical review prior to study initiation. Any subsequent modifications to these materials will be submitted to appropriate IRBs, and, at a minimum, they will be submitted annually for continuing review and approval by these same ethics boards.

Written informed consent will be obtained from each study participant (or a mark for those who are illiterate, which will be witnessed by a third party) prior to study enrollment. Each study site is responsible for developing an informed consent form for local use, based on the template provided with this protocol, that describes the purpose of the study, the study procedures and the risks and benefits of participation, in accordance with all applicable regulations. Participants will be provided with a copy of their informed consent form if they wish to receive it.

A small incentive will be provided to study participants in the Prevention for Positives component to compensate them for transportation and time.

All study-related information will be stored securely at the study site in areas with access limited to study staff. To maintain participant confidentiality, a coded number will identify all study data and administrative forms. All local databases will be secured with password-protected access systems. Forms, lists, logbooks, appointment books and any other listings that link participant ID numbers to other identifying information will be stored in a separate area with limited access. A participant's study information will not be released without the written permission of the participant, except as necessary to: authorized medical care providers; monitors for the National Institute of Allergy and Infectious Diseases (NIAID) and/or its contractors; representatives of the HPTN Coordinating and Operations Center (CORE) and/or the (Statistics and Data Management Center) SDMC; other government and regulatory authorities; and/or the site IRB.

No study-specific laboratory testing will be conducted under this protocol. Therefore, no additional study-related test results will need to be reported to authorities. HIV care data collected during routine clinical care will be reported per local HIV and AIDS reporting requirements.

6.0 SURVEY OF PATIENTS AND PROVIDERS

6.1 Study Objectives for Survey of Patients and Providers

The objectives of the survey of patients and providers study component are the following:

- To assess knowledge, attitudes and practices with regard to ART initiation, the potential of starting ART at higher CD4 cell counts, and the use of ART for prevention of HIV transmission

- To ascertain key socio-demographic and behavioral characteristics of study participants that cannot be obtained from routine HIV surveillance data

- To assess the acceptability and attitudes towards FIs

6.2 Design for the Survey of Patients and Providers

All participants in the Prevention for Positives component of the study will complete a patient survey module prior to and at the end of that study component. The survey will assess knowledge and attitudes regarding the use of ART in HIV disease and other key factors related to the feasibility and acceptability of the study's interventions.

Clinical providers who prescribe ART at HIV care sites in the intervention communities will be invited to complete a survey regarding their knowledge, attitudes and practices about the use of ART in HIV patients as well as FIs. Clinical providers will be surveyed prior to and following the viral suppression intervention to assess trends in the provider knowledge, attitudes and practices.

6.3 Study Population for the Survey of Patients and Providers

The patient survey component of the study will include HIV-positive patients at select HIV care sites that are participating in the Prevention for Positives component of the study.

Prescribing clinical providers (*e.g.* physicians, nurse practitioners and/or physician assistants) at select HIV care sites in the Bronx and Washington, D.C. will participate in provider survey.

6.3.1 Inclusion Criteria

The inclusion criteria for the patient survey are identical to those for the Prevention for Positives component of the study.

Prescribing clinical providers (*e.g.* physician, nurse practitioner/nurse-midwife, or physician assistant) at select HIV care sites are eligible for the provider survey.

6.3.2 Exclusion Criteria

The exclusion criteria for the patient survey are identical to those for the Prevention for Positives component of the study.

There are no exclusion criteria for the provider survey.

6.4 Study Sites for the Survey of Patients and Providers

The patient survey will be conducted at those HIV care sites involved in the Prevention for Positives component. The provider survey will survey providers from participating HIV care sites in the Bronx, NY and Washington, D.C.

6.5 Study Procedures for the Survey of Patients

Once consented and enrolled in the Prevention for Positives component, the clinic study staff will train participants how to use the computer tablet. Randomization to intervention (SOC plus full CARE+/ACASI counseling session) or control arm (SOC alone) is done automatically within the software application following the user's login with an anonymous study ID. Up to 110 individuals will be assigned to each arm, per site. At the end of the first (baseline, month 0) session, both arms will be presented, via the computer tool, with a series of questions (the patient survey). This patient survey module will be repeated only one more time, at the end of the intervention (month 12).

6.6 Study Procedures for the Survey of Providers

Providers at participating HIV care sites in both cities will be recruited by an introductory letter followed by up to two e-mail reminders, then two phone calls by study staff, asking them to take the brief survey on a secure, anonymous website.

Providers who go to the Web site will read and click on a brief consent, then complete a short survey assessing attitudes and practices.

At the completion of the survey, a printable coupon, compensating them for their time will be generated.

6.7 Study Duration for the Survey of Patients and Providers

Participants in the patient survey will complete the survey upon enrollment in the Prevention for Positives study and at the end of that study component.

The provider survey will also take place twice, once at the time of initiation of the overall HPTN 065 study and at the end of the viral suppression component.

6.8 Statistics and Data Analysis for Surveys of Patients and Providers

Data for analysis of this study component will be derived from answers to survey questions provided by both the patients and providers. Patient surveys will be administered electronically, after the CARE+ intervention at the beginning and at the end of the intervention. All patient responses will be stored in an electronic and secure database. Limited personnel will have access to this database. Provider surveys will be administered over the Internet. The survey will be secure and responses will also be stored securely. Survey data will be provided to the Statistical Center for HIV/AIDS Research and Prevention (SCHARP) for the following analyses:

- To assess baseline knowledge, attitudes and practices regarding the use of ART and other key factors related to the feasibility and acceptability of the study interventions in the subset of survey participants in the Prevention for Positives study component

  Descriptive statistics will be used to summarize the survey participants' attitudes regarding the use of ART and other key factors related to the feasibility and acceptability of the study interventions by site and combined.

- To compare the providers' knowledge, attitudes and practices about the use of ART, assessing these at study baseline and at study end at HIV care sites in the intervention communities

  Descriptive statistics will be used to summarize the prescribing providers' knowledge, attitudes and practices about the use of ART, at study baseline and study end by site and combined. Chi-squared statistic and paired test statistics will be used to compare the baseline data with study end data.

All Prevention for Positives data collected on the tablet computer will be password security-protected (anonymous study ID assigned timed to the millisecond of initial user log-in, with no

way for patients to access other user's data). All study data will be accessible only to investigators, study staff and biostatisticians assisting with the analysis. The CARE+ Prevention application will reside on each tablet computer with encrypted data transferred to a secured server at each clinic. The session database will be backed up by study staff. The 12 HIV care sites will send their study data to a SCHARP server on a routine basis. Only authorized study staff members are able to log onto the tablet computers or to access the master study database.

6.9 Human Subjects/Ethical Considerations

All patients participating in the Prevention for Positives study component will be administered the Patient Survey, regardless of whether or not they are randomized to the CARE+/ACASI intervention. Individual-level data will be analyzed. This component is human subjects research. Written informed consent will be obtained from participants at the time of consenting for participation in the Prevention for Positives study component.

Providers from participating HIV care sites will be invited to complete a Web-based survey regarding their knowledge, attitudes and practices concerning ART for treatment, frequency of HIV testing, and limited sociodemographic information. The survey will be anonymous, and will collect no identifying information. This component is public health research. Informed consent will be obtained from the provider's completing the survey. Before the survey will display, providers will read the consent and if they agree to participate, will indicate their agreement by clicking a button labeled "I agree."

The protocol and supporting study materials (the informed consent form and any advertising materials) will be submitted to appropriate IRBs (a central and/or local site IRBs) for ethical review prior to study initiation. Any subsequent modifications to these materials will be submitted to appropriate IRBs, and, at a minimum, they will be submitted annually for continuing review and approval by these same ethics boards.

Written informed consent (or a mark for those who are illiterate, which will be witnessed by a third party) will be obtained from each study participant willing to complete the patient survey prior to study enrollment. The informed consent for the survey is incorporated into the consent form for the Prevention for Positives component. Each study site is responsible for developing an informed consent form for local use for the patient survey, based on the template provided with the protocol, that describes the purpose of the survey, the survey procedures, and the risks and benefits of participation, in accordance with all applicable regulations. Participants completing the patient survey will be provided with a copy of their informed consent form if they wish to receive it.

No physical or psychosocial risks are anticipated from participation in the patient or provider surveys as they involve no invasive procedures or reports of illegal or socially stigmatized behaviors. Further, all data will be anonymized once merged into the study database, and no names will be used in publications.

A small incentive will be provided to survey participants, both providers and patients, to compensate them for their time. For the patients, this compensation is included in the incentive they receive for participating in the Prevention for Positives component of the study.

For patient surveys, all study-related information will be stored securely at the study site in areas with access limited to study staff. To maintain participant confidentiality, a coded number will identify all study data and administrative forms. All local databases will be secured with password-protected access systems. Forms, lists, logbooks, appointment books and any other listings that link participant ID numbers to other identifying information will be stored in a separate area with limited access. Survey information will not be released without the written permission of the participant, except as necessary to: authorized medical care providers; monitors of the NIAID and/or its contractors; representatives of the HPTN CORE and/or the SDMC; other government and regulatory authorities; and/or the site IRB.

6.10 Safety Monitoring and Adverse Event Reporting

The study team will not collect or report Adverse Events because there is no biomedical intervention. However, the team will collect and report all social harms that are brought to the attention of study staff members, using a study-specific incident report form. This form will be anonymous and will query common social harms such as altered personal relationships, forced change in housing, and physical violence. The form will also include space for a written narrative to document additional details of any social harm experienced. All research staff will be trained to properly complete the form. As a part of study training, research staff will also be trained on the provision of referrals to counseling and social service support. Reports of social harms will be reviewed quarterly or more often, if indicated, and reported to the medical officer together with any actions that are taken. Social harms will be summarized and reported to appropriate IRB(s) on an annual basis.

7.0 HIV SURVEILLANCE, ROUTINELY-COLLECTED AND OTHER SURVEY DATA

Use of HIV case and behavioral surveillance data and HIV testing data will be used for site selection in intervention communities and for process and outcome measures.

We will assess completeness, accuracy, timeliness and performance of the surveillance data.

7.1 HIV Testing Data

HIV testing data will be used to select HIV test sites for randomization to the intervention vs. SOC arm, and as a process measure for the testing intervention in ED and inpatient facilities (Table 5). For selection of HIV test sites for interventions, information on HIV testing is available for HIV test sites that are publicly supported through federal funds in each of the six communities included in this study; additional data are available for other HIV test sites supported in some local jurisdictions, such as NYC's AIDS Institute Reporting System. This includes the majority of HIV testing conducted by CBOs.

Table 5. Data Sources and Definitions

Data Sources and Definitions			
**Information needed**	**Description**	**Data source**	**Variables**
**HIV Testing Data**			
**Site selection, intervention communities**			
Volume of testing	Number of persons tested in XX year, by testing site	Local PEMS, local testing data	Name of testing facility, testing date
	Number of new diagnoses, by testing site	HIV surveillance data	Name of facility of diagnosis, date of diagnosis
	Where available, number of case reports to surveillance, by testing site	HIV surveillance data	Name of facility of diagnosis, date of diagnosis
**Process measures for HIV testing interventions, intervention communities**			
Number of persons eligible	Number of persons eligible	Project specific collection at intervention sites	Data may or may not already be collected at selected sites. If not collected, collection will be implemented for this study
Number of persons tested	Number of persons tested	Project specific collection at intervention sites	Data may or may not already be collected at selected sites. If not collected, collection will be implemented for this study
Number of new diagnoses	Number of new diagnoses	HIV surveillance data, and project specific collection at intervention sites	Surveillance data: name of testing facility, testing data. Data may or may not be collected by selected sites, and if not collected, can be implemented at site or use surveillance data.
**HIV Surveillance Data**			
**Outcome Measures**			
Number/proportion of persons newly diagnosed who entered care, by site	CD4 or VL within 3 months of diagnosis (CD4/VL date not equal to diagnosis date), by site	HIV surveillance data	HIV diagnosis date, name of testing site, VL date, VL result, CD4 date, CD4 result, demographics (DOB, sex, race/ethnicity, transmission category)

Data Sources and Definitions			
**Information needed**	**Description**	**Data source**	**Variables**
Number/proportion of previously diagnosed persons not in care entered into care, by site	CD4 or VL within 3 months of diagnostic test, among persons with previous diagnostic test and no CD4/VL within the past year, by site	HIV surveillance data	HIV diagnosis date, name of testing site, VL date, VL result, CD4 date, CD4 result, demographics (DOB, sex, race/ethnicity, transmission category)
VL suppression	Probability of undetectable VL amongst people established in care with previous VL/CD4, by site	HIV surveillance data	Name of treatment site, VL date, VL result, CD4 date, CD4 result, demographics (DOB, sex, race/ethnicity, transmission category)
**Behavioral Data**			
Number/proportion of persons who had an HIV test in the past 12 months	See BRFSS 2009 questionnaire:<br>20.1 Have you ever been tested for HIV? Do not count tests you may have had as part of a blood donation. Include testing fluid from your mouth.<br>(213)<br>1 Yes<br>2 No [Go to Q20.5]<br>7 Don't know / Not sure [Go to Q20.5]<br>9 Refused [Go to Q20.5]<br>20.2 Not including blood donations, in what month and year was your last HIV test?<br>http://www.cdc.gov/brfss/questionnaires/pdf-ques/2009brfss.pdf	Local surveys (see descriptions below)	Testing, testing date
Special HPTN 065 emphasis population: number/proportion of MSM who had an HIV test in the past 12 months	NHBS questions	National HIV Behavioral Surveillance (NHBS) system	Testing, testing date

The CDC has developed a data entry and reporting system, the PEMS, to strengthen monitoring and evaluation of HIV prevention programs. PEMS is used by health departments and CBOs funded through CDC HIV-prevention cooperative agreements. PEMS allows grantees to collect agency data, community planning data, program plan data, and client-level data. This assures a comprehensive set of standardized variables are available.

The client-level data include information on testing site, client demographics, risk factors, test information and testing history. This allows a description, by site, of the number

tested and number positive, and a description of the population tested or positive. Additional variables that can be used to describe sites include agency characteristics (budget, sites, workers, contracts, network agencies), program plans (program models, target populations, interventions (CTR, Health Communications/Public Information Outreach, settings, sessions, activities), service delivery (service activities, recruitment, referrals), and community planning activities (target populations, priority interventions).

Some areas have testing report systems in addition to PEMS. For example, New York State collects testing data through the AIDS Institute Reporting System. Florida has PEMS data as well as a counseling and testing data base that includes all the variables needed to report an HIV case as well as testing history questions; these two data sources cover all clients tested in the public sector, *e.g.*, registered counseling and testing sites (AIDS Service Organizations (ASOs), CBOs, faith-based initiatives), and county health department clinics (STD, TB, Family planning, *etc*.).

Another testing measure for selection of intervention sites that can be derived from surveillance data is the number of new diagnoses by site.

Process measures (Table 5) to monitor testing at the testing intervention sites will be collected directly from the participating facilities.

7.2 HIV Surveillance Data

The CDC's national system for the surveillance of HIV infection is based on mandatory name-based reporting of all HIV and AIDS cases in every state and Washington, D.C. Health departments maintain case records of every new and established HIV case according to the HIV case definition. Laboratory reports of positive HIV test results and provider reports of new confirmed diagnoses are tracked by the health departments. In most jurisdictions, data from mandatory reporting of CD4 cell count and VL from all laboratories are linked via case names to individuals maintained in the eHARS data entry and reporting system (not all states have mandatory reporting of all VL and CD4 cell count). Across the United States, and within each community, the system captures key variables for this study. The key data elements from HIV surveillance that will be used are listed in Table 6.

Table 6. Key Date Elements and Primary Use

Key Data Elements	Primary Use
CD4 result<br>CD4 date<br>VL result<br>VL date<br>Name of facility of diagnosis<br>Name of treatment facility (lab order)<br>Type of HIV test<br>HIV test date<br>HIV test result<br>HIV diagnosis date<br>AIDS diagnosis date	Study intervention outcome measures

Key Data Elements	Primary Use
Date of birth (DOB)<br>Race/ethnicity<br>Transmission category<br>Sex at birth	Stratification variables
Date of death<br>Cause of death<br>Previous negative test, lab based<br>Previous negative test, self-reported	Additional outcome measures
Place of residence at diagnosis<br>Current place of residence	Adjustment variables
Source of report<br>Surveillance method<br>Duplicate	Variables for data quality control

De-duplication (removing double-reported cases) of cases between jurisdictions occurs at least annually, with case tracing on an ongoing basis as individuals relocate and new diagnostic labs are investigated in a new jurisdiction. Cases are identified through active follow-up of positive Enzyme Immunoassay (EIA), positive WB and detectable VL reports from names not matched in the local eHARS database, as well as through reporting by providers. Communities selected for this study (both intervention and non-intervention communities) have quality data collected on these parameters.

HIV surveillance data can describe treatment success based on reported VL measurements. However, specific information on type of treatment received is not collected.

HIV surveillance data have been evaluated on the national level and for individual state/local program areas for completeness, timeliness, and quality of individual data items (Buehler, Berkelman et al. 1992; Rosenblum, Buehler et al. 1992; Greenberg, Hindin et al. 1993; Meyer, Jones et al. 1994; Klevens, Fleming et al. 1998; Schwarcz, Hsu et al. 1999; Solomon, Flynn et al. 1999; Jara, Gallagher et al. 2000; Doyle, Glynn et al. 2002; Hall, Song et al. 2006) and through annual progress reports [unpublished]). For the accuracy of case counts, all surveillance programs conduct annual linkage of HIV cases to death records, intrastate duplicate reviews, and interstate duplicate resolution based on potential duplicate listings received from CDC.

CDC sets standards for the completeness and timeliness of case reporting as well as the completeness and accuracy of individual data elements, against which national and individual program data are evaluated each year (e.g., 2008 diagnosis data are currently evaluated using data reported through December 2009) (Hall and Mokotoff 2007; Hall, Song et al. 2008). For 2007 diagnoses, New York City, Philadelphia, and Florida case counts were estimated to be >95% complete by December 2008 (note, while the other cities were transitioning to the new data system and completeness was not formally assessed, they closely monitor expected case reports from laboratories and providers to assure complete reporting). More than 80% of cases are reported to the health departments within 6 months of diagnosis. Critical variables include sex assigned at birth, race/ethnicity, and age; completeness in these variables is very high (near 100% for sex and age; >90% for race/ethnicity) and active follow up is conducted to obtain these data elements; all cases must have a diagnosis date (test result or physician diagnosis,

according to the CDC case definition). Additional variables of importance include risk factor information, and CD4 and VL test results. Risk factor information is available for more than 60% of cases (some areas achieve better than 80%); CDC has developed methods to adjust for missing risk factor information in local or national analyses using multiple imputation.

The completeness of CD4 and VL results, critical to this study, depends on 1) state laboratory reporting laws and regulations; 2) reporting/abstracting from providers; and 3) whether patients entered care. Most important to the completeness of these data is the assessment of whether all laboratories routinely report to the health departments to assure that this information is reported for any patients entering care. For example, both New York State and DC have laws requiring reporting of all values of CD4 and VL test results. The New York State Health Department has identified all labs conducting testing for New York residents and monitors all labs to assure that reporting is complete and there are no missing data. Accuracy is also assessed by comparing reports from labs against what is obtained from chart review and re-abstraction. Regarding lag times, the mean time for New York City receiving Western Blot lab results is 21 days from diagnosis and CD4 and VL results 30 days from test date. (Please see Appendix for NYC example of additional indicators and evaluation of reporting completeness and timeliness). In DC, similar processes have been implemented. Regarding lag times in DC, approximately 85% of District laboratory reports are received within 2 weeks of the test date via electronic reporting. The remaining 15% are reported via the US mail, averaging about a 3 week lag in reporting. (Please see Appendix for DC indicators).

Data quality is continuously monitored during surveillance activities. Any notification (e.g., HIV diagnostic lab report) initiates data abstraction by field staff, with assignments within days of report. Abstracted data is checked for quality and the data entry system includes data quality checks. In order to assist in the timely input and generation of surveillance data during this study, resources will be provided for additional personnel in the DOHs in all participating cities (intervention and non-intervention). As part of TLC Plus, both process and outcome evaluations for data quality (accuracy, completeness, and lag times) will be conducted for intervention and non-intervention cities on a routine schedule.

CDC funds the Morbidity Monitoring Project (MMP) (McNaghten, Wolfe et al. 2007), an interview and medical-record-abstraction project, to obtain information on the following questions: Are patients receiving care and treatment in accordance with United States Public Health Service (USPHS) guidelines? Are patients receiving care in Ryan White-funded facilities receiving the same quality of care as patients in private facilities? What are the barriers to receiving care and services? Included is a locally and nationally representative sample of HIV-positive adults in care .with assessments of: adherence; sexual behavior; drug use; care-seeking; clinical outcomes; treatment; CD4 cell count and VL; opportunistic illnesses; type and quality of care received; and met and unmet needs for HIV care and prevention services. This information is limited in scope for the purposes of this study (funded locations, sites included, *etc*). However, an assessment will be made to determine the information that can be gained from MMP.

7.2.1 Human Subjects/Ethical Considerations

HIV case surveillance data are collected as part of routine HIV surveillance as mandated by state or local laws or regulations. Similarly, other data are routinely collected by DOHs.

According to CDC's Guidelines for Defining Public Health Research and Public Health Non-Research (CDC 1999) and Title 45 Part 46 of the Code of Federal Regulations (DHHS 2005), the CDC has determined that HIV surveillance is not a research activity, and, therefore, does not require review by an IRB.

State and local HIV surveillance programs must comply with federal security and confidentiality guidelines for collecting, storing and releasing data (CDC 2006).

Data are sent to the CDC without personal identifiers.

7.3 Behavioral Data

Information on population HIV-testing rates is generally available for the United States as a whole and for select communities that have implemented behavioral surveys that are representative for their community. The primary measures are the proportion of persons who ever tested for HIV and the proportion of persons who tested for HIV within the past year. This information is critical to monitor trends of HIV testing in the future.

One behavioral survey conducted in all states and Washington, D.C. is the Behavioral Risk Factor Surveillance System (BRFSS) funded by CDC (CDC, http://www.cdc.gov/BRFSS/). BRFSS is an on-going, telephone, health survey system, tracking health conditions and risk behaviors in the United States yearly since 1984. Currently, data are collected monthly in all 50 states, Washington, D.C., Puerto Rico, the United States Virgin Islands and Guam. State and local areas may add questions to the standard questionnaire. However, oversampling of local areas is needed to make inferences for areas smaller than statewide, and representativeness/response rates have diminished with the widespread use of cell phones. Surveys conducted in the local areas are described in Table 5. One limitation of BRFSS surveys is the diminishing population reached using land-lines; therefore, any BRFSS-type survey used for this project should include cell phones.

Our approach for this study will be to support currently planned surveys to obtain representative HIV testing data from the communities.

Accuracy of recall of HIV testing will not be assessed as part of this study.

This study also includes a special emphasis population, MSM. The National HIV Behavioral Surveillance (NHBS) system is a CDC-funded project conducted in 25 cities in the United States, including Washington, D.C. The purpose of this serial cross-sectional study is to yield information about what people do that puts them at risk for HIV. NHBS has three cycles focusing on different risk groups: NHBS-MSM, injecting drug users (NHBS-IDU), and heterosexuals at risk of HIV infection (NHBS-HET).

Areas conducting NHBS will be able to obtain HIV testing data among MSM in the MSM cycle years.

7.3.1 Behavioral Data Collection in Washington, D.C.

Washington, D.C. implements HIV testing questions through the BRFSS and will include cell phones in 2009. Washington, D.C. also collects behavioral data via NHBS.

7.3.2 Behavioral Data Collection in New York City

NYC conducts the NYC Community Health Survey (CHS), a telephone survey conducted annually. CHS provides robust data on the health of New Yorkers, including neighborhood, borough and citywide estimates on a broad range of chronic diseases and behavioral risk factors.

The CHS is based on the BRFSS. The CHS is a cross-sectional survey that samples approximately 10,000 adults aged 18 and older from all five boroughs of NYC—Manhattan, Brooklyn, Queens, Bronx and Staten Island.

A computer-assisted telephone interviewing (CATI) system is used to collect survey data from respondents accessed by random-digit dialing of household-based land lines. Interviews are conducted in a variety of different languages.

All data collected are self-report.

Questions on HIV testing are included. NYC also collects behavioral data via NHBS.

7.3.3 Behavioral Data Collection in Miami

In Miami, behavioral data is collected via NHBS and BRFSS. BRFSS collected county data in 2007, but not in 2008 and 2009. County data will be collected again in 2010. However, representativeness for Miami and inclusion of cell phones need to be determined.

7.3.4 Behavioral Data Collection in Philadelphia

In Philadelphia, behavioral data is collected via NHBS and BRFSS/Southeastern Pennsylvania Household Health Survey (PHHS).

Public Health Management Corporation’s (PHMC) Southeastern Pennsylvania Household Health Surveys are extensive health surveys that provide timely information on more than 13,000 residents, both children and adults, living in Bucks, Chester, Delaware, Montgomery and Philadelphia counties. The survey targets key information about health status, personal health behaviors, and access to and utilization of area health services. These data are available at the census tract, ZIP code, county and regional level. The Household Health Survey provides primary data on a broad range of health topics such as health status, access to care, utilization of services, personal health behaviors, health screening information, health insurance status, women's health, child health, and older adult health and social support needs. The survey asks whether persons

have had an HIV test. The survey includes responses from more than 10,000 households in the region, representing more than 13,000 adults and children.

Interviews are conducted by telephone using a random-digit dial methodology. Adult and child respondents are selected randomly using the "last birthday" method.

7.3.5 Behavioral Data Collection in Chicago

In Chicago, behavioral data is collected via NHBS. The state conducts BRFSS and data is available for Chicago. However, representativeness for Chicago and inclusion of cell phones need to be reviewed.

7.3.6 Behavioral Data Collection in Houston

In Houston, behavioral data is collected via NHBS. The state conducts BRFSS. However, representativeness for Houston and inclusion of cell phones need to be determined.

8.0 ADMINISTRATIVE PROCEDURES AND OPERATIONAL CONSIDERATIONS

This study differs from other HPTN studies to date. In fact, it differs from most other studies conducted through HIV clinical trials networks. It is an ambitious effort that aims at identifying a strategy to tackle the entrenched HIV epidemic in some communities in the United States. It differs from traditional clinical trials in the following manners:

- The study will be a collaborative effort among the HPTN, the CDC, and the DOHs in the intervention and non-intervention communities.
- Many study outcomes will be based on data collected through surveillance systems in the public health programs of local health departments in the intervention and non-intervention communities.
- The study will include two site randomizations (one of HIV test sites and one of HIV care sites) as well as individual randomization in the Prevention for Positives cohort.
- The laboratory outcomes in this study will be based on standardized assays conducted in certified laboratories in the United States. No laboratory testing will be conducted at the HPTN Network Laboratory (NL).

The SDMC at SCHARP will assist in the design of the study, determine the sampling plan and coordinate the data collection from various sources. The SDMC will play the key role in communicating with staff responsible for surveillance in the jurisdictions where the study will be conducted. The SDMC will also lead data analysis efforts and generation of data reports.

The NL will assist in the design of the study as well as in guiding the conceptualization and selection of appropriate laboratory objectives and endpoints. The NL will provide expert input into the methodology for various assays, as needed. However, no assays will

be conducted at NL in the main part of the study. All laboratory data will be collected through surveillance systems or medical records abstraction (for the Prevention for Positives study component).

The HPTN CORE at Family Health International (FHI) will coordinate the development of the protocol and facilitate the implementation of the study in the intervention communities.

8.1 Study Activation

For this study, traditional DAIDS site activation processes will not be conducted. The study team will define a start date for the Expanded Testing component. The HPTN CORE will notify all participating HIV test sites when they may begin to distribute coupons to patients for the Linkage-to-Care study component and when participating HIV care sites may begin to distribute FIs for the Viral Suppression component.

Traditional participant informed consent will be obtained for the Prevention for Positives component and for the Patient Survey. Operationally, the Prevention for Positives intervention and the Patient Survey will be administered together during Month 0 and Month 12 of the Prevention for Positives study component. Therefore, a single comprehensive informed consent form was designed (see Appendix IIA). Following ethical review and approval, study sites will submit required administrative documentation — as listed in the study-specific procedures manual — to the HPTN CORE. CORE staff will work with study site staff and complete DAIDS protocol registration in accordance with the current DAIDS Protocol Registration Policy and Procedure Manual. Included in this step will be CORE and DAIDS review of each site-specific study informed consent form. Pending successful protocol registration and submission of all required documents, CORE staff will "activate" the site to begin study operations. Study implementation may not be initiated until a study activation notice is provided to the site.

The HPTN Core will also notify SCHARP and other network partners of dates that study components will start.

8.2 Study Coordination

Study implementation will be directed by this protocol as well as the SSP manual. The SSP manual will outline procedures for conducting study visits, data collection and processing, management and reporting, and other study operations.

Data for this study will be collected in three ways:

- The DOHs in Washington, D.C. and in the Bronx of NYC will provide aggregate data from their routine surveillance systems to the data management group for the study, SCHARP. SCHARP will conduct all data analyses.

- Data will also be collected from patients and providers using study-specific surveys. These data will also be submitted to SCHARP for analysis.

- For the Prevention for Positives study component, data will be collected by patient self-report and medical records abstraction. These data will be submitted to SCHARP.

Close coordination between protocol team members will be necessary to track study progress, respond to questions about proper study implementation and address other issues in a timely manner. Rates of accrual, adherence and follow-up will be monitored closely by the team as well as an HPTN study-monitoring committee (SMC) for the Prevention for Positives and survey components of the study.

8.3 Study Monitoring

This study will not undergo traditional HPTN monitoring by PPD Research Associates. However, the study will be carefully reviewed by an HPTN SMC on a regular basis. The SMC will monitor the quality of the data transmitted from the DOHs of the intervention and non-intervention communities. The SMC will also monitor for evidence of HIV-test-site and HIV-care-site migration due to the availability of FIs for linkage-to-care and viral suppression at some sites.

8.4 Protocol Compliance

The study will be conducted in full compliance with the protocol. The protocol will not be amended without prior written approval by the Protocol Co-Chairs and NIAID Medical Officer. All protocol amendments must be submitted to and approved by the relevant IRB(s) and the Division of AIDS (DAIDS) Regulatory Compliance Center (RCC) prior to implementing the amendment.

8.5 Human Subjects/Ethical Considerations

Expanded HIV testing. This component is public health practice. Social mobilization and emergency department testing is already taking place in the two intervention communities (Bronx and Washington DC) and is intended to be specific to the needs of those populations. While some observations from the overall TLC Plus project with regard to social mobilization and expanded testing may be applicable elsewhere in the United States, these activities were initiated originally for the specific benefit of the respective communities, and do not constitute research.

Linkage-to-Care. This component of the study involves a randomized intervention using financial incentives (FIs) to link HIV-positive individuals to HIV care after diagnosis. This component is public health research. In this component, HIV test sites will be randomized, not individuals, and no de novo individual-level data will be collected. Instead, (de-identified) HIV surveillance data routinely collected by the Departments of Health (DOH) in the two communities will be analyzed. Because this research involves minimal risk and would be impracticable with informed consent, a waiver of patient informed consent will be requested under 45 CFR 46.116 (c) or (d).

Viral Suppression. This component of the study involves an intervention using FIs to encourage HIV-positive individuals on ART to be adherent to their medications and to maintain HIV VL suppression. This component is public health research. In this component, HIV care sites will be randomized, not individuals, and no de novo individual-level data will be collected. Instead, (de-identified) HIV surveillance data routinely collected by the DOH in the two communities will be analyzed. Because this research involves minimal risk and would be impracticable with informed consent, a waiver of patient informed consent will be requested under 45 CFR 46.116 (c) or (d).

Prevention for Positives. In this component of the study, a subset of patients enrolled at select HIV care sites in the two intervention communities will be randomized either to an intervention arm (receiving computer-delivered intervention for sexual risk reduction plus SOC prevention activities) or to the control arm (receiving only the SOC prevention activities at the care site). Individual-level data will be collected and analyzed. This component is public health research requiring informed consent. Written informed consent will be obtained from participants.

Patient survey. All patients participating in the Prevention for Positives study component will be administered the Patient Survey. Individual-level data will be analyzed. This component is public health research. Written informed consent will be obtained from participants at the time of consenting for participation in the Prevention for Positives study component.

Provider survey. Providers from participating HIV care sites will be invited to complete a Web-based survey regarding their knowledge, attitudes and practices concerning ART for treatment, frequency of HIV testing, and limited sociodemographic information. The survey will be anonymous, and will collect no identifying information. This component is public health research. Informed consent will be obtained from the provider's completing the survey. Before the survey will display, providers will read the consent and if they agree to participate, will indicate their agreement by clicking a button labeled "I agree." Investigator's Records

Study-specific records will only be located at HIV care sites participating in the Prevention for Positives study component. At these sites, all study source documents (such as informed consent forms and patient ID linkage logs) will be maintained in a locked cabinet or room. Access to these records will be restricted to appropriate study staff members.

8.6 Use of Information and Publications

Publication of the results of this study will be governed by HPTN policies. Any presentation, abstract or manuscript will be submitted to the HPTN Manuscript Review Committee for review prior to submission. Similar review may be necessary by other collaborating organizations.

8.7 Study Discontinuation

The study may be discontinued at any time by NIAID, the HPTN, the Office for Human Research Protections (OHRP), government or regulatory authorities, and/or an IRB. However, should the study be stopped early, all sites and community partners would be

notified of such a decision, as well as the reasons behind it, prior to any termination activities.

9.0 REFERENCES

(CDC), C. f. D. C. a. P. (2009). "Late HIV testing - 34 states, 1996-2005." MMWR Morb Mortal Wkly Rep **58**(24): 661-5.

Aberg, J. A., J. E. Kaplan, et al. (2009). "Primary care guidelines for the management of persons infected with human immunodeficiency virus: 2009 update by the HIV medicine Association of the Infectious Diseases Society of America." Clin Infect Dis **49**(5): 651-81.

Anastos, K., M. F. Schneider, et al. (2005). "The association of race, sociodemographic, and behavioral characteristics with response to highly active antiretroviral therapy in women." J Acquir Immune Defic Syndr **39**(5): 537-44.

Applebaum, A. J., L. C. Reilly, et al. (2009). "The impact of neuropsychological functioning on adherence to HAART in HIV-infected substance abuse patients." AIDS Patient Care STDS **23**(6): 455-62.

Assefa, Y. and M. Lera (2009). "Universal voluntary HIV testing and immediate antiretroviral therapy." Lancet **373**(9669): 1080; author reply 1080-1.

Bakken, S., W. L. Holzemer, et al. (2000). "Relationships between perception of engagement with health care provider and demographic characteristics, health status, and adherence to therapeutic regimen in persons with HIV/AIDS." AIDS Patient Care STDS **14**(4): 189-97.

Bangsberg, D. R. (2008). "Preventing HIV antiretroviral resistance through better monitoring of treatment adherence." J Infect Dis **197 Suppl 3**: S272-8.

Bartlett, J. A. (2002). "Addressing the challenges of adherence." J Acquir Immune Defic Syndr **29 Suppl 1**: S2-10.

Berg, K. M., N. A. Cooperman, et al. (2009). "Self-efficacy and depression as mediators of the relationship between pain and antiretroviral adherence." AIDS Care **21**(2): 244-8.

Bigelow, G. E. and K. Silverman (1999). Theoretical and Empirical Foundations of Contingency Management Treatments for Drug Abuse. Motivating Behavior Change Among Illicit Drug Users. S. T. Higgins and K. Silverman. Washington, DC, American Psychological Association**:** 15-30.

Bradford, J. B., S. Coleman, et al. (2007). "HIV System Navigation: an emerging model to improve HIV care access." AIDS Patient Care STDS **21 Suppl 1**: S49-58.

Branson, B. M., H. H. Handsfield, et al. (2006). "Revised recommendations for HIV testing of adults, adolescents, and pregnant women in health-care settings." MMWR Recomm Rep **55**(RR-14): 1-17; quiz CE1-4.

Buchacz, K., R. K. Baker, et al. (2008). "Rates of hospitalizations and associated diagnoses in a large multisite cohort of HIV patients in the United States, 1994-2005." AIDS **22**(11): 1345-54.

Buehler, J. W., R. L. Berkelman, et al. (1992). "The completeness of AIDS surveillance." J Acquir Immune Defic Syndr **5**(3): 257-64.

Bunnell, R., J. P. Ekwaru, et al. (2006). "Changes in sexual behavior and risk of HIV transmission after antiretroviral therapy and prevention interventions in rural Uganda." AIDS **20**(1): 85-92.

Campsmith, M. L., P. Rhodes, et al. (2008). "HIV prevalence estimates--United States, 2006." MMWR Morb Mortal Wkly Rep **57**(39): 1073-6.

Castilla, J., P. Sobrino, et al. (2002). "Late diagnosis of HIV infection in the era of highly active antiretroviral therapy: consequences for AIDS incidence." AIDS **16**(14): 1945-51.

CDC. (1999). "Guidelines for Defining Public Health Research and Public Health Non-Research." Retrieved May 7, 2008, from http://www.cdc.gov/od/science/regs/hrpp/researchdefinition.htm.

CDC. (2006). "Technical Guidance for HIV/AIDS Surveillance Programs, Volume III: Security and Confidentiality Guidelines. ." Retrieved October 19, 2009, from http://www.cdc.gov/hiv/topics/surveillance/resources/guidelines/guidance/index.htm.

Cheng, D. M., H. Libman, et al. (2009). "Alcohol consumption and lipodystrophy in HIV-infected adults with alcohol problems." Alcohol **43**(1): 65-71.

Cohen, M. S., C. Gay, et al. (2007). "Narrative review: antiretroviral therapy to prevent the sexual transmission of HIV-1." Ann Intern Med **146**(8): 591-601.

Cohen, M. S., T. D. Mastro, et al. (2009). "Universal voluntary HIV testing and immediate antiretroviral therapy." Lancet **373**(9669): 1077; author reply 1080-1.

Craw, J. A., L. I. Gardner, et al. (2008). "Brief strengths-based case management promotes entry into HIV medical care: results of the antiretroviral treatment access study-II." J Acquir Immune Defic Syndr **47**(5): 597-606.

Crepaz, N., C. M. Lyles, et al. (2006). "Do prevention interventions reduce HIV risk behaviours among people living with HIV? A meta-analytic review of controlled trials." AIDS **20**(2): 143-57.

Cunningham, W. E., L. E. Markson, et al. (2000). "Prevalence and predictors of highly active antiretroviral therapy use in patients with HIV infection in the united states. HCSUS Consortium. HIV Cost and Services Utilization." J Acquir Immune Defic Syndr **25**(2): 115-23.

DHHS. (2005). "Code of Federal Regulations, Title 45 - Public Welfare, Part 46, Protection of Human Subjects. ." Retrieved May 7, 2008, from http://www.hhs.gov/ohrp/documents/OHRPRegulations.pdf.

Dieffenbach, C. W. and A. S. Fauci (2009). "Universal voluntary testing and treatment for prevention of HIV transmission." JAMA **301**(22): 2380-2.

Donatelle, R., D. Hudson, et al. (2004). "Incentives in smoking cessation: status of the field and implications for research and practice with pregnant smokers." Nicotine Tob Res **6 Suppl 2**: S163-79.

Donatelle, R. J., S. L. Prows, et al. (2000). "Randomised controlled trial using social support and financial incentives for high risk pregnant smokers: significant other supporter (SOS) program." Tob Control **9 Suppl 3**: III67-9.

Doyle, T. J., M. K. Glynn, et al. (2002). "Completeness of notifiable infectious disease reporting in the United States: an analytical literature review." Am J Epidemiol **155**(9): 866-74.

Duran, D., J. Beltrami, et al. (2008). "Persons tested for HIV--United States, 2006." MMWR Morb Mortal Wkly Rep **57**(31): 845-9.

Epstein, H. (2009). "Universal voluntary HIV testing and immediate antiretroviral therapy." Lancet **373**(9669): 1078-9; author reply 1080-1.

Fideli, U. S., S. A. Allen, et al. (2001). "Virologic and immunologic determinants of heterosexual transmission of human immunodeficiency virus type 1 in Africa." AIDS Res Hum Retroviruses **17**(10): 901-10.

Finkelstein, E. A., L. A. Linnan, et al. (2007). "A pilot study testing the effect of different levels of financial incentives on weight loss among overweight employees." J Occup Environ Med **49**(9): 981-9.

Friis-Moller, N., P. Reiss, et al. (2007). "Class of antiretroviral drugs and the risk of myocardial infarction." N Engl J Med **356**(17): 1723-35.

Gardner, L. I., L. R. Metsch, et al. (2005). "Efficacy of a brief case management intervention to link recently diagnosed HIV-infected persons to care." AIDS **19**(4): 423-31.
Gilbert, P., D. Ciccarone, et al. (2008). "Interactive "Video Doctor" counseling reduces drug and sexual risk behaviors among HIV-positive patients in diverse outpatient settings." PLoS One **3**(4): e1988.
Giordano, T. P., A. L. Gifford, et al. (2007). "Retention in care: a challenge to survival with HIV infection." Clin Infect Dis **44**(11): 1493-9.
Giordano, T. P., F. Visnegarwala, et al. (2005). "Patients referred to an urban HIV clinic frequently fail to establish care: factors predicting failure." AIDS Care **17**(6): 773-83.
Giuffrida, A. and D. J. Torgerson (1997). "Should we pay the patient? Review of financial incentives to enhance patient compliance." BMJ **315**(7110): 703-7.
Granich, R. M., C. F. Gilks, et al. (2009). "Universal voluntary HIV testing with immediate antiretroviral therapy as a strategy for elimination of HIV transmission: a mathematical model." Lancet **373**(9657): 48-57.
Greenberg, A. E., R. Hindin, et al. (1993). "The completeness of AIDS case reporting in New York City." JAMA **269**(23): 2995-3001.
Hall, H. I. and E. D. Mokotoff (2007). "Setting standards and an evaluation framework for human immunodeficiency virus/acquired immunodeficiency syndrome surveillance." J Public Health Manag Pract **13**(5): 519-23.
Hall, H. I., R. Song, et al. (2006). "Assessing the completeness of reporting of human immunodeficiency virus diagnoses in 2002-2003: capture-recapture methods." Am J Epidemiol **164**(4): 391-7.
Hall, H. I., R. Song, et al. (2008). "Estimation of HIV incidence in the United States." JAMA **300**(5): 520-9.
Hayes, R. J. and S. Bennett (1999). "Simple sample size calculation for cluster-randomized trials." Int J Epidemiol **28**(2): 319-26.
Higgins, S. T. and K. Silverman (1999). Motivating Behavior Change Among Illicit Drug Abusers: Research on Contingency Management Interventions. . American Psychological Association, Washington, DC.
Higgins, S. T., C. J. Wong, et al. (2000). "Contingent reinforcement increases cocaine abstinence during outpatient treatment and 1 year of follow-up." J Consult Clin Psychol **68**(1): 64-72.
Horberg, M. A., M. J. Silverberg, et al. (2008). "Effects of depression and selective serotonin reuptake inhibitor use on adherence to highly active antiretroviral therapy and on clinical outcomes in HIV-infected patients." J Acquir Immune Defic Syndr **47**(3): 384-90.
Hsieh, Y. H. and H. de Arazoza (2009). "Universal voluntary HIV testing and immediate antiretroviral therapy." Lancet **373**(9669): 1079-80; author reply 1080-1.
Jackevicius, C. A., M. Mamdani, et al. (2002). "Adherence with statin therapy in elderly patients with and without acute coronary syndromes." Jama **288**(4): 462-7.
Jara, M. M., K. M. Gallagher, et al. (2000). "Estimation of completeness of AIDS case reporting in Massachusetts." Epidemiology **11**(2): 209-13.
Jeffery, R. W., W. M. Gerber, et al. (1983). "Monetary contracts in weight control: effectiveness of group and individual contracts of varying size." J Consult Clin Psychol **51**(2): 242-8.
Jeffery, R. W., P. D. Thompson, et al. (1978). "Effects on weight reduction of strong monetary contracts for calorie restriction or weight loss." Behav Res Ther **16**(5): 363-9.

Johnson, B. T., M. P. Carey, et al. (2006). "Sexual risk reduction for persons living with HIV: research synthesis of randomized controlled trials, 1993 to 2004." J Acquir Immune Defic Syndr **41**(5): 642-50.

Jurgens, R., J. Cohen, et al. (2009). "Universal voluntary HIV testing and immediate antiretroviral therapy." Lancet **373**(9669): 1079; author reply 1080-1.

Justice, A. C. (2006). "Prioritizing primary care in HIV: comorbidity, toxicity, and demography." Top HIV Med **14**(5): 159-63.

Kane, R. L., P. E. Johnson, et al. (2004). "A structured review of the effect of economic incentives on consumers' preventive behavior." Am J Prev Med **27**(4): 327-52.

Kapetanovic, S., S. Christensen, et al. (2009). "Correlates of perinatal depression in HIV-infected women." AIDS Patient Care STDS **23**(2): 101-8.

Kitahata, M. M., S. J. Gange, et al. (2009). "Effect of early versus deferred antiretroviral therapy for HIV on survival." N Engl J Med **360**(18): 1815-26.

Klevens, R. M., P. L. Fleming, et al. (1998). "Completeness of HIV reporting in Louisiana, USA." Int J Epidemiol **27**(6): 1105.

Lazo, M., S. J. Gange, et al. (2007). "Patterns and predictors of changes in adherence to highly active antiretroviral therapy: longitudinal study of men and women." Clin Infect Dis **45**(10): 1377-85.

Levine, A. J., C. H. Hinkin, et al. (2005). "Variations in patterns of highly active antiretroviral therapy (HAART) adherence." AIDS Behav **9**(3): 355-62.

Lima, V. D., J. Geller, et al. (2007). "The effect of adherence on the association between depressive symptoms and mortality among HIV-infected individuals first initiating HAART." AIDS **21**(9): 1175-83.

Lussier, J. P., S. H. Heil, et al. (2006). "A meta-analysis of voucher-based reinforcement therapy for substance use disorders." Addiction **101**(2): 192-203.

Magnus, M., I. Kuo, et al. (2009). "Risk factors driving the emergence of a generalized heterosexual HIV epidemic in Washington, District of Columbia networks at risk." AIDS **23**(10): 1277-84.

Malotte, C. K., F. Rhodes, et al. (1998). "Tuberculosis screening and compliance with return for skin test reading among active drug users." Am J Public Health **88**(5): 792-6.

Marcus, A. C., C. P. Kaplan, et al. (1998). "Reducing loss-to-follow-up among women with abnormal Pap smears. Results from a randomized trial testing an intensive follow-up protocol and economic incentives." Med Care **36**(3): 397-410.

Markowitz, M., B. Y. Nguyen, et al. (2009). "Sustained Antiretroviral Effect of Raltegravir After 96 Weeks of Combination Therapy in Treatment-Naive Patients With HIV-1 Infection." J Acquir Immune Defic Syndr.

Marks, G., N. Crepaz, et al. (2006). "Estimating sexual transmission of HIV from persons aware and unaware that they are infected with the virus in the USA." AIDS **20**(10): 1447-50.

McNaghten, A. D., M. I. Wolfe, et al. (2007). "Improving the representativeness of behavioral and clinical surveillance for persons with HIV in the United States: the rationale for developing a population-based approach." PLoS One **2**(6): e550.

McQuillan, G. M., D. Kruszon-Moran, et al. (2006). "Prevalence of HIV in the US household population: the National Health and Nutrition Examination Surveys, 1988 to 2002." J Acquir Immune Defic Syndr **41**(5): 651-6.

Metsch, L. R., M. Pereyra, et al. (2004). "Delivery of HIV prevention counseling by physicians at HIV medical care settings in 4 US cities." Am J Public Health **94**(7): 1186-92.

Meyer, P. A., J. L. Jones, et al. (1994). "Completeness of reporting of diagnosed HIV-infected hospital inpatients." J Acquir Immune Defic Syndr **7**(10): 1067-73.

Mugavero, M., J. Ostermann, et al. (2006). "Barriers to antiretroviral adherence: the importance of depression, abuse, and other traumatic events." AIDS Patient Care STDS **20**(6): 418-28.

Mugavero, M. J., H. Y. Lin, et al. (2009). "Racial disparities in HIV virologic failure: do missed visits matter?" J Acquir Immune Defic Syndr **50**(1): 100-8.

Mugavero, M. J., H. Y. Lin, et al. (2007). "Failure to establish HIV care: characterizing the "no show" phenomenon." Clin Infect Dis **45**(1): 127-30.

Mugavero, M. J., H. Y. Lin, et al. (2009). "Missed visits and mortality among patients establishing initial outpatient HIV treatment." Clin Infect Dis **48**(2): 248-56.

NHBS-Het Survey (2009). NHBS-Het Survey, 2007-2008, District HIV Testing Data-the first 35,000 rapid tests.

Noar, S. M., H. G. Black, et al. (2009). "Efficacy of computer technology-based HIV prevention interventions: a meta-analysis." AIDS **23**(1): 107-15.

Norman, L. R., M. Basso, et al. (2009). "Neuropsychological consequences of HIV and substance abuse: a literature review and implications for treatment and future research." Curr Drug Abuse Rev **2**(2): 143-56.

Palella, F., C. Armon, et al. (2008). Enhanced Survival Associated with Use of HIV Susceptibility Testing among HAART-experienced Patients in the HIV Outpatient Study (HOPS) 15th Conference on Retroviruses and Opportunistic Infections.

Palella, F. J., Jr., K. M. Delaney, et al. (1998). "Declining morbidity and mortality among patients with advanced human immunodeficiency virus infection. HIV Outpatient Study Investigators." N Engl J Med **338**(13): 853-60.

Paltiel, A. D., M. C. Weinstein, et al. (2005). "Expanded screening for HIV in the United States--an analysis of cost-effectiveness." N Engl J Med **352**(6): 586-95.

Panel on Antiretroviral Guidelines for Adults and Adolescents. (2009). "Guidelines for the use of antiretroviral agents in HIV-1-infected adults and adolescents. Department of Health and Human Services. December 1, 2009." Retrieved December 21, 2009, from **http://www.aidsinfo.nih.gov/ContentFiles/AdultandAdolescentGL.pdf**.

Pence, B. W. (2009). "The impact of mental health and traumatic life experiences on antiretroviral treatment outcomes for people living with HIV/AIDS." J Antimicrob Chemother **63**(4): 636-40.

Phillips, A. N., C. Leen, et al. (2007). "Risk of extensive virological failure to the three original antiretroviral drug classes over long-term follow-up from the start of therapy in patients with HIV infection: an observational cohort study." Lancet **370**(9603): 1923-8.

Prevention, C. f. D. C. a. (2002). AIDS/HIV Surveillance Report. Atlanta, Centers for Disease Control and Prevention.

Quinn, T. C., M. J. Wawer, et al. (2000). "Viral load and heterosexual transmission of human immunodeficiency virus type 1. Rakai Project Study Group." N Engl J Med **342**(13): 921-9.

Rajabiun, S., R. K. Mallinson, et al. (2007). ""Getting me back on track": the role of outreach interventions in engaging and retaining people living with HIV/AIDS in medical care." AIDS Patient Care STDS **21 Suppl 1**: S20-9.

Reed, J. B., D. Hanson, et al. (2009). "HIV testing factors associated with delayed entry into HIV medical care among HIV-infected persons from eighteen states, United States, 2000-2004." AIDS Patient Care STDS **23**(9): 765-73.

Rigsby, M., M. Rosen, et al. (2000). "Cue-dose training with monetary reinforcement." Journal of General Internal Medicine **15**(12): 841-847.
Robison, L. S., A. O. Westfall, et al. (2008). "Short-term discontinuation of HAART regimens more common in vulnerable patient populations." AIDS Res Hum Retroviruses **24**(11): 1347-55.
Rosen, M., K. Dieckhaus, et al. (2007). "Improved adherence with contingency management." AIDS Patient Care and STDs **21**(1): 30-40.
Rosenblum, L., J. W. Buehler, et al. (1992). "The completeness of AIDS case reporting, 1988: a multisite collaborative surveillance project." Am J Public Health **82**(11): 1495-9.
Roux, P., M. P. Carrieri, et al. (2009). "Effect of anxiety symptoms on adherence to highly active antiretroviral therapy in HIV-infected women." J Clin Psychiatry **70**(9): 1328-9.
Royal, S. W., D. P. Kidder, et al. (2009). "Factors associated with adherence to highly active antiretroviral therapy in homeless or unstably housed adults living with HIV." AIDS Care **21**(4): 448-55.
Safren, S., R. Knauz, et al. (2006). "CBT for HIV medication adherence and depression: process and outcomes at post-treatment and three-month cross over." Ann Behav Med **31**: S006.
Sanders, G. D., A. M. Bayoumi, et al. (2005). "Cost-effectiveness of screening for HIV in the era of highly active antiretroviral therapy." N Engl J Med **352**(6): 570-85.
Schneider, E., S. Whitmore, et al. (2008). "Revised surveillance case definitions for HIV infection among adults, adolescents, and children aged <18 months and for HIV infection and AIDS among children aged 18 months to <13 years--United States, 2008." MMWR Recomm Rep **57**(RR-10): 1-12.
Schroeder, S. A. (2007). "Shattuck Lecture. We can do better--improving the health of the American people." N Engl J Med **357**(12): 1221-8.
Schwarcz, S. K., L. C. Hsu, et al. (1999). "The impact of the 1993 AIDS case definition on the completeness and timeliness of AIDS surveillance." AIDS **13**(9): 1109-14.
Seal, K. H., A. H. Kral, et al. (2003). "A randomized controlled trial of monetary incentives vs. outreach to enhance adherence to the hepatitis B vaccine series among injection drug users." Drug Alcohol Depend **71**(2): 127-31.
Sethi, A. K., D. D. Celentano, et al. (2003). "Association between adherence to antiretroviral therapy and human immunodeficiency virus drug resistance." Clin Infect Dis **37**(8): 1112-8.
Shapiro, M. F., S. C. Morton, et al. (1999). "Variations in the care of HIV-infected adults in the United States: results from the HIV Cost and Services Utilization Study." JAMA **281**(24): 2305-15.
Simoni, J. M., A. E. Kurth, et al. (2006). "Self-report measures of antiretroviral therapy adherence: A review with recommendations for HIV research and clinical management." AIDS Behav **10**(3): 227-45.
Simoni, J. M., C. R. Pearson, et al. (2006). "Efficacy of interventions in improving highly active antiretroviral therapy adherence and HIV-1 RNA viral load. A meta-analytic review of randomized controlled trials." J Acquir Immune Defic Syndr **43 Suppl 1**: S23-35.
Solomon, L., C. Flynn, et al. (1999). "Evaluation of a statewide non-name-based HIV surveillance system." J Acquir Immune Defic Syndr **22**(3): 272-9.
Steigbigel, R. T., D. A. Cooper, et al. (2008). "Raltegravir with optimized background therapy for resistant HIV-1 infection." N Engl J Med **359**(4): 339-54.

Sterne, J. A., M. May, et al. (2009). "Timing of initiation of antiretroviral therapy in AIDS-free HIV-1-infected patients: a collaborative analysis of 18 HIV cohort studies." Lancet **373**(9672): 1352-63.

Stevens-Simon, C., P. O'Connor, et al. (1994). "Incentives enhance postpartum compliance among adolescent prenatal patients." J Adolesc Health **15**(5): 396-9.

Sullivan, P., K. Kayitenkore, et al. (2009). Reduction of HIV Transmission Risk and High Risk Sex while Prescribed ART: Results from Discordant Couples in Rwanda and Zambia. 16th Conference on Retroviruses and Opportunistic Infections, Montreal, Canada.

Sutherland, K., J. B. Christianson, et al. (2008). "Impact of targeted financial incentives on personal health behavior: a review of the literature." Med Care Res Rev **65**(6 Suppl): 36S-78S.

Teshale, E., L. Kamimoto, et al. (2005). Estimated Number of HIV-infected Persons Eligible for and Receiving HIV Antiretroviral Therapy, 2003--United States 12th Conference on Retroviruses and Opportunistic Infections. Boston, MA.

Thomson, A., R. Hayes, et al. (2009). "Measures of between-cluster variability in cluster randomized trials with binary outcomes." Stat Med **28**(12): 1739-51.

Tobias, C. R., W. Cunningham, et al. (2007). "Living with HIV but without medical care: barriers to engagement." AIDS Patient Care STDS **21**(6): 426-34.

Torian, L. V., E. W. Wiewel, et al. (2008). "Risk factors for delayed initiation of medical care after diagnosis of human immunodeficiency virus." Arch Intern Med **168**(11): 1181-7.

Valdiserri, R. O., D. R. Holtgrave, et al. (1999). "Promoting early HIV diagnosis and entry into care." AIDS **13**(17): 2317-30.

Vlahov, D., A. M. Tang, et al. (2000). "Increased frequency of overdose mortality among HIV infected injection drug users." Additional Research **8**: 311-325.

Volpp, K. G., A. Gurmankin Levy, et al. (2006). "A randomized controlled trial of financial incentives for smoking cessation." Cancer Epidemiol Biomarkers Prev **15**(1): 12-8.

Volpp, K. G., L. K. John, et al. (2008). "Financial incentive-based approaches for weight loss: a randomized trial." JAMA **300**(22): 2631-7.

Volpp, K. G., G. Loewenstein, et al. (2008). "A test of financial incentives to improve warfarin adherence." BMC Health Serv Res **8**: 272.

Volpp, K. G., A. B. Troxel, et al. (2009). "A randomized, controlled trial of financial incentives for smoking cessation." N Engl J Med **360**(7): 699-709.

von Wyl, V., S. Yerly, et al. (2009). "Long-term trends of HIV type 1 drug resistance prevalence among antiretroviral treatment-experienced patients in Switzerland." Clin Infect Dis **48**(7): 979-87.

Wagner, B. G. and S. Blower. (2009). "Voluntary universal testing and treatment is unlikely to lead to HIV elimination: a modeling analysis." Nature Preceedings Posted 29 Oct 2009. Retrieved November 30, 2009, from http://precedings.nature.com/documents/3917/version/1.

Walensky, R. P., M. C. Weinstein, et al. (2005). "Optimal allocation of testing dollars: the example of HIV counseling, testing, and referral." Med Decis Making **25**(3): 321-9.

Washington, D. D. o. H. (2009). "Vital Stats." 2009, from www.doh.dc.gov\cppe\vital stats.

Weaver, M. R., C. J. Conover, et al. (2009). "Cost-effectiveness analysis of integrated care for people with HIV, chronic mental illness and substance abuse disorders." J Ment Health Policy Econ **12**(1): 33-46.

Weber, R., L. Christen, et al. (2004). "Effect of individual cognitive behaviour intervention on adherence to antiretroviral therapy: prospective randomized trial." Antivir Ther **9**(1): 85-95.

Weintrob, A. C., G. A. Grandits, et al. (2009). "Virologic Response Differences Between African Americans and European Americans Initiating Highly Active Antiretroviral Therapy With Equal Access to Care." J Acquir Immune Defic Syndr.

When to Start Consortium (2009). "Timing of initiation of antiretroviral therapy in AIDS-free HIV-1-infected patients: a collaborative analysis of 18 HIV cohort studies." Lancet **373**(9672): 1352-63.

Wilkin, T. J. and R. M. Gulick (2008). "When to start antiretroviral therapy?" Clin Infect Dis **47**(12): 1580-6.

Wood, E., T. Kerr, et al. (2008). "Poor adherence to HIV monitoring and treatment guidelines for HIV-infected injection drug users." HIV Med **9**(7): 503-7.

Wyatt, G. E., D. Longshore, et al. (2004). "The efficacy of an integrated risk reduction intervention for HIV-positive women with child sexual abuse histories." AIDS Behav **8**(4): 453-62.

Zetola, N. M., K. Bernstein, et al. (2009). "Using surveillance data to monitor entry into care of newly diagnosed HIV-infected persons: San Francisco, 2006-2007." BMC Public Health **9**: 17.

APPENDICES

Appendix I: Schedule of Study Visits and Procedures

Schedule of Study Visits and Procedures
Prevention for Positives Computerized Patient Intervention and Survey

	HIV Care Site<br>Visit #1<br>Enrollment	HIV Care Site<br>Visit #2<br>(Month 3)	HIV Care Site<br>Visit #3- Visit #6<br>(Months 6, 9, 12; & 18)
**Administrative and Regulatory Procedures**			
Pre-screening	X		
Administer Informed Consent	X		
Confirm Eligibility	X		
Collect Locator Information	X		
Administer CARE+/ACASI	X	X	X
Administer Survey	X		X (Month 12 only)
Provide Compensation	X	X	X

Appendix IIA: Patient Computer-Delivered Intervention and Survey Informed Consent Form

TITLE OF THE RESEARCH:

HPTN 065- TLC-Plus: A Study to Evaluate the Feasibility of an Enhanced Test, Link-to-Care, Plus Treat Approach for HIV Prevention in the United States

HPTN 065, Version 1.0 01 March 2010

DAIDS ID: 11685

SPONSOR: National Institute of Allergy and Infectious Diseases (NIAID), National Institute on Drug Abuse (NIDA), National Institute of Mental Health (NIMH), National Institutes of Health (NIH)

PATIENT COMPUTER-DELIVERED INTERVENTION AND SURVEY INFORMED CONSENT
INVESTIGATOR OF RECORD: (*insert name*)
PHONE: (*insert number*)

Introduction

You have been asked to take part in a study that is testing a new computer program to help HIV-positive people. The research that we invite you to participate in will use a computer to privately ask you questions and give you feedback. You will be asked to use the computer a total of 6 times. You will use the computer every three months when you come in for your clinic visits for twelve months and again at 18 months.

Around 1320 people who are HIV-positive in Washington, D.C. and in the Bronx will participate in the study. You do not have to know how to use a computer or be able to read to be in this study.

What will happen during this study?

We will use a computer to talk about what's going on for you with HIV. During your first and 12-month visit, we will also ask you questions about your knowledge of HIV, your knowledge of medications for treating HIV and your feelings about medical care for people with HIV.

We hope that you will feel comfortable answering all of the questions openly and honestly, but you may refuse to answer any of the questions or stop at any time. You will also be provided with contact and referral information if any of the questions raises issues that you would like to talk about further, at this or some later time.

We anticipate that the computer sessions will take you approximately XXX minutes to complete. For your time and effort, we will reimburse you $XXXX. There is no cost to you to participate in this part of study.

People who choose to join the study will be assigned to a study group. There are only two groups:

- One study group will be asked questions by the computer program.

- The second study group will be asked the same questions and will also be shown some videos. The videos will be short and will include HIV risk reduction topics. After the videos are shown, the computer then will help people create a health plan.

You will have a 50% chance of being in the group that is asked questions by the computer program. You will also have a 50% chance of being in the group that is asked questions and shown videos. The group assignment will be made randomly by the computer program. Staff at the site where you get your HIV care cannot assign you to a group and the staff will not know which group you are assigned to.

Computer sessions for both study groups are anonymous. No names or identifying information will be attached to the computer. You will get an anonymous printout at the end of your session, which you can choose to share with your provider, if you want. You do not have to show your provider the printout.

For both study groups, we would like permission to access medical records at the HIV clinic. We will use this information to evaluate HIV Viral Load, CD4 cell count, and other information relevant to your health, at each computer session. We would only like to access your information for as long as you are enrolled in the study. Once you have completed the study we will no longer access your medical records at any HIV clinic. We will not share information with the clinic staff about your answers to the questions on the computer. If you decide to allow us to access your health information for the study, you will need to sign a form at your doctor's office giving them permission to let us see your records.

For the study group that is asked questions and shown videos, there are questions about depression, suicide and domestic violence. If you are assigned to this group and if your answers to these questions show that you may be depressed, suicidal or are currently in an abusive relationship, a health worker here at the clinic will follow-up with you but will not know what you may be having trouble with. For example, the healthcare worker will not know whether you indicated that you are suicidal or whether you indicated that you are in an abusive relationship. We will not share your actual answers with staff at the clinic. You can decide what information you want to share with the healthcare worker.

What are the potential benefits?

There may be no direct benefits to you. We hope the information we collect will help us find better ways to provide HIV care in your community. You may feel a benefit from sharing your experiences with someone who is interested in your opinions.

What are the possible risks or discomforts?

It is possible that answering the questions on the computer may make you embarrassed or upset. You may refuse to answer any of the questions or stop answering at any time. The greatest risk may involve your privacy. The steps that the study team has taken to protect your privacy are described below.

How will your privacy be protected?

We cannot guarantee absolute confidentiality. However, we will do everything possible to protect your confidentiality if you join this study.

To protect your privacy, you will meet with a healthcare provider in a private area where others cannot overhear conversations with you. While you are participating in the computerized session, you will be given headphones and a place to sit where no one can look over your shoulder to see what you are doing.

Every effort will be made to keep your personal information confidential. Your personal information (name, address, phone number) will be protected by the research clinic. This information will not be used in any publication of information about this study.

In addition to the efforts made by the study staff to keep your personal information confidential, we have obtained a Certificate of Confidentiality from the United States federal government. This certificate protects researchers from being forced to tell people who are not connected with this study, such as the court system, about your participation. Any publication that results from this study will not use your name or identify you personally.

People who may review your records include ***(insert name of site)*** IRB, National Institutes of Health (NIH), government or regulatory agencies, study staff, and the study monitors. Also, the Certificate of Confidentiality does not prevent you from releasing information about yourself and your participation in the study. The certificate cannot keep the United States government from auditing or evaluating federally funded projects such as this one. Even with the Certificate of Confidentiality, if the study staff learns of possible child abuse and/or neglect or a risk of harm to you or others, we will tell the proper authorities.

Your participation is voluntary.

You are not required to join this study. You do not have to participate in the computer sessions for us. If you decide to participate, you may refuse to answer any of the questions or stop at any time without reducing or affecting any care that you receive at this site. If you do decide to leave the study we will ask you to complete one final computer session, however, you will not be required to do this.

What are some reasons why you may be withdrawn from this activity without your consent?

You may be withdrawn from the study without your consent for the following reasons:

- The research study, or this part of the study, is stopped or canceled
- The study staff feels that completing the study or this part of the study would be harmful to you or others

What happens if you are injured by this activity?

Because this activity only involves answering questions, reading messages and viewing videos, it is very unlikely that you could be injured. However, if you are injured as a result of joining this study, you will be given immediate treatment for your injuries. You may have to pay for this care. There is no program for compensation either through this institution or the United States NIH. You will not be giving up any of your legal rights by signing this consent form.

SIGNATURE PAGE

Persons to Contact for Problems or Questions
For questions about this study or a research-related injury, contact:

- ***(site insert name of the investigator or other study staff)***
- ***(site insert telephone number and physical address of above)***

For questions about your rights as a research participant, contact:

- ***(site insert name or title of person on the Institutional Review Board (IRB) or other organization appropriate for the site)***
- ***(site insert telephone number and physical address of above)***

If you have either read or have heard the information in this consent form, if all of your questions have been answered, and if you agree to take part in the computer assisted interview and patient survey questionnaire, please sign your name on the line below.

______________________	__________________________________
Patient's Name (print)	Patient's Signature and Date
______________________	__________________________________
Study Staff Conducting Consent Discussion (print)	Study Staff Signature and Date
______________________	__________________________________
Witness' Name (print) (As appropriate)	Witness' Signature and Date

Appendix IIB: Provider Survey Online Informed Consent Text

The NIH-funded HIV Prevention Trials Network (HPTN) is conducting a study to assess the feasibility of increasing HIV testing and facilitating linkage to care for HIV-infected-positive patients with the eventual goal of reducing incident HIV infection in the U.S. For the study to successfully impact HIV testing and treatment, the study team first needs to understand current practices of front-line providers.

You are invited to participate in a brief survey. This usually takes about *_XXX_ minutes to complete. You were selected because of your experience and geographic location. The questionnaire includes questions about your knowledge of HIV, your knowledge of medications for treating HIV, and your feelings/opinions about medical care for people with HIV. No personally identifying information will be collected about you, only some basic demographics. We hope that you will feel comfortable answering all of the questions openly and honestly, but you may refuse to answer any of the questions or stop completing the questionnaire, at any time. For your time and effort in completing this survey, we are providing you with a $50 Amazon.com gift certificate. You may access the certificate by printing the web coupon attached to this survey.

If you have read the information in this consent form, and if you agree to take part in the questionnaire, please CLICK on the link below. This will constitute your informed consent to participate in this survey.

I CONSENT

Appendix IIIA: Adult HIV/AIDS Confidential Case Report

(Currently under revision, Office of Management and Budget expiration February 2010)

I. STATE/LOCAL USE ONLY

Patient's Name: ______ Phone No.: ( ) ______
(Last, First, M.I.)

Address: ______ City: ______ County: ______ State: ______ Zip Code: ______

RETURN TO STATE/LOCAL HEALTH DEPARTMENT

– Patient identifier information is not transmitted to CDC! –

U.S. DEPARTMENT OF HEALTH & HUMAN SERVICES
Centers for Disease Control and Prevention

ADULT HIV/AIDS CONFIDENTIAL CASE REPORT

(Patients ≥13 years of age at time of diagnosis)

CDC
CENTERS FOR DISEASE CONTROL AND PREVENTION

II. HEALTH DEPARTMENT USE ONLY

Form Approved OMB No. 0920-0573 Exp Date 2/28/2010

DATE FORM COMPLETED: Mo. Day Yr.

REPORT SOURCE: ☐☐

SOUNDEX CODE: ☐☐☐☐

REPORT STATUS: 1 New Report 2 Update

REPORTING HEALTH DEPARTMENT:
State: ______
City/County: ______

State Patient No.: ☐☐☐☐☐☐☐☐☐☐

City/County Patient No.: ☐☐☐☐☐☐☐☐☐☐

III. DEMOGRAPHIC INFORMATION

DIAGNOSTIC STATUS AT REPORT (check one):
1 HIV Infection (not AIDS)
2 AIDS

AGE AT DIAGNOSIS: ☐☐ Years / ☐☐ Years

DATE OF BIRTH: Mo. Day Yr.

CURRENT STATUS: Alive 1 Dead 2 Unk. 9

DATE OF DEATH: Mo. Day Yr.

STATE/TERRITORY OF DEATH: ______

SEX: 1 Male 2 Female

ETHNICITY: (select one) 1 Hispanic 9 Unk 2 Not Hispanic or Latino

RACE: (select one or more) ☐ American Indian/Alaska Native ☐ Black or African American ☐ Asian ☐ Native Hawaiian or Other Pacific Islander ☐ White ☐ Unk

COUNTRY OF BIRTH: 1 U.S. 7 U.S. Dependencies and Possessions (including Puerto Rico) (specify): ______ 8 Other (specify): ______ 9 Unk

RESIDENCE AT DIAGNOSIS:
City: ______ County: ______ State/Country: ______ Zip Code: ☐☐☐☐☐☐☐☐☐

IV. FACILITY OF DIAGNOSIS

Facility Name ______

City ______

State/Country ______

FACILITY SETTING (check one)
1 Public 2 Private 3 Federal 9 Unk.

FACILITY TYPE (check one)
01 Physician, HMO 31 Hospital, Inpatient
88 Other (specify): ______

This report to the Centers for Disease Control and Prevention (CDC) is authorized by law (Sections 304 and 306 of the Public Health Service Act, 42 USC 242b and 242k). Response in this case is voluntary for federal government purposes, but may be mandatory under state and local statutes. Your cooperation is necessary for the understanding and control of HIV/AIDS. Information in CDC's HIV/AIDS surveillance system that would permit identification of any individual on whom a record is maintained, is collected with a guarantee that it will be held in confidence, will be used only for the purposes stated in the assurance on file at the local health department, and will not otherwise be disclosed or released without the consent of the individual in accordance with Section 308(d) of the Public Health Service Act (42 USC 242m).

V. PATIENT HISTORY

AFTER 1977 AND PRECEDING THE FIRST POSITIVE HIV ANTIBODY TEST OR AIDS DIAGNOSIS, THIS PATIENT HAD (Respond to **ALL** Categories):

	Yes	No	Unk.
• Sex with male	1	0	9
• Sex with female	1	0	9
• Injected nonprescription drugs	1	0	9
• Received clotting factor for hemophilia/coagulation disorder	1	0	9

Specify disorder: 1 Factor VIII (Hemophilia A) 2 Factor IX (Hemophilia B) 8 Other (specify): ______

• *HETEROSEXUAL* relations with any of the following:

	Yes	No	Unk.
• Intravenous/injection drug user	1	0	9
• Bisexual male	1	0	9
• Person with hemophilia/coagulation disorder	1	0	9
• Transfusion recipient with documented HIV infection	1	0	9
• Transplant recipient with documented HIV infection	1	0	9
• Person with AIDS or documented HIV infection, risk not specified	1	0	9
• Received transfusion of blood/blood components (other than clotting factor)	1	0	9

First Mo. Yr. ☐☐ ☐☐ Last Mo. Yr. ☐☐ ☐☐

	Yes	No	Unk.
• Received transplant of tissue/organs or artificial insemination	1	0	9
• Worked in a health-care or clinical laboratory setting	1	0	9

(specify occupation): ______

VI. LABORATORY DATA

1. HIV ANTIBODY TESTS AT DIAGNOSIS: (Indicate first test)

	Pos	Neg	Ind	Not Done	TEST DATE Mo.	Yr.
• HIV–1 EIA	1	0	–	9		
• HIV–1/HIV–2 combination EIA	1	0	–	9		
• HIV–1 Western blot/IFA	1	0	8	9		
• Other HIV antibody test (specify): ______	1	0	8	9		

• Date of last documented negative HIV test Mo. Yr.
(specify type): ______

• If HIV laboratory tests were not documented, is HIV diagnosis documented by a physician? Yes 1 No 0 Unk. 9

If yes, provide date of documentation by physician Mo. Yr.

2. POSITIVE HIV DETECTION TEST: (Record earliest test) Mo. Yr.
☐ culture ☐ antigen ☐ PCR, DNA or RNA probe
• Other (specify): ______

3. DETECTABLE VIRAL LOAD TEST: (Record most recent test)
Test type* ☐☐ COPIES/ML ☐,☐☐☐,☐☐☐ Mo. Yr.

*Type: 11. NASBA (Organon) 12. RT-PCR (Roche) 13. bDNA(Chiron) 18. Other

4. IMMUNOLOGIC LAB TESTS:

AT OR CLOSEST TO CURRENT DIAGNOSTIC STATUS Mo. Yr.
• CD4 Count ☐,☐☐☐ cells/μL
• CD4 Percent ☐☐ %

First <200 μL or <14% Mo. Yr.
• CD4 Count ☐,☐☐☐ cells/μL
• CD4 Percent ☐☐ %

CDC 50.42A REV. 03/2007 (Page 1 of 2) – ADULT HIV/AIDS CONFIDENTIAL CASE REPORT –

VII. STATE/LOCAL USE ONLY

Physician's Name: ______________________ Phone No.: ( ) __________ Medical Record No. __________
(Last, First, M.I.)
Hospital/Facility: ______________________ Person Completing Form: __________ Phone No.: ( ) __________

– Patient identifier information is not transmitted to CDC! –

VIII. CLINICAL STATUS

CLINICAL RECORD REVIEWED: Yes 1 No 0

ENTER DATE PATIENT WAS DIAGNOSED AS: Asymptomatic (including acute retroviral syndrome and persistent generalized lymphadenopathy): Mo. Yr. Symptomatic (not AIDS): Mo. Yr.

AIDS INDICATOR DISEASES	Initial Diagnosis Def.	Pres.	Initial Date Mo. Yr.	AIDS INDICATOR DISEASES	Initial Diagnosis Def.	Pres.	Initial Date Mo. Yr.
Candidiasis, bronchi, trachea, or lungs	1	NA		Lymphoma, Burkitt's (or equivalent term)	1	NA	
Candidiasis, esophageal	1	2		Lymphoma, immunoblastic (or equivalent term)	1	NA	
Carcinoma, invasive cervical	1	NA		Lymphoma, primary in brain	1	NA	
Coccidioidomycosis, disseminated or extrapulmonary	1	NA		*Mycobacterium avium* complex or *M.kansasii*, disseminated or extrapulmonary	1	2	
Cryptococcosis, extrapulmonary	1	NA		*M. tuberculosis*, pulmonary*	1	2	
Cryptosporidiosis, chronic intestinal (>1 mo. duration)	1	NA		*M. tuberculosis*, disseminated or extrapulmonary*	1	2	
Cytomegalovirus disease (other than in liver, spleen, or nodes)	1	NA		*Mycobacterium*, of other species or unidentified species, disseminated or extrapulmonary	1	2	
Cytomegalovirus retinitis (with loss of vision)	1	2		*Pneumocystis carinii* pneumonia	1	2	
HIV encephalopathy	1	NA		Pneumonia, recurrent, in 12 mo. period	1	2	
Herpes simplex: chronic ulcer(s) (>1 mo. duration); or bronchitis, pneumonitis or esophagitis	1	NA		Progressive multifocal leukoencephalopathy	1	NA	
Histoplasmosis, disseminated or extrapulmonary	1	NA		Salmonella septicemia, recurrent	1	NA	
Isosporiasis, chronic intestinal (>1 mo. duration)	1	NA		Toxoplasmosis of brain	1	2	
Kaposi's sarcoma	1	2		Wasting syndrome due to HIV	1	NA	

Def. = definitive diagnosis Pres. = presumptive diagnosis

* RVCT CASE NO.:

- **If HIV tests were not positive or were not done, does this patient have an immunodeficiency that would disqualify him/her from the AIDS case definition?** 1 Yes 0 No 9 Unknown

IX. TREATMENT/SERVICES REFERRALS

Has this patient been informed of his/her HIV infection? 1 Yes 0 No 9 Unk.

This patient's partners will be notified about their HIV exposure and counseled by:
1 Health department 2 Physician/provider 3 Patient 9 Unknown

This patient is receiving or has been referred for:

	Yes	No	NA	Unk.
• HIV related medical services	1	0	–	9
• Substance abuse treatment services	1	0	8	9

This patient received or is receiving:

	Yes	No	Unk.
• Anti-retroviral therapy	1	0	9
• PCP prophylaxis	1	0	9

This patient has been enrolled at:

Clinical Trial	Clinic
1 NIH-sponsored	1 HRSA-sponsored
2 Other	2 Other
3 None	3 None
9 Unknown	9 Unknown

This patient's medical treatment is <u>primarily</u> reimbursed by:
1 Medicaid 2 Private insurance/HMO
3 No coverage 4 Other Public Funding
7 Clinical trial/government program 9 Unknown

FOR WOMEN:
- This patient is receiving or has been referred for gynecological or obstetrical services: 1 Yes 0 No 9 Unknown
- Is this patient currently pregnant? 1 Yes 0 No 9 Unknown
- Has this patient delivered live-born infants? 1 Yes (if delivered after 1977, provide birth information below for the most recent birth) 0 No 9 Unknown

CHILD'S DATE OF BIRTH: Mo. Day Yr.

Hospital of Birth: __________
City: __________ State: __________

Child's Soundex:

Child's State Patient No.

X. COMMENTS: __________

Public reporting burden of this collection of information is estimated to average 20 minutes per response, including the time for reviewing instructions, searching existing data sources, gathering and maintaining the data needed, and completing and reviewing the collection of information. An agency may not conduct or sponsor, and a person is not required to respond to a collection of information unless it displays a currently valid OMB control number. Send comments regarding this burden estimate or any other aspect of this collection of information, including suggestions for reducing this burden to CDC, Project Clearance Officer, 1600 Clifton Road, MS D-74, Atlanta, GA 30333, ATTN: PRA (0920-0573). **Do not send the completed form to this address.**

CDC 50.42A REV. 03/2007 (Page 2 of 2) – ADULT HIV/AIDS CONFIDENTIAL CASE REPORT –

Appendix IIIB: Behavioral Risk Factor Surveillance System (BRFSS)

(Section 20: HIV/AIDS)

The next few questions are about the national health problem of HIV, the virus that causes AIDS. Please remember that your answers are strictly confidential and that you don't have to answer every question if you do not want to. Although we will ask you about testing, we will not ask you about the results of any test you may have had.

20.1 Have you ever been tested for HIV? Do not count tests you may have had as part of a blood donation. Include testing fluid from your mouth.

1	Yes		7	Don't know / Not sure	**[Go to Q20.5]**
2	No	**[Go to Q20.5]**	9	Refused	**[Go to Q20.5]**

20.2 Not including blood donations, in what month and year was your last HIV test?

NOTE: If response is before January 1985, code "Don't know."

CATI INSTRUCTION: If the respondent remembers the year but cannot remember the month, code the first two digits 77 and the last four digits for the year.

_ _ /_ _ _ _	Code month and year
7 7/ 7 7 7 7	Don't know / Not sure
9 9/ 9 9 9 9	Refused

20.3 Where did you have your last HIV test — at a private doctor or HMO office, at a counseling and testing site, at a hospital, at a clinic, in a jail or prison, at a drug treatment facility, at home, or somewhere else?

0 1	Private doctor or HMO office
0 2	Counseling and testing site
0 3	Hospital
0 4	Clinic
0 5	Jail or prison (or other correctional facility)
0 6	Drug treatment facility
0 7	At home
0 8	Somewhere else
7 7	Don't know / Not sure
9 9	Refused

CATI note: Ask Q20.4; if Q20.2 = within last 12 months. Otherwise, go to Q20.5.

20.4 Was it a rapid test where you could get your results within a couple of hours?

1	Yes	7	Don't know / Not sure
2	No	9	Refused

20.5 I'm going to read you a list. When I'm done, please tell me if any of the situations apply to you. You do not need to tell me which one.

- You have used intravenous drugs in the past year.
- You have been treated for a sexually transmitted or venereal disease in the past year.
- You have given or received money or drugs in exchange for sex in the past year.
- You had anal sex without a condom in the past year.

Do any of these situations apply to you?

1	Yes	7	Don't know / Not sure
2	No	9	Refused

Appendix IIIC: 2008 PEMS Variable Requirements

PEMS Required Variables
Quick Reference Guide
February 8, 2008

This document provides a summary of the variable requirements for the January 1 and July 1, 2008 data collection periods, excluding variable requirements for HIV Testing and Partner Counseling and Referral Services (PCRS). HIV Testing variable requirements are currently specified in the HIV Testing Form and Variables Manual and the CDC HIV Testing Variables Data Dictionary. Requirements for PCRS will be released later in 2008. Since this document only provides a summary of the requirements, please refer to the PEMS DVS for a more detailed description of definitions and value choices.

Variable Number	Variable Name	HD & CBO Reported Required
	**General Agency Information (Table A)**	
A01	Agency Name	Required
A01a	PEMS Agency ID	Required
A02	Community Plan Jurisdiction	Required
A03	Employer Identification Number (EIN)	Required
A04	Street Address 1	Required
A06	City	Required
A08	State	Required
A09	Zip Code	Required
A10	Agency Website	Required
A11	Agency DUNS Number	Required
A12	Agency Type	Required
A13	Faith-based	Required
A14	Race/Ethnicity Minority Focused	Required
A18	Directly Funded Agency	Required
A21	Agency Contact Last Name	Required
A22	Agency Contact First Name	Required
A23	Agency Contact Title	Required
A24	Agency Contact Phone	Required
A25	Agency Contact Fax	Required
A26	Agency Contact Email	Required
	**CDC Program Announcement Award Information (Table B)**	
B01	CDC HIV Prevention PA Number	Required
B02	CDC HIV Prevention PA Budget Start Date	Required
B03	CDC HIV Prevention PA Budget End Date	Required
B04	CDC HIV Prevention PA Award Number	Required
B06	Total CDC HIV Prevention Award Amount	Required
B06a	Annual CDC HIV Prevention Award Amount Expended	Required
B07	Amount Allocated for Community Planning	Required
B08	Amount Allocated for Prevention Services	Required
B09	Amount Allocated for Evaluation	Required
B10	Amount Allocated for Capacity Building	Required

PEMS Required Variables
Quick Reference Guide
February 8, 2008

Variable Number	Variable Name	HD & CBO Reported Required
	**Contractor Information (Table C)**	
C01	Agency Name	Required
C04	City	Required
C06	State	Required
C07	Zip Code	Required
C13	Employer Identification Number (EIN)	Required
C14	DUNS Number	Required
C15	Agency Type	Required
C16	Agency Activities	Required
C17	Faith-based	Required
C18	Race/Ethnicity Minority Focused	Required
C19	Contract Start Date-Month	Required
C20	Contract Start Date-Year	Required
C21	Contract End Date- Month	Required
C22	Contract End Date- Year	Required
C23	Total Contract Amount Awarded	Required
C25	CDC HIV Prevention Program Announcement Number	Required
C26	CDC HIV Prevention PA Budget Start Date	Required
C27	CDC HIV Prevention PA Budget End Date	Required
	**Site Information (Table S)**	
S01	Site ID	Required
S03	Site Name	Required
S04	Site Type	Required
S08	County	Required
S09	State	Required
S10	Zip Code	Required
S16	Use of Mobile Unit	Required
	**Program Name - Planning (Table D)**	
D01	Program Name	Required
D02	Community Planning Jurisdiction	Required
D03	Community Planning Year	Required
	**Program Model and Budget - Planning (Table E1)**	
E101	Program Model Name	Required
E102	Evidence Base	Required
E103	CDC Recommended Guidelines	Required
E104	Other Basis for Program Model	Required
E104-1	Specify Other Basis for Program Model	Required
E105	Target Population	Required
E107	Program Model Start Date	Required
E108	Program Model End Date	Required

Version 2.0
Feb 2008

Required Variables for Data Collection 2008
Page 2

PEMS Required Variables
Quick Reference Guide
February 8, 2008

Variable Number	Variable Name	HD & CBO Reported Required
E109	Proposed Annual Budget	Required
	**Intervention Plan Characteristics (Table F)**	
F01	Intervention Type	Required
F02	Intervention ID	Required
F02a	Intervention Name	Required
F03	HIV+ Intervention	Required
F04	Perinatal Intervention	Required
F05	Total Number of Clients	Required
F06	Sub-Total Target Population	Required
F07	Planned Number of Cycles	Required
F08	Number of Sessions	Required
F09	Unit of Delivery	Required
F11	Delivery Method	Required
F14	Level of Data Collection	Required
	**Client Characteristics (Table G)**	
G101	Date Collected	Required
G102	PEMS Client Unique Key	Required
G112	Date of Birth - Year	Required
G113	Calculated Age (System Generated)	Required
G114	Ethnicity	Required
G116	Race	Required
G120	State/Territory of Residence	Required
G123	Assigned Sex at Birth	Required
G124	Current Gender	Required
G200	Date Collected	Required
G204	Previous HIV Test	Required
G205	Self Reported HIV Test Result	Required
G208	In HIV Medical Care/Treatment (only if HIV+)	Required
G209	Pregnant (only if female)	Required
G210	In Prenatal Care (only if pregnant)	Required
G211	Client Risk Factors ***	Required
G212	Additional Client Risk Factors ^^^	Required
G213	Recent STD (Not HIV)	Required

***Note: The recall period for client risk factors is 12 months.
^^^Note: Additional value choices for risk factors added:
Sex without using a condom
Sharing drug injection equipment

PEMS Required Variables
Quick Reference Guide
February 8, 2008

Variable Number	Variable Name	HD & CBO Reported Required
**Client Intervention Charactertics (Table H)**		
H01	Intervention ID	Required
H01a	Intervention Name	Required
H03	Cyce	Required
H04a	Form ID (Counseling & Testing Only)	Required
H05	Session Number	Required
H06	Session Date	Required
H10	Site Name/ID	Required
H13	Recruitment Source	Required
H18	Recruitment Source - Service/Intervention Type	Required
H21	Incentive Provided	Required
H22	Unit of Delivery	Required
H23	Delivery Method	Required
**Referral (Table X7)**		
X702	Referral Date	Required
X702a	Reason Client Not Refrerred to Medical Care	Required
X703	Referral Service Type	Required
X706	Referral Outcome	Required
X710	Referral Close Date	Required
X712	HIV Test Performed	Required
X713	HIV Test Result	Required
X714	Confirmatory Test	Required
X714a	HIV Test Result Provided	Required

PEMS Required Variables
Quick Reference Guide
February 8, 2008

Variable Number	Variable Name	HD & CBO Reported Required
	**Aggregate HE/RR and Outreach (Table AG)**	
AG00	Intervention Name/ID	Required
AG01	Session Number	Required
AG02	Date of Event/Session	Required
AG03	Duration of Event/Session	Required
AG04	Number of Client Contacts	Required
AG05a	Delivery Method	Required
AG05c	Incentive Provided	Required
AG06	Site Name/ID	Required
AG08a	Client Primary Risk - MSM	Required
AG08b	Client Primary Risk - IDU	Required
AG08c	Client Primary Risk - MSM/IDU	Required
AG08d	Client Primary Risk - Sex Involving Transgender	Required
AG08e	Client Primary Risk - Heterosexual Contact	Required
AG08f	Client Primary Risk - Other/Risk Not Identified	Required
AG09a	Client Gender - Male	Required
AG09b	Client Gender - Female	Required
AG09c	Client Gender - Transgender MTF	Required
AG09d	Client Gender - Transgender FTM	Required
AG10a	Client Ethnicity - Hispanic or Latino	Required
AG10b	Client Ethnicity - Not Hispanic or Latino	Required
AG11a	Client Race - American Indian or Alaska Native	Required
AG11b	Client Race - Asian	Required
AG11c	Client Race - Black or African American	Required
AG11d	Client Race - Native Hawaiian or Other Pacific Islander	Required
AG11e	Client Race - White	Required
AG11f	Client Race - Multiracial	Required
AG12a	Client Age - Under 13 years	Required
AG12b	Client Age - 13 - 18 years	Required
AG12c	Client Age - 19-24 years	Required
AG12d	Client Age - 25 - 34 years	Required
AG12e	Client Age - 35 - 44 years	Required
AG12f	Client Age - 45 years and over	Required
AG14a	Materials Distributed - Male Condoms	Required
AG14b	Materials Distributed - Female Condoms	Required
AG14c	Materials Distributed - Bleach or Safer Injection Kits	Required
AG14d	Materials Distributed - Education Materials	Required
AG14e	Materials Distributed - Safe Sex Kits	Required
AG14f	Materials Distributed - Referral list	Required
AG14g	Materials Distributed - Role Model Stories	Required
AG14h	Materials Distributed - Other (specify)	Required
AG15	Aggregate Data Collection Method	Required

PEMS Required Variables
Quick Reference Guide
February 8, 2008

Variable Number	Variable Name	HD & CBO Reported Required
**Health Communication / Public Information (Table HC)**		
HC01	Intervention Name/ID	Required
HC02	HC/PI Delivery Method	Required
HC05	Event Start Date	Required
HC06	Event End Date	Required
HC07	Total Number of Airings	Required
HC08	Estimated total Exposures	Required
HC09	Number of Materials Distributed	Required
HC10	Total Number of Web Hits	Required
HC11	Total Number of Attendees	Required
HC12	Number of Callers	Required
HC13	Number of Callers Referred	Required
HC14	Distribution - Male condoms	Required
HC15	Distribution - Female condoms	Required
HC16	Distribution - Lubricants	Required
HC17	Distribution - Bleach or Safer Injection Kits	Required
HC18	Distribution - Referral Lists	Required
HC19	Distribution - Safe sex kits	Required
HC20	Distribution - Other	Required
HC21	Site Name/ID	Required
**Community Planning Level (Table CP-A/B/C)**		
CP-A01	Name of HIV Prevention CPG	HD only
CP-A02	Community Plan Year	HD only
CP-B01	Priority Population	HD only
CP-B02	Rank	HD only
CP-B03	Age	HD only
CP-B04	Gender	HD only
CP-B05	Ethnicity	HD only
CP-B06	Race	HD only
CP-B07	HIV Status	HD only
CP-B08	Geo Location	HD only
CP-B09	Transmission Risk	HD only
CP-C01	Name of the Prevention Activity/Intervention	HD only
CP-C02	Prevention Activity/Intervention Type	HD only
CP-C04	Evidence Based	HD only
CP-C05	CDC Recommended Guidelines	HD only
CP-C06	Other Basis for Intervention	HD only
CP-C07	Activity	HD only

Appendix IIID: NYC HIV Surveillance Performance Indicators

New York State requires named reporting of all diagnoses of HIV and AIDS, all HIV-related illness, all positive Western Blot (WB) tests for HIV antibody, all VL and CD4 lymphocyte values, and all HIV genotypes. The NYC HIV/AIDS Reporting System (HARS) is a population-based registry that since 1981 has been continuously updated with new, de-duplicated diagnoses and laboratory results. All incoming provider and laboratory reports that do not match an existing registry record initiate a field investigation with medical record review to confirm the case, date and disposition of diagnosis and collect all other data required for surveillance and partner notification. HARS also obtains data through regular matches with other disease registries, the NYC Death Registry, the National Death Index and the Social Security Death Master File. Because of its comprehensive nature, long history (AIDS reporting since 1981, HIV reporting since 2000) and location (AIDS epicenter, largest in the west), the system can reasonably be characterized as the largest longitudinal community HIV/AIDS database in the world. It is therefore an ideal source of population-level outcome data for communities mounting interventions to improve early diagnosis and uptake of care.

Because of the size, history, continuous feed of new information (>35,000 new laboratory results received per month), and reliance on outside entities (laboratories, medical records) of the surveillance system, control of its quality, completeness, accuracy and timeliness is an ongoing challenge. The program follows a set of monthly performance indicators to track data quality and staff and provider performance, and meets on a quarterly basis to review indicators, identify problem areas and take steps toward corrective action. The following points summarize the volume and periodicity of electronic laboratory reporting, the volume, timeliness and outcome of field investigations, the periodic registry matches, internal matching and deduplication procedures, and the interstate deduplication activities of the program.

NYC HIV Core Surveillance Monthly Performance Indicators July 1, 2008-June 30, 2009

Electronic laboratory reporting from laboratory to NYS and from NYS to NYC in calendar year 2008:

- Western Blot file received weekly (total N = 15,308)
- VL file received every two weeks (total N = 337,418)
- Low CD4 (<200) received every four weeks (total N = 95,479)
- High CD4 (200-499) received every 4-5 weeks (total N = 192,494)
- Very High CD4 (500+) received periodically (total N = 134,019)
- Total laboratory reports excluding genotype received 2008= 640,699
- Nucleotide sequences received monthly (total cumulative N 2008-2009= 71,074)

Total number of laboratory reports received and processed July 1, 2008-June 30, 2009: 453,791

- Mean 37,816 laboratory reports were received per month
- Mean 7,003 unique individuals were represented by these reports
- Mean 1,045 reports were potential new cases and initiated field investigations
- Mean 18,999 reports matched to previous cases

- Range 158-240 reports could not be assigned because of missing name, DOB, provider, or other critical matching data (specific labs flagged by city and state for corrective action)

NYS electronic laboratory reporting lag indicators 2008-9:

- Western Blot
    - Time from draw date to submission by laboratory (varies by lab, negotiated by state, range = 1-30 days, mean = 14 days)
    - Mean time from state to city 7 days
    - Total time to city mean = 21 days
- VL
    - Time from draw date to submission (varies by lab, negotiated by state, range = 1-30 days, mean = 7 days
    - Mean time from state to city = 21 days
    - Mean time from draw date to city = 30 days
- CD4
    - Varies by test result, transmissions every 3 weeks
    - Mean time from draw date to submission to state = 7 days
    - Mean time from state to city = 30 days
- Genotype
    - Mean time from draw date to submission by laboratory 90 days
    - Mean time from state to city = 30 days

Field investigations:

- 12,539 field investigations initiated on potential new cases (non-matches to registry July 1, 2008-June 30, 2009
- 4,472 (35.7%) dispositioned as new diagnoses
    - 20.9% previously reported
    - 40.1% non-cases
    - 2% patient not at site/chart not found
    - 1.5% out of jurisdiction
- Process:
    - 93% of new case investigations returned to surveillance within 2 months of initiation
    - 50% of new diagnoses had provider report form (PRF) submitted as required by NYS Public Health Law Article 21

Vital Status Ascertainment

- Quarterly matches of HIV Registry with NYC vital registry file on HIV-related cause of death
- Semiannual match of HIV Registry with NYC death registry for all causes of death
- Annual match of HIV Registry against Social Security Death Master File
- Annual or biannual match of HIV presumed living cases in HIV Registry against National Death Index

Internal Duplicates and Merges

- Program algorithm ("DupMerge") identified potential duplicate pairs:
  - Same last name, first name, DOB (16 pairs 2008-2009, 130 cumulative)
  - Same SSN (1 pair 2008)
  - Same death certificate and date of death (1 pair 2008, 400 cumulative)
  - Same NYSID number (500 cumulative pairs)
  - Other categories (300 cumulative pairs)
- Corrections
  - Identified, entered, and investigation initiated N=379
  - Investigation completed, fix in progress N=70
  - Investigation completed, fix accomplished N=253
  - Duplicates eliminated: 116 cases deleted
- New matching product in test mode now – IBM Quality Stage(
  - Uses reference dataset to standardize key variables, e.g., first name, last name, date of birth, Social Security number, street address, city and zip
  - Divides matching variables into blocks, sequentially matches and rates blocks in multiple data passes, and creates match weight
  - Test file: 4.6 million records in pre-eHARS laboratory database
    - 672,257 duplicate tests identified and removed
    - 3.7/3.9 million laboratory records rated "high scoring" matches by QS
    - Generated 3,500 cases for manual review
      - 780 duplicate pairs, many of which had already been identified by standard program algorithm and "DupMerge" facility
      - 2,700 cases with mismatched lab documents (represents 30,588 laboratory reports)

Interstate Duplicates – Routine Interstate Duplicate Review

- Backlog of 17,000 cases dating from 2005 was cleared in 2009, entered into Duplicate Review tab in eHARS and submitted to CDC

- Review completed of 2,795 (89%) potential duplicate pairs out of 3,148 potential interstate duplicates assigned for resolution by CDC in 2008.

Completeness and Timeliness of Reporting

- NYC used least squares regression to estimate number of new diagnoses expected in 2007
- Of 3,862 cases expected to be reported in 2007, 3,829 (99.2%) had been reported to the NYCDOHMH by December 31, 2008 (within one calendar year of the date of diagnosis

Appendix IIIE: Washington, D.C. Core HIV Surveillance System

The District has had AIDS reporting since 1985 and transitioned to names based HIV reporting in November, 2006. Since the transition to names based HIV reporting, the District has worked to develop policies and procedures to control the quality, completeness, accuracy and timeliness of HIV reporting data. The District of Columbia requires named reporting of all diagnoses of HIV and AIDS, all HIV-related illness, all positive Western Blot (WB) tests for HIV antibody, all VL and CD4 lymphocyte values, and all HIV genotypes. The District's enhanced HIV/AIDS Reporting System (eHARS) is a population-based registry that since 1981 has been continuously updated with new, de-duplicated diagnoses and laboratory results. All incoming provider and laboratory reports that do not match an existing registry record initiate a field investigation with medical record review to confirm the case, date and disposition of diagnosis and collect all other data required for surveillance and partner notification. The Districts eHARS also obtains data through routine matches with other disease registries; Sexually Transmitted Disease Surveillance system (STD*MIS), the Districts electronic Death Registry (eDeath Registry), electronic Birth Registry (eBirth) and HIV client level data from HRSA and AIDS Drug Assistance Program(ADAP) as well as the Social Security Death Master File. The surveillance program follows a set of monthly performance indicators to track data quality and staff and provider performance, and meets on a monthly basis to review indicators, identify problem areas and take appropriate action steps.

Laboratory Reporting Summary

According to the District's Laboratory Licensing Administration, there are 27 laboratories licensed to do HIV testing in the District. Annually these laboratories are surveyed to assess their testing volume and the types of testing they perform. In the District, positive western blots and all CD4 and VL values are reportable. In 2008, only 2 smaller testing laboratories were found not to be in compliance with HIV laboratory reporting requirements, accounting for less than 1% of testing in the District. Completeness of reporting has improved significantly since the Districts transition to names based HIV reporting in 2006 and has continued to improve annually as more labs in the District begin electronic laboratory reporting (ELR). To improve on timeliness of laboratory reporting, in August, 2009, laboratories were notified of the legal requirements for reporting and received detailed instructions on tests they are required to report including data from 2007-present. Below is a summary of the laboratory reports received during 2008 and 2009.

The following points summarize the volume and periodicity of electronic laboratory reporting, the volume, timeliness and outcome of field investigations, the periodic registry matches, internal matching and deduplication procedures, and the interstate deduplication activities of the program.

Electronic laboratory reporting from laboratory to DC-DOH

Estimated total number of laboratory reports received and processed in the District of Columbia, 2008-2009

Number of Lab Reports Per Year

Lab Test	2008	2009	Total
WBLOT	2,175	1,138	3,028
CD4CNT	5,029	5,532	9,178
CD4PCT	4,870	5,378	8,903
VL	11,634	12,428	20,955

- Western Blot file received monthly (total N = 126)
- VL file received monthly (total N = 873)
- Number and Proportion of CD4, Low CD4 (<200), monthly (total N = 191 (37.1%))
- Number and Proportion of CD4, High CD4 (200-499) monthly (total N = 166 (32.2%))
- Number and Proportion of CD4, Very High CD4 (500+) monthly (total N = 158 (30.7%)
- Number and Proportion of VL, Undetectable (<400 C/ML), monthly (total N = 491 (46.3%))
- Number and Proportion of VL, Detectable (400-10,000 C/ML) monthly (total N = 264 (24.9%))
- Number and Proportion of VL, Very High (>10,000 C/ML) monthly (total N = 305 (28.8%))
- Mean 2,005 laboratory reports were received per month
- Mean 176 reports were potential new cases and initiated field investigations. These reports on average were for 53 unique people
- Mean 1,174 reports matched to previous cases or to 387 unique people

De-duplication of eHARS

All "newly reported" HIV cases must undergo thorough de-duplication within the District and with other jurisdictions. Monthly de-duplication of HIV/AIDS is done prior to sending records to CDC and completed using version SAS 9.0 and Linkplus. The key data elements for matching include the following:

Name
Date of Birth
Gender
Social Security Number

Intrastate/Interstate De-Duplication

If a duplicate exists, the record with the earliest date of diagnosis is retained and any additional information is added to this case record. In addition to reconciling Routine Interstate De-Duplication lists generated by CDC, any cases under investigation that are reported with an indication of being an out of state resident are referred to the appropriate jurisdiction.

Review of other Documents to ensure completeness of reporting:

The District employs the following routine database matches to assess the completeness, timeliness and quality of HIV reporting data.

Vital Status Ascertainment

- Death certificates are received monthly from Vital Records. Quarterly matches with DC vital statistics database
- Annual match of HIV Registry against Social Security Death Master File

.

AIDS Drug Assistance Program (ADAP)

- Quarterly matches conducted to update existing case data and to identify new cases to eHARS surveillance database

Medicaid

- Claims for persons with ICD9/10 codes indicative of HIV/AIDS are reviewed quarterly to update existing case data and to identify new cases

Timeliness

Approximately 85% of District laboratory reports are received within 2 weeks of the test date via electronic reporting. The remaining 15% are reported via the US mail, averaging about a 3 week lag in reporting.

HPTN 065
TLC-Plus: A Study to Evaluate the Feasibility of an Enhanced Test, Link to Care, Plus Treat Approach for HIV Prevention in the United States

A Study of the HIV Prevention Trials Network

Sponsored by:

Division of AIDS (DAIDS), U.S. National Institute of Allergy and Infectious Diseases (NIAID)
U.S. National Institutes of Health (NIH)

Protocol Chair:
Wafaa El-Sadr, M.D., MPH
Columbia University and Harlem Hospital
New York, NY, USA

Protocol Co-Chair:
Bernard Branson, M.D.
Centers for Disease Control and Prevention (CDC)
Atlanta, GA, USA

Version 3.0
FINAL
14 January 2014

DAIDS Protocol #: 11685
Non-IND Study

HPTN 065

TLC-Plus: A Study to Evaluate the Feasibility of an Enhanced Test, Link to Care, Plus Treat Approach for HIV Prevention in the United States

TABLE OF CONTENTS

LIST OF ABBREVIATIONS AND ACRONYMS V
PROTOCOL TEAM ROSTER VIII
INVESTIGATOR SIGNATURE PAGE XIII
SCHEMA XIV
OVERVIEW OF STUDY DESIGN AND RANDOMIZATION SCHEME XVII
STUDY TIMELINE XVIII
1.0 INTRODUCTION 1
1.1 Background and Prior Research 1
1.2 Purpose 17
1.3 Rationale 18
1.4 Protocol Structure 23
2.0 EXPANDED HIV TESTING 23
2.1 Study Objectives for Expanded HIV Testing 23
2.2 Design for Expanded HIV Testing 24
2.3 Study Population for Expanded HIV Testing 24
2.4 Study Sites for Expanded HIV Testing 25
2.5 Interventions for Enhanced HIV Testing Activities 25
2.6 Study Procedures for Expanded HIV Testing 27
2.7 Study Duration for Expanded HIV Testing 32
2.8 Statistics and Data Analysis for Expanded HIV Testing 33
2.9 Human Subjects/Ethical Considerations 35
3.0 LINKAGE-TO-CARE 36
3.1 Study Objectives for Linkage-to-Care 36
3.2 Design for Linkage-to-Care 36
3.3 Study Population for Linkage-to-Care 37
3.4 Study Sites for Linkage-to-Care 37
3.5 Intervention for Linkage-to-Care 37
3.6 Study Procedures for Linkage-to-Care 38
3.7 Study Duration for Linkage-to-Care 40
3.8 Statistics and Data Analysis for Linkage-to-Care 41
3.9 Human Subjects/Ethical Considerations 45
3.10 Safety Monitoring and Adverse Event Reporting 46
4.0 VIRAL SUPPRESSION 46
4.1 Study Objectives for Viral Suppression 46
4.2 Design for Viral Suppression 47
4.3 Study Population for Viral Suppression 47

4.4 Study Sites for Viral Suppression 47
4.5 Intervention for Viral Suppression 48
4.6 Study Procedures for Viral Suppression 48
4.7 Study Duration for Viral Suppression 49
4.8 Statistics and Data Analysis for Viral Suppression 50
4.9 Human Subjects/Ethical Considerations 55
4.10 Safety Monitoring and Adverse Event Reporting 55
5.0 PREVENTION FOR POSITIVES 56
5.1 Study Objectives for Prevention for Positives 56
5.2 Design for Prevention for Positives 56
5.3 Study Population for Prevention for Positives 56
5.4 Study Sites for Prevention for Positives 57
5.5 Intervention for Prevention for Positives 57
5.6 Study Procedures for Prevention for Positives 58
5.7 Study Duration for Prevention for Positives 59
5.8 Safety Monitoring and Adverse Event Reporting 59
5.9 Statistics and Data Analysis for Prevention for Positives 59
5.10 Human Subjects/Ethical Considerations 63
6.0 SURVEY OF PATIENTS AND PROVIDERS 64
6.1 Study Objectives for the Survey of Patients and Providers 64
6.2 Design for the Survey of Patients and Providers 64
6.3 Study Population for the Survey of Patients and Providers 65
6.4 Study Sites for the Survey of Patients and Providers 65
6.5 Study Procedures for the Survey of Patients 65
6.6 Study Procedures for the Survey of Providers 66
6.7 Study Duration for the Survey of Patients and Providers 66
6.8 Statistics and Data Analysis for Surveys of Patients and Providers 66
6.9 Human Subjects/Ethical Considerations 67
6.10 Safety Monitoring and Adverse Event Reporting 68
7.0 HIV SURVEILLANCE, ROUTINELY-COLLECTED AND OTHER SURVEY DATA 68
7.1 HIV Testing Data 68
7.2 HIV Surveillance Data 71
7.3 Behavioral Data 74
8.0 ADMINISTRATIVE PROCEDURES AND OPERATIONAL CONSIDERATIONS 76
8.1 Study Activation 77
8.2 Study Coordination 77
8.3 Study Monitoring 78
8.4 Protocol Compliance 78
8.5 Human Subjects/Ethical Considerations 79
8.6 Use of Information and Publications 80
8.7 Study Discontinuation 80
9.0 REFERENCES 81
APPENDICES 89

APPENDIX I: SCHEDULE OF STUDY VISITS AND PROCEDURES 90
APPENDIX IIA: PATIENT COMPUTER-DELIVERED INTERVENTION AND SURVEY INFORMED CONSENT FORM..........92
APPENDIX IIB: PROVIDER SURVEY ONLINE INFORMED CONSENT TEXT 101
APPENDIX IIIA: ADULT HIV/AIDS CONFIDENTIAL CASE REPORT 103
APPENDIX IIIB: BEHAVIORAL RISK FACTOR SURVEILLANCE SYSTEM (BRFSS)..106
APPENDIX IIIC: 2008 PEMS VARIABLE REQUIREMENTS 108
APPENDIX IIID: NYC HIV SURVEILLANCE PERFORMANCE INDICATORS..115
APPENDIX IIIE: WASHINGTON, D.C. CORE HIV SURVEILLANCE SYSTEM...120

HPTN 065
TLC-Plus: A Study to Evaluate the Feasibility of an Enhanced Test, Link to Care, Plus Treat Approach for HIV Prevention in the United States

LIST OF ABBREVIATIONS AND ACRONYMS

AA	African American
ACASI	Audio Computer-Assisted Self Interview
ACTG	AIDS Clinical Trials Group
AIDS	Acquired Immunodeficiency Syndrome
ADAP	AIDS Drug Assistance Program
ALIVE	AIDS Link to Intravenous Experience
AMPATH	Academic Model for the Prevention and Treatment of HIV/AIDS
ART	Antiretroviral Therapy
ARTAS	Antiretroviral Treatment Access Study
ASD	Adult/Adolescent Spectrum of HIV Disease
ASO	AIDS Service Organization
BRFSS	Behavioral Risk Factor Surveillance System
CARE+	Computer Assessment and Risk Reduction Education for HIV-positives
CATI	Computer-Assisted Telephone Interviewing
CBO	Community-Based Organization
CD4	surface glycoprotein that denotes helper T cells
CDC	Centers for Disease Control and Prevention
CHS	Community Health Survey
CLIA	Clinical Laboratory Improvement Amendments
CORE	Coordinating and Operations Center
CRF	Case Report Form
CSTE	Counsel of State and Territorial Epidemiologists
CTRS	Counseling, Testing and Referral Services
DAIDS	Division of AIDS
DC	District of Columbia
DHHS	Department of Health and Human Services
DOB	Date of Birth
DOC	Department of Corrections
DOH	Department of Health
DOHMH	Department of Health and Mental Hygiene
ED	Emergency Department
eHARS	enhanced HIV/AIDS Reporting System
EIA	Enzyme Immunoassay
EMR	Electronic Medical Record
FI	Financial Incentive
FSU	Field Services Unit
FY	Fiscal Year
GE	General Electric
GEE	Generalized Estimating Equations
HbA1c	Hemoglobin A1C

LIST OF ABBREVIATIONS AND ACRONYMS (continued)

HAART	Highly Active Antiretroviral Therapy
HAHSTA	HIV/AIDS, Hepatitis, STD and TB Administration
HARS	HIV/AIDS Reporting System
HCPI	Health Communications/Public Information
HCSUS	HIV Cost and Services Utilization Study
HHS	Health and Human Services
HIPAA	Health Insurance Portability and Accountability Act
HIV	Human Immunodeficiency Virus
HOPS	HIV Outpatient Study
HOPWA	Housing Opportunities for People with AIDS
HPTN	HIV Prevention Trials Network
HRSA	Health Resources and Services Administration
ICC	Intra-class Correlation
ICF	Informed Consent Form
ICT	Information and Communication Technologies
ID	Identification
IDSA	Infectious Disease Society of America
IDU	Injecting Drug User
INR	International Normalized Ratio
IPV	Intimate Partner Violence
IRB	Institutional Review Board
JAIDS	Journal of Acquired Immune Deficiency Syndromes
LoA	Letter of Amendment
MEMS	Medication Event Monitoring System
MCO	Managed Care Organization
MMP	Medical Monitoring Project
MMP	Morbidity Monitoring Project
MMWR	Morbidity and Mortality Weekly Report
MSM	Men who have Sex with Men
MTRH	Moi Teaching and Referral Hospital
NAAT	Nucleic Acid Amplification Testing
NHANES	National Health and Nutrition Examination Survey
NHBS	National HIV Behavioral Surveillance
NHBS-HET	National HIV Behavioral Surveillance – At Risk Heterosexuals
NHBS-IDU	National HIV Behavioral Surveillance – Injection Drug Users
NHBS-MSM	National HIV Behavioral Surveillance – Men who have Sex with Men
NHIS	National Health Interview Survey
NIAID	National Institute of Allergy and Infectious Diseases
NIH	National Institutes of Health
NIMH	National Institutes of Mental Health
NL	Network Laboratory
NY	New York

LIST OF ABBREVIATIONS AND ACRONYMS (continued)

NYC	New York City
OARAC	Office of AIDS Research Advisory Committee
OHRP	Office for Human Research Protections
PEMS	Program Evaluation and Monitoring System
PHMC	Public Health Management Corporation
PLWHA	People Living With HIV/AIDS
PRO	Protocol Registration Office
RSC	Regulatory Support Center
RCT	Randomized Controlled Trial
RFA	Request for Applications
RNA	Ribonucleic Acid
SCHARP	Statistical Center for HIV/AIDS Research and Prevention
SDMC	Statistics and Data Management Center
SES	Socio-economic Status
SHAS	Supplement to HIV/AIDS Surveillance
SMART	Strategies for Management of Antiretroviral Therapy
SMC	Study Monitoring Committee
SOC	Standard of Care
SQL	Structured Query Language
SSP	Study-Specific Procedures Manual
STD	Sexually Transmitted Disease
TA	Technical Assistance
TB	Tuberculosis
TLC	Test and Link to Care
UODT	Universal Offer Development Team
U.S.	United States
USPHS	United States Public Health Service
USA	United States of America
VL	Viral Load
WB	Western Blot
WHO	World Health Organization

HPTN 065
TLC-Plus: A Study to Evaluate the Feasibility of an Enhanced Test, Link to Care, Plus Treat Approach for HIV Prevention in the United States

PROTOCOL TEAM ROSTER

Geetha Beauchamp, MS
SCHARP, Vaccine and Infectious Disease Institute
Fred Hutchinson Cancer Research Center
Seattle, Washington
Phone (206) 667-6167
Fax: (206) 667-4812
Email: geetha@scharp.org

Nanette Benbow, MAS
Director
Surveillance, Epidemiology and Research Section
STI/HIV Division
Chicago Department of Public Health
333 South State Street, Room 2150
Chicago, IL 60604
Phone: (312) 747-9620
Fax: (312) 745-3923
Email: nanette.benbow@cityofchicago.org

Constance Benson, M.D.
Professor of Medicine
Division of Infectious Diseases
Principal Investigator/Chair, AIDS Clinical Trial Groups
I.D. Fellowship Program Director
Director, Antiviral Research Center
University of California, San Diego
U.S. Mail: 200 W. Arbor Drive
Mail Code 8208
San Diego, CA 92103-8208
FedEx, UPS, or Overnight Mail:
220 Dickinson Street, Suite A
San Diego, CA 92103
Phone: (619) 543-8080
Fax: (619) 543-5066
Email: cbenson@ucsd.edu

Judith Berger, M.D.
Chief, Division of Infectious Diseases and HIV Care: Medical Director Pathways Center for Comprehensive Care
St. Barnabas Hospital
4422 Third Ave
Bronx, NY 10457
Phone: (718) 960-6205
Fax: (718) 960-3218
Email: jberger@sbhny.org

Kathleen A. Brady, M.D.
Medical Director/Medical Epidemiologist
AIDS Activities Coordinating Office
Philadelphia Department of Public Health
1101 Market St., 8th Floor
Philadelphia, PA 19107
Phone: (215) 685-4778
Fax: (215) 685-4774
Email: Kathleen.A.Brady@phila.gov

Bernard Branson, M.D.
Center for Disease Control and Prevention
Division of HIV/AIDS Prevention
1600 Clifton Road MS D-21
Atlanta Georgia 30333
Phone: (404) 639-6166
Fax: (404) 639-0897
Email: BBranson@cdc.gov

Kate Buchacz, Ph.D.
Centers for Disease Control and Prevention
Divisions of HIV/AIDS Prevention
1600 Clifton Road, MS E-45
Atlanta, GA 30333
Phone: (404) 639-5167
Fax: (404)-639-6127
E-mail: acu7@cdc.gov

David Burns, M.D., M.P.H.
Division of AIDS, Prevention Research Branch, National Institute of Allergy and Infectious Diseases
6700 B Rockledge Drive
Bethesda, MD, 20892
Phone: (301) 435-8896
Fax: (301) 496-8530
Email: burnsda@niaid.nih.

Ruth Concepcion, M.S.W.
Psychosocial Counselor / Walk-In Supervisor
Ryan White Part D and Linkage to Life Program
Dominican Sisters Family Health Service, Inc.
279 Alexander Avenue
Bronx, NY 10454
Phone: (718) 665-6558, x1413
Fax: (718) 665-9297
Email: RConcepcion@dsfhs.org

Blayne Cutler, M.D., Ph.D.
Director, HIV Prevention
Bureau of HIV/AIDS Prevention and Control
New York City Department of Health and Mental Hygiene
40 Worth Street, CN-A1
New York, NY 10013
Phone: (212) 788-4484
Email: bcutler@health.nyc.gov

Deborah Donnell, Ph.D.
SCHARP, Vaccine and Infectious Disease Institute
Fred Hutchinson Cancer Research Center
1100 Fairview Ave. N.,
Mailstop: M2-C200
P.O. Box 19024
Seattle, WA 98109-1024
Phone: (206) 667-5661
Email: deborah@scharp.org

Vanessa Elharrar, M.D., M.P.H.
Medical Officer, Deputy Branch Chief
Clinical Prevention Research Branch
PSP/DAIDS/NIAID/NIH
6700-B Rockledge Drive, 5th Fl. Rm. 5125
Bethesda, MD 20892
Phone: (301) 827-0845
Fax: (301) 402-3684
Email: elharrarva@niaid.nih.gov

Rick Elion, M.D.
Whitman Walker Clinic
1701 14th St., NW
Washington, DC 20009
Phone: (202) 745-6142
Fax: (202) 745-0238
Email: Rickelion@gmail.com

Wafaa El-Sadr, M.D., M.P.H.
Columbia University and Harlem Hospital
Mailman School of Public Health
722 West 168th Street
Room 715
New York, NY 10032
Phone: (212) 342-0532 or (212) 939-2936
Fax: (212) 342-1824 or (212) 939-2968
Email: wme1@mail.cumc.columbia.edu

Lisa K. Fitzpatrick. M.D., M.P.H.
Medical Director, Infectious Diseases Care Center
Scientific Liaison, NIH DC Program for AIDS Progress
1328 Southern Avenue, SE, Suite #216
Washington, DC 20032
Phone: (202) 574-6909
Email: lfitzpatrick@united-medicalcenter.com

Donna Futterman, M.D.
Director, Adolescent AIDS Program
Professor of Clinical Pediatrics
Montefiore Medical Center
Albert Einstein College of Medicine
111 East 210th Street
Bronx, NY 10467
Phone: (718) 882-0322
Email: dfutterman@adolescentAIDS.org

Theresa Gamble, Ph.D.
FHI 360
2224 E. NC 54
Durham, NC 27713
Phone: (919) 544-7040, Ex 11350
Fax: (919) 544-7261
Email: tgamble@fhi360.org

Fred Gordin, M.D.
Chief, Infectious Diseases
Professor of Medicine
The George Washington University
Veterans Affairs Medical Center
50 Irving St. NW
Washington, DC 20422
Phone: (202) 745-830l
Fax: (202) 745-8432
Email: Fred.Gordin@va.gov

Becky Grigg, Ph.D.
Bureau of HIV/AIDS
4052 Bald Cypress Way, Bin #A09
Tallahassee, Florida 32399-1715
Phone: (850) 245-4432
Fax: (850) 922-4263
Email: Becky_Grigg@doh.state.fl.us

Cynthia Grossman, Ph.D.
National Institute of Mental Health
6001 Executive Boulevard
Room 6201, MSC 9619
Bethesda, MD, 20892
Phone: (301) 443-8962
Email: grossmanc@mail.nih.gov

H. Irene Hall, Ph.D.
CDC - Division of HIV/AIDS Prevention
National Center for HIV/AIDS, Viral
Hepatitis, STD, and TB Prevention
1600 Clifton Road, MS E 47
Atlanta, Georgia 30333
Phone: (404) 639-2050
Fax: (404) 639-2980
Email: ixh1@cdc.gov

Sally Hodder, M.D.
UMDNJ- Medical School
185 South Orange Ave., MSB I-510
Newark, NJ 07103
Phone: (973) 972-3846
Fax: (973) 972-2122
Email: hoddersa@umdnj.edu

Jessica Justman, M.D.
Columbia University
Mailman School of Public Health
722 West 168th Street, Room #714
New York, NY 10032
Phone: (212) 342-0537
Fax: (212) 342-1824
E-mail: jj2158@columbia.edu

Michael Kharfen
Senior Deputy Director
HIV/AIDS, Hepatitis, STD & TB
Administration
D.C. Department of Health
899 N. Capitol St., NE, Washington, DC
20002
Phone: (202) 671-4843
Phone (cell): (202) 262-3996
Fax: (202) 671-4860
Email: Michael.Kharfen@dc.gov

Ann Kurth, CNM, Ph.D.
Professor, New York University
College of Nursing
726 Broadway, Rm. 1006
New York, NY 10003
Phone: (212) 998-5316
Fax: (212) 995-3413
Cell: (206) 795-3616
Email: akurth@nyu.edu

Jason Leider, M.D., Ph.D.
North Bronx Health Care Network
1400 Pelham Parkway South
Building #5, Rm 622
Bronx, NY 10461
Phone: (718) 918-3669
Fax: (718) 918-7686
Email: Jason.Leider@nbhn.net

Kenneth Mayer, M.D.
Brown University School of Medicine
Miriam Hospital of Rhode Island
Division of Infectious Diseases
164 Summit Avenue
Providence, R.I. 02906
Phone: (401) 793-4710
Fax: (401) 793-4709
Email: Kenneth_Mayer@brown.edu

Jeffrey Meyer, M.D., M.P.H.
Epidemiologist Supervisor
Bureau of Epidemiology, 4th Floor
Houston Department of Health and Human Services
8000 N Stadium Drive
Houston, Texas 77054
Phone: (832) 393-4567
Fax: (832) 395-9955
Email: jeffrey.meyer@houstontx.gov

June Pollydore
Rapid Testing Program Coordinator
1331 Rhode Island Ave NE
Washington, DC 20018
Phone: (202) 483-7003
Fax: (202) 483-7330
Email: june@womenscollective.org

Candia Richards-Clarke, M.P.H.
Chief Operating Officer
Bronx AIDS Services, Inc.
540 East Fordham Rd
Bronx, NY 10458
Phone: (718) 295-5605
Fax: (718) 733-3429
Email: ctrichards@basnyc.org

Paul Richardson, M.Sc.
Johns Hopkins University
600 North Wolfe Street
Pathology 313
Baltimore, MD 21287
Phone: (410) 502-0435
Fax: (410) 614-0430
Email: pricha18@jhmi.edu

Nirupama Sista, Ph.D.
FHI 360
2224 E. NC 54
Durham, NC 27713
Phone: (919) 544-7040, Ext. 11590
Fax: (919) 544-7261
Email: nsista@fhi360.org

Edward E. Telzak, M.D.
Chief, Division of Infectious Diseases
Director, AIDS Program
Bronx-Lebanon Hospital Center
1650 Grand Concourse
Bronx, NY 10456
Phone: (718) 960-1212
Fax: (718) 960-2054
Email: etelzak@bronxleb.org

Lucia V. Torian, Ph.D.
Deputy Director, HIV Epidemiology and Field Services Program
New York City Department of Health and Mental Hygiene
125 Worth St., CN#44
New York, NY 10013
Phone: (212) 442-3461
Fax: (212) 442-3482
Email: ltorian@health.nyc.gov

Melissa Turner, M.S.W., LICSW
Veteran Affairs Medical Center
Department of Infectious Diseases
50 Irving Street NW
Room 2C-211A
Washington, D.C. 20422
Phone: (202) 745-8695
Phone: (202) 745-8000, Ext. 5667
Fax: (202) 745-8432
Email: melissa.turner@va.gov

Kevin Volpp, M.D., Ph.D.
Staff Physician, CHERP,
Philadelphia VA Medical Center
Director, Center for Health Incentives,
Leonard Davis Institute of Health Economics
Associate Professor, University of Pennsylvania School of Medicine and the Wharton School
1232 Blockley Hall, 423 Guardian Drive
Philadelphia, PA 19104-6021
Phone: (215) 573-0270
Fax: (215) 573-8778

Douglas J. Ward, M.D.
DuPont Circle Physicians Group
1737 20th Street, North West
Washington, DC 20009
Phone: (202) 745-0201
Fax: (202) 332-2794
Email: dward@dupontdocs.com

HPTN 065
TLC-Plus: A Study to Evaluate the Feasibility of an Enhanced Test, Link to Care, Plus Treat Approach for HIV Prevention in the United States
Version 3.0/ 14 January 2014

INVESTIGATOR SIGNATURE PAGE

A Study of the HIV Prevention Trials Network (HPTN)

Sponsored by:
Division of AIDS (DAIDS), U.S. National Institute of Allergy and Infectious Diseases (NIAID)
U.S. National Institutes of Health (NIH)

I, the Investigator of Record, agree to conduct this study in full accordance with the provisions of this protocol. I agree to maintain all study documentation for a minimum of three years after submission of the site's final Financial Status Report to the Division of AIDS (DAIDS), unless otherwise specified by DAIDS or the HIV Prevention Trials Network (HPTN) Coordinating and Operations Center. Publication of the results of this study will be governed by HPTN policies. Any presentation, abstract or manuscript will be made available by the investigators to the HPTN Manuscript Review Committee and DAIDS for review prior to submission.

I have read and understand the information in this protocol and will ensure that all associates, colleagues and employees assisting in the conduct of the study are informed about the obligations incurred by their contribution to the study.

____
Name of Investigator of Record

________________________________ ________________________________
Signature of Investigator of Record Date

HPTN 065

TLC-Plus: A Study to Evaluate the Feasibility of an Enhanced Test, Link to Care, Plus Treat Approach for HIV Prevention in the United States

SCHEMA

Purpose: The main purpose of this study is to evaluate the feasibility of an enhanced community-level test, link to care, plus treat strategy in the United States. The study includes the following components:

- Expanded Human Immunodeficiency Virus (HIV) Testing
- Linkage-to-Care
- Viral Suppression
- Prevention for Positives
- Patient and Provider Surveys

Design: Each component of the study involves an independent design but is interrelated to the other components.

- The Expanded HIV Testing component involves social mobilization, with targeted messaging to promote testing, and implementation of the universal offer of HIV testing in emergency departments (EDs) and hospital inpatient admissions.
- The Linkage-to-Care and Viral Suppression components involve site randomization to test the effectiveness of a financial incentive (FI) intervention compared with the standard of care (SOC).
- The Prevention for Positives component uses individual randomization to compare the SOC plus a computer-delivered intervention with the SOC.
- The Patient and Provider Surveys will be administered at specific time points during the study to assess knowledge, attitudes and practices regarding early initiation of antiretroviral therapy (ART) and the FI interventions.

Study Sites: The study will be conducted in two intervention communities (the Bronx, New York and Washington, D.C.) and surveillance data from these communities will be compared with that from four non-intervention communities (Chicago, Illinois; Houston, Texas; Miami, Florida; and Philadelphia, Pennsylvania).

Study Population: This study will primarily target individuals age 18 years and older, and will also include younger individuals who are legally able to consent for HIV testing and care according to the state or local law in the two study communities. All of the study components will include HIV-positive individuals, with the exception of the provider survey sub-component.

Study Objectives: The main objective of the study is to determine the feasibility of a community focused enhanced test and link-to-care strategy in the United States. The study includes feasibility objectives for the Expanded HIV Testing, Linkage-to-Care and Viral Suppression components, and effectiveness objectives for the Linkage-to-Care, Viral Suppression and Prevention for Positive components.

Study Duration: The study will take place over 36 months.

SCHEMA (Continued)

Study Components:

The study includes the following five components:

Expanded HIV Testing: The expanded HIV testing intervention will supplement ongoing social mobilization and HIV testing efforts already in place in the two intervention communities. Refined messages will be added to ongoing social marketing and social networking efforts, targeted to increase HIV testing and testing frequency among men who have sex with men (MSM) and other subpopulations disproportionately affected by HIV. The intervention for EDs and hospital admissions includes encouraging hospital leadership and staff to institute standing orders for universal HIV testing. It also includes providing financial support for an increased number of HIV tests, and use of novel mechanisms to deliver information about HIV testing to patients.

Linkage-to-Care: HIV test sites will be randomized to either the FI intervention or to the SOC for linkage of HIV-positive patients identified at the testing sites to HIV care sites. At HIV test sites assigned to FIs, HIV-positive patients will be provided with a coupon to redeem at participating HIV care sites in the community. Upon completion of HIV laboratory testing, patients with coupons will be given an FI ($25 gift card) at an HIV care site. A $100 gift card will be provided to patients upon completion of a care visit that includes interaction with a healthcare provider and discussion of HIV laboratory test results (*e.g.* CD4 cell count and viral load (VL) measurements).

Viral Suppression: HIV care sites will be randomized to either the FI intervention or the SOC for the achievement and maintenance of viral suppression. HIV care sites assigned to the FI intervention will provide an FI ($70 gift card) to HIV-positive patients on antiretroviral therapy (ART) demonstrating a suppressed VL (as defined by <400 copies/mL) at quarterly care visits.

Prevention for Positives: In a subset of patients enrolled from select HIV care sites in the two intervention communities, patients will be randomized either to an intervention arm (receiving SOC prevention activities plus a computer-delivered intervention for sexual and behavioral risk reduction) or to the control arm (receiving only the SOC prevention activities at the care site). In the intervention arm a modified version of the computerized counseling platform called Computer Assessment and Risk Reduction Education for HIV-positives (CARE+) will be used with an Audio Computer Assisted Self-Interviewing technique (ACASI). The CARE+/ACASI intervention session will ascertain behavioral risk, assess self-efficacy/motivation, and provide tailored feedback on specific risk behaviors. The computer-delivered intervention session will be administered every three months for one year. Participants in the control arm will also have CARE+/ACASI sessions every three months. However, participants in the control arm will only be administered behavior assessments via CARE+/ACASI and will not receive prevention messaging.

SCHEMA (Continued)

Study Components (continued):

Patient and Provider Surveys: The same computer system used for the Prevention for Positives intervention will be used to administer a survey to patients enrolled in that study component. The survey will assess their knowledge and attitudes towards ART use for treatment and prevention, ART adherence and FIs. Providers from these sites will be invited to complete a Web-based survey regarding their knowledge, attitudes and practices concerning ART for treatment and prevention as well as use of FIs. Both surveys will also collect some key sociodemographic data on surveyed populations to allow more in depth characterization of these populations.

Study Size: Each of the five study components has a target sample size.

- The universal offer of HIV testing will be made in EDs and during hospital admission at ~seven facilities in the Bronx, NY and ~seven in Washington, D.C. Additional focused messages promoting testing will be targeted to the entire population of each intervention community: the Bronx (population 1.4 million) and Washington, D.C. (population 600,000).
- The Linkage-to-Care component includes 40 HIV test sites (20 in each intervention community) and 40 HIV care sites (20 in each intervention community). We project that, by the end of the study, approximately 3000 new individuals in the two intervention communities will have tested positive for HIV.
- The Viral Suppression component includes the 40 HIV care sites (20 in each intervention community). Throughout the study duration, approximately 30,000 HIV-positive individuals will be in care, with an estimated 75% (22,500) eligible for ART in the two intervention communities.
- The Prevention for Positives component will be conducted at a total of twelve sites (six in each intervention community) with up to a total of 1320 patients participating.
- The patients at the twelve sites participating in the Prevention for Positives component will be surveyed. Providers at all participating HIV care sites will be invited to complete the provider survey.

HPTN 065
TLC-Plus: A Study to Evaluate the Feasibility of an Enhanced Test, Link to Care, Plus Treat Approach for HIV Prevention in the United States

OVERVIEW OF STUDY DESIGN AND RANDOMIZATION SCHEME

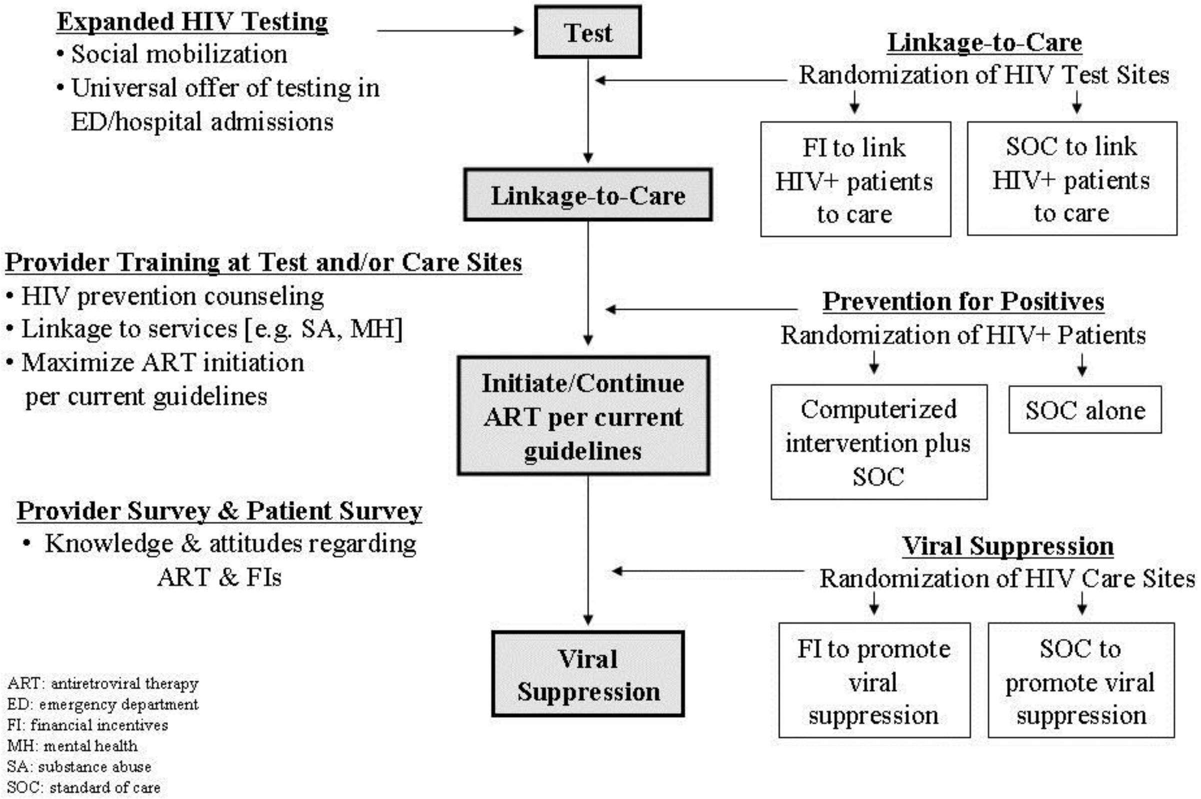

Summary of Study Components

Study Component	Design	Outcomes
Expanded HIV Testing	Descriptive, ecologic study	Feasibility
Linkage-to-Care	Two-arm, site-randomized, prospective, effectiveness study	Feasibility and effectiveness
Viral Suppression	Two-arm, site-randomized, prospective, effectiveness study	Feasibility and effectiveness
Prevention for Positives	Two-arm, individually-randomized, prospective, effectiveness study	Effectiveness
Patient and Provider Surveys	Quantitative	Survey

HPTN 065
TLC-Plus: A Study to Evaluate the Feasibility of an Enhanced Test, Link to Care, Plus Treat Approach for HIV Prevention in the United States

STUDY TIMELINE

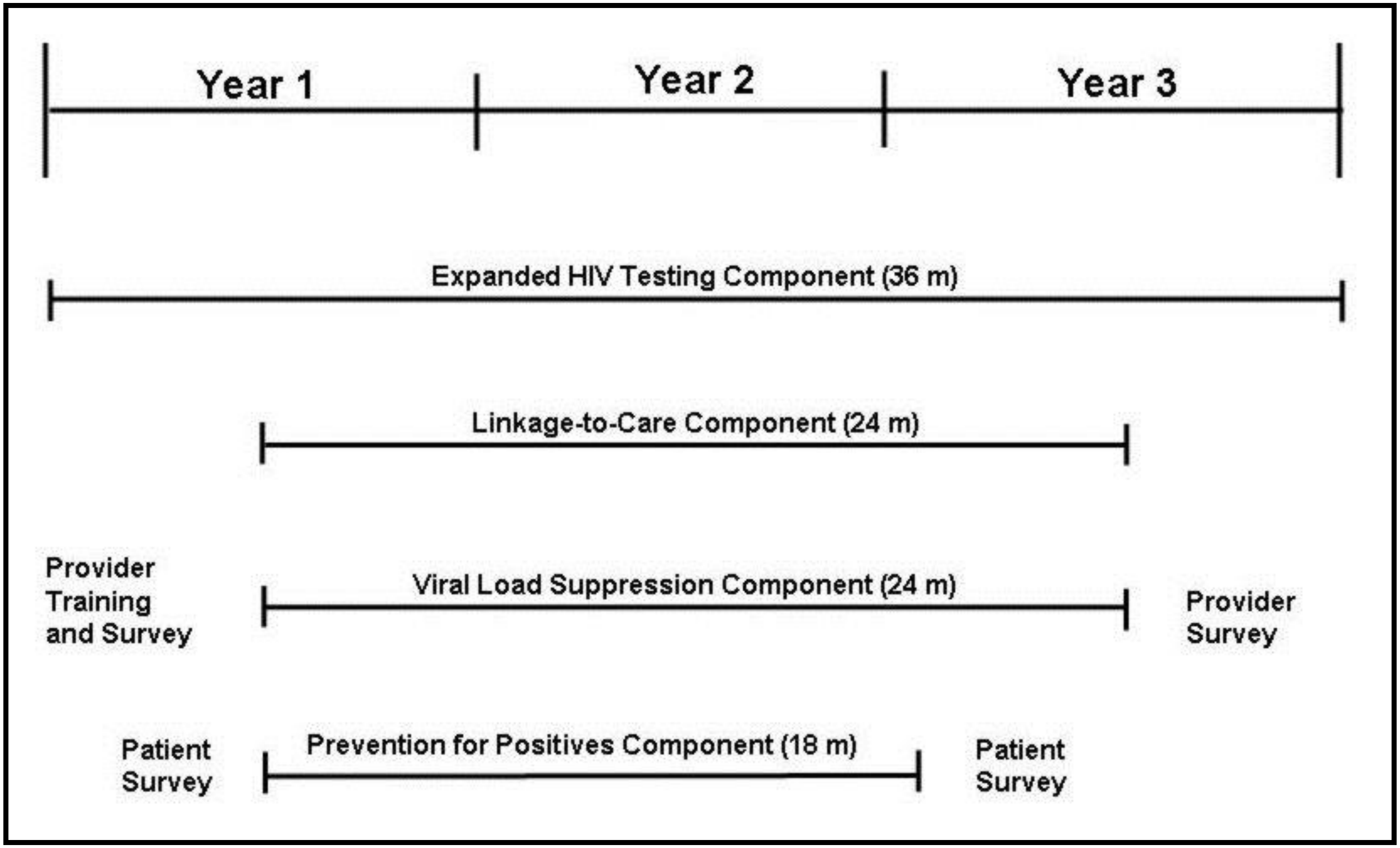

1.0 INTRODUCTION

1.1 Background and Prior Research

1.1.1 The Test-and-Treat Approach

The test-and-treat (TNT) strategy to prevent human immunodeficiency virus (HIV) transmission is based on expanded HIV testing to identify undiagnosed HIV infection, combined with prompt and effective initiation of ART to lower HIV viral load (VL) levels. While the potential effect of ART on HIV transmission has been previously studied by several investigators (Cohen, Gay et al. 2007), it received renewed attention in 2009. Granich and colleagues at the World Health Organization (WHO) published results of a modeling exercise that assessed the potential effect of such an approach on the HIV epidemic in South Africa (Granich, Gilks et al. 2009). The assumptions in the model included annual HIV testing of all adults older than 15 years of age, prompt initiation of ART in all those infected irrespective of disease stage, and a 99% decrease in infectiousness with highly effective first-line ART. The model indicated a dramatic drop in HIV incidence within 10 years and reduction of HIV prevalence to 1 percent in 50 years. The results of this modeling exercise have generated interest in the evaluation of such an approach (Assefa and Lera 2009; Cohen, Mastro et al. 2009; Dieffenbach and Fauci 2009; Epstein 2009; Granich, Gilks et al. 2009; Hsieh and de Arazoza 2009; Jurgens, Cohen et al. 2009; Wagner and Blower 2009).

The TNT strategy is hypothesized to achieve its effect on HIV transmission through the following two pathways:

- HIV testing identifies HIV-positive persons who, after learning their status, adopt safer behaviors, which decreases HIV transmission.
- HIV-positive individuals who initiate ART, and then maintain high levels of adherence and achieve viral suppression, are less infectious, which decreases HIV transmission.

Figure 1: Test and Treat Concept

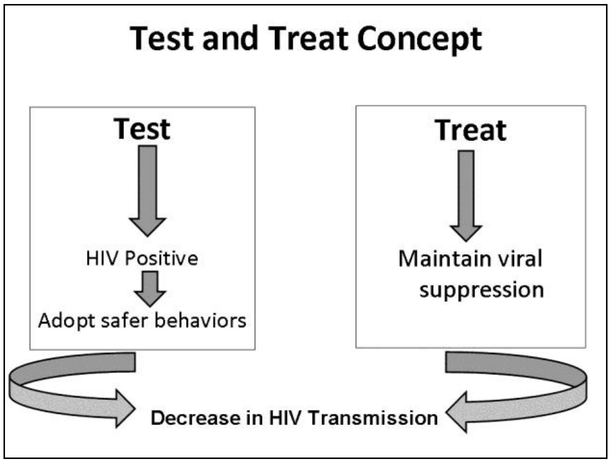

HIV screening has been found to be as cost-effective as other routine health interventions (Paltiel, Weinstein et al. 2005; Sanders, Bayoumi et al. 2005) and initiatives to expand HIV screening are underway in the United States. However, to optimize health outcomes, expanded testing efforts must be coupled with initiatives that ensure that both the newly diagnosed and those already known to have HIV infection are effectively linked to HIV care (Walensky, Weinstein et al. 2005) and receive ART as indicated with optimal adherence and suppression of viral replication.

The use of ART for prevention is supported by observational data from discordant couples that suggest that use of ART by the HIV sero-positive partner is associated with lower HIV incidence in the sero-negative partner (Quinn, Wawer et al. 2000; Sullivan, Kayitenkore et al. 2009). Other data also indicate a decrease in rates of HIV infection with use of ART (Bunnell, Ekwaru et al. 2006). However, definitive data for the effect of ART on transmission awaits the outcome of HIV Prevention Trials Network (HPTN) 052/AIDS Clinical Trials Group (ACTG) 5245. In addition, if ART is to be used widely for prevention purposes, its risk/benefit needs to be defined for a population not eligible for ART based on current therapeutic guidelines. Definitive data are lacking for the optimal timing of ART initiation for clinical benefit (Wilkin and Gulick 2008). Two ongoing randomized clinical trials (RCTs), the INSIGHT START study and HPTN 052/ACTG 5245, should yield such data on the efficacy and safety of initiation of ART at higher CD4 cell counts than the currently recommended threshold (Panel on Antiretroviral Guidelines for Adults and Adolescents 2009). In HPTN 052, HIV-positive individuals with CD4 count between 350 and 550 cells/mm^3 and who have HIV-discordant partners are randomized to initiate ART at CD4 cell count between 350 and 550 cells/mm^3 or at CD4 cell count between 200 and 250 cells/mm^3. The purpose of the latter study is to determine the effectiveness of ART on the sexual transmission of HIV to the uninfected partner as well as to determine the long-term effectiveness and safety of use of ART. In the INSIGHT START Study, HIV -positive patients with CD4 cell count of >500 cells/mm^3 are randomized to immediate ART initiation versus deferral of ART to when the CD4 cell count falls below 350 cells/mm^3.

1.1.2 HIV Testing in the United States, Washington, D.C., and the Bronx

1.1.2.1 Current HIV Testing in the United States

Based on data from the 2006 National Health Interview Survey (NHIS), the Centers for Disease Control and Prevention (CDC) estimates that 40% of all non-elderly adults (18 - 64 years) in the United States have been tested for HIV. A much smaller percentage of this population (10%) reported testing for HIV within the year prior to the survey. These levels of HIV testing, for both having ever been tested and for those tested in the past year, have remained constant from 2001 to 2006, suggesting that some individuals are tested repeatedly, while the majority of the U.S. population (60%) remains untested.

Approximately 21% of HIV-positive patients in the United States are not aware of their HIV infection (Campsmith, Rhodes et al. 2008) and not only may miss the benefits of HIV care and timely ART but also may continue to transmit HIV to their partners.

To increase the level of HIV testing in the United States and reduce the frequency of late HIV diagnoses, the CDC revised its recommendations for HIV testing in healthcare

settings and several federal agencies have new initiatives to increase testing in specific subpopulations (Duran, Beltrami et al. 2008). In 2006, the CDC recommended opt-out HIV screening as part of routine clinical care for adults and adolescents, including pregnant women, in all healthcare settings (Branson, Handsfield et al. 2006). In order to lower some of the barriers to testing, CDC recommendations require neither separate written consent nor HIV-prevention counseling as mandatory elements of HIV testing.

In September 2007, CDC funded an Expanded Testing Initiative in the 26 U.S. jurisdictions with the largest number of Acquired Immunodeficiency Syndrome (AIDS) cases. Through June 2009, 1.2 million persons have been tested under the Expanded Testing Initiative, with identification of nearly 14,400 new HIV diagnoses, 63% of which were among persons previously unaware they were infected. These tests were performed in Emergency Departments EDs (34%), sexually transmitted disease (STD) clinics (26%), community health centers (18%), corrections facilities (13%), and hospital inpatient settings (1.4%).

1.1.2.2 Current HIV Testing Activities in New York City and the Bronx

In the past three years, the New York City (NYC) Department of Health and Mental Hygiene (DOHMH) has scaled up its efforts to support routine HIV testing throughout its jurisdiction. As part of that effort, “The Bronx Knows” project was conceptualized. This program was initiated in early 2007 with the intention of scaling up HIV testing throughout the borough of the Bronx. After one year of capacity building, “The Bronx Knows” initiative was launched publicly on June 27, 2008 (National HIV Testing Day). The goals of the initiative are three-fold: to ensure that all Bronx residents who have never had an HIV test are screened for HIV over three years, to identify Bronx residents with undiagnosed HIV, and to link these HIV-positive individuals to high quality care and supportive services. While these are the stated goals of the initiative, residents with ongoing risk for HIV acquisition are also encouraged to be tested at least annually, per CDC recommendations.

As part of “The Bronx Knows,” the NYC DOHMH provides free test kits to organizations that offer HIV testing to uninsured individuals and to Community-Based Organizations (CBOs) that do not have dedicated funds for HIV testing. The DOHMH also provides technical assistance (TA) in the following areas: logistical/infrastructure change to support routine HIV screening (including on-site TA), obtaining clinical laboratory improvement amendment (CLIA) waivers, billing/reimbursement, rapid HIV testing technologies and data collection. In addition, the NYC DOHMH conducts sector-based workshops and Webinars in which participants review best practices and barriers experienced in their own sector (hospital, clinic or CBO). Finally, DOHMH provides ongoing social marketing and media campaigns targeting both Bronx residents (promoting routine HIV testing) and providers (promoting routine screening of patients for HIV).

By January 2009, more than 70 organizations had joined “The Bronx Knows.” These include seven of eight Bronx hospitals, 39 community health clinics and 20 of the borough's largest CBOs. Other participants include colleges/universities, faith-based institutions, local commercial establishments and community boards. Preliminary

aggregate data show that HIV testing among reporting organizations has increased by 28% since the launch of the initiative, despite New York State's requirement of separate signed informed consent for HIV testing.

1.1.2.3 Current HIV Testing Activities in Washington, D.C.

The Washington, D.C. Department of Health (DOH) has made HIV its number one health priority. Efforts have focused on aggressive diagnosis and treatment of HIV throughout Washington, D.C. Washington, D.C. does not require separate signed consent for HIV testing and was the first jurisdiction in the country to commit to and implement a policy of routine, opt-out HIV testing for residents. In June 2006, Washington, D.C. launched its, "Come Together D.C. - Get Screened for HIV" campaign. This campaign promoted testing among residents and recruited new testing organizations and testing sites. Starting in fiscal year 2007 (FY07) and continuing through FY08, testing expansion focused on scaling up routine, opt-out testing in the Department of Corrections (DOC) jail settings as well as in medical settings with special emphasis on hospital EDs, primary medical settings, managed care organizations (MCOs) and CBOs. In 2008, Washington, D.C. began implementation of a six-pronged strategy to scale up routine HIV screening citywide. The goals of this strategy are to: 1) increase the scope and scale of routine screening for HIV in clinical settings; 2) provide HIV testing and referral services, with an emphasis on identifying newly infected persons and ensuring that test results are received; 3) establish models to more efficiently link HIV-positive individuals to care; 4) collaborate with DOCs, medical care entities and CBOs to encourage and support Counseling, Testing and Referral Services (CTRS); 5) collect and analyze data to determine the scope and reach of CTRS programs; and 6) develop and disseminate tools to address myths and barriers related to routine HIV screening.

Despite efforts to increase provider awareness and practice of routinely offering HIV testing, behavioral survey and testing data suggest missed opportunities for routine testing in medical settings are still quite frequent, with nearly 75% of newly diagnosed HIV-positive persons reporting having seen a healthcare provider in the past twelve months without having been diagnosed (NHBS-Het Survey 2009). As a complement to efforts to increase provider participation in routine HIV testing, Washington, D.C.'s expansion efforts also include a direct-to-consumer marketing campaign called "Ask for the Test," which seeks to drive consumer demand by encouraging clients to ask for HIV testing if it is not routinely offered. It is anticipated that the approaches described above could result in an additional 85,000 tests per year.

1.1.3 HIV Care and ART Utilization in the United States, Washington, D.C. and the Bronx

The most recent population-based estimates on the proportion of HIV-positive Americans who are in HIV care and receiving ART are from 2003. These estimates depend on: 1) the fraction of HIV-positive persons who are undiagnosed and unaware of their HIV infection; 2) the fraction who have been diagnosed with HIV but are not in care; and 3) the changing recommendations with respect to timing of initiation of ART. Using data from the CDC's national HIV surveillance system and its 10-city Adult/Adolescent Spectrum of HIV Disease (ASD) project, the CDC estimated that, as of 2003, there were

480,000 Americans between the ages of 15 and 49 living with HIV or AIDS who were eligible for ART at a threshold of CD4 cell count < 350 cells/mm^3 (Teshale, Kamimoto et al. 2005). Of all eligible persons, approximately 340,000 (71%) were diagnosed with HIV and in care. Only 268,000 (55% of all eligible) were receiving ART. Based on these findings, the CDC recommended that three critical components be incorporated into the national HIV-prevention strategy: 1) increasing the number of HIV-positive individuals who are aware of their status; 2) linking them to HIV-prevention and care services; and 3) increasing the number receiving ART per federal guidelines (Branson, Handsfield et al. 2006). Given the growing number of HIV-infected persons living in the United States, estimated at 1.1 million as of 2006 (Campsmith, Rhodes et al. 2008), and the trend for initiation of therapy at CD4 cell count > 350 cells/mm^3 for some subsets of patients (Panel on Antiretroviral Guidelines for Adults and Adolescents 2009), it is likely that a greater number of persons (than the 0.5 million estimated in 2003 by Teshale et al.) are eligible for and in need of ART nationwide today.

In 2007, the CDC launched the Medical Monitoring Project (MMP) based on a national probability sample of HIV-positive persons receiving care in the United States to obtain data on patterns of use and quality of HIV care and prevention services among such persons, and their clinical and virologic status (McNaghten, Wolfe et al. 2007). Preliminary data from the MMP project are expected in 2010. Available data on current patterns of HIV care, ART treatment and ART adherence come primarily from a few large U.S. HIV cohorts (Palella, Delaney et al. 1998; Lazo, Gange et al. 2007; Mugavero, Lin et al. 2009). For instance, the CDC-funded HIV Outpatient Study (HOPS) has shown persistent reductions in mortality and hospitalizations due to increasing use of highly active antiretroviral therapy (HAART) over time (Palella, Delaney et al. 1998; Buchacz, Baker et al. 2008). Data from the HIV Cost and Services Utilization Study (HCSUS) (Cunningham, Markson et al. 2000) and the HIV Research Network (Gebo, JAIDS 2005) point to disparities in use of ART by sociodemographic characteristics (*e.g.*, race, gender and insurance status), some of which appear to persist to the present (Palella, Armon et al. 2008).

Linkage-to-care is required for HIV-positive persons to realize the benefits of both HIV care and prompt initiation of ART. Recently updated Infectious Disease Society of America (IDSA) guidelines for the management of persons with HIV emphasized the importance of linkage and retention in primary care (Aberg, Kaplan et al. 2009). Poor engagement in care has been found to be a predictor of higher mortality. Specifically, HIV-positive persons with poor retention in care have been found to have 50% higher mortality rates (Giordano, Gifford et al. 2007). Appropriate care includes determining stage of HIV disease through clinical evaluation and measurement of CD4 cell count and HIV ribonucleic acid (RNA) levels, other health maintenance interventions such as Pap smears for women, tuberculin skin testing, provision of drugs for prevention of opportunistic infections, health education, risk reduction, supportive counseling and ART for patients eligible for such treatment (Panel on Antiretroviral Guidelines for Adults and Adolescents 2009). However, data from multiple studies indicate both failure and delay in linkage-to-care (Shapiro, Morton et al. 1999; Giordano, Visnegarwala et al. 2005; Tobias, Cunningham et al. 2007). Nationally, various studies estimate that only 60-75% of persons are linked to HIV care within three to six months of receipt of HIV diagnosis (Torian, Wiewel et al. 2008; Reed, Hanson et al. 2009; Zetola, Bernstein et al. 2009).

In the supplement to HIV/AIDS surveillance (SHAS) project, analyses of interview data collected during 2000-2004 from over 20 U.S. cities, counties, and states, indicated that 72% of persons reported entering HIV care within three months of testing HIV-positive (Reed, Hanson et al. 2009). Barriers associated with failure or delayed entry into HIV care include structural, financial and personal/cultural factors. Such factors include first-time HIV testing and anonymous HIV testing (Reed, Hanson et al. 2009), longer waiting time for initial care appointment (Mugavero, Lin et al. 2007) or being diagnosed at earlier stage of HIV disease when patients report "feeling well" (Tobias, Cunningham et al. 2007). In a NYC study using 2003 HIV surveillance data, Torian et al. found that 64% of patients initiated HIV care within three months of HIV diagnosis, 19% initiated care more than three months after diagnosis, and 17% never initiated care. Delay in initiation of care was associated with HIV testing in a community site, in the correctional system, at a sexually transmitted infection or tuberculosis (TB) clinic, non-white race, injection drug use and foreign birth (Torian, Wiewel et al. 2008).

The national five-year multisite Outreach Initiative funded by Health Resources and Services Administration (HRSA) in 2001 identified a number of effective programmatic interventions for engaging and retaining HIV-positive persons in care, including education and outreach, strengthening of referrals, provision of linkage coordination and navigation services, and case management (Rajabiun, Mallinson et al. 2007; Tobias, Cunningham et al. 2007). For instance, an "HIV system navigation" approach was successful in reducing barriers to establishing care and improved health outcomes in a multisite study over 12-month period (Bradford, Coleman et al. 2007). The CDC-funded Antiretroviral Treatment Access Study (ARTAS) strengths-based case management intervention, delivered at CBOs and health departments in 10 sites across the United States during 2005-2006, resulted in 79% of recently diagnosed HIV-positive persons receiving HIV medical care within six months of enrolling in the study (Gardner, Metsch et al. 2005; Craw, Gardner et al. 2008).

1.1.3.1 Expected Rates of Viral Suppression

The epidemiology of HIV infection in the United States reveals a disproportionate percentage of African American (AA) people (41%) living with HIV compared to the general population (12%). Outcomes of virologic efficacy in all populations impacted by HIV in the United States have been assessed in studies to determine rates of success of such treatment and its effect on mortality and morbidity. An analysis from 1995 -2001 revealed that mortality rates for HIV-positive white men in the United States declined by 85% compared to 50% and 65% for HIV-positive AA women and men respectively (Prevention 2002). The latter finding may relate to factors such as late diagnosis of HIV, access to ART and challenges in achieving high rates of adherence with treatment.

A number of studies to evaluate these differences between race and virologic efficacy have been published. Weintrob et al. (Weintrob, Grandits et al. 2009) demonstrated significantly lower odds of obtaining virologic suppression in AA compared to Caucasians at six and 12 months in a cohort of military personnel. This group was studied because such individuals are likely to have similar access to medications and care. However, several factors may have still influenced this finding including adherence with ART, provider bias, or differences in education status impacting selection bias

regarding provider selection of initial therapy, or how long patients stay on a given regimen. Anastos et al. (Anastos, Schneider et al. 2005) reported similar findings in terms of rates of viral suppression in 961 HIV-infected women enrolled in the Women's Interagency HIV Study (WIHS). AA women were 30% less likely to achieve viral suppression and were also 30% more likely to rebound after achieving viral suppression. The reasons for these differences remains unclear, as subsequent adjustment for multiple variables including biologic and behavioral variables did not eliminate the differences noted by race. There have been other studies showing genetically determined factors that influence drug levels, but studies that link these same factors to virologic success or toxicity have been inconclusive.

Thus, evidence suggests that, in addition to the disproportionate impact of HIV on racial/ethnic minorities in the United States, there are racial disparities in terms of outcomes of HIV disease and these persist in HIV treatment. It is unclear if this disparity in outcomes points toward race as a biologic determinant or as an underlying complex sociologic marker. Nonetheless, the selection of measure of virologic success at an HIV RNA level of < 400 copies/mL is a reasonable measurement that should reflect success of ART in individuals irrespective of racial group.

1.1.3.2 Access to Support Services for Mental Health, Substance Use and Homelessness

Linkage to HIV care is critical for those infected with HIV; however, underlying conditions, such as substance use (Robison, Westfall et al. 2008; Wood, Kerr et al. 2008; Applebaum, Reilly et al. 2009; Norman, Basso et al. 2009), mental illness (Berg, Cooperman et al. 2009; Kapetanovic, Christensen et al. 2009; Roux, Carrieri et al. 2009), and homelessness (Royal, Kidder et al. 2009) can have significant impact on a person's ability to become successfully linked to HIV care, as well as to adhere to ART. Depression and anxiety are the most common psychiatric diagnoses in HIV-positive persons and are 5-10 times more common in this population than in the general population (Pence 2009). A limited number of randomized clinical trials have demonstrated the beneficial effect of various psychotherapy-based interventions on ART adherence (Weber, Christen et al. 2004; Wyatt, Longshore et al. 2004; Safren, Knauz et al. 2006). In addition, substance use may impact side effects of ART. For example, Cheng et al. noted that subjects with high alcohol use had higher odds of experiencing lipodystrophy (odds = 2.07, adjusted odds ratio = 0.90, 95% confidence interval = 4.73) compared to those with lower alcohol use (Cheng, Libman et al. 2009). It is estimated that at least 13% of HIV patients have mental health and substance use disorders (Weaver, Conover et al. 2009).

Access to supportive services such as substance use management, mental health, homelessness prevention and adherence support are some of the key components in comprehensive HIV programs. Such supportive services are available in both of the intervention cities. Study test sites will maintain the ability to refer clients with urgent needs (e.g., mental health) for necessary services. However, test sites will not routinely link patients to support services. The DOHs in both intervention communities actively encourage a *single* referral of HIV-positive patients from test sites to care sites. Multiple referrals can be overwhelming to newly HIV-diagnosed patients and may give the wrong impression that an individual must be drug-free in order to engage in HIV care, and may

paradoxically delay entry into care. Additionally, testing sites usually do not have ongoing relationships with persons in need of support services, and are thus not well suited for evaluating sustained linkages to these resources or adequacy of services to meet patient needs.

Participating HIV care sites will serve as the comprehensive 'medical home' for coordination of all HIV-positive patient care needs, including substance use, mental health, and other support services. As newly diagnosed HIV-positive patients present for care, care sites will evaluate CD4 cell count and VL. Care sites will then create an appropriate medical plan for each patient, consistent with current HIV care and treatment guidelines. Prior to study implementation, all HIV care site staff will be trained on the importance of linking HIV-positive patients to appropriate support services.

1.1.3.3 HIV Care and ART Utilization in Washington, D.C.

Historically, Washington, D.C., like several other jurisdictions, has had limited success in ensuring that clients newly diagnosed with HIV are routinely and rapidly linked to a primary HIV "medical home." In 2006, before the HIV testing scale up, only approximately 50% of persons newly diagnosed with HIV were linked to care, as evidenced by a baseline CD4 cell count within six months of diagnosis reported to HIV surveillance. In contrast, in 2007, approximately 67% of the newly diagnosed HIV cases had laboratory evidence (CD4 cell count or VL) of a first HIV-related medical care visit within six months of the initial diagnosis.

In 2008, Washington, D.C. began to look beyond initial linkage-to-care to continuum-of-care after diagnosis. The number of HIV/AIDS cases reported between 2004 and 2007 increased 20.3% from 1,239 cases to 1,490 cases; data from 2008 are still preliminary but include 1,198 reports to date. Sixty-seven percent (67%) of cases had a first CD4 cell count, percentage or VL reported within three months of HIV diagnosis in 2008. The median CD4 cell count among newly diagnosed cases increased 57% from 216 cells/µl in 2004 to 340 cells/µl in 2008. Among newly diagnosed AIDS cases, the proportion of late testers decreased from 66% in 2004 to 57% in 2008.

Washington, D.C. supports full access to HIV care and treatment for all HIV-positive residents through a combination of regular Medicaid/Medicare, the Medicaid 1115 waiver, and Ryan White-funded services, including the AIDS Drug Assistance Program (ADAP). However, despite availability of services, use and outcomes of HIV care in Washington, D.C. remain sub-optimal. Preliminary review of ADAP data suggests irregular utilization at client level, with approximately 60% of enrollees picking up medications in a given month. Moreover, a preliminary review of Ryan White-funded, primary HIV-care providers suggests that only between 25-60% of clients in care are on ART, with approximately 50% of those being virally suppressed (unpublished data from ADAP and grantee reports).

Washington, D.C. is starting to address sub-optimal utilization and outcomes through a number of initiatives. First, a mass media information campaign promoting the availability of treatment was instrumental in increasing ADAP enrollment over 50% within 18 months. Since 2007, the HIV/AIDS, Hepatitis, STD and TB administration (HAHSTA) has also conducted treatment promotion through the "It's Free to Treat Your

HIV" campaign, which is currently being retooled with patient components (emphasizing treatment enrollment and retention) and a provider toolkit (improving patient linkages and outcomes). Second, Washington, D.C. is collaborating with CDC to identify ways to better utilize routine ADAP pharmacy data to identify irregular utilization and build rapid feedback loops to providers. Third, the Ryan White Planning Council and HAHSTA have adopted a "health first" approach, making navigation (for linkage-to-care from positive test), re-capture (bringing persons lost to follow-up back into HIV care), and retention core priorities for all new funding opportunities for 2009-2012.

HIV care in Washington, D.C. is supported by a range of funding sources, to ensure that every individual in need of HIV treatment is provided both primary care and the supportive services necessary to remain in care. HAHSTA directly supports primary medical care programs at nine organizations in Washington, D.C. These primary medical care programs serve as a hub for services, with the responsibility of coordinating the services needed by their clients. In 2008 HAHSTA began implementing a best practice, four Rs (recruitment, recapture, retaining and results) approach to improving the continuum and continuity of HIV care in Washington, D.C. Recruitment or rapid entry to care links individuals who receive a positive HIV test immediately into primary care facilities, and includes special programs, such as: 1) Red Carpet Entry to Care services; 2) adult and adolescent healthcare navigation services; and 3) Rapid Entry to Care initiatives. To recapture patients into care, HAHSTA partnered with several funded care providers over three months to bring back into care nearly 900 people known to be living with HIV but found to not have had a CD4 cell count or VL test in the previous six months. For example, Family Medical Counseling Services re-established care with approximately 70% of 450 clients identified as lost to care, often after more than 10 contacts. Finally, retaining people living with HIV into care and ensuring quality results is a critical component of ensuring the continuity and continuum of care. HAHSTA has supported best-practice, outcome-based management of HIV-positive clients in care with affiliated primary care providers. HAHSTA has partnered with funded providers to use E-clinical Works and other electronic medical record (EMR) systems to institute evidence-based best practices such as deploying coordinated care teams, using focused, comprehensive health messaging, and identifying outcomes at every clinical encounter, with the goals of reducing missed medical appointments, increasing efficiency, increasing prophylaxis and increasing viral suppression.

Another specific strategy to increase linkage-to-care in Washington, D.C. is the requirement that all individuals with preliminary positive rapid HIV tests be linked immediately into care. When confirmatory testing is needed, it is performed at the initial medical visit, along with CD4 cell count, VL and other routine tests for HIV-positive individuals.

Most recently, HAHSTA funded Navigator Services, consisting of an Adult Navigator and an Adolescent Navigator, to facilitate the linkage of HIV-positive individuals into care and treatment. This new citywide resource supports the efforts of independent clinicians who have historically diagnosed up to 40% of our new HIV-positive persons. Navigator services provide individualized support to link newly diagnosed and previously identified positives not in care to a medical home for ongoing specialized HIV care and treatment, as well as evaluate these clients for any other social service needs.

1.1.3.4 HIV Care and ART Utilization in the Bronx, New York

Recent data evaluating linkage-to-care in NYC generally, and the Bronx specifically, have shown slightly more robust linkage rates than in Washington, D.C. However, retention in care is comparable. Most recent surveillance data for NYC show that approximately 70% of newly diagnosed persons have laboratory evidence of a first HIV-related medical care visit (demonstrating linkage-to-care) within three months of their initial diagnosis of HIV. The linkage rate in the Bronx is equivalent to that citywide. Continuity of care, defined by evidence of a medical visit (CD4 cell count or VL reported in the HIV/AID Registry) at least every six months, was 54.4% for NYC overall and 49.3% in the Bronx. Among persons initiating care within three months of HIV (non-AIDS) diagnosis, median CD4 cell count was 439 cells/mm^3 citywide and 451 cells/mm^3 in the Bronx. Finally, 69% of persons eligible under Department of Health and Human Services (DHHS) guidelines to receive ART citywide (CD4<350), and 70% of eligible persons in the Bronx, had achieved an undetectable VL within a median of six months after initiation of care.

HIV-related treatment and economic services are widely available for all HIV-positive New Yorkers, regardless of means. A combination of Medicaid/Medicare services, Ryan White-funded services, including the ADAP, Housing Opportunities for People with AIDS (HOPWA), and benefits available through the HIV/AIDS Services Administration of NYC's Human Resources Administration ensure broad coverage to meet treatment and social service needs. Despite this network of support, outcomes of HIV care for New Yorkers including Bronx residents, as described above, indicate some potential for gain.

NYC has undertaken at least two major initiatives to attempt to make headway in these areas. Beginning in December 2009, the NYC DOH will begin funding 27 agencies throughout NYC, including six agencies in the Bronx, for comprehensive coordination of care using Ryan White funds. Coordination of care will include health system navigation, medical case management with treatment adherence, and educational coaching with goals of viral suppression and self-sufficiency for individual patients. Additionally, an expanding Field Services Unit (FSU), created in 2006, stations public health advisors on site at ten tertiary hospitals in the highest prevalence neighborhoods of NYC, as well as at Rikers Island jail, a large correctional facility. FSU staff assists index patients and physicians with partner elicitation, partner notification and testing of partners. FSU staff also work to recapture patients who have fallen out of medical care.

1.1.4 Adherence to ART and Viral Suppression in the United States, Washington, D.C. and the Bronx

Non-adherence to ART is common among patients in the United States, with the percentage of prescribed doses taken estimated between 60% to 70% (Simoni, Pearson et al. 2006). Somewhat higher adherence levels are reported over shorter recall periods (Mugavero, Ostermann et al. 2006; Lazo, Gange et al. 2007). However, levels of adherence to ART as high as 80% to 95%, depending on the type of regimen, may be necessary to achieve and maintain maximal viral suppression and optimize clinical outcomes (Bangsberg 2008). Incomplete adherence has been among the most important factors related to virologic failure, the emergence of drug resistance, and ultimately

progression of HIV disease to AIDS and death (Lazo, Gange et al. 2007; Lima, Geller et al. 2007; Horberg, Silverberg et al. 2008). Non-adherence also contributes to the transmission of drug-resistant HIV strains (Sethi, Celentano et al. 2003).

Recently introduced ART regimens are less toxic, more tolerable and simpler, particularly if available in fixed-dose combinations (ARV treatment guidelines). These include ART regimens with new agents from established classes and new classes of ART that have been recently shown to be effective in suppression of resistant viral strains (Steigbigel, Cooper et al. 2008; Markowitz, Nguyen et al. 2009). Multiple clinical trials have shown the ability to achieve suppressed viral replication with the use of such regimens in a large proportion of patients. However, suboptimal adherence remains common, and young age, active drug and alcohol use, and depression are associated with poor adherence to ART (Levine, Hinkin et al. 2005; Lazo, Gange et al. 2007; Horberg, Silverberg et al. 2008). In clinical practice, adherence is typically assessed by self-report (Simoni, Kurth et al. 2006) and can be corroborated by the use of MEMS caps, pill counts, biologic markers (*e.g.*, plasma drug levels), pharmacy refill data, (Bangsberg 2008) and ultimately by the measurement of HIV VL.

In addition to optimizing ART regimens, effective approaches to improving ART adherence include ART-readiness training, adherence-related case management, various forms of counseling, pharmacist-based support, telephone support, reminder devices, and directly observed therapy (Bartlett 2002; Bangsberg 2008). A recent meta-analysis of randomized behavioral interventions conducted mostly in the United States found that participants receiving adherence interventions were 1.5 times as likely to report 95% adherence and 1.25 times as likely to achieve undetectable VL than controls (Simoni, Pearson et al. 2006). Improving ART adherence requires a combination of methods appropriate to the patient and clinical setting. Among the most important predictors of ART adherence are engagement in medical care and continuous adherence with medical visits (Aberg, Kaplan et al. 2009). Alterable factors known to impact adherence, such as mental health problems, active substance use, homelessness, inadequate level of social/economic support (Magnus, Kuo et al. 2009), as well as the therapeutic relationship between patient and provider should be addressed proactively (Bakken, Holzemer et al. 2000; Bangsberg 2008).

Both in the Bronx, NY and Washington, D.C., the health departments consider adherence support services to be an essential component of a comprehensive HIV-care package. These health departments monitor trends in adherence by reviewing HIV VLs and CD4 cell count data reported to HIV surveillance systems. For example, HAHSTA approaches the promotion of treatment adherence as a responsibility shared by all providers or services including physicians, physician assistants, nurses, pharmacists and other clinical care providers. Upon enrollment, and periodically thereafter, each client's records are reviewed for evidence of viral suppression as well as assessed for the need for specialized services to improve the ability of the client to take HIV medications effectively and remain consistently in care. A wide range of services is available for those in need, including linkages to mental health and substance use programs, education on HIV, programs designed to assist with the necessities of daily living, and ongoing individual or group psychosocial and peer support.

1.1.5 Prevention for Positives

Meta-analyses show that prevention efforts in HIV-positive individuals (Prevention for Positives) (Crepaz, Lyles et al. 2006; Johnson, Carey et al. 2006), can be effective in decreasing risky behaviors. However, most Prevention for Positives interventions have been assessed only in research contexts and require substantial investments of staff and resources to deliver with fidelity, suggesting that population-level uptake may not likely follow. Sexual risk assessments and risk-reduction counseling are not routinely performed in many HIV clinical settings, and even when they are, quality and consistency are variable (Metsch, Pereyra et al. 2004). Information and communication technologies (ICT) may facilitate scale-up of prevention interventions, as they utilize the client's time for self-monitoring, deliver content with fidelity, can include multiple languages, and, once programmed, can be used for multiple computers with marginal cost beyond cost of the hardware. A recent meta-analysis found that these tools are efficacious for reducing HIV transmission risk (Noar, Black et al. 2009). A recent study by Gerbert et al. found that computer-delivered "video doctor" counseling to 960 HIV-positive patients was associated with reduced transmission risk behavior (Gilbert, Ciccarone et al. 2008). CARE+ Prevention is one example of an intervention that may be effective in decreasing sexual behaviors most likely to transmit HIV. Other studies have shown that Prevention for Positives interventions are less effective with patients with ongoing substance use and mental health conditions.

In Washington, D.C. HAHSTA is in the process of developing a Prevention for Positives initiative that includes consistent and repeated delivery of prevention interventions by clinicians to people living with HIV/AIDS (PLWHA) in care and treatment settings. The strengths of this clinic-based approach include linking the prevention of HIV transmission to the treatment of HIV, offering repeated opportunities to intervene in high-risk behavior, and serving as a source of information, support and linkage to needed services for the HIV-positive individual. The five areas of focus for this initiative will be: 1) social support for disclosure; 2) treatment adherence for viral suppression; 3) mental health; 4) substance use; and 5) general prevention interventions, such as distribution of condoms.

The NYC DOHMH has begun a concerted effort to prioritize prevention activities with HIV-positive individuals. Beginning in 2007, the department began funding 19 agencies to conduct 16 different behavioral interventions that have clear evidence of achieving behavior change. While many of these interventions work with HIV-positive and high-risk HIV-negative individuals, six agencies were funded to conduct interventions that focus primarily on HIV-positive individuals, such as "Healthy Relationships." Partner services activities as described above, operating primarily through the FSU and the Contact Notification Assistance Program, also focus directly on HIV-positive individuals as well as soliciting, notifying and testing partners of index patients. As part of their routine work, FSU staff members also assist in linkage and health navigation activities, discuss risk reduction, and provide free condoms, along with condom education as needed.

In 2009, the NYC DOHMH introduced a CDC-developed training course for NYC healthcare providers focusing exclusively on HIV prevention for positive patients called

"Ask, Screen, Intervene." The course was offered quarterly and will continue in 2010. The Department further developed a comprehensive prevention-for-positives protocol for clinicians and non-clinician CBO staff, which will be made available in 2010. Finally, both the Ryan White comprehensive coordination of care program and a planned new funding stream will increase dedicated prevention activities for HIV-positive persons beginning in 2010.

1.1.6 Provider and Patient Attitudes Towards ART Use

Mathematical models that have addressed the issue of use of ART for prevention of HIV transmission have largely concluded that ART would be most effective for HIV prevention if used in the largest proportion of HIV-positive populations (Granich, Gilks et al. 2009). This implies that such treatment would be provided to HIV-positive individuals irrespective of their eligibility for ART initiation, as detailed in the available national or international guidelines. To date, such guidelines (Health and Human Services (HHS) guidelines, WHO ART guidelines) have been largely based on evidence supporting benefits versus risks of the use of ART for the individual with HIV infection, rather than based on prevention considerations.

Providers have largely utilized such recommendations to guide their decisions for when to initiate or use ART in HIV-positive individuals, aiming to provide patients in their care with the opportunity to achieve optimal outcomes for their individual health and well-being. Use of ART in HIV-positive individuals to benefit their partners and the community is a concept that goes beyond this framework and places providers in a dilemma, should the well-being of their patients be affected adversely by therapy whose aim is to reduce potential transmission to others in the community.

Similarly, from the patient's perspective, ART use has been largely perceived to provide individual benefits. Thus, in making decisions regarding initiation of ART, patients make their assessment based on information provided to them regarding benefits and risks to their own health rather than based on potential effect on transmission of HIV to others. Adding reduction in infectiousness as a consideration will need to be weighed and patient knowledge and attitudes regarding such use carefully evaluated.

1.1.7 Financial Incentives

The use of financial incentives (FIs) to modify behaviors has become increasingly common in settings both within and outside the healthcare sector. Outside the healthcare setting, experiments have ranged from conditional cash transfers to alleviate poverty among low socio-economic status (SES) individuals in NYC to efforts to improve school performance within NYC, Chicago and Washington, D.C. Within the healthcare setting, in recent years, there has been increasingly widespread use of FIs among insurers and employers who see this as an approach with great promise to help individuals make better tradeoffs between unhealthy behaviors (that have immediate gratification) in favor of healthier behaviors (that have delayed benefits). For example, NYC is evaluating the effectiveness of FIs in achieving normal HbA1c levels in individuals with diabetes. In fact, such efforts have now been shown to be effective in changing health behavior and improving health outcomes in a variety of clinical contexts.

The interest in interventions aimed at improving patient behaviors is in part due to recognition that unhealthy behaviors may be a bigger contributor to poor health and premature mortality than inadequate healthcare delivery. Experts have estimated that unhealthy behaviors, such as medication non-adherence, smoking, poor diet and sedentary lifestyles account for as much as 40% of premature mortality in the United States, whereas deficiencies in healthcare delivery account for only 10% of premature mortality (Schroeder 2007). Many factors such as the social and structural environment, public policies, genetics and provider access and quality affect the rate of such behaviors. However, individuals' behavioral choices are clearly a central driver and are potentially more amenable to incentives.

Early evidence on incentives suggests that incentive-based approaches can be highly effective in two areas in particular: (1) changing short-term health behaviors related to preventive services that involve a limited number of visits, and (2) reducing the use of addictive substances. Examples of effectiveness of incentives in increasing use of preventive services include studies that have shown increases in rates of follow-up for abnormal pap smears (Marcus, Kaplan et al. 1998), postpartum visits by adolescents (Stevens-Simon, O'Connor et al. 1994), TB test reading (Malotte, Rhodes et al. 1998), and the rate at which IV drug users received all three doses of hepatitis B vaccine (Seal, Kral et al. 2003). The evidence that such approaches are effective in reducing the rate of use of addictive substances, such as cocaine (Higgins and Silverman 1999; Lussier, Heil et al. 2006) and nicotine in the short term, (Donatelle, Hudson et al. 2004; Volpp, Gurmankin Levy et al. 2006), as well as for short-term weight loss (Jeffery, Thompson et al. 1978; Jeffery, Gerber et al. 1983; Finkelstein, Linnan et al. 2007), suggests that financial rewards designed to incent long-term changes in behavior could be applicable to a wide range of other health behaviors in which frequent reinforcement and longitudinal follow-up are necessary.

However, evidence of effectiveness of incentives is not limited to the areas cited above. A review of 11 randomized trials of FIs found that in 10 studies, FIs promoted adherence better than any tested alternative, leading to better blood pressure control, better appointment attendance and higher immunization rates (Giuffrida and Torgerson 1997). More recent reviews of economic incentives found that a wide range of incentive mechanisms are effective in changing behavior (Kane, Johnson et al. 2004; Sutherland, Christianson et al. 2008).

Another reason to consider further testing of incentive-based approaches is that many highly efficacious medical tests, treatments and medications have limited effectiveness due to patient behaviors. For example, by one year after having a myocardial infarction, nearly half of patients prescribed cholesterol medications have stopped taking them (Jackevicius, Mamdani et al. 2002). Similarly, the effectiveness of HIV medications would be much higher if rates of adherence increased to the point where benefits demonstrated in clinical trials could also be seen in high rates of effectiveness in communities across the United States.

More recent research has found that FIs double the rate of attendance at and completion of a smoking-cessation program, triple long-term smoking cessation rates and substantially increase medication adherence among patients on warfarin. For example, in

a two-arm RCT of 878 employees at General Electric (GE), (Volpp, Troxel et al. 2009) incentives of up to $750 resulted in quit rates at nine-12 months triple those in the control group (p<.001). Other studies have examined the use of incentives for weight loss (Volpp, John et al. 2008) and medication adherence (Volpp, Loewenstein et al. 2008).

Two studies have been published in which FIs were used for ART adherence. In both cases, the payments made a significant improvement in ART adherence (Rigsby, Rosen et al. 2000; Rosen, Dieckhaus et al. 2007). The findings that FIs can help modify difficult-to-change behaviors whose cessation requires ongoing reinforcement, such as quitting alcohol or cocaine use, are particularly striking and suggest that a well-designed incentive program can succeed in changing behaviors in clinical contexts in which many other approaches have been unsuccessful (Bigelow and Silverman 1999; Donatelle, Prows et al. 2000; Higgins, Wong et al. 2000).

The amounts we selected for use as FI in this study are based on the following principles: 1) review of relevant literature and experiences, 2) input from investigators with knowledge of target communities and populations, 3) input from community groups/advisors from the study communities and elsewhere, and 4) input from key staff in departments of health and public health entities in the relevant communities. Based on these factors, the amount of $125 for successful linkage-to-care, and a maximum of $630 for viral suppression throughout the study period were thought to be appropriate amounts for evaluation in this study.

With regard to the sustainability of any effect caused by the FI after it is removed, a recent study on incentives for smoking cessation (Volpp, Troxel et al. 2009) demonstrated that the ratio of tobacco cessation among incentive to control group participants at nine-12 months (2.9) remained significant six months after cessation of incentive payments (ratio of quit rates was 2.6 at 15-18 months). In other work, shorter durations of incentives have been associated with higher relapse rates. It is of interest that based on the findings of the latter study on smoking cessation, GE announced plans to implement a program for FIs for smoking cessation nationally for all its 152,000 employees.

Studies involving FIs have focused on both process measures (the behavior in question, such as medication adherence) and outcomes (smoking cessation). There are several precedents for the use of outcomes (*e.g.,* VL) to assess the effect of FIs on medication adherence. Several studies that used FIs to improve adherence with medication (*e.g.,* warfarin or insulin) have measured biologic outcomes to evaluate adherence (*e.g.,* INR, HbA1c) (Volpp, Loewenstein et al. 2008). It is essential that FIs be provided in such a way that they yield a verifiable outcome. Because lack of VL suppression most often results from lack of adherence, the use of FIs to encourage ART adherence is plausible from both a medical and a behavioral-economics perspective. The relative effectiveness of focusing incentives on process versus outcomes is generally unknown and is ultimately an empirical question. In Washington, D.C., a demonstration project indicated that a focus on outcomes (rather than process) has been associated with improvements in the areas of HIV prevention and care (Hadar, personal communications). Specifically, rewarding HIV-care organizations for every three patients they successfully re-linked to care, regardless of the effort expended, yielded positive results. In this study, FIs will

focus on outcomes – the completion of care visits for the Linkage-to-Care component and in the Viral Suppression component.

In contingency management studies, non-monetary rewards have often been used in place of monetary rewards because of concerns that cash might increase the likelihood of substance use. However, studies have not supported such concern. In the AIDS Link to Intravenous Experience (ALIVE) study, ethnographers trailed the drug users after they received the money to assess where they went. They found that participants mostly went to food establishments after they received the money. There was no difference in the rates of death caused by overdose in participants when analyzed by days after study visit or amount of reimbursement (Vlahov, Tang et al. 2000).

1.1.8 HIV Surveillance Data

Since 1982, all 50 U.S. states and Washington, D.C. have reported AIDS cases to the CDC using a standardized case report form (CRF) (Schneider, Whitmore et al. 2008). In 1994, the CDC integrated national reporting of HIV with AIDS case reporting, at which time 25 states with confidential, name-based HIV reporting started submitting case reports to the CDC. Over time, additional states implemented name-based HIV reporting and started reporting these cases to CDC. By April 2008, all states and Washington, D.C. had implemented name-based HIV reporting and were reporting cases to the CDC.

HIV case surveillance data are collected as part of routine HIV surveillance, as mandated by state or local laws or regulations. Named reporting is required for all diagnoses of HIV and AIDS and all HIV-related illness according to the case definition. Information is collected on demographic characteristics of persons diagnosed with HIV, transmission risk factors, facility of diagnosis, diagnostic tests (*e.g.*, positive Western Blot (WB) tests for HIV antibody) and death. All areas require laboratory reporting of VL and CD4 cell count values. While not all areas require reporting of all values of VL or CD4 cell count (some limit reporting to detectable VL and/or CD4 <200 or <14%), all areas collect the first results of such tests after HIV diagnosis through chart review or laboratory reporting, where all values are reported through voluntary laboratory reporting. Both intervention communities require reporting of all laboratory results. Some areas such as NY also require reporting of all HIV genotypes.

These population-based registries are continuously updated with new, de-duplicated diagnoses and laboratory results. Incoming diagnostic WB and VL reports from providers and laboratories that cannot be matched to an existing registry record initiate a field investigation to confirm the case, date and disposition of diagnosis and collect other data required for surveillance. Data are also obtained through matches with other disease registries, the state and local death registries, the National Death Index and the Social Security Death Master File. Assessments of duplicate cases occur both on the state and national level (potential duplicates are identified based on soundex code [a phonetic algorithm for indexing names by sound, as pronounced in English] and selected demographic characteristics), while elimination of such cases occurs at the state level.

HIV reporting and laboratory reporting requirements allow virtually complete surveillance of diagnoses of HIV, stage of disease at HIV diagnosis, and number of

people diagnosed and presumed to be living with HIV. Using CD4 cell count and VL test ordering as proxy measures for initiating HIV primary care, after the first positive WB test, allows for calculation of the time between diagnosis and initiation of care. Frequency of visits, regularity of U.S. DHHS-recommended laboratory monitoring, and estimates of the proportion of cases eligible for ART are now possible with CD4 cell count and VL result reports. These laboratory indicators also allow estimates of the number and characteristics of cases not in care. Clusters of highly resistant HIV will be detectable when state genotype reporting systems become operational.

The CDC has developed a data entry and reporting system, the Program Evaluation and Monitoring System (PEMS), to strengthen monitoring and evaluation of HIV-prevention programs. PEMS is used by health departments and CBOs funded through CDC HIV-prevention cooperative agreements. PEMS allows grantees to collect agency data, community planning data, program plan data, and client-level data. This assures a comprehensive set of standardized variables are available.

Some areas have implemented additional surveillance activities, such as behavioral risk factor surveys and supplemental surveillance activities supported by the CDC (*e.g.*, incidence surveillance). The National HIV Behavioral Surveillance (NHBS), which takes place in 25 cities, can provide information on testing among various population groups including among MSM, a special emphasis group in this project. Where available, the Morbidity Monitoring Project (MMP, a 26-site study) may provide information on treatment among those in care. In addition, data are collected on testing conducted in all sites receiving funding from the CDC and in additional sites as required by state or local laws or regulations.

1.2 Purpose

The main purpose of this study is to assess the feasibility of a community-level test, link to care, plus treat strategy in the United States. The study will involve evaluation of the feasibility of some components and the effectiveness of others.

The study will assess the following:

- The feasibility of expanding HIV testing via social mobilization and universal offer of HIV testing in EDs and inpatient hospital admissions
- The feasibility and effectiveness of facilitating the linkage of HIV-positive patients to HIV care sites
- The feasibility and effectiveness of different strategies for assuring maximum initiation of ART for clinically eligible patients according to current guidelines
- The feasibility and effectiveness of different strategies for promoting high adherence to ART and maintenance of VL suppression
- The effectiveness of a computer-assisted program for Prevention for Positives
- Patient and provider attitudes towards the initiation of ART in early HIV disease

The primary outcomes of this study's package of interventions will be determined through measurement of change from baseline (at the initiation of the study) over the duration of the study (see study timeline) in key parameters in two intervention communities in the United States. Observations in four non-intervention communities will help assess the influence of current trends in HIV testing and care expansion in the United States.

This study will serve as a proof-of-concept, formative study. It will provide key information to guide the design and anticipate the costs of a future large, randomized, community-level clinical trial of full implementation of a test-and-treat strategy in the United States. This study uses innovative approaches including: 1) a community focus; 2) multi-component strategies that include behavioral and biomedical interventions; 3) the use of routinely reported HIV surveillance data to determine key outcomes; and 4) partnership with both local DOHs and the CDC. Findings from this study could also inform test-and-treat efforts in other developed countries with epidemics similar to that in the United States.

HPTN 065 will help generate estimates for some of the parameters that, taken together, will describe an "index of participation." Such an index of participation (upon which the test-and-treat strategy would ultimately depend) is based on a cascade: the percentage of HIV-infected persons tested and identified within a community, the percentage of such individuals linked-to-care, and the percentage who initiate and remain adherent to ART and maintain ongoing viral suppression. For example, even with optimistic assumptions (80% of infected persons tested; 80% linked to care, and 80% adherence to ART) the index of participation would be 51%. These parameters have, to date, been treated as assumptions in test-and-treat models. The mathematical model described by Granich et al. assumed that all adults would be tested annually with a 100% sensitive, 100% specific test; that all HIV-infected persons would enter care and start ART as soon as they were diagnosed irrespective of HIV disease stage; that there would be a decrease of 99% in their infectiousness and that only 1.5% of subjects would discontinue ART each year, for an unrealistic index of participation of 98.5% (Granich, Gilks et al. 2009).

HPTN 065 will examine the feasibility and relative effectiveness of various interventions that individually aim at optimizing each component related to the index of participation. These insights together with data on the magnitude of the effect of ART on infectiousness (to be obtained from other studies), could be incorporated into future models that aim to determine the potential effect of a test and treat strategy on incidence in various communities in the United States.

1.3 Rationale

1.3.1 Rationale for a Test-and-Treat Approach

More than one million persons in the United States are currently living with HIV and about 21% are unaware of their HIV-positive status. The HIV epidemic in the United States affects specific subpopulations and localized "hot spots." Individuals may be at risk for HIV because of their own risky behaviors or the high prevalence of HIV among

persons whom they encounter, or a combination of both. Risk may be compounded by high rates of undiagnosed HIV infection within these same communities.

In 2006, there were an estimated 56,000 new HIV infections in the United States; a number that has been approximately stable for the past decade (Hall, Song et al. 2008). Certain municipalities and communities in the United States bear the brunt of the HIV epidemic. This includes certain geographic areas in the United States and specific subsets of individuals defined by behavior and/or by race/ethnicity. The data from 2006 indicate that MSM and AAs are most severely affected. For example, in Washington, D.C., three percent of the population is HIV-positive. National Health and Nutrition Examination Survey (NHANES) data indicate that HIV prevalence among AAs age 40-49 is nearly 3% among women and 4.5% among men (McQuillan, Kruszon-Moran et al. 2006).

The central factors that drive the HIV epidemic in the United States are: (1) the number of individuals unaware of their HIV infection who continue behaviors likely to transmit HIV; (2) the frequency of late diagnosis of HIV infection; and (3) delay in access to care, delay in initiation of ART and suboptimal adherence, leading to failure of sustained viral suppression. Approximately 40% of persons are diagnosed with AIDS within a year of their first HIV-positive test (Valdiserri, Holtgrave et al. 1999; Castilla, Sobrino et al. 2002; (CDC) 2009), too late to realize the full benefit of advances in HIV management. Persons with undiagnosed HIV infection may also unwittingly transmit HIV to partners. While they represent less than one quarter of the entire HIV-infected population, persons with undiagnosed HIV infection account for more than 50-70% of new sexually transmitted infections in the United States and are 2.5 times more likely to transmit HIV than persons who are aware they are infected (Marks, Crepaz et al. 2006). Evidence also indicates that many diagnosed individuals delay or fail to engage in HIV care (Torian, Wiewel et al. 2008), and for those who have initiated ART, retention in care and adherence to ART is suboptimal (Lazo, Gange et al. 2007; Mugavero, Lin et al. 2009).

Since the beginning of this decade, evidence has accumulated that higher plasma HIV RNA is associated with increased risk of HIV transmission (Quinn, Wawer et al. 2000; Fideli, Allen et al. 2001). More recent data suggest that use of ART is associated with a substantial decrease in rate of HIV transmission from the HIV-infected to uninfected sexual partner (Bunnell, Ekwaru et al. 2006; Sullivan, Kayitenkore et al. 2009). The availability of various ART regimens capable of suppressing viral replication in various subsets of HIV-positive individuals offers the opportunity to determine the potential effectiveness of use of ART on HIV incidence in a community. While various models suggest that such a strategy may be associated with a successful impact on the trajectory of the HIV epidemic, empiric data are needed to confirm this hypothesis.

It is widely anticipated that if a TNT approach is to achieve its desired impact, it would require the following ambitious effort: universal HIV testing efforts; prompt and effective linkage of all individuals with HIV infection to HIV care; timely initiation of ART; and sustained suppression of HIV replication in all HIV-positive individuals. It is evident that assessment of such an intervention requires a deeper understanding of each of its components.

Thus, we will examine five study components highly relevant to the TNT approach. Four study components are interventions and one is a survey. The four study interventions to be assessed include: expanded HIV testing, linkage from HIV testing sites to HIV care sites, viral suppression with use of ART, and a computer-delivered intervention to achieve safer behaviors among HIV-positive individuals. In addition, surveys for both HIV-positive persons and HIV-care providers will be done to determine knowledge and attitudes regarding early use of ART and financial incentives.

1.3.2 Rationale for Choice of Intervention Communities

This study, Test, Link-to-Care, Plus Treat, will assess five components in two communities in the United States, the Bronx, NY and Washington, D.C. These communities were selected due to: (1) the severe impact of the epidemic on these communities (NYC has the largest number of cases of HIV of all cities in the United States and Washington, D.C. has the highest HIV seroprevalence), and (2) the substantial efforts these cities have already made to improve HIV diagnosis, facilitate linkage-to-care, and support adherence.

NYC has the oldest, largest and most heterogeneous epidemic in the Western World. By June 30, 2008, a cumulative total of 207,687 persons in NYC had been diagnosed and reported with HIV. 43,344 (20.8%) of these persons were residents of the Bronx at the time of diagnosis. A cumulative total of 100,378 persons with HIV have died over the course of the epidemic (48.3% of cumulative diagnoses). There have been 20,052 deaths (19.9% of cumulative deaths citywide) among Bronx residents. In 2007 HIV was the third leading cause of death in persons under age 65.

As of June 30, 2008, there were 104,234 persons diagnosed, reported and living with HIV in NYC. 22,479 (21.6%) were residents of the Bronx. Citywide, the rate of new HIV diagnoses was 46.8/100,000 population. The diagnosis rate in the Bronx was 62.4/100,000 population. The Bronx rate was second to that of Manhattan, which reported a diagnosis rate of 70.5/100,000 population. Citywide, 26.1% of newly diagnosed persons have already progressed to AIDS at the time they first learn they are infected with HIV (concurrent diagnosis of HIV and AIDS). The borough with the highest proportion of concurrent diagnoses (27.3%) is the Bronx.

Washington, D.C. is in the midst of a generalized HIV epidemic. At the end of December, 2007, there were 15,120 persons diagnosed and reported living with HIV in Washington, D.C. Between 2003 and 2007, there were 7,432 new HIV/AIDS cases reported, bringing to more than 3% the proportion of Washington, D.C.'s adult population diagnosed and living with HIV. It is estimated that one-third to one-half of infected persons may be unaware of their HIV infection (DC NHBS data). All wards in Washington, D.C. (with the exception of Ward 3) have prevalence rates above the Joint United Nations Programme on HIV/AIDS (UNAIDS)/WHO threshold for a generalized HIV epidemic. More than two-thirds of new HIV diagnoses are in people over age 30 years. More than 7% of Washington, D.C. residents age 40-49 and 5.2% of D.C. residents age 50-59 years are diagnosed and living with HIV. The rate of HIV is highest among black males (6.5%), but 3.0% of Hispanic males and 2.5% of white males and black females are estimated to be living with HIV. In examining the most recent trend

data on HIV (not AIDS) and newly reported AIDS cases, mode of transmission trends are changing. Heterosexuals account for over 37% of newly reported HIV cases and 31.5% of newly reported AIDS cases, followed by MSM (25.8% and 29.0%, respectively) and injection drug users (13.2% and 21.7% respectively). With the advent of ART, deaths due to AIDS have significantly decreased over the last 15 years. HIV/AIDS, however, is still the leading cause of death in Washington, D.C. residents age 25-44 (Washington 2009). In 2007, there were 138 deaths from AIDS or AIDS-related complications.

1.3.3 Rationale for Choice of Interventions

The study will focus on expansion of HIV testing in hospital emergency departments and inpatient units. The rationale for expanding the offering of HIV screening in EDs emanates from numerous studies in urban EDs that indicate a 0.7% - 1% yield of new HIV diagnoses among those tested. Many patients with HIV infection visit EDs, but remain undiagnosed. For example, of all patients with new HIV diagnoses in South Carolina from 2001-2005, 73% had previously visited healthcare facilities, but were not tested for HIV; 79% of these visits were to an ED. Although many EDs in both the intervention and non-intervention cities have initiated HIV screening, most ED programs are unable to offer screening 24/7, and many are unable to test all patients who agree to be tested.

A similar yield of new HIV diagnoses might be expected from testing hospital admissions. Data from the NY Health and Hospitals Corporation (the organization that operates 11 public hospitals in NYC where expanded HIV screening began in 2006), and from Boston Medical Center's HIV Inpatient Testing Service, indicated an HIV positivity rate of 2.5% - 5% among inpatients who agreed to be tested (selectively thus which may or may not reflect the prevalence of HIV infection among all inpatients, including those who refused HIV testing or were not offered testing). No data exist on the yield of routine HIV screening of all hospital admissions. This approach was traditionally a significant component of syphilis screening efforts. Similarly, this approach may be a cost-effective way to expand HIV screening and to ensure that no HIV-positive patient who encounters the healthcare system remains undiagnosed. This component of the HPTN 065 study (*i.e.* supporting expanded ED- and hospital-based offering of testing) will be a supplement to ongoing testing efforts in these communities.

The study will also aim to use social mobilization to increase HIV testing volume and frequency. Because of the bimodal nature of the HIV epidemic in the Bronx and in Washington, D.C. (generalized, especially among AAs, and concentrated among MSM) the study will also include another strategy in the testing component. Through social mobilization, focused messages will be created for the intervention communities on the importance of getting tested for HIV and knowing one's status (tested in past year). In addition, specific messages will be created to promote more frequent retesting for sexually active MSM—preferably twice a year, but at a minimum, annually.

In this study, we will not aim to identify acute infections. Unpublished data from the CDC's Acute HIV Infection Study demonstrate that HIV retesting as described above is considerably more cost-effective than Nucleic Acid Amplification Testing (NAAT) screening for acute infection.

The study will utilize FIs to promote linkage of HIV-positive individuals from testing to care sites and to achieve and maintain viral suppression. As described in Section 1.1.7 FIs hold great promise for achieving the target behaviors. The study will use a site-randomized approach to compare the effectiveness of FIs to the standard of care (SOC) in achieving these two goals.

Some TNT models assume that all HIV-positive individuals will initiate ART irrespective of their CD4 cell count. Evidence from observational studies supports earlier initiation of ART, with the goal of delaying HIV disease progression and reducing mortality rates (Kitahata, Gange et al. 2009; Sterne, May et al. 2009; When to Start Consortium 2009). The availability of safer, simpler, more tolerable and potent ART regimens has reduced, but not eliminated, some concerns about the development of long-term toxicities and complications among HIV-positive patients (Justice 2006; Friis-Moller, Reiss et al. 2007) and reduced concerns about development of antiretroviral resistance (Phillips, Leen et al. 2007; von Wyl, Yerly et al. 2009). However, in this study, the provision of ART will be guided by the prevailing DHHS Guidelines for the Use of ART in HIV-Positive Adults and Adolescents (Panel on Antiretroviral Guidelines for Adults and Adolescents 2009). Providers at participating sites will receive educational trainings on best practices in HIV management, per current guidelines, and will be encouraged to promptly and appropriately evaluate their patients for eligibility for ART, maximize ART initiation when indicated per current guidelines, and switch regimens when indicated.

Lastly, while TNT models have not directly addressed the contribution of efforts to promote safer behaviors to reduce transmission from HIV-positive persons, unsafe sex behaviors by HIV-positive persons are a major factor contributing to ongoing HIV transmission. Therefore, in this study, we will evaluate an individually randomized computer-delivered prevention-for-positives component to reduce unsafe sexual and injection-drug using behaviors. In this manner, we will gather feasibility and effectiveness data to inform about the value of such an intervention for future studies.

1.3.4 Rationale for Use of Surveillance and Routinely-collected Data

The study will utilize routinely reported HIV surveillance data collected in the United States to determine the effects of the various interventions. The United States is fortunate to have a robust HIV surveillance system that was established at the advent of the epidemic in the early 1980's. Over the ensuing years, this system has been expanded and refined. At present, in many communities, it includes data on all positive HIV tests, demographic and risk behavior data on all those found to be HIV-positive, collection of all CD4 cell count and HIV RNA results, and information on AIDS-related deaths, among other data elements. Thus, this system offers a remarkable opportunity to assess various site-level or community-level interventions.

The evaluation of each of the present study's components, and the feasibility of use of existing HIV surveillance data, will be critical to the design, implementation and evaluation of a large, future, community-focused test-and-treat study, with HIV incidence as the endpoint of interest. The collaborative process utilized in the development of this study that includes partnership among the National Institutes of Health (NIH)-funded

HPTN, the CDC, and departments of health is noteworthy. In addition, the engagement of diverse settings, and organizations, including HIV testing sites, HIV care sites, health facilities, providers, CBOs, advocates and leaders is critical to its success and necessary for future large studies of TNT strategies.

1.4 Protocol Structure

This protocol describes the five study components: 1) expansion of HIV testing, 2) enhancing the linkage of HIV-positive individuals to HIV care sites, 3) maintenance of VL suppression, 4) use of a computer-delivered intervention to decrease high-risk sex behaviors (Prevention for Positives), and 5) provider and patient surveys to gather data on knowledge, attitudes and practices regarding ART and ART adherence (potentially starting at higher CD4 cells counts) and FIs. Sections 2-6 are devoted to the objectives; study design, interventions, and procedures; data and statistical analysis; and ethical issues of each study component. Sections 7-8 contain information applicable to all study components (HIV surveillance data, data sources and administrative/operational issues).

2.0 EXPANDED HIV TESTING

2.1 Study Objectives for Expanded HIV Testing

The intervention communities have committed substantial programmatic funds to expanded testing. The study will aim for expansion of HIV testing efforts in the intervention communities building on ongoing efforts in these communities. The study will focus on two elements: 1) social mobilization and 2) universal offer of HIV testing and counseling in hospital EDs and inpatient units.

True evaluation of the efficacy of increased testing and its impact on detecting undiagnosed infections could only be achieved through a high-quality random sample, representative of the general population, to obtain information about HIV status and knowledge. The expense and scale of such an effort is beyond the scope of a feasibility study. Thus, we have adopted the planned ecological approach of monitoring process and outcome measures to capture increase in HIV testing and change in number and characteristics of newly detected infections throughout the course of the study.

The **primary feasibility objective**s for expanded HIV testing, through a combination of focused and enhanced social mobilization efforts, will be measured by assessing the following trends in the intervention cities:

- Volume of testing in publicly funded testing sites
- Number of newly identified HIV-positive individuals
- Site of diagnosis for newly identified individuals
- Initial CD4 cell count results after first positive HIV test (to assess proportion eligible for ART at different initiation thresholds)
- Proportion of newly identified individuals with HIV concurrently diagnosed with AIDS
- Proportion of population tested for HIV in the last year (local behavioral surveys)

Because expanded testing initiatives are already underway in numerous jurisdictions, the magnitude and trends of changes in the intervention cities will be compared to similar measures in the non-intervention cities, in an attempt to account for secular trends independent of the study components.

- The following variables in the intervention cities will be assessed:
    - Costs of enhanced testing activities in EDs and for hospital admissions
    - Costs of social mobilization
    - Intensity of social mobilization activities (content, number, timing of activities, and type of activity, such as TV, radio, brochures, posters, community leaders, events, *etc*.)

The protocol team will track all of the expenses listed above. Funds for these items will be held centrally and only disbursed when line item bills are presented. Funds provided by HPTN 065 will be a small supplement to DOH program efforts in Washington, D.C and the Bronx, NY. The protocol team will request costs of existing activities from the DOHs.

The **primary feasibility objectives** for expanded HIV testing in EDs and for hospital admissions will be measured by determining the following:

- Proportion of visits to EDs and admissions to hospitals who receive an HIV test (including all individuals permitted by local or state law to consent for HIV testing in the study communities)
- Number of patients tested in EDs newly identified as HIV-positive and their demographic characteristics
- Number of patients tested during hospital admissions newly identified as HIV-positive and their demographic characteristics
- Number of tested patients identified with previously diagnosed HIV who are not in care
- Cost of support for additional staff and HIV tests

2.2 Design for Expanded HIV Testing

This study component will aim to substantially expand HIV testing activities in the two intervention communities. The Expanded HIV Testing component is a descriptive ecologic study. For this component of the study, outcomes will be evaluated through routinely reported surveillance and process data. Because extensive interventions to expand testing have already been undertaken, the study team will catalogue the existing activities in the intervention communities as they relate to social mobilization, enhanced testing and linkage activities, in collaboration with departments of health in the two communities.

2.3 Study Population for Expanded HIV Testing

The target age population for the social mobilization element of expanded HIV testing will be all persons age 13 and older in the Bronx, NY and Washington, D.C., with special emphasis on MSM.

The target population for the implementation of universal offer of testing in EDs and during hospital admissions will be individuals permitted to consent for HIV testing according to New York State or Washington, D.C. law.

2.3.1 Inclusion Criteria

The inclusion criteria for the universal offer of HIV testing are the following:

- Individuals who are permitted to consent for HIV testing according to New York State or Washington, D.C. law
- Capacity to understand and provide consent for HIV testing
- Admission to a Bronx, NY or Washington, D.C. ED and/or a Bronx, NY or Washington, D.C. hospital

2.3.2 Exclusion Criteria

The exclusion criteria for the universal offer of HIV testing are:

- Lacks the capacity to provide consent for HIV testing
- Acute or urgent medical condition that might be adversely affected by process of obtaining consent or performing HIV test

2.4 Study Sites for Expanded HIV Testing

2.4.1 Hospital/ED Study Sites in the Bronx, NY

Efforts to expand HIV testing will be undertaken at approximately seven Bronx hospitals with at least a Level I trauma center/ED and with daily hospital admissions. Overall, total ED visits at these hospitals in 2006 ranged from 49,635 to 134,969 per hospital.

2.4.2 Hospital/ED Study Sites in Washington, D.C.

Efforts to expand HIV testing will be undertaken at approximately seven Washington, D.C. hospitals. Routine HIV testing through the ED at George Washington University Hospital and throughout Howard University Hospital, begun in 2006, accounted for nearly 25% of all publicly supported tests in Washington, D.C. and 20% of reported positives in the HIV testing data in 2008.

2.5 Interventions for Enhanced HIV Testing Activities

The goal of the enhanced HIV testing activities to be implemented in the intervention communities is to increase the proportion of individuals from the community who have been tested within the prior year, with the intent of achieving earlier detection of HIV infection. Two populations are being targeted: those with ongoing risk through their own or partner risk behaviors and those with prevalent HIV-infection who are unaware of

their HIV infection. The former are targeted for regular HIV testing, at least annually, the latter are targeted for one-time “capture” testing.

At least three factors hamper the existing HIV testing promotion programs: limitations on the ability to influence hospital infrastructure; the inability to collect enhanced data from unfunded agencies; and limited resources to sustain social marketing campaigns. Within each institution, multiple stakeholders (including physicians, administrators and Department of Health staff) have identified necessary logistical changes that would be required to bring HIV screening to scale. Because staff have many other duties that often preclude their ability to focus on these key logistical or infrastructure change, the necessary coordination and follow-through between departments has not taken place. The ability to hire dedicated staff solely on overcoming key logistical barriers to routine HIV screening within each institution should significantly address this first limitation.

Programs such as “The Bronx Knows” have been able to collect aggregate data on HIV screening from all of its testing partners; however, because there has been no direct funding to each organization to support this data collection, the complexity and quantity of process data available to evaluate the initiatives are limited. The study will fund enhanced data collection directly at each site and allow creation of a more robust data set to evaluate various aspects of the testing promotion.

Although funds provided by the CDC in 2008 and 2009 have allowed for the development and dissemination of initial phases of consumer campaigns encouraging HIV testing, resource limitations hampered the sustainability of social messaging campaigns. Supplemental funding from the study will improve both sustainability of ongoing messaging and the creative development of additional components, including a module geared to medical providers. In both jurisdictions, the messaging to the general adolescent/adult population has been to get tested – either annually (Washington, D.C. message) or at least once (Bronx message). However, in both jurisdictions, some sub-populations, such as men who have sex with men, would be best served by more frequent repeated testing. Delivery of targeted enhanced testing messages alongside general testing messages will be supported by the TLC-Plus study.

In addition, the earliest models of routine HIV testing in medical settings were often stand-alone projects working in parallel with the existing medical services, necessary in order to prove feasibility in an environment where routine testing was felt to be burdensome or risky. These models have often relied extensively on the availability of rapid point-of-care HIV tests. However, as routine testing expands new models of integrated testing and lab-based large platform testing may be more appropriate to sustainably meet volume, workload, and cost-efficiency needs. The “early adopter paradigm” applies here – once a program has initial success in routine testing, support is needed for continued evolution or shift of that model. The TLC-Plus study will supplement CDC funding for expanded testing to support this evolution.

The study will build on current social mobilization efforts and communications plans in the intervention communities by adding two refined messages:

- All sexually active individuals age 13 and older should have had an HIV test within the prior year
- Sexually active MSM should seek HIV testing, at least annually, but ideally every six months

This study component will include social marketing to the general population in the two intervention communities, with outreach to the MSM community, healthcare providers, CBOs and social networking systems.

The study aims to achieve the goal of universal offer of HIV testing in EDs and during hospital admissions through the combination of the following approaches, tailored to the needs of each facility:

- Outreach to facility directors and key leaders at these institutions
- Establishment of HIV-testing goals for each facility
- Establishment of HIV testing as part of routine admission orders
- Provision of financial support for HIV-testing kits needed for increased testing
- Development and implementation of computer-delivered information on HIV testing
- Provision of computer terminals

2.6 Study Procedures for Expanded HIV Testing

2.6.1 Study Procedures for Social Mobilization

Both the Bronx, NY and Washington, D.C. have ongoing social mobilization activities as part of their HIV campaigns ("The Bronx Knows" and "DC Takes on HIV"). The HPTN 065 study will provide additional resources to craft and fine tune key messages aimed at increased testing and testing frequency as well as increase testing frequency in specific populations. In addition, the study will also support the development of messages that highlight importance of linkage to HIV care and HIV treatment. Focus groups will assist in developing these messages and in evaluating tools to determine their acceptability and effectiveness. The details of the social mobilization interventions will be determined at the beginning of the study; however, they may include, but are not limited to: flyers/brochures in clubs frequented by MSM; radio commercials; Internet ads/links to study information on sites commonly visited by MSM; and utilizing CBO staff to disseminate "word-of-mouth" messaging in the community.

2.6.1.1 Study Procedures for Social Mobilization in the Bronx

The study will supplement current efforts by the NYC DOHMH for the creative development and placement of social marketing materials throughout the Bronx, NY. Materials produced will both reinforce and supplement current social marketing messaging regarding the importance of routine HIV screening in healthcare settings and will further refine the messaging to the two target populations as indicated above.

The NYC DOHMH will develop social marketing materials using its standard procedures, including (but not limited to) contracting with local advertising agencies to create and produce materials, per NYC contracting practices.

All messaging will be subject to approval by the NYC materials review board, as is the practice for other CDC-funded social marketing materials developed by the NYC DOHMH. Sample materials produced also will be provided to the NYC DOHMH Institutional Review Board (IRB) for review.

Decisions regarding dissemination of social marketing materials will be made by the Bureau of HIV/AIDS Prevention and Control in the NYC DOHMH.

In general, placement of social marketing materials developed for MSM will focus on locations frequently visited by MSM including the Bronx Community Pride Center and local MSM-friendly Bronx clubs, such as Mi Gente. Where possible and appropriate, social marketing materials will be placed on the Internet (*e.g.* on social networking sites frequented by MSM in the Bronx).

2.6.1.2 Study Procedures for Social Mobilization in Washington, D.C.

Financial support will be provided to supplement the comprehensive social marketing program the Washington, D.C. DOH has already developed. This multi-phased program seeks to scale up routine HIV testing, promote treatment and promote behaviors to reduce risk of infection. The program is branded with the umbrella message "DC Takes On HIV" and promotes routine HIV testing in medical settings. Other aspects of the social marketing campaign include a consumer component entitled "Ask for the Test" and a provider component, "We Offer the Test." Using principles successfully implemented by the pharmaceutical industry, the campaign aims to drive consumers to ask for HIV testing when they visit their doctor. The message for providers is that patients will expect HIV testing as part of their standard healthcare. The consumer program features both traditional media (public transit, newspaper, radio and television advertising) and new media (Internet and text messaging). The DOH has developed an umbrella Web site www.DCTakesOnHIV.com with links to resources about HIV testing and services. The DOH has also established a text messaging service where residents can text "DCTEST" to 365247, receive a health fact, and then search by zip code for the nearest free HIV-testing location.

The provider component, "We Offer The Test," includes a toolkit (handbook, pocket card, poster, information cards for patients and appointment cards for HIV specialists) to make it easier for practitioners to implement routine HIV testing. The provider materials

include practical steps to make HIV testing routine, including sample scripts for use with patients, information on billing codes, information cards outlining what patients should know about both negative and positive results, and a “refusal” card that informs patients of the health risk from not getting an HIV test. DOH has formed a partnership with the Global Business Coalition and Pfizer to implement a pilot program under which Pfizer sales representatives will promote routine testing and provide the toolkit during their regular visits to medical practitioners. The pilot will start with 200 physicians and practices in Washington, D.C.

Washington, D.C. will also use social networking media (social Web sites such as Facebook and MySpace) to increase access to Washington, D.C. residents, and will use Twitter as a vehicle to communicate relevant information on HIV. Currently, the DOH has established these sites and a Twitter account for its free condom distribution program. DOH has entered into a contract with a public relations firm to advance the social marketing materials.

2.6.2 Study Procedures for Universal Offer of HIV Testing in EDs and Hospital Admissions in the Bronx

2.6.2.1 Emergency Departments

New York State has requirements for specific information that must be provided to patients before they sign separate, informed consent for HIV testing. HIV testing is already being offered in some EDs in the Bronx. Under this protocol, testing will be expanded at the EDs where it is currently offered and introduced in those that are not testing. Given the complexity and unique exigencies of each hospital's own logistics, this study will work within each facility to establish a procedure for universal offer of HIV testing. A universal offer development team (UODT), hired at the beginning of the study period for each intervention community, will conduct a series of meetings with staff at multiple levels of seniority within each hospital who have some stake in ED care. The UODT will work with these staff to identify and overcome current barriers to universal offer of HIV testing in their ED. Key staff may include the ED Director, the chief of nursing, laboratory director, chief ED residents and chief administrators. The UODT will also work with the ED leadership to set goals for testing and will provide quarterly results to each hospital to create and implement strategies for improvement.

Where necessary to improve logistics, laptop computers attached to rotating stands that can be easily moved from bay to bay will be provided by the study team to the ED site. Videos that meet New York State requirements for pre-test counseling will be loaded onto the laptops. Patients can view the videos and indicate whether they agree to test. Where possible, the video will be set up so that when a patient indicates interactively that he or she agrees to an HIV test, a tester (a nurse, physician or dedicated tester) will be notified and the test will be performed. While the patient waits for results, post-test, HIV-prevention messaging will also be viewable, if the patient chooses to watch it.

All EDs will be provided with free HIV test kits for individuals who are uninsured and cannot bill an insurance carrier (including Medicaid) for HIV screening. Free test kits

will be provided in proportion to the percentage of uninsured individuals seen in that ED who receive an offer of HIV testing at baseline.

The study will also provide EDs with a downloadable computer widget application on routine HIV testing that can be placed on the desktops of all ED computer terminals (either an updated version of the tool created by St. Vincent's Medical Center, with funding from the New York State AIDS Institute, or a similar tool). This widget will not only provide the full CDC Guidelines for routine HIV screening, but will include brief checklists that walk providers through the process of obtaining written consent (currently still required in NY), conducting an HIV test, providing results and handling a positive HIV diagnosis.

In working with the complex logistics in EDs, the study team will also operate within existing hospital admission processes as much as possible.

2.6.2.2 Hospital Admissions

Although some NYC hospitals have initiated programs to offer HIV testing for inpatients, none has yet implemented universal offer of testing at the time of hospital admission. At the beginning of the study period, the UODT will conduct meetings at hospitals with staff at multiple levels of seniority who have some stake in the inpatient admissions process and inpatient medical care. The UODT will work with these staff to identify and overcome current barriers to a universal offer of HIV testing in the hospital admissions process. Key staff will include the medical director, the chief of nursing, the laboratory director, internal medicine and surgery chief residents and chief administrators. The UODT will also work with the medical director and/or other designated hospital leadership to set goals for testing and will provide quarterly results to each hospital to create and implement strategies for improvement.

For any hospital that has set standing (basic, preset) admitting orders, the UODT will work with the Medical Director (or her/his designate) and the hospital's information technology department to have HIV testing added to these standing admitting orders, in accordance with hospital policies. The UODT will also work with the hospital's laboratory to overcome any barriers to placing HIV testing on hospital standing orders. All HIV testing completed as part of this protocol, whether in the ED or during hospital admission, will be conducted according to the laws of New York State. The UODT will work to ensure that all necessary paperwork is streamlined and available on all floors for the admitting teams and for admitting nurses.

All hospital admitting teams will ascertain if the patient has already been tested in the ED during his/her current visit, so that duplicate HIV testing is not performed. If possible, prior tests offered in the ED will be entered electronically, so that the admitting team will know whether the patient had been offered the test.

Admitting teams will be instructed to review the key pre-test counseling points that are required by New York State, obtain consent and, if possible, order the test with routine admitting phlebotomy. If ordering the test with routine phlebotomy is not possible, an HIV rapid test will be performed.

Alternatively (if desired) hospital admitting teams can be provided with laptop computers attached to rotating stands that can be easily moved from patient to patient. Videos that meet New York State requirements for pre-test counseling will be loaded onto the laptops. Patients can view the videos and indicate whether they agree to testing. Where possible, the video will be set up so that when patients indicate interactively that they agree to an HIV test, a tester (a nurse, physician or dedicated tester) will be notified and the test will be performed.

As with EDs, free HIV tests will be provided to the facilities for uninsured persons offered testing on admission. The percentage of tests offered will be derived in similar fashion to the percentage offered for ED testing. Where possible, the same downloadable widget provided to EDs will be provided to inpatient admitting teams for use on their computer terminals.

2.6.3 Study Procedures for Universal Offer of HIV Testing in EDs and Hospital Admissions in Washington, D.C.

2.6.3.1 Emergency Departments

Washington, D.C. EDs have been focal points for the implementation of routine HIV screening because many Washington, D.C. residents only access healthcare through the city's EDs. HIV testing in EDs can be integrated as a routine, opt-out procedure because Washington, D.C. does not require a separate written consent form for HIV testing. Patients are provided with written or verbal information about HIV testing, advised that a test is recommended, and (depending on available staff) a point-of-care rapid test is performed, unless the patient declines. In addition to the two institutions currently conducting routine testing in EDs, the Washington, D.C. DOH has begun to engage six additional EDs to undertake routine, opt-out HIV testing. HAHSTA recently completed review of a Request for Applications (RFA) to support expansion of routine HIV screening in EDs and hospital centers by providing support for a part-time coordinator at each hospital to promote implementation of routine testing. A coordinator at each facility will work with hospital leadership and department heads (CEOs and medical, lab and data staff) to develop plans for routine HIV screening in the ED and in other departments in the hospital system. In Washington, D.C., health insurers are required to reimburse for a voluntary HIV test performed during an insured's visit, regardless of the reason for that ED visit. Study funds will be used to support the cost of tests for patients who are uninsured. In addition, the study will support a part-time coordinator at each institution, working with management and ED and laboratory staff toward implementation of routine screening. The Washington, D.C. DOH will provide TA, including staff training, to all partnering hospitals.

Washington, D.C. has developed a provider toolkit for routine, opt-out HIV testing that includes an introductory brochure, pocket card, result cards and opt-out card. These tools define routine, opt-out HIV testing and provide checklists for the screening process, from test introduction to the provision of follow-up appointments for HIV-positive individuals. These materials will be available for wide distribution to the staff of partnering EDs participating in routine, opt-out HIV testing and to clinical providers throughout Washington, D.C.

The Washington, D.C. DOH will also provide test kits to partner EDs for use with uninsured patients. Although Washington, D.C. legislation mandates reimbursement for HIV testing performed in EDs, none of our partner hospitals has yet developed a protocol for billing for HIV tests. Free tests kits will be made available to hospitals while they develop systems to achieve sustainability through billing and reimbursement procedures. As those systems mature, the hospitals will assume responsibility for procuring test kits independently.

The study will provide financial support to participating EDs in order to increase staffing for HIV testing activities.

2.6.3.2 Hospital Admissions

Washington, D.C. hospitals are already at varying stages in the process of offering HIV testing to inpatients, but none conduct routine screening at admission. Seven of Washington, D.C.'s eight hospitals are performing rapid HIV testing in labor and delivery units for women without a documented HIV test result. Howard University Hospital currently makes rapid HIV testing available during regular business hours, upon physician or patient request. United Medical Center's long-range goal is to implement routine, opt-out HIV testing throughout the hospital.

Intensive TA will be provided to hospitals for admission testing, as with ED HIV testing. In an effort to promote the most cost-effective method of HIV testing, the DOH and study-supported coordinator at each institution will work with hospitals to add routine, opt-out HIV testing to standing orders for admission blood work for all hospital admissions. Washington, D.C.'s lack of a requirement for separate signed consent for HIV testing makes this a feasible option. Hospitals will also be encouraged to offer point-of-care tests for persons who are not tested at the time of admission.

Hospital admissions staff will have access to the routine, opt-out HIV testing reference materials originally developed for EDs. The Washington, D.C. DOH will also provide HIV test kits to hospitals for inpatient testing that must be performed at point-of care (when not done as part of admitting blood work) until they achieve sustainability through billing and reimbursements.

The study will provide financial support to participating hospitals in order to increase staffing for HIV testing activities.

2.7 Study Duration for Expanded HIV Testing

The expanded HIV testing component of this protocol will continue for the duration of the feasibility study, currently projected to be 36 months. It is expected—by virtue of emphasizing stakeholder buy-in, providing training and logistical support, and improving staff and systems capacity— that several key activities undertaken as part of this protocol, such as establishing HIV testing as part of routine admission orders, will be sustained after the study is completed.

2.8 Statistics and Data Analysis for Expanded HIV Testing

2.8.1 Endpoints

2.8.1.1 Endpoints for feasibility of enhancing HIV testing through a combination of focused and enhanced social mobilization efforts

- Number and results of HIV tests per month in publicly funded testing sites (local health department data)
- Number, transmission category and testing source of newly identified cases in HIV surveillance data
- Initial CD4 cell count of newly identified HIV cases in surveillance data
- Number of newly identified HIV cases concomitantly diagnosed with AIDS in surveillance data
- Proportion of persons in the community tested for HIV in the last year (local population-based behavioral surveys)

2.8.1.2 Endpoints for feasibility of routine offer of HIV testing at emergency departments and inpatient units

- Proportion and number of total ED visits and admissions to hospital where patients receive HIV testing
- Number of HIV tests in EDs where HIV infection is newly identified
- Number of HIV tests in hospital admissions where patients receive HIV testing
- Proportion of hospital admissions who have newly identified HIV infection
- Number of tested patients identified with previously diagnosed HIV who are not in care
- Cost of support for additional staff and HIV tests

2.8.2 HIV Testing Intervention Goals

More than 72,000 publicly supported HIV tests were conducted in Washington, D.C. in 2008, of which approximately 1000 were new positive diagnoses. Likewise, between April 2008 and March 2009, 161,619 HIV tests were performed as part of "The Bronx Knows" initiative, of which approximately 700 were new, positive diagnoses. This study will evaluate the increase in overall testing in Washington, D.C. and the Bronx. In addition to the volume of testing and number of new HIV diagnoses, the study will evaluate trends in CD4 cell count at first diagnosis.

Enhanced access to HIV testing in both ED and inpatient settings will occur throughout the entire 36-month study period. With regard to specific targets for the testing effort, the study will aim to offer HIV testing to 80% of eligible individuals during 80% of visits

with a 60% acceptance rate in the EDs. Likewise, the study will aim to offer HIV testing to 80% of eligible individuals with a 70% acceptance for inpatient admissions. The absolute numbers that these percentages represent will vary from hospital to hospital. The study will evaluate the ED and inpatient HIV data for increases in volume of testers, increases in number of HIV-positive tests and increased volume and percentage of new HIV infections detected.

2.8.3 HIV Testing Baseline Data

Table 1 contains the current levels of testing volume and newly identified cases from Health Departments and Surveillance data in each of the six cities (intervention and non-intervention) for 2007 and 2008.

Table 1. Testing Volume and Results in the Six Intervention and Non-Intervention Communities

Cities	2007				2008			
	No. of HIV Tests N	No. of Sites N	HIV+ Results % (n)	Newly Identified HIV+ % (n)	No. of HIV Tests N	No. of Sites N	HIV+ Results % (n)	Newly Identified HIV+ % (n)
Bronx[a]	127,947	21	1.3% (1,600)	727[b]	161,619[c]	21	0.9% (1,513)[c]	0.4% (691)[c]
Washington, D.C.	43,271	47	2.8% (1,192)	1.6% (702)	72,864	55	2.5 % (1,828)	0.8% (555)
Chicago	Not Available				77,616	42	1.0% (789)	0.5% (363)
Houston	38,612	16	1.05% (404)	1.70% (128)[d]	38,763	16	1.50% (580)	1.29% (109) [d]
Philadelphia	44,504	315	1.8% (780)	1.6% (710)	66,246	315	1.0% (678)	0.7% (489)
Miami	48,696	100	3.1% (1509)	1.8% (878)	59,510	117	2.7% (1611)	1.6% (912)

a) Data from *The Bronx Knows* Initiative
b) Data on linkage-to-care and newly diagnosed are only available for 19 agencies participating in *The Bronx Knows* for 2007.
c) *The Bronx Knows* agencies began reporting data in April of 2008; data reported here therefore covers the period of April 2008-March 2009 to provide one year's worth of data.
d) Data on newly identified HIV+ in Houston included only a subset of testing facilities. Numerators and denominators for these data reflect only that subset of tests/facilities.

2.8.4 Data Analysis

Data to be analyzed for this study component will be obtained from routine HIV surveillance sources and from utilization data collected by participating hospitals.

Endpoint measures of the potential feasibility of large-scale implementation of an expanded HIV testing strategy will be obtained by two analytic strategies: 1) comparison within the intervention communities before vs. after the intervention, and 2) comparison of change between communities with vs. without the intervention. For the within-community comparison, we will monitor the change from pre- to post-

intervention, with the primary interest in the change achieved at the end of the intervention period. For the between-community comparison, we will compare the change in communities with vs. without the intervention. Thus, we will test two indicators of change as a result of the intervention:

- **Within**: Comparison of outcomes of interest before vs. after intervention in an intervention community

- **Difference of differences**: Comparison of the change in a measure during the study period in communities with vs. without the intervention cities

Measurement of change or temporal trends in any of the outcome measures of the intervention implies that both of the above indicators of change will be computed.

2.9 Human Subjects/Ethical Considerations

The expanded HIV testing study component is a public health practice. Two geographic areas will participate in this study component: Washington, D.C. and the Bronx, NYC. Social mobilization and emergency department testing is already taking place in the two intervention communities (Bronx and Washington, D.C.) and is intended to be specific to the needs of those populations. While some observations from the overall TLC Plus project with regard to social mobilization and expanded testing may be applicable elsewhere in the United States, these activities were initiated originally for the specific benefit of the respective communities, and do not constitute research (CDC 1999).

The protocol will be submitted to appropriate IRBs (a central and/or local site IRBs) for ethical review prior to study initiation. Any subsequent modifications to the protocol will be submitted to appropriate IRBs, and, at a minimum, the protocol will be submitted annually for continuing review and approval by these same ethics boards. Because the expanded HIV testing component is public health practice applied in the two study communities, the study team will request that IRBs reviewing the protocol as a whole consider only the expanded testing component to be a non-research component, and therefore not subject to the requirements of 45CFR46. The other three components of this protocol constitute research and will be addressed in separate sections.

In Washington, D.C., written informed consent is not required for routine HIV testing; only oral consent, as part of standard voluntary opt-out HIV testing, is required. In contrast, New York State requires written informed consent for individuals undergoing HIV testing. For the expanded HIV testing component of this study, participants will undergo HIV testing according to the SOC and legal requirements of their jurisdiction.

To assess the impact of the study on HIV testing in the communities of Washington, D.C. and the Bronx, surveillance data routinely collected by the DOHs will be analyzed. No individual data, other than what exists in the surveillance databases, will be collected from study participants in the HIV-testing component of this study.

No study-specific laboratory testing will be conducted under this protocol. Therefore, no additional study-related test results will be reported to authorities. HIV-testing data for

tests performed in the EDs, hospitals and community test sites in Washington, D.C. and the Bronx, NY will be reported per local HIV reporting requirements.

The study team will neither collect nor report Adverse Events because there is no biomedical intervention. Again, due to the nature of this study component, the team will not collect or report Social Harms.

3.0 LINKAGE-TO-CARE

3.1 Study Objectives for Linkage-to-Care

The **primary feasibility objective** for using FIs to facilitate linkage of HIV-positive patients to HIV care sites will be measured by determining the following:

- Overall cost of the program, including staffing, program materials and incentives
- Extent of other available linkage-to-care activities (case managers, peer navigation, *etc*.)

The **primary effectiveness objective** for using FIs to facilitate linkage of patients from HIV testing to HIV care sites is the following:

- To increase the proportion of newly HIV-diagnosed and out-of-care patients linking to care within three months of identification

The protocol team will track all of the expenses listed above. Funds for these items will be held centrally and only disbursed when line-item bills are presented.

In order to monitor the implementation of the FI program, certain parameters will be gathered from a subset of participating sites at various time points to ensure program quality. These parameters will include: the proportion and number of patients eligible for incentives (the number of HIV–positives at a participating site), the proportion and number of patients receiving incentives, and the amount received compared with the total number who are eligible.

3.2 Design for Linkage-to-Care

The Linkage-to-Care component of the study is a two-arm, site-randomized, prospective, effectiveness clinical trial conducted within each intervention community. This study component compares the effectiveness of an FI intervention to link HIV-positive individuals from HIV test sites to HIV care sites with the usual SOC procedures. Each HIV test site will be randomized to either the intervention or SOC arm of the study. For this component of the study, study outcomes will be evaluated through routinely reported HIV surveillance data. Using surveillance data, study outcomes will continue to be evaluated for a period of one year after the FI intervention ends.

In order to identify the SOC for linkage, against which the incentives intervention will be assessed, an appropriate facility administrator will complete a brief survey that will collect key attributes of HIV testing sites, including types of navigation and support services already available to patients to facilitate linkage-to-care. Data will be collected from sites annually from 2009 - 2013.

For the purposes of this protocol, we consider an HIV-positive individual linked to care when that person has a VL or CD4 cell count assessment at an HIV care site within 3 months of confirmatory WB testing.

3.3 Study Population for Linkage-to-Care

The Linkage-to-Care component of the study will include all individuals ages 12 and older who are permitted to consent, or can be consented for HIV care by a parent/legal guardian according to New York State or Washington, D.C. law, and who are newly found to be HIV-positive at HIV test sites participating in the study. This study component will also include individuals who have been previously diagnosed with HIV but have been out of care for at least a year and are reconfirmed for HIV infection by standard laboratory tests.

3.4 Study Sites for Linkage-to-Care

Twenty HIV test sites will be selected from Washington, D.C. and 20 HIV test sites will be selected from the Bronx to participate in the Linkage-to-Care component of this study.

HIV test sites will be selected based on two primary criteria: 1) site agreement to participate in this component of the study, and 2) sites with the highest volume of HIV-positive individuals identified in the previous year. Additional site selection criteria may be considered if a total of 40 HIV test sites cannot be chosen based on the primary criteria.

In each community, these 20 sites will be randomized such that 10 will use the FI intervention, described in Section 3.5, to link HIV-patients to care and 10 will use the SOC only.

The site randomization will be balanced by the following two baseline characteristics:

- The number of HIV-positive individuals identified in the previous year
- The rate of linkage-to-care within three months of HIV diagnosis over the course of the calendar year prior to study initiation

In addition, 20 care sites will be selected in Washington, D.C. and 20 care sites will be selected in the Bronx, NY. These care sites will redeem the coupons provided to patients by the test sites selected for the FI arm.

3.5 Intervention for Linkage-to-Care

At HIV test sites assigned to FIs, individuals permitted to consent for HIV care according to New York State or Washington, D.C. law who test HIV-positive will be provided with a coupon that is redeemable at participating HIV care sites. These coupons will be designed so they will not breach patient confidentiality (for example, there will be no patient names, no indication of HIV status, and no clinic names on these coupons). Patients presenting coupons to participating HIV care sites will be given an FI upon completion of a blood draw/lab visit ($25), which is usually the first visit, and another FI

($100) upon an interaction with their healthcare provider, which is usually at a second visit. Alternatively, for those patients who complete a comprehensive visit at participating care sites, a visit that includes both the lab and provider components for Linkage-to-Care, a $125 FI gift card will be provided.

The coupons distributed at the HIV test sites must be redeemed at the HIV care sites within three months of the date that a participant receives them.

The proportion of persons successfully linked to care within three months of their HIV-positive test will be compared, through the use of routinely collected HIV surveillance data, between sites implementing the incentives intervention and those with SOC procedures for linkage-to-care.

3.6 Study Procedures for Linkage-to-Care

The specific procedures for HIV test sites to obtain and distribute the FI coupons for linkage-to-care, as well as the procedures for the redemption of these coupons at HIV care sites, are outlined in the HPTN 065 Study-Specific Procedures Manual (SSP).

3.6.1 Procedures at Test Sites

Participating test sites will link all HIV-positive patients to an HIV care site. Each test site will be provided with a listing of participating HIV care sites (with contact information) to give to HIV-positive patients. In addition, test sites will give HIV-positive patients coupons redeemable for FIs at HIV care sites.

Prior to study implementation, all HIV test site staff will be trained on the procedures for the FI intervention. Providers will also be trained on HIV prevention counseling, and the importance of linking HIV-positive patients to care.

Study test sites will maintain the ability to refer clients with urgent needs (e.g., mental health) for necessary services. However, test sites will not routinely link patients to support services. The DOHs in both intervention communities actively encourage a *single* referral of HIV-positive patients from test sites to care sites. Multiple referrals can be overwhelming to newly HIV-diagnosed patients and may give the wrong impression that an individual must be drug-free in order to engage in HIV care, and may paradoxically delay entry into care. Additionally, testing sites usually do not have ongoing relationships with persons in need of support services, and are thus not well suited for evaluating sustained linkages to these resources or adequacy of services to meet patient needs.

3.6.2 Procedures at Care Sites

Participating HIV care sites will serve as the comprehensive ‘medical home’ for coordination of all HIV-positive patient care needs, including substance use, mental health, and other support services.

As newly diagnosed HIV-positive patients present for care, care sites will evaluate CD4 cell count and VL. Care sites will then create an appropriate medical plan for each patient, consistent with current HIV care and treatment guidelines. Simultaneously, care

sites will also assess patients and link them as appropriate to support services. Finally, valid coupons distributed by HIV test sites will be redeemed with FI gift cards.

Prior to study implementation, all HIV care site staff will be trained on the procedures for the FI intervention, ART initiation according to current guidelines, and the importance of linking HIV-positive patients to appropriate support services. In addition, as part of study orientation and training for HIV care providers, existing support service linkages will be reviewed.

To collect clinic-level information on services available to patients in the two jurisdictions, HIV care sites will be surveyed at baseline and annually thereafter to ascertain support services available on- and off-site (e.g., social services, substance use treatment, support groups, mental health resources, etc). The study team will develop and maintain a comprehensive list of support services in the two intervention communities. In addition, the study will regularly update lists for care sites of contact persons and referral processes at support service agencies.

Because the study will use surveillance data to measure outcomes and will not collect data on individual patients, the study will not be able to document or track linkages to ancillary services in the Linkage-to-care and Viral Suppression study components. Information on referrals made and completed by patients is not part of the surveillance data that will be analyzed for study outcomes. Likewise, offering FIs for completion of linkages to drug treatment or mental health services is not possible, as it would also require following individual patients with explicit data collection. However, for the subset of patients completing Patient Survey, we will collect data on the frequency of use, ease of access, and patient experiences with support services (including mental health, drug abuse, case management, etc).

3.6.3 Monitoring HIV Test Sites

It is possible that members of the intervention communities will learn of the FI to strengthen linkage-to-care and seek out HIV test sites offering these incentives. In all likelihood, the majority of individuals who seek testing will not know their HIV status yet and, thus, will be unaware that incentives are being offered for linkage-to-care. In addition, many of the HIV test sites in the study (~seven out of the 20 participating HIV test sites in each city) will be hospitals in which HIV testing will take place in emergency departments and inpatient units. Individuals seen in these settings are usually not there for the purpose of obtaining an HIV test. Thus, it is unlikely that these individuals will seek medical services based on the availability of FIs for linkage to HIV care.

However, it is possible that some individuals, who are aware of their HIV infection, may "recycle" through the HIV test sites due to the availability of FIs. There are some natural barriers to this phenomenon, as it is likely that the staff at these HIV test sites will recognize individuals who are testing with undue frequency. In these cases, staff may withhold the FI coupons from those who have already received them for a prior HIV-positive test. Another natural barrier is that individuals are known at HIV care sites, so individuals will be unable to re-link repeatedly to the same HIV care site. In addition, the FI intervention is designed such that multiple activities (lab-work and provider

interaction, both of which take time and effort) are required for the full redemption, which may discourage some from repeated linkage.

It should also be noted that based on the HIV prevalence in the intervention cities, relatively few people test positive out of all of those who test. Thus, the number of people who could "recycle" through the HIV test sites is relatively small compared to the total testing done in these venues.

Despite all of the barriers to HIV-test-site migration and linkage recycling, FI may encourage these phenomena; thus, the study team will use surveillance data to monitor these potential situations. Specifically, the team will monitor the number of duplicate linkage-to-care events in the name/ID-based surveillance data system over time. So, for example, in the case where a person re-tests at five unique HIV test sites (all offering linkage-to-care incentives) and links to five unique participating HIV care sites over the course of six months, HIV surveillance data will capture this event.

In addition, the team will monitor whether the number of positive HIV tests becomes severely disproportionately distributed among the participating HIV test sites (a sign that HIV testing is much reduced or increased at some sites). This information will be examined in an on-going fashion, and if site migration or linkage recycling becomes highly extensive so that it becomes a burden on HIV test or care sites, or threatens the validity of the evaluation, this component of the study will be re-evaluated. Such re-evaluation may include discussions with the community advisory group to design other mechanisms to minimize such migration, or, if needed, a redesign of this component of the study may be embarked on by the study team.

3.7 Study Duration for Linkage-to-Care

The duration of the FI intervention for the Linkage-to-Care component of the study is depicted in Figure 2. Coupons will be available at the HIV test sites randomized to the FI arm for 21 months. These coupons can be redeemed after the initial visit with an HIV provider at HIV care sites for a total of 24 months (for the entire time coupons are distributed at certain HIV test sites plus an additional three months).

Figure 2: Duration of FI Intervention for Linkage-to-Care

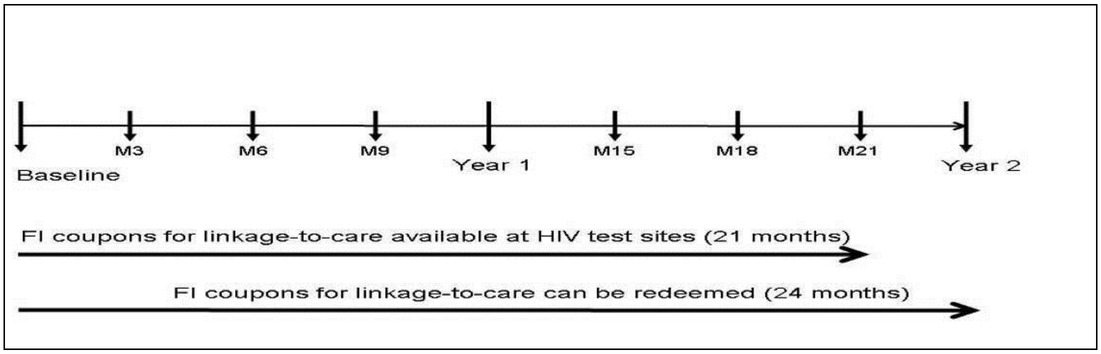

3.8 Statistics and Data Analysis for Linkage-to-Care

3.8.1 Endpoints

3.8.1.1 Endpoints for the feasibility of using financial incentives in the HIV-positive population for Linkage-to-Care

The feasibility endpoints for the Linkage-to-Care component of the study are the following:

- Number of individuals eligible for incentives (see section 3.3) and number of individuals receiving incentives (upon linkage to HIV care) at participating sites
- Cost of the program including staffing, infrastructure and incentives

3.8.1.2 Endpoints for the Effectiveness of Linkage-to-Care

In this site-randomized design, Linkage-to-Care effectiveness endpoints will be evaluated for each site and intervention effectiveness assessed by comparing site outcomes for intervention vs. SOC sites.

The effectiveness endpoints for the Linkage-to-Care component of the study will be ascertained based on HIV surveillance data and will include the following:

- Proportion of HIV-positive individuals at each testing site, who either have a newly detected HIV infection (based on new surveillance case with confirmed WB) or who were previously diagnosed but were out of care (based on no VL or CD4 ascertainment in the year prior to a repeat WB test in an existing surveillance case), and who presently are linked to care as evidenced by having a CD4 cell count and/or VL measurement at a separate visit within three months of WB confirmation

- Mean time interval at each testing site from HIV diagnosis (WB confirmation) to first CD4 cell count or VL for those with newly detected HIV infection and those who were previously diagnosed but were out of care

- Proportion of HIV-positive individuals at a testing site (overall, and separately for those with newly detected HIV infection and those previously diagnosed who were out of care) with at least two CD4 cell count and VL measurements in the prior year

3.8.2 Sample Size and Power

The primary endpoint of this study component is proportion of cases (newly diagnosed and previously diagnosed but not in care) with a CD4 cell count and/or VL measurement within three months of the diagnostic WB.

Randomization is by testing site, with balance achieved for baseline volume of testing and baseline linkage-to-care rates. Accrual of participants into the Linkage-to-Care

component of the study will occur over the first 24 months of the study, when eligible persons receive the FI coupon at an HIV test site randomized to the FI arm of the study. There will be no follow-up for this component of the study, other than the two visits in care (or a combined lab/provider intake visit).

In 2007, in the 20 testing sites with the highest volume in the Bronx, NY, 611 newly diagnosed HIV-infected people were identified. Of those, 77% were liked to care within three months, with linkage at different testing sites ranging from 30% to 100%. The mean number of newly diagnosed cases per provider in 2008 was 31, and ranged from five to 93. The intra-class correlation (ICC) of proportion linked to care in the Bronx was 0.27.

In 2008, in the 20 testing sites in Washington, D.C, the jurisdiction with the highest volume of people testing HIV-positive, 783 newly infected cases were identified. Of these, 67% were linked to care, ranging across different testing sites from 5% to 100%. The mean number of newly diagnosed cases per provider in 2008 was 40, and ranged from eight to 143. The ICC of proportion linked was 0.31.

These estimates do not include the population of those re-linking to care. It is anticipated that linkage success in SOC for this population will be lower because of the history of loss to care. We could reasonably expect that: 1) the number of people eligible for linkage-to-care will be higher because of longer study duration and addition of re-linked to care cases; 2) the proportion linked to care will be lower because of the re-linked to care cases; and, therefore, 3) the ICC will be smaller. It is assumed that the number of cases to be linked to care during the 24-month study duration will be similar to these past case loads on an annual basis. We will randomize 20 testing sites in each intervention city, and we expect to identify between 1200 and 2400 individuals for linkage-to-care, a mean of 60-120 people per testing site.

Table 2 below gives the mean number of linkage cases needed per testing site to detect an increase in linkage-to-care given 20 sites per arm (*i.e.* 40 in all). With a mean ranging from 80 to 100 linkages per site during the 24 months of the study, we would have 80% power, even with a high ICC, to detect a 13% change in proportion linked to care. If higher numbers are eligible for intervention due to linkage-to-care (*e.g.* 140), we would attain 90% power to detect this magnitude of difference. Note that the ICC coefficient estimated from the linkage-to-care of newly detected cases is high in large part because of sites with a small number of newly detected cases and 100% success in linking to care. It is probable that the ICC observed in the trial will be lower.

This sample size calculation for clustered designs uses the approach described by Thomson, Hayes and Cousens (Hayes and Bennett 1999; Thomson, Hayes et al. 2009).

Table 2. Mean number at each site required to achieve sufficient power to detect differences in proportion of newly detected or reconnected to care within three months of testing. Number of clusters fixed at 20 per arm

Mean percentage linked to care at a site		Standard deviation of percent linked to care		Intra-class correlation	Mean number of potential cases for linkage-to-care per testing site	
Standard of care	Intervention	Standard of care	Intervention		80% power	90% power
67%	80%	17%	12%	0.127	37	49
67%	80%	20%	15%	0.183	54	72
67%	80%	26%	19%	0.309	102	137
67%	75%	17%	14%	0.127	105	140
67%	75%	20%	17%	0.183	152	203
67%	75%	26%	22%	0.309	287	384

3.8.3 Randomization Scheme

In the Linkage-to-Care study component, HIV testing sites will be randomized to use FIs or the SOC.

HIV test sites will be assigned to one of the following two arms:

- An arm offering FIs: Test sites assigned to this arm will provide coupons to all individuals testing HIV-positive, who are not already linked to HIV care. The coupons can be redeemed for the FI at a participating HIV care site.

- An arm continuing with SOC: Each person who tests HIV-positive, and is not currently in care, will be directed to HIV care sites using the site's SOC procedures.

The site randomization will be balanced by the following two baseline characteristics:

- The number of HIV-positive individuals identified in the previous year

- The rate of linkage-to-care within three months of HIV diagnosis over the course of the calendar year prior to study initiation

3.8.4 Data Analysis

Data to be analyzed for this study component will be obtained from routine HIV surveillance data as well as from the participating sites. Participating sites will maintain tracking logs, both for coupons provided to HIV-positive individuals at the test sites and for FI disbursement at the care sites. Information from the tracking logs, with all participant-identifying information removed, will be provided to the study team.

The analysis approach for testing the effectiveness of an FI intervention to link HIV-positive individuals from HIV test sites to HIV care sites compared to the SOC is described below. Analysis of secondary endpoints will be detailed in the statistical analysis plan.

3.8.4.1 Primary Effectiveness Analysis

The population for assessment of Linkage-to-Care is the following:

1. All individuals with newly detected HIV infections reported to the HIV surveillance system for the DOH jurisdiction of Washington, D.C. and the Bronx, NY who were tested at any of the 40 testing sites in the study

2. All participants eligible for re-linkage to care, defined as a WB recorded in the enhanced HIV/AIDS Reporting System (eHARs), at any of the 40 testing sites in the study after the implementation of FIs, who had no VL or CD4 assessment by a provider in the previous 12 months

The endpoint is the percentage of participants with a VL or CD4 cell count assessment within three months of the WB confirmation (separate from clinical assessment done at the time of the confirmation visit). Participants in the FI arm are attributed to the linkage-to-care strategy in place at the site where the first HIV-positive result was detected.

The estimation of intervention effect will test for a change in odds ratio of linkage-to-care between the SOC and FI test sites, assuming a correlation within participants tested at the same site, using Generalized Estimating Equations (GEE) methods.

3.8.4.2 Feasibility Endpoints

Analysis of feasibility endpoints will use descriptive statistics to characterize the variability of uptake of FIs across types of testing venues.

3.9 Human Subjects/Ethical Considerations

The Linkage-to-Care component of the study involves a randomized intervention using FIs to link HIV-positive individuals to HIV care after diagnosis. This component is public health research. In this component, HIV test sites will be randomized, not individuals, and no de novo individual-level data will be collected. Instead, (de-identified) HIV surveillance data routinely collected by the Departments of Health (DOH) in the two communities will be analyzed. Because this research involves minimal risk and would be impracticable with informed consent, a waiver of patient informed consent will be requested under 45 CFR 46.116 (c) or (d).

The protocol will be submitted to appropriate IRBs (a central and/or local site IRBs) for ethical review prior to study initiation. Any subsequent modifications to the protocol will be submitted to appropriate IRBs, and, at a minimum, the protocol will be submitted annually for continuing review and approval by these same ethics boards.

This component of the study involves an intervention using FIs to link HIV-positive individuals to HIV care after diagnosis. These incentives are described in detail in the intervention section for this study component. No additional incentives (for example for transportation) will be given to individuals who participate in this component of the study.

No individual data, other than what exists in the surveillance databases, will be collected from study participants in the Linkage-to-Care component of this study. To assess the impact of the study on Linkage-to-Care in the communities of Washington, D.C. and the Bronx, HIV surveillance data routinely collected by the DOH will be analyzed. Data from the DOHs will be provided to the study team in a de-identified fashion, as per usual HIV surveillance procedures.

No study-specific laboratory testing will be conducted under this protocol. Therefore, no additional study-related test results will need to be reported to authorities. HIV testing and care data collected during routine clinical care will be reported per local HIV and AIDS reporting requirements.

3.10 Safety Monitoring and Adverse Event Reporting

The study team will not collect or report Adverse Events because there is no biomedical intervention. However, the team will collect and report all social harms that are brought to the attention of study staff members, using a study-specific incident report form. This form will be anonymous and will query common social harms such as altered personal relationships, forced change in housing, and physical violence. The form will also include space for a written narrative to document additional details of any social harm experienced. All research staff will be trained to properly complete the form. As a part of study training, research staff will also be trained on the provision of referrals to counseling and social service support. Reports of social harms will be reviewed quarterly or more often, if indicated, and reported to the medical officer together with any actions that are taken. Social harms will be summarized and reported to appropriate IRB(s) on an annual basis.

4.0 VIRAL SUPPRESSION

4.1 Study Objectives for Viral Suppression

The **primary feasibility objectives** for using FIs to incentivize HIV-positive patients to achieve and maintain viral suppression (HIV RNA < 400 copies/mL) will be measured by determining the following:

- Proportion and number of patients in care, on ART (eligible for incentives), and receiving incentives upon achieving VL suppression
- Number of incentives disbursed compared with the total number of incentives available to eligible patients over the study duration [measurement of duration of viral suppression]
- Overall cost of program including staffing, infrastructure and incentives
- Number of patients previously adherent to ART who change from non-intervention to intervention care sites

In order to monitor the implementation of the FI program, certain parameters will be gathered from a subset of participating sites at various time points to ensure program quality. These parameters will include: the proportion and number of patients in care, on ART (eligible for incentives) and receiving incentives, as well as the incentive amount received compared with the total number on ART who would be eligible.

The **primary effectiveness objective** is to compare an FI intervention with the SOC for achieving and maintaining suppressed VL in HIV-positive patients in care, managed under the prevailing guidelines for the initiation of ART. For sites in each intervention arm, this objective will be measured by the following:

- Comparing the mean proportion of patients in care at a site who have suppressed VL (HIV RNA <400 copies/mL) during the fixed 12-month calendar evaluation period beginning 12 months after initiation of the FIs at that site
- Comparing the trend in mean proportion of patients in care at a site who have suppressed VL over time since initiation of FIs

- Comparing the mean proportion of patients who have suppressed VL (HIV RNA <400 copies/mL) after cessation of incentives

4.2 Design for Viral Suppression

The viral suppression component of the study is a two-arm, site-randomized, prospective, effectiveness clinical trial. It will be conducted within each intervention community to assess the effectiveness of a FI intervention for achieving and maintaining suppressed VL (HIV RNA < 400 copies/mL) in HIV-positive individuals (who have initiated ART under prevailing guidelines) compared to SOC for supporting patients in achieving adherence and virologic suppression. Each HIV care site will be randomized to either the intervention or SOC arm of the study. For this component of the trial, study outcomes will be evaluated through routinely reported HIV surveillance data. Using surveillance data, study outcomes will continue to be evaluated for a period of one year after the FI intervention ends.

In order to identify the SOC for ART adherence/viral suppression support, against which the incentives intervention will be assessed, we will develop a brief survey to be completed by an appropriate facility administrator, which will collect key attributes of HIV care sites, including types of ART adherence support, case management services, and other support services already available to patients. Data will be collected from sites annually from 2009 - 2013.

4.3 Study Population for Viral Suppression

The study population for the viral suppression component of the study will include all individuals ages 12 and older who are permitted to consent, or can be consented for HIV care by a parent/legal guardian according to New York State or Washington, D.C. law, who have initiated care at participating HIV care sites.

4.4 Study Sites for Viral Suppression

Twenty HIV care sites will be selected from Washington, D.C. and 20 HIV care sites will be selected from the Bronx to participate in this component of the study. Sites will be selected based on two primary criteria: 1) site agreement to participate in this component of the study, and 2) sites with the highest number of HIV-positive patients in care in the previous year. Additional site selection criteria may be considered if a total of 40 HIV care sites cannot be chosen based on the primary criteria.

HIV care sites will be randomized 1:1 to the FI vs. SOC, stratified by city and balanced by size of patient population in a clinic and baseline levels of proportion of VL showing suppression. In each city, these 20 sites will be randomized such that 10 will use the FI intervention, described in Section 4.5, to promote viral suppression in HIV-patients on ART and 10 will continue the existing SOC.

The site randomization will be balanced by the following two baseline characteristics:

- The size of the site's HIV-positive patient case load

- The proportion of HIV-positive patients with VL suppression at each site

4.5 Intervention for Viral Suppression

HIV-positive individuals at HIV care sites assigned to the FI intervention will receive FIs (gift cards worth $70) to reinforce adherence to ART as measured by confirmation of each suppressed VL measurement (<400 copies/mL). The gift cards will be distributed at the HIV Care sites during each routine, quarterly clinic visit that a study participant demonstrates a suppressed VL.

In the event participants report full adherence to their providers but have not succeeded in achieving VL suppression, providers will be expected to obtain resistance testing, as recommended by current treatment guidelines, and to alter their patients' regimens accordingly. Once patients with resistant HIV are on appropriate regimens, they will again be eligible for FIs for viral suppression. The specific procedures for HIV care sites to distribute the FIs for viral suppression will be outlined in the HPTN 065 SSP.

This intervention will be compared to SOC support for ART adherence through the use of routinely collected HIV surveillance data.

4.6 Study Procedures for Viral Suppression

At the initiation of the study, the study team, along with experts in treatment and management of HIV disease, will provide training to providers in the intervention communities on current HIV-treatment guidelines. Training will encourage providers to maximize initiation of ART per current guidelines and will emphasize the following: eligibility criteria for ART as defined in the guidelines; assessment of patient readiness to start ART (if eligible); prompt initiation of ART (if eligible); use of recommended regimens; the provision of supportive services to ensure adherence; regular clinical and laboratory monitoring; the importance of assessment for evidence of treatment failure; reinforcement of adherence; obtaining HIV resistance testing (if appropriate); and modification of failing regimen with new effective regimen. All HIV care site staff will also be trained on the procedures for the FI intervention prior to implementation. Along with this training, the importance of linkage to supportive services will be emphasized, including income assistance, housing support, substance use management and mental health services. Each HIV care site, regardless of the study arm it is randomized to, will be provided with a listing of available resources in the community with contact names and numbers. Providers will also be trained on HIV prevention counseling.

A baseline survey will be conducted to determine the availability of supportive services at all participating HIV care sites (*e.g.* social services, adherence support, substance use treatment, support groups, mental health resources, *etc.*) and to determine whether these services are available on site or by referral. In addition, an effort will be made to monitor the number of new referrals and waiting times for appointments to assess program capacity.

The specific procedures for HIV care sites to distribute the FIs for viral suppression will be outlined in the HPTN 065 SSP.

4.6.1 Monitoring HIV Care Sites

It is possible that members of the intervention communities, particularly those who are HIV-positive, will learn of the FI intervention to promote viral suppression and seek out HIV care sites offering these incentives. In an effort to minimize significant migration of patients from HIV care sites without FIs to ones that offer them, the following rules have been put into place:

- A patient must have at least one VL measurement at the HIV care site between three and nine months prior to the first suppressed VL for which he or she may receive an incentive. Both of these VL measurements must be done at the same HIV care site.

- All subsequent VL measurements for which a patient may receive FIs must be performed at the same HIV care site.

If a patient changes HIV care sites during the course of the study, he or she will be required to "establish care" at the new HIV care site prior to receiving any FI for a suppressed VL as indicated above. Specifically, to receive an FI again, the individual must have a VL measurement at the new HIV care site and return in three to nine months for a repeat VL measurement. The results of the second test will determine whether the person receives the FI or not.

Despite this barrier to HIV-care-site migration, the FI intervention may encourage this phenomenon; thus, the study team will use surveillance data to monitor the case load at each site at frequent intervals. By monitoring HIV care site case load over time, the team will be able to determine if significant migration is taking place. This will motivate discussions with the community advisory group to design other mechanisms to minimize such migration or if needed a redesign of this component of the study may be embarked on by the study team.

4.7 Study Duration for Viral Suppression

The duration for the FI intervention for the viral suppression component of the study is depicted in Figure 3. Participants will be eligible to receive FIs for suppressed (< 400 copies/mL) VL measurements once every three months throughout the entire 24-month study period. Participants who miss a quarterly visit(s) remain eligible for incentives as long as they present back to care with a suppressed VL measurement.

Patients with HIV diagnoses returning to care after a hiatus (of at least a year) and individuals who switch HIV care sites during the study are all eligible for the FIs. However they must have at least one VL measurement at three months (or more) at the same HIV care site as the qualifying VL measurement for which they can receive an FI.

Accrual of participants into the Viral Suppression component of the study will occur over the entire 24 months of the study. Some participants may receive up to nine FI payments if they are seen at the same HIV care site throughout the entire study, had been receiving care at that the same site before the study began, and maintain viral suppression throughout the 24 months.

Figure 3: Duration of FI Intervention for Viral Suppression

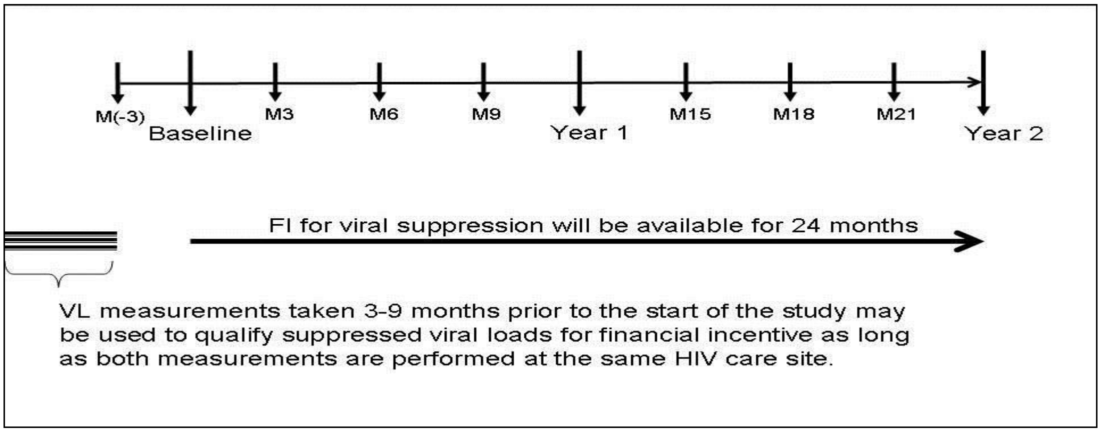

4.8 Statistics and Data Analysis for Viral Suppression

4.8.1 Endpoints

4.8.1.1 Endpoints for the feasibility of using financial incentives in the HIV-positive population for viral suppression

The feasibility endpoints for the viral suppression component of the study will include the following:

- Number of individuals eligible for incentives and receiving incentives at a select subset of sites for select time points
- Cost of program including staffing, infrastructure and incentives

4.8.1.2 Endpoints for the Effectiveness of Viral Suppression

The effectiveness endpoints for the viral suppression component of the study will include comparing between sites in the intervention and SOC arms:

- Probability of an HIV-positive patient in care at a site having a suppressed VL (<400 copies/mL) in the 12-month calendar assessment period beginning 12 months after initiation of the assessment period
- Number of identified HIV-positive patients in care who have sustained viral suppression (see below)

The primary endpoint of the intervention is evaluated using all VL ordered at each site in the 12-month period beginning 12 months after initiation of FIs. An individual will be defined as in care at a site if two VL or CD4 assessments were ordered at that site in the year prior to initiation of this study, or if care (defined by two VL/CD4 assessments) is initiated at the site during the study. PLWHA will be assigned to sites (and thus arms) based on the site where they are in care.

To avoid ascertainment bias that would arise from missing VL assessments in the in-care cohort, we will impute whether VL is suppressed in each quarter of the 12-month assessment period a patient is in care (this is based on an SOC where VL assessments are ordered approximately every three months). For example, a patient with a VL assessment in each quarter will have an endpoint assessment for each quarter. A patient who has a suppressed VL assessment in the first, third and fifth quarters, but no assessment in the second and fourth, will be assumed to be suppressed in the second and fourth. However a patient with a suppressed VL in the first quarter and no subsequent assessment throughout the study will be assumed not suppressed in all subsequent quarters. The algorithm for imputing VL suppression (incorporating the sequence of VL assessments and including management of the reporting lag in surveillance data), inclusion of assessments in the quarters preceding and following the assessment period, handling of cases in treatment at more that one site, and censoring because of moving outside the surveillance area, death, *etc*. will be detailed in the statistical analysis plan. Contamination between arms will be tracked by detection of patients with VL assessments at multiple providers in the year.

It should be noted that the existing surveillance procedures include extensive efforts to link cases across jurisdictions. Individuals moving outside the jurisdiction will be censored at last measure prior to relocation.

4.8.2 Sample Size and Power

The top 20 provider sites in Washington, D.C. and the Bronx account for approximately 5,000 HIV patients in Washington, D.C. and 16,000 in the Bronx (exclusive of incarcerated patients in HIV care). It is assumed that the number of cases in care during the 24-month study duration will be similar to these past case loads on an annual basis. In the Bronx, VL was suppressed in 57% of 8,316 patients with VL assessed in 2008. The mean number of patients per clinic with a VL assessment in 2008 in the top 20 care sites was 400, ranging from 50 to 3,300. The ICC of proportion virally suppressed estimated from the Bronx 2008 data was 0.07. In Washington, D.C., 67% of 2,926 patients with VL assessments in 2008 achieved viral suppression at their most recent assessment. The mean number of patients per clinic with VL assessments was 245, ranging from 35 to 1,400 in the top 20 clinics. The ICC of proportion virally suppressed estimated from the Washington, D.C. 2008 data was 0.104.

Table 3 shows the mean number of patients with VL assessments needed per clinic, assuming 20 clinics in each arm (40 clinics in total), to detect a change in proportion of virally suppressed for a range of effect, power and ICC. If the clinics had a mean of 219 patients with VL assessments in the 12 months of evaluation, we would have 90% power

to detect an increase in viral suppression from 60% to 66% between the SOC and intervention clinics.

Sample size calculation for clustered designs use the approach described by Thomson, Hayes and Cousens (Thomson, Hayes et al. 2009).

Table 3. Mean number of patients in care at a site required to achieve sufficient power to detect differences in proportion with suppressed VL. Number of clusters fixed at 20 per arm.

Percent in care virally suppressed		Standard Deviance of percent virally suppressed		Intra-class correlation	Mean number of patients in care	
Standard of care	Intervention	Standard of care	Intervention		80% power	90% power
60%	70%	15%	13%	0.094	57	76
60%	70%	16%	14%	0.105	62	83
60%	70%	18%	16%	0.135	78	104
60%	66%	15%	14%	0.094	163	217
60%	66%	16%	15%	0.105	179	219
60%	66%	18%	13%	0.135	199	299

4.8.3 Randomization Scheme

In the viral suppression study component, facilities that provide HIV care services (HIV care sites) will be randomized to use FIs or the SOC.

HIV care sites will be assigned to one of the following two arms:

- Sites offering FIs for VL suppression: FIs offered to all those who are on ART, upon the confirmation of each suppressed VL measurement (<400 copies/mL)

- Sites continuing with SOC to encourage VL suppression: Each person on ART will be offered support via the site's SOC procedures to attend HIV care site visits and remain adherent to their ART regimen in order to achieve and maintain VL suppression

The site randomization will be balanced by the following two baseline characteristics:

- The size of the site's HIV-positive patient case load

- The proportion of HIV-positive patients with VL suppression at each site

4.8.4 Data Analysis

Data to be analyzed for this study component will be obtained from routine HIV surveillance data as well as from the participating sites. Participating sites will maintain tracking logs for FIs provided to participants maintaining suppressed VLs. Information from the tracking logs, with all participant-identifying information removed, will be provided to the study team.

The data for the effectiveness of an FI intervention for high ART adherence to achieve viral suppression compared to the SOC will be analyzed as described below. Analysis of secondary endpoints will be detailed in the statistical analysis plan.

4.8.4.1 Primary Effectiveness Analysis

The assessment cohort includes all HIV cases identified from HIV surveillance in the intervention city DOH jurisdiction that have a VL assessment in the 24 months of trial duration. The assessment period for each site begins 12 months after the onset of FIs in each city. An intention-to-treat approach will be used to assign patients to the care site assignment existing at the trial onset. For the 12-month assessment period (*i.e.* from 12 through 24 months), all VLs in the surveillance data assessed by the 40 clinics in the study are used to assess differences in percent achieving viral suppression by study arm. Details about each site's contribution of patients who enter into care during the study will be provided in the statistical analysis plan. As described in 4.8.1.2, an imputation scheme for assessing VL suppression during the 12 months of the assessment that avoids the bias of missing assessments will be applied to all patients in care.

The estimate of intervention effect is the odds ratio of VL suppression in the FI intervention compared to SOC arms, assuming both a within-person and within-clinic correlation, stratified by city, using GEE methods. Sensitivity analysis will be conducted to assess the impact of patients who switch providers or establish care at multiple providers during the trial.

4.8.4.2 Feasibility Endpoints

Descriptive statistics will be used to summarize the mean uptake and overall cost of FIs and its variation between sites by site and population characteristics.

4.9 Human Subjects/Ethical Considerations

The Viral Suppression component of the study involves an intervention using FIs to encourage HIV-positive individuals on ART to be adherent to their medications and to maintain HIV VL suppression. This component is public health research. In this component, HIV care sites will be randomized, not individuals, and no de novo individual-level data will be collected. Instead, (de-identified) HIV surveillance data routinely collected by the DOH in the two communities will be analyzed. Because this research involves minimal risk and would be impracticable with informed consent, a waiver of patient informed consent will be requested under 45 CFR 46.116 (c) or (d).

The protocol will be submitted to appropriate IRBs (a central and/or local site IRBs) for ethical review prior to study initiation. Any subsequent modifications to the protocol will be submitted to appropriate IRBs, and, at a minimum, the protocol will be submitted annually for continuing review and approval by these same ethics boards.

No individual data, other than what exists in the surveillance databases, will be collected from study participants in the viral suppression component of this study. To assess the impact of the study on viral suppression in the communities of Washington, D.C. and the Bronx, surveillance data routinely collected by the DOHs will be analyzed. Data from the DOHs will be provided to the study team in a de-identified fashion, as per usual HIV surveillance procedures.

No study-specific laboratory testing will be conducted under this protocol. Therefore, no additional study-related test results will need to be reported to authorities. HIV testing and care data collected during routine clinical care will be reported per local HIV and AIDS reporting requirements.

4.10 Safety Monitoring and Adverse Event Reporting

The study team will not collect or report Adverse Events because there is no biomedical intervention. However, the team will collect and report all social harms that are brought to the attention of study staff members, using a study-specific incident report form. This form will be anonymous and will query common social harms such as altered personal relationships, forced change in housing, and physical violence. The form will also include space for a written narrative to document additional details of any social harm experienced. All research staff will be trained to properly complete the form. As a part

of study training, research staff will also be trained on the provision of referrals to counseling and social service support. Reports of social harms will be reviewed quarterly or more often, if indicated, and reported to the medical officer together with any actions that are taken. Social harms will be summarized and reported to appropriate IRB(s) on an annual basis.

5.0 PREVENTION FOR POSITIVES

5.1 Study Objectives for Prevention for Positives

The primary objective of the Prevention for Positives study component is to evaluate the effectiveness of a computer-delivered counseling intervention ("CARE+ Prevention") containing Prevention for Positives messages in addition to SOC compared to SOC alone. Both intervention and control groups will have access to available SOC support and counseling services available to HIV-positive patients at the participating HIV care sites. All participants will have regular assessment of risk behaviors. The objective for this study component will be measured by comparing key self-reported HIV-transmission sexual risk behaviors.

5.2 Design for Prevention for Positives

The Prevention for Positives component of the study is a two-arm, individually randomized, prospective, effectiveness trial to be conducted at 12 HIV care sites (6 within each intervention community). This component will compare CARE+ containing Prevention for Positives messages in addition to SOC with SOC alone. All consenting individuals will be randomly assigned to one of the two arms of the study. In this component of the study, participant informed consent will be obtained for all participants in both of the study arms. Limited medical records data pertaining to HIV disease and individual data will be abstracted and analyzed in this study component.

5.3 Study Population for Prevention for Positives

The Prevention for Positives component of the study will include HIV-positive patients at a select number of participating HIV care sites. These patients will include individuals newly diagnosed with HIV and linked to care, and those established in care. ART use is not required for participation.

5.3.1 Inclusion Criteria

The inclusion criteria for this study component are as follows:

- All individuals who are permitted to consent for HIV care according to New York State or Washington, D.C. law
- Receiving care at the selected HIV care sites in the Bronx or Washington, D.C.
- Have attended the clinic one or more times in the last seven months

- Able to understand either spoken English or Spanish
- Able and willing to provide informed consent

Subjects enrolled into the Prevention for Positives component of the study will participate in the patient survey (see Section 6.0).

5.3.2 Exclusion Criteria

The exclusion criteria for this study component are the following:

- Not seen in the clinic in the last seven months
- History or evidence of altered mentation, inebriation or substance use that would interfere with participation in the study
- Unable or unwilling to provide informed consent
- Participation in another study focusing on HIV prevention for positives

5.4 Study Sites for Prevention for Positives

Participants will be recruited for the Prevention for Positives component of the study from six HIV care sites (three FI sites and three SOC of care sites) in each intervention city.

The three sites among the FI sites and the three sites in the SOC sites in each city will be selected based on the following criteria:

- The highest volume of HIV-positive patients in care
- Site agreement to participate in this component of the study

5.5 Intervention for Prevention for Positives

A modified version of CARE+ for HIV-positives will be used with Audio Computer Assisted Self-Interviewing (ACASI) to measure behavioral risk and to compare a full computer counseling intervention (assesses self-efficacy and motivation, provides tailored feedback on specific risk behaviors, shows skill-building videos, and helps the user make a risk-reduction plan) to a control session consisting only of ACASI ascertainment of risk behavior.

An additional component will be the utilization of the computer-based system described above to measure factors that impact both the acceptability of the interventions utilized in this study and the feasibility of future TNT strategies. Other data, beyond those that can be obtained from routine HIV surveillance data, will be captured including the following:

- HIV testing history and prior experience with linkage/drop out from care (if applicable)

- CD4 cell count (via chart abstraction, not routinely available from HIV surveillance), at study enrollment, and then every 3 months up to and including month 12

- VL (via chart abstraction, not routinely available from HIV surveillance), at study enrollment, and then every 3 months up to and including month 12

CD4 cell counts and HIV RNA levels obtained from month 0 through month 12, will be extracted from participant's medical records to enable individual-level subgroup analyses of adherence and virologic responses among CARE+ participants who are receiving versus not receiving FIs for virologic suppression, as well as other subgroup analyses, such as:

- Behaviors likely to transmit HIV (sexual and parenteral)

- Receipt of and experience with services to promote retention and adherence

- Knowledge of, attitudes towards, and the feasibility of early initiation of ART

- Knowledge of and attitudes towards use of ART for prevention in partners

5.6 Study Procedures for Prevention for Positives

5.6.1 Recruitment Process

Study staff at the HIV care sites will be trained for the Prevention for Positives study procedures and on human subject requirements. Clinic or study staff will systematically approach potential subjects in the waiting room or during consultation and inform them of the study. Brief, anonymous demographic data will be noted for those individuals refusing study participation, to assess comparability to study participants. Persons who are interested in the study will be assessed for eligibility and consented in a private area of the clinic. The study staff will provide information to the participant on the use of the tablet computer and headphones, and assist the participant with the anonymous ID log-in and beginning of the session, after which the staff person will remain nearby only to provide assistance if the participant requests it.

5.6.2 Co-Enrollment Guidelines

Prevention for Positives study participants will be allowed to be enrolled in other studies except those focused on reducing the risk of HIV transmission.

5.6.3 Participant Retention

Once a participant enrolls in the Prevention for Positives component of this study, the research staff will make every effort to retain him/her for 12 months of follow-up in order to minimize possible bias associated with loss to follow-up.

5.6.4 Participant Withdrawal

Participants may voluntarily withdraw from the study for any reason at any time. Participants also may be withdrawn by the study sponsor, site IRBs, government or regulatory authorities, the Investigator of Record (after consultation with the Protocol Chair), or Division of AIDS (DAIDS) Medical Officer.

Participants who elect to withdraw from the study prior to month 12 will be asked to complete one last computer session, however, participants will not be required to do so. Study staff will record the reason(s) for all withdrawals from the study in participants' study records.

5.7 Study Duration for Prevention for Positives

Duration of the Prevention for Positives study component is 12 months.

Patients enrolled in the Prevention for Positives component of the study will complete the CARE+/ACASI tool at baseline (month 0) and every three months thereafter for one year (at months 3, 6, 9, and 12).

5.8 Safety Monitoring and Adverse Event Reporting

Physical and psychological risks of this study are expected to be minimal, as no medical/surgical or pharmacologic procedures are planned. The Prevention for Positives study component addresses sensitive behaviors around sex and social interactions. Questions regarding sex may cause embarrassment or discomfort. Participants will be informed in writing and verbally that they may skip any questions and/or drop out of the study at any time without repercussion. Mental stress identified in the course of the study, such as depression, suicidality and intimate partner violence (IPV) will be followed-up appropriately, with counseling services available through the clinics. Counseling services for any other issues may be requested by participants during their routine appointment at the HIV care site.

Researchers will do everything possible to emphasize and maintain the confidentiality of participants, and safeguards for protecting confidentiality of data will be strictly enforced.

The study team will not collect or report Adverse Events because there is no biomedical intervention. However, the team will collect and report all social harms that are brought to the attention of study staff members, using a study-specific incident report form. This form will be anonymous and will query common social harms such as altered personal relationships, forced change in housing, and physical violence. The form will also include space for a written narrative to document additional details of any social harm experienced. All research staff will be trained to properly complete the form. As a part of study training, research staff will also be trained on the provision of referrals to counseling and social service support. Reports of social harms will be reviewed quarterly or more often, if indicated, and reported to the medical officer together with any actions that are taken. Social harms will be summarized and reported to appropriate IRB(s) on an annual basis.

5.9 Statistics and Data Analysis for Prevention for Positives

5.9.1 Endpoints

The primary endpoint for the effectiveness of the Prevention for Positives component of the study is the following:

- The proportion of participants reporting any unprotected vaginal or anal sex the last time they had sex, evaluated for all partners and also separately for primary and non-primary partners.

The secondary endpoints for the effectiveness of CARE+ Positive Prevention component of the study are the following:

- Proportion of those who had unprotected vaginal or anal sex the last time they had sex with negative or unknown HIV status partners, evaluated for all partners and also separately for primary and non-primary partners.

- The frequency of participants reporting any unprotected vaginal or anal sex in the previous three months with non-primary partners.

- The number of different persons with whom the participant shared needles or works (including cookers and cottons) in the previous three months.

5.9.2 Accrual, Follow-up, and Sample Size

The primary behavioral endpoint of this study component is the proportion of patients reporting any unprotected vaginal or anal sex the last time they had sex. In the Strategies for Management of Antiretroviral Therapy (SMART) study, at 11.2% of visits participants reported any unprotected anal or vaginal sex in the previous two months. Using post-enrollment data; 5.4% of patient visits included a report of unprotected anal or vaginal sex with an HIV-uninfected partner in the previous two months.

Individual randomization will occur, potentially stratified by gender.

Using a 2-sided alpha of 5%, and calculating the sample size required to achieve 90% power to detect differences in percentages of participant visits where high-risk behaviors are reported in each arm, Table 4 presents a number of assumptions. A within-person, ICC of 0.3-0.4 is assumed, based on data from the SMART trial behavioral endpoints.

Assuming at least one follow-up visit for 90% of enrollees, and an average number of visits per participant of 3.75 amongst retained participants, the study will enroll 660 per arm (1320 in total) to allow for loss due to attrition and loss to follow-up. It is estimated that around 110 participants will be recruited per HIV care site, with a target enrollment of two subjects/day per site. Target enrollment should be reached within eight-12 months of initiation, and accrual will be terminated at 1320 participants or after approximately 12 months of accrual, whichever occurs first.

It is anticipated that a total of approximately 1320 individuals will be randomized. Data will be collected from each person an average of 4 times during the study (out of four possible visits following enrollment at month 0, *i.e.* months 3, 6, 9, and 12). Allowing for

loss to follow-up, with 522 people available for assessment per arm, the study has 90% power to detect a decrease from 11% to 8% in proportion of patients reporting any unprotected vaginal or anal sex at a given visit during the study.

Table 4: Per-Arm Sample Size Required for 90% Power to Detect Difference in Proportion Reporting High Risk Behavior at a Visit, with Two-sided Alpha of 5%, Assuming Four Visits per Participant

Control (%)	Intervention (%)	Mean Number of Visits	Intra-class Correlation	Number of People per Arm	Intra-class Correlation	Number of People per Arm
5.4%	3.0%	4	0.4	378	0.3	373
5.4%	3.5%	4	0.4	638	0.3	630
5.4%	4.0%	4	0.4	1240	0.3	1223
7.5%	4.0%	4	0.4	243	0.3	238
7.5%	4.5%	4	0.4	344	0.3	337
7.5%	5.0%	4	0.4	515	0.3	505
11%	7.5%	4	0.4	385	0.3	374
11%	8.0%	4	0.4	537	0.3	522
11%	8.5%	4	0.4	792	0.3	769

5.9.3 Randomization Scheme

Randomization to intervention (SOC and full CARE+ Prevention counseling session) or control arm (SOC alone) is done automatically within the software application using a pseudo-random number generator.

5.9.4 Data Analysis

Data to be analyzed for this study component will be obtained from the CARE+ electronic storage system and abstracted from participant's medical records. As participants proceed through the CARE+ intervention, their answers will automatically be stored electronically in a secure system. Access to the storage system will be limited to essential personnel. CD4 cell counts and HIV RNA levels obtained from month 0 through month 12, will be extracted from participant's medical records to enable individual-level subgroup analyses of adherence and virologic responses among CARE+ participants who are receiving versus not receiving FIs for virologic suppression, as well as other subgroup analyses.

The primary behavioral endpoint of this study component is the proportion reporting any unprotected vaginal or anal sex the last time they had sex.

Effectiveness of the CARE+ Prevention for Positives component will assess differences in proportion reporting self-reported risk behavior for participants in the CARE+ Prevention arm compared to the control arm. GEE methods for repeated measures will be used to assess the difference in proportion reporting high-risk behavior between the two study arms for participants. Major subgroup analyses for those on ART and not on ART will be conducted.

5.10 Human Subjects/Ethical Considerations

In the Prevention for Positives component of the study, a subset of patients enrolled at select HIV care sites in the two intervention communities will be randomized either to an intervention arm (receiving SOC prevention activities plus a computer-delivered intervention) or to the control arm (receiving only the SOC prevention activities at the care site). Individual-level data will be collected and analyzed. This component is a public health research requiring informed consent. Written informed consent will be obtained from participants.

The protocol and supporting study materials (the informed consent form and any advertising materials) will be submitted to appropriate IRBs (a central and/or local site IRBs) for ethical review prior to study initiation. Any subsequent modifications to these materials will be submitted to appropriate IRBs, and, at a minimum, they will be submitted annually for continuing review and approval by these same ethics boards.

Written informed consent will be obtained from each study participant (or a mark for those who are illiterate, which will be witnessed by a third party) prior to study enrollment. Each study site is responsible for developing an informed consent form for local use, based on the template provided with this protocol that describes the purpose of

the study, the study procedures and the risks and benefits of participation, in accordance with all applicable regulations. Participants will be provided with a copy of their informed consent form if they wish to receive it.

A small incentive will be provided to study participants in the Prevention for Positives component to compensate them for transportation and time.

All study-related information will be stored securely at the study site in areas with access limited to study staff. To maintain participant confidentiality, a coded number will identify all study data and administrative forms. All local databases will be secured with password-protected access systems. Forms, lists, logbooks, appointment books and any other listings that link participant ID numbers to other identifying information will be stored in a separate area with limited access. A participant's study information will not be released without the written permission of the participant, except as necessary to: authorized medical care providers; monitors for the National Institute of Allergy and Infectious Diseases (NIAID) and/or its contractors; representatives of the HPTN Coordinating and Operations Center (CORE) and/or the (Statistics and Data Management Center) SDMC; other government and regulatory authorities; and/or the site IRB.

No study-specific laboratory testing will be conducted under this protocol. Therefore, no additional study-related test results will need to be reported to authorities. HIV care data collected during routine clinical care will be reported per local HIV and AIDS reporting requirements.

6.0 SURVEY OF PATIENTS AND PROVIDERS

6.1 Study Objectives for the Survey of Patients and Providers

The objectives of the survey of patients and providers study component are the following:

- To assess knowledge, attitudes and practices with regard to ART initiation, the potential of starting ART at higher CD4 cell counts, and the use of ART for prevention of HIV transmission
- To ascertain key socio-demographic and behavioral characteristics of study participants that cannot be obtained from routine HIV surveillance data
- To assess the acceptability and attitudes towards FIs

6.2 Design for the Survey of Patients and Providers

All participants in the Prevention for Positives component of the study will complete a patient survey module prior to and at the end of that study component. The survey will assess knowledge and attitudes regarding the use of ART in HIV disease and other key factors related to the feasibility and acceptability of the study's interventions.

Clinical providers who prescribe ART at HIV care sites in the intervention communities will be invited to complete a survey regarding their knowledge, attitudes and practices about the use of ART in HIV patients as well as FIs. Clinical providers will be surveyed

prior to and following the viral suppression intervention to assess trends in the provider knowledge, attitudes and practices.

6.3 Study Population for the Survey of Patients and Providers

The patient survey component of the study will include HIV-positive patients at select HIV care sites that are participating in the Prevention for Positives component of the study.

Prescribing clinical providers (*e.g.* physicians, nurse practitioners and/or physician assistants) at select HIV care sites in the Bronx and Washington, D.C. will participate in provider survey.

6.3.1 Inclusion Criteria

The inclusion criteria for the patient survey are identical to those for the Prevention for Positives component of the study.

Prescribing clinical providers (*e.g.* physician, nurse practitioner/nurse-midwife, or physician assistant) at select HIV care sites are eligible for the provider survey.

6.3.2 Exclusion Criteria

The exclusion criteria for the patient survey are identical to those for the Prevention for Positives component of the study.

There are no exclusion criteria for the provider survey.

6.4 Study Sites for the Survey of Patients and Providers

The patient survey will be conducted at those HIV care sites involved in the Prevention for Positives component. The provider survey will survey providers from participating HIV care sites in the Bronx, NY and Washington, D.C.

6.5 Study Procedures for the Survey of Patients

Once consented and enrolled in the Prevention for Positives component, the clinic study staff will train participants how to use the computer tablet. Randomization to intervention (SOC plus full CARE+/ACASI counseling session) or control arm (SOC alone) is done automatically within the software application following the user's login with an anonymous study ID. Up to 110 individuals will be assigned to each arm, per site. At the end of the first (baseline, month 0) session, both arms will be presented, via the computer tool, with a series of questions (the patient survey). This patient survey module will be repeated only one more time, at the end of the intervention (month 12).

6.6 Study Procedures for the Survey of Providers

Providers at participating HIV care sites in both cities will be recruited by an introductory letter followed by up to two e-mail reminders, then two phone calls by study staff, asking them to take the brief survey on a secure, anonymous website.

Providers who go to the Web site will read and click on a brief consent, then complete a short survey assessing attitudes and practices.

At the completion of the survey, a printable coupon, compensating them for their time will be generated.

6.7 Study Duration for the Survey of Patients and Providers

Participants in the patient survey will complete the survey upon enrollment in the Prevention for Positives study and at the end of that study component.

The provider survey will also take place twice, once at the time of initiation of the overall HPTN 065 study and at the end of the viral suppression component.

6.8 Statistics and Data Analysis for Surveys of Patients and Providers

Data for analysis of this study component will be derived from answers to survey questions provided by both the patients and providers. Patient surveys will be administered electronically, after the CARE+ intervention at the beginning and at the end of the intervention. All patient responses will be stored in an electronic and secure database. Limited personnel will have access to this database. Provider surveys will be administered over the Internet. The survey will be secure and responses will also be stored securely. Survey data will be provided to the Statistical Center for HIV/AIDS Research and Prevention (SCHARP) for the following analyses:

- To assess baseline knowledge, attitudes and practices regarding the use of ART and other key factors related to the feasibility and acceptability of the study interventions in the subset of survey participants in the Prevention for Positives study component

  Descriptive statistics will be used to summarize the survey participants' attitudes regarding the use of ART and other key factors related to the feasibility and acceptability of the study interventions by site and combined.

- To compare the providers' knowledge, attitudes and practices about the use of ART, assessing these at study baseline and at study end at HIV care sites in the intervention communities

  Descriptive statistics will be used to summarize the prescribing providers' knowledge, attitudes and practices about the use of ART, at study baseline and study end by site and combined. Chi-squared statistics will be used to compare the baseline data with study end data.

All Prevention for Positives data collected on the tablet computer will be password security-protected (anonymous study ID assigned timed to the millisecond of initial user log-in, with no way for patients to access other user's data). All study data will be accessible only to investigators, study staff and biostatisticians assisting with the analysis. The CARE+ Prevention application will reside on each tablet computer with encrypted data transferred to a secured server at each clinic. The session database will be backed up by study staff. The 12 HIV care sites will send their study data to a SCHARP server on a routine basis. Only authorized study staff members are able to log onto the tablet computers or to access the master study database.

6.9 Human Subjects/Ethical Considerations

All patients participating in the Prevention for Positives study component will be administered the Patient Survey, regardless of whether or not they are randomized to the CARE+/ACASI intervention. Individual-level data will be analyzed. This component is human subjects research. Written informed consent will be obtained from participants at the time of consenting for participation in the Prevention for Positives study component.

Providers from participating HIV care sites will be invited to complete a Web-based survey regarding their knowledge, attitudes and practices concerning ART for treatment, frequency of HIV testing, and limited sociodemographic information. The survey will be anonymous, and will collect no identifying information. This component is public health research. Informed consent will be obtained from the provider's completing the survey. Before the survey will display, providers will read the consent and if they agree to participate, will indicate their agreement by clicking a button labeled "I agree."

The protocol and supporting study materials (the informed consent form and any advertising materials) will be submitted to appropriate IRBs (a central and/or local site IRBs) for ethical review prior to study initiation. Any subsequent modifications to these materials will be submitted to appropriate IRBs, and, at a minimum, they will be submitted annually for continuing review and approval by these same ethics boards.

Written informed consent (or a mark for those who are illiterate, which will be witnessed by a third party) will be obtained from each study participant willing to complete the patient survey prior to study enrollment. The informed consent for the survey is incorporated into the consent form for the Prevention for Positives component. Each study site is responsible for developing an informed consent form for local use for the patient survey, based on the template provided with the protocol, that describes the purpose of the survey, the survey procedures, and the risks and benefits of participation, in accordance with all applicable regulations. Participants completing the patient survey will be provided with a copy of their informed consent form if they wish to receive it.

No physical or psychosocial risks are anticipated from participation in the patient or provider surveys as they involve no invasive procedures or reports of illegal or socially stigmatized behaviors. Further, all data will be anonymized once merged into the study database, and no names will be used in publications.

A small incentive will be provided to survey participants, both providers and patients, to compensate them for their time. For the patients, this compensation is included in

the incentive they receive for participating in the Prevention for Positives component of the study.

For patient surveys, all study-related information will be stored securely at the study site in areas with access limited to study staff. To maintain participant confidentiality, a coded number will identify all study data and administrative forms. All local databases will be secured with password-protected access systems. Forms, lists, logbooks, appointment books and any other listings that link participant ID numbers to other identifying information will be stored in a separate area with limited access. Survey information will not be released without the written permission of the participant, except as necessary to: authorized medical care providers; monitors of the NIAID and/or its contractors; representatives of the HPTN CORE and/or the SDMC; other government and regulatory authorities; and/or the site IRB.

6.10 Safety Monitoring and Adverse Event Reporting

The study team will not collect or report Adverse Events because there is no biomedical intervention. However, the team will collect and report all social harms that are brought to the attention of study staff members, using a study-specific incident report form. This form will be anonymous and will query common social harms such as altered personal relationships, forced change in housing, and physical violence. The form will also include space for a written narrative to document additional details of any social harm experienced. All research staff will be trained to properly complete the form. As a part of study training, research staff will also be trained on the provision of referrals to counseling and social service support. Reports of social harms will be reviewed quarterly or more often, if indicated, and reported to the medical officer together with any actions that are taken. Social harms will be summarized and reported to appropriate IRB(s) on an annual basis.

7.0 HIV SURVEILLANCE, ROUTINELY-COLLECTED AND OTHER SURVEY DATA

Use of HIV case and behavioral surveillance data and HIV testing data will be used for site selection in intervention communities and for process and outcome measures. Other data, including site provided data, may be utilized for these purposes in certain circumstances.

We will assess completeness, accuracy, timeliness and performance of the surveillance and any other data.

7.1 HIV Testing Data

HIV testing data will be used to select HIV test sites for randomization to the intervention vs. SOC arm, and as a process measure for the testing intervention in ED and inpatient facilities (Table 5). For selection of HIV test sites for interventions, information on HIV testing is available for HIV test sites that are publicly supported through federal funds in each of the six communities included in this study; additional data are available for other

HIV test sites supported in some local jurisdictions, such as NYC's AIDS Institute Reporting System. This includes the majority of HIV testing conducted by CBOs.

Table 5. Data Sources and Definitions

**Data Sources and Definitions**			
**Information needed**	**Description**	**Data source**	**Variables**
**HIV Testing Data**			
**Site selection, intervention communities**			
Volume of testing	Number of persons tested in XX year, by testing site	Local PEMS, local testing data	Name of testing facility, testing date
	Number of new diagnoses, by testing site	HIV surveillance data	Name of facility of diagnosis, date of diagnosis
	Where available, number of case reports to surveillance, by testing site	HIV surveillance data	Name of facility of diagnosis, date of diagnosis
**Process measures for HIV testing interventions, intervention communities**			
Number of persons eligible	Number of persons eligible	Project specific collection at intervention sites	Data may or may not already be collected at selected sites. If not collected, collection will be implemented for this study
Number of persons tested	Number of persons tested	Project specific collection at intervention sites	Data may or may not already be collected at selected sites. If not collected, collection will be implemented for this study
Number of new diagnoses	Number of new diagnoses	HIV surveillance data, and project specific collection at intervention sites	Surveillance data: name of testing facility, testing data. Data may or may not be collected by selected sites, and if not collected, can be implemented at site or use surveillance data.
**HIV Surveillance Data**			
**Outcome Measures**			
Number/proportion of persons newly diagnosed who entered care, by site	CD4 or VL within 3 months of diagnosis (CD4/VL date not equal to diagnosis date), by site	HIV surveillance data	HIV diagnosis date, name of testing site, VL date, VL result, CD4 date, CD4 result, demographics (DOB, sex, race/ethnicity, transmission category)

Data Sources and Definitions			
**Information needed**	**Description**	**Data source**	**Variables**
Number/proportion of previously diagnosed persons not in care entered into care, by site	CD4 or VL within 3 months of diagnostic test, among persons with previous diagnostic test and no CD4/VL within the past year, by site	HIV surveillance data	HIV diagnosis date, name of testing site, VL date, VL result, CD4 date, CD4 result, demographics (DOB, sex, race/ethnicity, transmission category)
VL suppression	Probability of undetectable VL amongst people established in care with previous VL/CD4, by site	HIV surveillance data	Name of treatment site, VL date, VL result, CD4 date, CD4 result, demographics (DOB, sex, race/ethnicity, transmission category)
**Behavioral Data**			
Number/proportion of persons who had an HIV test in the past 12 months	See BRFSS 2009 questionnaire: 20.1 Have you ever been tested for HIV? Do not count tests you may have had as part of a blood donation. Include testing fluid from your mouth. (213) 1 Yes 2 No [Go to Q20.5] 7 Don't know / Not sure [Go to Q20.5] 9 Refused [Go to Q20.5] 20.2 Not including blood donations, in what month and year was your last HIV test? http://www.cdc.gov/brfss/questionnaires/pdf-ques/2009brfss.pdf	Local surveys (see descriptions below)	Testing, testing date
Special HPTN 065 emphasis population: number/proportion of MSM who had an HIV test in the past 12 months	NHBS questions	National HIV Behavioral Surveillance (NHBS) system	Testing, testing date

The CDC has developed a data entry and reporting system, the PEMS, to strengthen monitoring and evaluation of HIV prevention programs. PEMS is used by health departments and CBOs funded through CDC HIV-prevention cooperative agreements. PEMS allows grantees to collect agency data, community planning data, program plan data, and client-level data. This assures a comprehensive set of standardized variables are available.

The client-level data include information on testing site, client demographics, risk factors, test information and testing history. This allows a description, by site, of the number

tested and number positive, and a description of the population tested or positive. Additional variables that can be used to describe sites include agency characteristics (budget, sites, workers, contracts, network agencies), program plans (program models, target populations, interventions (CTR, Health Communications/Public Information Outreach, settings, sessions, activities), service delivery (service activities, recruitment, referrals), and community planning activities (target populations, priority interventions).

Some areas have testing report systems in addition to PEMS. For example, New York State collects testing data through the AIDS Institute Reporting System. Florida has PEMS data as well as a counseling and testing data base that includes all the variables needed to report an HIV case as well as testing history questions; these two data sources cover all clients tested in the public sector, *e.g.*, registered counseling and testing sites (AIDS Service Organizations (ASOs), CBOs, faith-based initiatives), and county health department clinics (STD, TB, Family planning, *etc*.).

Another testing measure for selection of intervention sites that can be derived from surveillance data is the number of new diagnoses by site.

Process measures (Table 5) to monitor testing at the testing intervention sites will be collected directly from the participating facilities.

7.2 HIV Surveillance Data

The CDC's national system for the surveillance of HIV infection is based on mandatory name-based reporting of all HIV and AIDS cases in every state and Washington, D.C. Health departments maintain case records of every new and established HIV case according to the HIV case definition. Laboratory reports of positive HIV test results and provider reports of new confirmed diagnoses are tracked by the health departments. In most jurisdictions, data from mandatory reporting of CD4 cell count and VL from all laboratories are linked via case names to individuals maintained in the eHARS data entry and reporting system (not all states have mandatory reporting of all VL and CD4 cell count). Across the United States, and within each community, the system captures key variables for this study. The key data elements from HIV surveillance that will be used are listed in Table 6.

Table 6. Key Date Elements and Primary Use

Key Data Elements	Primary Use
CD4 result<br>CD4 date<br>VL result<br>VL date<br>Name of facility of diagnosis<br>Name of treatment facility (lab order)<br>Type of HIV test<br>HIV test date<br>HIV test result<br>HIV diagnosis date<br>AIDS diagnosis date	Study intervention outcome measures

Key Data Elements	Primary Use
Date of birth (DOB)<br>Race/ethnicity<br>Transmission category<br>Sex at birth	Stratification variables
Date of death<br>Cause of death<br>Previous negative test, lab based<br>Previous negative test, self-reported	Additional outcome measures
Place of residence at diagnosis<br>Current place of residence	Adjustment variables
Source of report<br>Surveillance method<br>Duplicate	Variables for data quality control

De-duplication (removing double-reported cases) of cases between jurisdictions occurs at least annually, with case tracing on an ongoing basis as individuals relocate and new diagnostic labs are investigated in a new jurisdiction. Cases are identified through active follow-up of positive Enzyme Immunoassay (EIA), positive WB and detectable VL reports from names not matched in the local eHARS database, as well as through reporting by providers. Communities selected for this study (both intervention and non-intervention communities) have quality data collected on these parameters.

HIV surveillance data can describe treatment success based on reported VL measurements. However, specific information on type of treatment received is not collected.

HIV surveillance data have been evaluated on the national level and for individual state/local program areas for completeness, timeliness, and quality of individual data items (Buehler, Berkelman et al. 1992; Rosenblum, Buehler et al. 1992; Greenberg, Hindin et al. 1993; Meyer, Jones et al. 1994; Klevens, Fleming et al. 1998; Schwarcz, Hsu et al. 1999; Solomon, Flynn et al. 1999; Jara, Gallagher et al. 2000; Doyle, Glynn et al. 2002; Hall, Song et al. 2006) and through annual progress reports [unpublished]). For the accuracy of case counts, all surveillance programs conduct annual linkage of HIV cases to death records, intrastate duplicate reviews, and interstate duplicate resolution based on potential duplicate listings received from CDC.

CDC sets standards for the completeness and timeliness of case reporting as well as the completeness and accuracy of individual data elements, against which national and individual program data are evaluated each year (e.g., 2008 diagnosis data are currently evaluated using data reported through December 2009) (Hall and Mokotoff 2007; Hall, Song et al. 2008). For 2007 diagnoses, New York City, Philadelphia, and Florida case counts were estimated to be >95% complete by December 2008 (note, while the other cities were transitioning to the new data system and completeness was not formally assessed, they closely monitor expected case reports from laboratories and providers to assure complete reporting). More than 80% of cases are reported to the health departments within 6 months of diagnosis. Critical variables include sex assigned at birth, race/ethnicity, and age; completeness in these variables is very high (near 100% for sex and age; >90% for race/ethnicity) and active follow up is conducted to obtain these data elements; all cases must have a diagnosis date (test result or physician diagnosis,

according to the CDC case definition). Additional variables of importance include risk factor information, and CD4 and VL test results. Risk factor information is available for more than 60% of cases (some areas achieve better than 80%); CDC has developed methods to adjust for missing risk factor information in local or national analyses using multiple imputation.

The completeness of CD4 and VL results, critical to this study, depends on 1) state laboratory reporting laws and regulations; 2) reporting/abstracting from providers; and 3) whether patients entered care. Most important to the completeness of these data is the assessment of whether all laboratories routinely report to the health departments to assure that this information is reported for any patients entering care. For example, both New York State and DC have laws requiring reporting of all values of CD4 and VL test results. The New York State Health Department has identified all labs conducting testing for New York residents and monitors all labs to assure that reporting is complete and there are no missing data. Accuracy is also assessed by comparing reports from labs against what is obtained from chart review and re-abstraction. Regarding lag times, the mean time for New York City receiving Western Blot lab results is 21 days from diagnosis and CD4 and VL results 30 days from test date. (Please see Appendix for NYC example of additional indicators and evaluation of reporting completeness and timeliness). In DC, similar processes have been implemented. Regarding lag times in DC, approximately 85% of District laboratory reports are received within 2 weeks of the test date via electronic reporting. The remaining 15% are reported via the US mail, averaging about a 3 week lag in reporting. (Please see Appendix for DC indicators).

Data quality is continuously monitored during surveillance activities. Any notification (e.g., HIV diagnostic lab report) initiates data abstraction by field staff, with assignments within days of report. Abstracted data is checked for quality and the data entry system includes data quality checks. In order to assist in the timely input and generation of surveillance data during this study, resources will be provided for additional personnel in the DOHs in all participating cities (intervention and non-intervention). As part of TLC Plus, both process and outcome evaluations for data quality (accuracy, completeness, and lag times) will be conducted for intervention and non-intervention cities on a routine schedule.

CDC funds the Morbidity Monitoring Project (MMP) (McNaghten, Wolfe et al. 2007), an interview and medical-record-abstraction project, to obtain information on the following questions: Are patients receiving care and treatment in accordance with United States Public Health Service (USPHS) guidelines? Are patients receiving care in Ryan White-funded facilities receiving the same quality of care as patients in private facilities? What are the barriers to receiving care and services? Included is a locally and nationally representative sample of HIV-positive adults in care .with assessments of: adherence; sexual behavior; drug use; care-seeking; clinical outcomes; treatment; CD4 cell count and VL; opportunistic illnesses; type and quality of care received; and met and unmet needs for HIV care and prevention services. This information is limited in scope for the purposes of this study (funded locations, sites included, *etc*). However, an assessment will be made to determine the information that can be gained from MMP.

7.2.1 Human Subjects/Ethical Considerations

HIV case surveillance data are collected as part of routine HIV surveillance as mandated by state or local laws or regulations. Similarly, other data are routinely collected by DOHs.

According to CDC's Guidelines for Defining Public Health Research and Public Health Non-Research (CDC 1999) and Title 45 Part 46 of the Code of Federal Regulations (DHHS 2005), the CDC has determined that HIV surveillance is not a research activity, and, therefore, does not require review by an IRB.

State and local HIV surveillance programs must comply with federal security and confidentiality guidelines for collecting, storing and releasing data (CDC 2006).

Data are sent to the CDC without personal identifiers.

7.3 Behavioral Data

Information on population HIV-testing rates is generally available for the United States as a whole and for select communities that have implemented behavioral surveys that are representative for their community. The primary measures are the proportion of persons who ever tested for HIV and the proportion of persons who tested for HIV within the past year. This information is critical to monitor trends of HIV testing in the future.

One behavioral survey conducted in all states and Washington, D.C. is the Behavioral Risk Factor Surveillance System (BRFSS) funded by CDC (CDC, http://www.cdc.gov/BRFSS/). BRFSS is an on-going, telephone, health survey system, tracking health conditions and risk behaviors in the United States yearly since 1984. Currently, data are collected monthly in all 50 states, Washington, D.C., Puerto Rico, the United States Virgin Islands and Guam. State and local areas may add questions to the standard questionnaire. However, oversampling of local areas is needed to make inferences for areas smaller than statewide, and representativeness/response rates have diminished with the widespread use of cell phones. Surveys conducted in the local areas are described in Table 5. One limitation of BRFSS surveys is the diminishing population reached using land-lines; therefore, any BRFSS-type survey used for this project should include cell phones.

Our approach for this study will be to support currently planned surveys to obtain representative HIV testing data from the communities.

Accuracy of recall of HIV testing will not be assessed as part of this study.

This study also includes a special emphasis population, MSM. The National HIV Behavioral Surveillance (NHBS) system is a CDC-funded project conducted in 25 cities in the United States, including Washington, D.C. The purpose of this serial cross-sectional study is to yield information about what people do that puts them at risk for HIV. NHBS has three cycles focusing on different risk groups: NHBS-MSM, injecting drug users (NHBS-IDU), and heterosexuals at risk of HIV infection (NHBS-HET).

Areas conducting NHBS will be able to obtain HIV testing data among MSM in the MSM cycle years.

7.3.1 Behavioral Data Collection in Washington, D.C.

Washington, D.C. implements HIV testing questions through the BRFSS and will include cell phones in 2009. Washington, D.C. also collects behavioral data via NHBS.

7.3.2 Behavioral Data Collection in New York City

NYC conducts the NYC Community Health Survey (CHS), a telephone survey conducted annually. CHS provides robust data on the health of New Yorkers, including neighborhood, borough and citywide estimates on a broad range of chronic diseases and behavioral risk factors.

The CHS is based on the BRFSS. The CHS is a cross-sectional survey that samples approximately 10,000 adults aged 18 and older from all five boroughs of NYC—Manhattan, Brooklyn, Queens, Bronx and Staten Island.

A computer-assisted telephone interviewing (CATI) system is used to collect survey data from respondents accessed by random-digit dialing of household-based land lines. Interviews are conducted in a variety of different languages.

All data collected are self-report.

Questions on HIV testing are included. NYC also collects behavioral data via NHBS.

7.3.3 Behavioral Data Collection in Miami

In Miami, behavioral data is collected via NHBS and BRFSS. BRFSS collected county data in 2007, but not in 2008 and 2009. County data will be collected again in 2010. However, representativeness for Miami and inclusion of cell phones need to be determined.

7.3.4 Behavioral Data Collection in Philadelphia

In Philadelphia, behavioral data is collected via NHBS and BRFSS/Southeastern Pennsylvania Household Health Survey (PHHS).

Public Health Management Corporation's (PHMC) Southeastern Pennsylvania Household Health Surveys are extensive health surveys that provide timely information on more than 13,000 residents, both children and adults, living in Bucks, Chester, Delaware, Montgomery and Philadelphia counties. The survey targets key information about health status, personal health behaviors, and access to and utilization of area health services. These data are available at the census tract, ZIP code, county and regional level. The Household Health Survey provides primary data on a broad range of health topics such as health status, access to care, utilization of services, personal health behaviors, health screening information, health insurance status, women's health, child health, and older adult health and social support needs. The survey asks whether persons

have had an HIV test. The survey includes responses from more than 10,000 households in the region, representing more than 13,000 adults and children.

Interviews are conducted by telephone using a random-digit dial methodology. Adult and child respondents are selected randomly using the "last birthday" method.

7.3.5 Behavioral Data Collection in Chicago

In Chicago, behavioral data is collected via NHBS. The state conducts BRFSS and data is available for Chicago. However, representativeness for Chicago and inclusion of cell phones need to be reviewed.

7.3.6 Behavioral Data Collection in Houston

In Houston, behavioral data is collected via NHBS. The state conducts BRFSS. However, representativeness for Houston and inclusion of cell phones need to be determined.

8.0 ADMINISTRATIVE PROCEDURES AND OPERATIONAL CONSIDERATIONS

This study differs from other HPTN studies to date. In fact, it differs from most other studies conducted through HIV clinical trials networks. It is an ambitious effort that aims at identifying a strategy to tackle the entrenched HIV epidemic in some communities in the United States. It differs from traditional clinical trials in the following manners:

- The study will be a collaborative effort among the HPTN, the CDC, and the DOHs in the intervention and non-intervention communities.
- Many study outcomes will be based on data collected through surveillance systems in the public health programs of local health departments in the intervention and non-intervention communities.
- The study will include two site randomizations (one of HIV test sites and one of HIV care sites) as well as individual randomization in the Prevention for Positives cohort.
- The laboratory outcomes in this study will be based on standardized assays conducted in certified laboratories in the United States. No laboratory testing will be conducted at the HPTN Network Laboratory (NL).

The SDMC at SCHARP will assist in the design of the study, determine the sampling plan and coordinate the data collection from various sources. The SDMC will play the key role in communicating with staff responsible for surveillance in the jurisdictions where the study will be conducted. The SDMC will also lead data analysis efforts and generation of data reports.

The NL will assist in the design of the study as well as in guiding the conceptualization and selection of appropriate laboratory objectives and endpoints. The NL will provide expert input into the methodology for various assays, as needed. However, no assays will

be conducted at NL in the main part of the study. All laboratory data will be collected through surveillance systems or medical records abstraction (for the Prevention for Positives study component).

The HPTN CORE at Family Health International (FHI) will coordinate the development of the protocol and facilitate the implementation of the study in the intervention communities.

8.1 Study Activation

For this study, traditional DAIDS site activation and protocol registration procedures will not be conducted for the following study components: Expanded HIV Testing, Linkage-to-Care, Viral Suppression, and Provider Surveys. The study team will define a start date for the Expanded Testing component. The HPTN CORE will notify all participating HIV test sites when they may begin to distribute coupons to patients for the Linkage-to-Care study component and when participating HIV care sites may begin to distribute FIs for the Viral Suppression component.

Protocol registration and modified DAIDS site activation will be conducted for the following study components: Prevention for Positives and Patient Surveys. Traditional participant informed consent will be obtained for the Prevention for Positives component and for the Patient Survey. Operationally, the Prevention for Positives intervention and the Patient Survey will be administered together during Month 0 and Month 12 of the Prevention for Positives study component. Therefore, a single comprehensive informed consent form was designed (see Appendix IIA). Following ethical review and approval, study sites will submit required administrative documentation — as listed in the study-specific procedures manual — to the HPTN CORE. CORE staff will work with study site staff and complete DAIDS protocol registration in accordance with the current DAIDS Protocol Registration Policy and Procedure Manual. Included in this step will be CORE review of each site-specific study informed consent form. Site-specific informed consent forms (ICFs) *WILL NOT* be reviewed and approved by the DAIDS PRO and sites will receive an Initial Registration Notification from the DAIDS PRO that indicates successful completion of the protocol registration process. A copy of the Initial Registration Notification should be retained in the site's regulatory files. Pending successful protocol registration and submission of all required documents, CORE staff will "activate" the site to begin study operations. Study implementation may not be initiated until a study activation notice is provided to the site.

The HPTN Core will also notify SCHARP and other network partners of dates that study components will start.

8.2 Study Coordination

Study implementation will be directed by this protocol as well as the SSP manual. The SSP manual will outline procedures for conducting study visits, data collection and processing, management and reporting, and other study operations.

Data for this study will be collected in three ways:

- The DOHs in Washington, D.C. and in the Bronx of NYC will provide aggregate data from their routine surveillance systems to the data management group for the study, SCHARP. SCHARP will conduct all data analyses.
- Data will also be collected from patients and providers using study-specific surveys. These data will also be submitted to SCHARP for analysis.
- For the Prevention for Positives study component, data will be collected by patient self-report and medical records abstraction. These data will be submitted to SCHARP.

Close coordination between protocol team members will be necessary to track study progress, respond to questions about proper study implementation and address other issues in a timely manner. Rates of accrual, adherence and follow-up will be monitored closely by the team as well as an HPTN study-monitoring committee (SMC) for the Prevention for Positives and survey components of the study.

8.3 Study Monitoring

This study will not undergo traditional HPTN monitoring by PPD Research Associates. However, the study will be carefully reviewed by an HPTN SMC and/or external monitoring group on a regular basis. Study elements that will be monitored include data quality, achievement of milestones, and magnitude of utilization of study test and care sites, as well as migration of patients between test and care sites based on their randomization assignments.

8.4 Protocol Compliance

The study will be conducted in full compliance with the protocol. The protocol will not be amended without prior written approval by the Protocol Co-Chairs and NIAID Medical Officer. All protocol amendments must be submitted to and approved by the relevant IRB(s) prior to implementing the amendment. Upon receiving final IRB approval for all protocol amendments, including Letter of Amendments [LoAs] and full protocol amendments, sites should implement the amendment immediately.

For the Prevention for Positives and Patient Survey study components, sites are required to submit an amendment (LoA or full amendment) registration packet to the DAIDS PRO at the RSC. HPTN CORE staff will work with study site staff to complete this procedure. Site-specific ICF(s) WILL NOT be reviewed and approved by the DAIDS PRO and sites will receive an LoA or Amendment Registration Notification from the DAIDS PRO that indicates successful completion of the amendment protocol registration process. A LoA or Amendment Registration Notification from the DAIDS PRO is not required prior to implementing the LoA or full amendment. A copy of the final LoA or Amendment Registration Notification issued by the DAIDS PRO should be retained in the site's regulatory files.

For additional information on the protocol registration process and specific documents required for initial and amendment registrations, refer to the current version of the DAIDS Protocol Registration Manual.

8.5 Human Subjects/Ethical Considerations

Prior to implementation of this protocol, and any subsequent full version amendments, each site must have the protocol and the protocol consent form(s) approved (for Prevention for Positives study component only), as appropriate, by their local institutional review board (IRB).

Expanded HIV testing. This component is public health practice. Social mobilization and emergency department testing is already taking place in the two intervention communities (Bronx and Washington DC) and is intended to be specific to the needs of those populations. While some observations from the overall TLC Plus project with regard to social mobilization and expanded testing may be applicable elsewhere in the United States, these activities were initiated originally for the specific benefit of the respective communities, and do not constitute research.

Linkage-to-Care. This component of the study involves a randomized intervention using financial incentives (FIs) to link HIV-positive individuals to HIV care after diagnosis. This component is public health research. In this component, HIV test sites will be randomized, not individuals, and no de novo individual-level data will be collected. Instead, (de-identified) HIV surveillance data routinely collected by the Departments of Health (DOH) in the two communities will be analyzed. Because this research involves minimal risk and would be impracticable with informed consent, a waiver of patient informed consent will be requested under 45 CFR 46.116 (c) or (d).

Viral Suppression. This component of the study involves an intervention using FIs to encourage HIV-positive individuals on ART to be adherent to their medications and to maintain HIV VL suppression. This component is public health research. In this component, HIV care sites will be randomized, not individuals, and no de novo individual-level data will be collected. Instead, (de-identified) HIV surveillance data routinely collected by the DOH in the two communities will be analyzed. Because this research involves minimal risk and would be impracticable with informed consent, a waiver of patient informed consent will be requested under 45 CFR 46.116 (c) or (d).

Prevention for Positives. In this component of the study, a subset of patients enrolled at select HIV care sites in the two intervention communities will be randomized either to an intervention arm (receiving computer-delivered intervention for sexual risk reduction plus SOC prevention activities) or to the control arm (receiving only the SOC prevention activities at the care site). Individual-level data will be collected and analyzed. This component is public health research requiring informed consent. Written informed consent will be obtained from participants.

Patient survey. All patients participating in the Prevention for Positives study component will be administered the Patient Survey. Individual-level data will be analyzed. This component is public health research. Written informed consent will be obtained from

participants at the time of consenting for participation in the Prevention for Positives study component.

Provider survey. Providers from participating HIV care sites will be invited to complete a Web-based survey regarding their knowledge, attitudes and practices concerning ART for treatment, frequency of HIV testing, and limited sociodemographic information. The survey will be anonymous, and will collect no identifying information. This component is public health research. Informed consent will be obtained from the provider's completing the survey. Before the survey will display, providers will read the consent and if they agree to participate, will indicate their agreement by clicking a button labeled "I agree." Investigator's Records

Study-specific records will only be located at HIV care sites participating in the Prevention for Positives study component. At these sites, all study source documents (such as informed consent forms and patient ID linkage logs) will be maintained in a locked cabinet or room. Access to these records will be restricted to appropriate study staff members, NIAID, OHRP, government or regulatory authorities, and/or IRB.

8.6 Use of Information and Publications

Publication of the results of this study will be governed by HPTN policies. Any presentation, abstract or manuscript will be submitted to the HPTN Manuscript Review Committee for review prior to submission. Similar review may be necessary by other collaborating organizations.

8.7 Study Discontinuation

The study may be discontinued at any time by NIAID, the HPTN, the Office for Human Research Protections (OHRP), government or regulatory authorities, and/or an IRB. However, should the study be stopped early, all sites and community partners would be notified of such a decision, as well as the reasons behind it, prior to any termination activities.

9.0 REFERENCES

(CDC), C. f. D. C. a. P. (2009). "Late HIV testing - 34 states, 1996-2005." MMWR Morb Mortal Wkly Rep **58**(24): 661-5.

Aberg, J. A., J. E. Kaplan, et al. (2009). "Primary care guidelines for the management of persons infected with human immunodeficiency virus: 2009 update by the HIV medicine Association of the Infectious Diseases Society of America." Clin Infect Dis **49**(5): 651-81.

Anastos, K., M. F. Schneider, et al. (2005). "The association of race, sociodemographic, and behavioral characteristics with response to highly active antiretroviral therapy in women." J Acquir Immune Defic Syndr **39**(5): 537-44.

Applebaum, A. J., L. C. Reilly, et al. (2009). "The impact of neuropsychological functioning on adherence to HAART in HIV-infected substance abuse patients." AIDS Patient Care STDS **23**(6): 455-62.

Assefa, Y. and M. Lera (2009). "Universal voluntary HIV testing and immediate antiretroviral therapy." Lancet **373**(9669): 1080; author reply 1080-1.

Bakken, S., W. L. Holzemer, et al. (2000). "Relationships between perception of engagement with health care provider and demographic characteristics, health status, and adherence to therapeutic regimen in persons with HIV/AIDS." AIDS Patient Care STDS **14**(4): 189-97.

Bangsberg, D. R. (2008). "Preventing HIV antiretroviral resistance through better monitoring of treatment adherence." J Infect Dis **197 Suppl 3**: S272-8.

Bartlett, J. A. (2002). "Addressing the challenges of adherence." J Acquir Immune Defic Syndr **29 Suppl 1**: S2-10.

Berg, K. M., N. A. Cooperman, et al. (2009). "Self-efficacy and depression as mediators of the relationship between pain and antiretroviral adherence." AIDS Care **21**(2): 244-8.

Bigelow, G. E. and K. Silverman (1999). Theoretical and Empirical Foundations of Contingency Management Treatments for Drug Abuse. Motivating Behavior Change Among Illicit Drug Users. S. T. Higgins and K. Silverman. Washington, DC, American Psychological Association**:** 15-30.

Bradford, J. B., S. Coleman, et al. (2007). "HIV System Navigation: an emerging model to improve HIV care access." AIDS Patient Care STDS **21 Suppl 1**: S49-58.

Branson, B. M., H. H. Handsfield, et al. (2006). "Revised recommendations for HIV testing of adults, adolescents, and pregnant women in health-care settings." MMWR Recomm Rep **55**(RR-14): 1-17; quiz CE1-4.

Buchacz, K., R. K. Baker, et al. (2008). "Rates of hospitalizations and associated diagnoses in a large multisite cohort of HIV patients in the United States, 1994-2005." AIDS **22**(11): 1345-54.

Buehler, J. W., R. L. Berkelman, et al. (1992). "The completeness of AIDS surveillance." J Acquir Immune Defic Syndr **5**(3): 257-64.

Bunnell, R., J. P. Ekwaru, et al. (2006). "Changes in sexual behavior and risk of HIV transmission after antiretroviral therapy and prevention interventions in rural Uganda." AIDS **20**(1): 85-92.

Campsmith, M. L., P. Rhodes, et al. (2008). "HIV prevalence estimates--United States, 2006." MMWR Morb Mortal Wkly Rep **57**(39): 1073-6.

Castilla, J., P. Sobrino, et al. (2002). "Late diagnosis of HIV infection in the era of highly active antiretroviral therapy: consequences for AIDS incidence." AIDS **16**(14): 1945-51.

CDC. (1999). "Guidelines for Defining Public Health Research and Public Health Non-Research." Retrieved May 7, 2008, from http://www.cdc.gov/od/science/regs/hrpp/researchdefinition.htm.
CDC. (2006). "Technical Guidance for HIV/AIDS Surveillance Programs, Volume III: Security and Confidentiality Guidelines. ." Retrieved October 19, 2009, from http://www.cdc.gov/hiv/topics/surveillance/resources/guidelines/guidance/index.htm.
Cheng, D. M., H. Libman, et al. (2009). "Alcohol consumption and lipodystrophy in HIV-infected adults with alcohol problems." Alcohol **43**(1): 65-71.
Cohen, M. S., C. Gay, et al. (2007). "Narrative review: antiretroviral therapy to prevent the sexual transmission of HIV-1." Ann Intern Med **146**(8): 591-601.
Cohen, M. S., T. D. Mastro, et al. (2009). "Universal voluntary HIV testing and immediate antiretroviral therapy." Lancet **373**(9669): 1077; author reply 1080-1.
Craw, J. A., L. I. Gardner, et al. (2008). "Brief strengths-based case management promotes entry into HIV medical care: results of the antiretroviral treatment access study-II." J Acquir Immune Defic Syndr **47**(5): 597-606.
Crepaz, N., C. M. Lyles, et al. (2006). "Do prevention interventions reduce HIV risk behaviours among people living with HIV? A meta-analytic review of controlled trials." AIDS **20**(2): 143-57.
Cunningham, W. E., L. E. Markson, et al. (2000). "Prevalence and predictors of highly active antiretroviral therapy use in patients with HIV infection in the united states. HCSUS Consortium. HIV Cost and Services Utilization." J Acquir Immune Defic Syndr **25**(2): 115-23.
DHHS. (2005). "Code of Federal Regulations, Title 45 - Public Welfare, Part 46, Protection of Human Subjects. ." Retrieved May 7, 2008, from http://www.hhs.gov/ohrp/documents/OHRPRegulations.pdf.
Dieffenbach, C. W. and A. S. Fauci (2009). "Universal voluntary testing and treatment for prevention of HIV transmission." JAMA **301**(22): 2380-2.
Donatelle, R., D. Hudson, et al. (2004). "Incentives in smoking cessation: status of the field and implications for research and practice with pregnant smokers." Nicotine Tob Res **6 Suppl 2**: S163-79.
Donatelle, R. J., S. L. Prows, et al. (2000). "Randomised controlled trial using social support and financial incentives for high risk pregnant smokers: significant other supporter (SOS) program." Tob Control **9 Suppl 3**: III67-9.
Doyle, T. J., M. K. Glynn, et al. (2002). "Completeness of notifiable infectious disease reporting in the United States: an analytical literature review." Am J Epidemiol **155**(9): 866-74.
Duran, D., J. Beltrami, et al. (2008). "Persons tested for HIV--United States, 2006." MMWR Morb Mortal Wkly Rep **57**(31): 845-9.
Epstein, H. (2009). "Universal voluntary HIV testing and immediate antiretroviral therapy." Lancet **373**(9669): 1078-9; author reply 1080-1.
Fideli, U. S., S. A. Allen, et al. (2001). "Virologic and immunologic determinants of heterosexual transmission of human immunodeficiency virus type 1 in Africa." AIDS Res Hum Retroviruses **17**(10): 901-10.
Finkelstein, E. A., L. A. Linnan, et al. (2007). "A pilot study testing the effect of different levels of financial incentives on weight loss among overweight employees." J Occup Environ Med **49**(9): 981-9.
Friis-Moller, N., P. Reiss, et al. (2007). "Class of antiretroviral drugs and the risk of myocardial infarction." N Engl J Med **356**(17): 1723-35.

Gardner, L. I., L. R. Metsch, et al. (2005). "Efficacy of a brief case management intervention to link recently diagnosed HIV-infected persons to care." AIDS **19**(4): 423-31.
Gilbert, P., D. Ciccarone, et al. (2008). "Interactive "Video Doctor" counseling reduces drug and sexual risk behaviors among HIV-positive patients in diverse outpatient settings." PLoS One **3**(4): e1988.
Giordano, T. P., A. L. Gifford, et al. (2007). "Retention in care: a challenge to survival with HIV infection." Clin Infect Dis **44**(11): 1493-9.
Giordano, T. P., F. Visnegarwala, et al. (2005). "Patients referred to an urban HIV clinic frequently fail to establish care: factors predicting failure." AIDS Care **17**(6): 773-83.
Giuffrida, A. and D. J. Torgerson (1997). "Should we pay the patient? Review of financial incentives to enhance patient compliance." BMJ **315**(7110): 703-7.
Granich, R. M., C. F. Gilks, et al. (2009). "Universal voluntary HIV testing with immediate antiretroviral therapy as a strategy for elimination of HIV transmission: a mathematical model." Lancet **373**(9657): 48-57.
Greenberg, A. E., R. Hindin, et al. (1993). "The completeness of AIDS case reporting in New York City." JAMA **269**(23): 2995-3001.
Hall, H. I. and E. D. Mokotoff (2007). "Setting standards and an evaluation framework for human immunodeficiency virus/acquired immunodeficiency syndrome surveillance." J Public Health Manag Pract **13**(5): 519-23.
Hall, H. I., R. Song, et al. (2006). "Assessing the completeness of reporting of human immunodeficiency virus diagnoses in 2002-2003: capture-recapture methods." Am J Epidemiol **164**(4): 391-7.
Hall, H. I., R. Song, et al. (2008). "Estimation of HIV incidence in the United States." JAMA **300**(5): 520-9.
Hayes, R. J. and S. Bennett (1999). "Simple sample size calculation for cluster-randomized trials." Int J Epidemiol **28**(2): 319-26.
Higgins, S. T. and K. Silverman (1999). Motivating Behavior Change Among Illicit Drug Abusers: Research on Contingency Management Interventions. . American Psychological Association, Washington, DC.
Higgins, S. T., C. J. Wong, et al. (2000). "Contingent reinforcement increases cocaine abstinence during outpatient treatment and 1 year of follow-up." J Consult Clin Psychol **68**(1): 64-72.
Horberg, M. A., M. J. Silverberg, et al. (2008). "Effects of depression and selective serotonin reuptake inhibitor use on adherence to highly active antiretroviral therapy and on clinical outcomes in HIV-infected patients." J Acquir Immune Defic Syndr **47**(3): 384-90.
Hsieh, Y. H. and H. de Arazoza (2009). "Universal voluntary HIV testing and immediate antiretroviral therapy." Lancet **373**(9669): 1079-80; author reply 1080-1.
Jackevicius, C. A., M. Mamdani, et al. (2002). "Adherence with statin therapy in elderly patients with and without acute coronary syndromes." Jama **288**(4): 462-7.
Jara, M. M., K. M. Gallagher, et al. (2000). "Estimation of completeness of AIDS case reporting in Massachusetts." Epidemiology **11**(2): 209-13.
Jeffery, R. W., W. M. Gerber, et al. (1983). "Monetary contracts in weight control: effectiveness of group and individual contracts of varying size." J Consult Clin Psychol **51**(2): 242-8.
Jeffery, R. W., P. D. Thompson, et al. (1978). "Effects on weight reduction of strong monetary contracts for calorie restriction or weight loss." Behav Res Ther **16**(5): 363-9.

Johnson, B. T., M. P. Carey, et al. (2006). "Sexual risk reduction for persons living with HIV: research synthesis of randomized controlled trials, 1993 to 2004." J Acquir Immune Defic Syndr **41**(5): 642-50.

Jurgens, R., J. Cohen, et al. (2009). "Universal voluntary HIV testing and immediate antiretroviral therapy." Lancet **373**(9669): 1079; author reply 1080-1.

Justice, A. C. (2006). "Prioritizing primary care in HIV: comorbidity, toxicity, and demography." Top HIV Med **14**(5): 159-63.

Kane, R. L., P. E. Johnson, et al. (2004). "A structured review of the effect of economic incentives on consumers' preventive behavior." Am J Prev Med **27**(4): 327-52.

Kapetanovic, S., S. Christensen, et al. (2009). "Correlates of perinatal depression in HIV-infected women." AIDS Patient Care STDS **23**(2): 101-8.

Kitahata, M. M., S. J. Gange, et al. (2009). "Effect of early versus deferred antiretroviral therapy for HIV on survival." N Engl J Med **360**(18): 1815-26.

Klevens, R. M., P. L. Fleming, et al. (1998). "Completeness of HIV reporting in Louisiana, USA." Int J Epidemiol **27**(6): 1105.

Lazo, M., S. J. Gange, et al. (2007). "Patterns and predictors of changes in adherence to highly active antiretroviral therapy: longitudinal study of men and women." Clin Infect Dis **45**(10): 1377-85.

Levine, A. J., C. H. Hinkin, et al. (2005). "Variations in patterns of highly active antiretroviral therapy (HAART) adherence." AIDS Behav **9**(3): 355-62.

Lima, V. D., J. Geller, et al. (2007). "The effect of adherence on the association between depressive symptoms and mortality among HIV-infected individuals first initiating HAART." AIDS **21**(9): 1175-83.

Lussier, J. P., S. H. Heil, et al. (2006). "A meta-analysis of voucher-based reinforcement therapy for substance use disorders." Addiction **101**(2): 192-203.

Magnus, M., I. Kuo, et al. (2009). "Risk factors driving the emergence of a generalized heterosexual HIV epidemic in Washington, District of Columbia networks at risk." AIDS **23**(10): 1277-84.

Malotte, C. K., F. Rhodes, et al. (1998). "Tuberculosis screening and compliance with return for skin test reading among active drug users." Am J Public Health **88**(5): 792-6.

Marcus, A. C., C. P. Kaplan, et al. (1998). "Reducing loss-to-follow-up among women with abnormal Pap smears. Results from a randomized trial testing an intensive follow-up protocol and economic incentives." Med Care **36**(3): 397-410.

Markowitz, M., B. Y. Nguyen, et al. (2009). "Sustained Antiretroviral Effect of Raltegravir After 96 Weeks of Combination Therapy in Treatment-Naive Patients With HIV-1 Infection." J Acquir Immune Defic Syndr.

Marks, G., N. Crepaz, et al. (2006). "Estimating sexual transmission of HIV from persons aware and unaware that they are infected with the virus in the USA." AIDS **20**(10): 1447-50.

McNaghten, A. D., M. I. Wolfe, et al. (2007). "Improving the representativeness of behavioral and clinical surveillance for persons with HIV in the United States: the rationale for developing a population-based approach." PLoS One **2**(6): e550.

McQuillan, G. M., D. Kruszon-Moran, et al. (2006). "Prevalence of HIV in the US household population: the National Health and Nutrition Examination Surveys, 1988 to 2002." J Acquir Immune Defic Syndr **41**(5): 651-6.

Metsch, L. R., M. Pereyra, et al. (2004). "Delivery of HIV prevention counseling by physicians at HIV medical care settings in 4 US cities." Am J Public Health **94**(7): 1186-92.

Meyer, P. A., J. L. Jones, et al. (1994). "Completeness of reporting of diagnosed HIV-infected hospital inpatients." J Acquir Immune Defic Syndr **7**(10): 1067-73.

Mugavero, M., J. Ostermann, et al. (2006). "Barriers to antiretroviral adherence: the importance of depression, abuse, and other traumatic events." AIDS Patient Care STDS **20**(6): 418-28.

Mugavero, M. J., H. Y. Lin, et al. (2009). "Racial disparities in HIV virologic failure: do missed visits matter?" J Acquir Immune Defic Syndr **50**(1): 100-8.

Mugavero, M. J., H. Y. Lin, et al. (2007). "Failure to establish HIV care: characterizing the "no show" phenomenon." Clin Infect Dis **45**(1): 127-30.

Mugavero, M. J., H. Y. Lin, et al. (2009). "Missed visits and mortality among patients establishing initial outpatient HIV treatment." Clin Infect Dis **48**(2): 248-56.

NHBS-Het Survey (2009). NHBS-Het Survey, 2007-2008, District HIV Testing Data-the first 35,000 rapid tests.

Noar, S. M., H. G. Black, et al. (2009). "Efficacy of computer technology-based HIV prevention interventions: a meta-analysis." AIDS **23**(1): 107-15.

Norman, L. R., M. Basso, et al. (2009). "Neuropsychological consequences of HIV and substance abuse: a literature review and implications for treatment and future research." Curr Drug Abuse Rev **2**(2): 143-56.

Palella, F., C. Armon, et al. (2008). Enhanced Survival Associated with Use of HIV Susceptibility Testing among HAART-experienced Patients in the HIV Outpatient Study (HOPS) 15th Conference on Retroviruses and Opportunistic Infections.

Palella, F. J., Jr., K. M. Delaney, et al. (1998). "Declining morbidity and mortality among patients with advanced human immunodeficiency virus infection. HIV Outpatient Study Investigators." N Engl J Med **338**(13): 853-60.

Paltiel, A. D., M. C. Weinstein, et al. (2005). "Expanded screening for HIV in the United States--an analysis of cost-effectiveness." N Engl J Med **352**(6): 586-95.

Panel on Antiretroviral Guidelines for Adults and Adolescents. (2009). "Guidelines for the use of antiretroviral agents in HIV-1-infected adults and adolescents. Department of Health and Human Services. December 1, 2009." Retrieved December 21, 2009, from **http://www.aidsinfo.nih.gov/ContentFiles/AdultandAdolescentGL.pdf**.

Pence, B. W. (2009). "The impact of mental health and traumatic life experiences on antiretroviral treatment outcomes for people living with HIV/AIDS." J Antimicrob Chemother **63**(4): 636-40.

Phillips, A. N., C. Leen, et al. (2007). "Risk of extensive virological failure to the three original antiretroviral drug classes over long-term follow-up from the start of therapy in patients with HIV infection: an observational cohort study." Lancet **370**(9603): 1923-8.

Prevention, C. f. D. C. a. (2002). AIDS/HIV Surveillance Report. Atlanta, Centers for Disease Control and Prevention.

Quinn, T. C., M. J. Wawer, et al. (2000). "Viral load and heterosexual transmission of human immunodeficiency virus type 1. Rakai Project Study Group." N Engl J Med **342**(13): 921-9.

Rajabiun, S., R. K. Mallinson, et al. (2007). ""Getting me back on track": the role of outreach interventions in engaging and retaining people living with HIV/AIDS in medical care." AIDS Patient Care STDS **21 Suppl 1**: S20-9.

Reed, J. B., D. Hanson, et al. (2009). "HIV testing factors associated with delayed entry into HIV medical care among HIV-infected persons from eighteen states, United States, 2000-2004." AIDS Patient Care STDS **23**(9): 765-73.

Rigsby, M., M. Rosen, et al. (2000). "Cue-dose training with monetary reinforcement." Journal of General Internal Medicine **15**(12): 841-847.
Robison, L. S., A. O. Westfall, et al. (2008). "Short-term discontinuation of HAART regimens more common in vulnerable patient populations." AIDS Res Hum Retroviruses **24**(11): 1347-55.
Rosen, M., K. Dieckhaus, et al. (2007). "Improved adherence with contingency management." AIDS Patient Care and STDs **21**(1): 30-40.
Rosenblum, L., J. W. Buehler, et al. (1992). "The completeness of AIDS case reporting, 1988: a multisite collaborative surveillance project." Am J Public Health **82**(11): 1495-9.
Roux, P., M. P. Carrieri, et al. (2009). "Effect of anxiety symptoms on adherence to highly active antiretroviral therapy in HIV-infected women." J Clin Psychiatry **70**(9): 1328-9.
Royal, S. W., D. P. Kidder, et al. (2009). "Factors associated with adherence to highly active antiretroviral therapy in homeless or unstably housed adults living with HIV." AIDS Care **21**(4): 448-55.
Safren, S., R. Knauz, et al. (2006). "CBT for HIV medication adherence and depression: process and outcomes at post-treatment and three-month cross over." Ann Behav Med **31**: S006.
Sanders, G. D., A. M. Bayoumi, et al. (2005). "Cost-effectiveness of screening for HIV in the era of highly active antiretroviral therapy." N Engl J Med **352**(6): 570-85.
Schneider, E., S. Whitmore, et al. (2008). "Revised surveillance case definitions for HIV infection among adults, adolescents, and children aged <18 months and for HIV infection and AIDS among children aged 18 months to <13 years--United States, 2008." MMWR Recomm Rep **57**(RR-10): 1-12.
Schroeder, S. A. (2007). "Shattuck Lecture. We can do better--improving the health of the American people." N Engl J Med **357**(12): 1221-8.
Schwarcz, S. K., L. C. Hsu, et al. (1999). "The impact of the 1993 AIDS case definition on the completeness and timeliness of AIDS surveillance." AIDS **13**(9): 1109-14.
Seal, K. H., A. H. Kral, et al. (2003). "A randomized controlled trial of monetary incentives vs. outreach to enhance adherence to the hepatitis B vaccine series among injection drug users." Drug Alcohol Depend **71**(2): 127-31.
Sethi, A. K., D. D. Celentano, et al. (2003). "Association between adherence to antiretroviral therapy and human immunodeficiency virus drug resistance." Clin Infect Dis **37**(8): 1112-8.
Shapiro, M. F., S. C. Morton, et al. (1999). "Variations in the care of HIV-infected adults in the United States: results from the HIV Cost and Services Utilization Study." JAMA **281**(24): 2305-15.
Simoni, J. M., A. E. Kurth, et al. (2006). "Self-report measures of antiretroviral therapy adherence: A review with recommendations for HIV research and clinical management." AIDS Behav **10**(3): 227-45.
Simoni, J. M., C. R. Pearson, et al. (2006). "Efficacy of interventions in improving highly active antiretroviral therapy adherence and HIV-1 RNA viral load. A meta-analytic review of randomized controlled trials." J Acquir Immune Defic Syndr **43 Suppl 1**: S23-35.
Solomon, L., C. Flynn, et al. (1999). "Evaluation of a statewide non-name-based HIV surveillance system." J Acquir Immune Defic Syndr **22**(3): 272-9.
Steigbigel, R. T., D. A. Cooper, et al. (2008). "Raltegravir with optimized background therapy for resistant HIV-1 infection." N Engl J Med **359**(4): 339-54.

Sterne, J. A., M. May, et al. (2009). "Timing of initiation of antiretroviral therapy in AIDS-free HIV-1-infected patients: a collaborative analysis of 18 HIV cohort studies." Lancet **373**(9672): 1352-63.

Stevens-Simon, C., P. O'Connor, et al. (1994). "Incentives enhance postpartum compliance among adolescent prenatal patients." J Adolesc Health **15**(5): 396-9.

Sullivan, P., K. Kayitenkore, et al. (2009). Reduction of HIV Transmission Risk and High Risk Sex while Prescribed ART: Results from Discordant Couples in Rwanda and Zambia. 16th Conference on Retroviruses and Opportunistic Infections, Montreal, Canada.

Sutherland, K., J. B. Christianson, et al. (2008). "Impact of targeted financial incentives on personal health behavior: a review of the literature." Med Care Res Rev **65**(6 Suppl): 36S-78S.

Teshale, E., L. Kamimoto, et al. (2005). Estimated Number of HIV-infected Persons Eligible for and Receiving HIV Antiretroviral Therapy, 2003--United States 12th Conference on Retroviruses and Opportunistic Infections. Boston, MA.

Thomson, A., R. Hayes, et al. (2009). "Measures of between-cluster variability in cluster randomized trials with binary outcomes." Stat Med **28**(12): 1739-51.

Tobias, C. R., W. Cunningham, et al. (2007). "Living with HIV but without medical care: barriers to engagement." AIDS Patient Care STDS **21**(6): 426-34.

Torian, L. V., E. W. Wiewel, et al. (2008). "Risk factors for delayed initiation of medical care after diagnosis of human immunodeficiency virus." Arch Intern Med **168**(11): 1181-7.

Valdiserri, R. O., D. R. Holtgrave, et al. (1999). "Promoting early HIV diagnosis and entry into care." AIDS **13**(17): 2317-30.

Vlahov, D., A. M. Tang, et al. (2000). "Increased frequency of overdose mortality among HIV infected injection drug users." Additional Research **8**: 311-325.

Volpp, K. G., A. Gurmankin Levy, et al. (2006). "A randomized controlled trial of financial incentives for smoking cessation." Cancer Epidemiol Biomarkers Prev **15**(1): 12-8.

Volpp, K. G., L. K. John, et al. (2008). "Financial incentive-based approaches for weight loss: a randomized trial." JAMA **300**(22): 2631-7.

Volpp, K. G., G. Loewenstein, et al. (2008). "A test of financial incentives to improve warfarin adherence." BMC Health Serv Res **8**: 272.

Volpp, K. G., A. B. Troxel, et al. (2009). "A randomized, controlled trial of financial incentives for smoking cessation." N Engl J Med **360**(7): 699-709.

von Wyl, V., S. Yerly, et al. (2009). "Long-term trends of HIV type 1 drug resistance prevalence among antiretroviral treatment-experienced patients in Switzerland." Clin Infect Dis **48**(7): 979-87.

Wagner, B. G. and S. Blower. (2009). "Voluntary universal testing and treatment is unlikely to lead to HIV elimination: a modeling analysis." Nature Preceedings Posted 29 Oct 2009. Retrieved November 30, 2009, from http://precedings.nature.com/documents/3917/version/1.

Walensky, R. P., M. C. Weinstein, et al. (2005). "Optimal allocation of testing dollars: the example of HIV counseling, testing, and referral." Med Decis Making **25**(3): 321-9.

Washington, D. D. o. H. (2009). "Vital Stats." 2009, from www.doh.dc.gov\cppe\vital stats.

Weaver, M. R., C. J. Conover, et al. (2009). "Cost-effectiveness analysis of integrated care for people with HIV, chronic mental illness and substance abuse disorders." J Ment Health Policy Econ **12**(1): 33-46.

Weber, R., L. Christen, et al. (2004). "Effect of individual cognitive behaviour intervention on adherence to antiretroviral therapy: prospective randomized trial." Antivir Ther **9**(1): 85-95.

Weintrob, A. C., G. A. Grandits, et al. (2009). "Virologic Response Differences Between African Americans and European Americans Initiating Highly Active Antiretroviral Therapy With Equal Access to Care." J Acquir Immune Defic Syndr.

When to Start Consortium (2009). "Timing of initiation of antiretroviral therapy in AIDS-free HIV-1-infected patients: a collaborative analysis of 18 HIV cohort studies." Lancet **373**(9672): 1352-63.

Wilkin, T. J. and R. M. Gulick (2008). "When to start antiretroviral therapy?" Clin Infect Dis **47**(12): 1580-6.

Wood, E., T. Kerr, et al. (2008). "Poor adherence to HIV monitoring and treatment guidelines for HIV-infected injection drug users." HIV Med **9**(7): 503-7.

Wyatt, G. E., D. Longshore, et al. (2004). "The efficacy of an integrated risk reduction intervention for HIV-positive women with child sexual abuse histories." AIDS Behav **8**(4): 453-62.

Zetola, N. M., K. Bernstein, et al. (2009). "Using surveillance data to monitor entry into care of newly diagnosed HIV-infected persons: San Francisco, 2006-2007." BMC Public Health **9**: 17.

APPENDICES

Appendix I: Schedule of Study Visits and Procedures

Schedule of Study Visits and Procedures
Prevention for Positives Computerized Patient Intervention and Survey

	HIV Care Site<br>Visit #1<br>Enrollment		HIV Care Site<br>Visit #2- Visit #5<br>(Months 3, 6, 9 & 12)
**Administrative and Regulatory Procedures**			
Pre-screening	X		
Administer Informed Consent	X		
Confirm Eligibility	X		
Collect Locator Information	X		
Administer CARE+/ACASI	X		X
Administer Survey	X		X (Month 12 only)
Provide Compensation	X		X

Appendix IIA: Patient Computer-Delivered Intervention and Survey Informed Consent Form

COMPUTER-DELIVERED INTERVENTION AND SURVEY SUBJECT INFORMATION AND CONSENT FORM AND AUTHORIZATION TO USE AND DISCLOSE PERSONAL HEALTH INFORMATION FOR RESEARCH:

**Title of the Research Study:**	TLC-Plus: A Study to Evaluate the Feasibility of an Enhanced Test, Link-to-Care, Plus Treat Approach for HIV Prevention in the United States
**Protocol #:**	HPTN 065, Version 3.0, 14 January 2014<br>DAIDS ID: 11685
**Sponsor:**	National Institute of Allergy and Infectious Diseases (NIAID), National Institute on Drug Abuse (NIDA), National Institute of Mental Health (NIMH), National Institutes of Health (NIH)
**Investigator of Record:**	(*insert name*)
**Research Site Address(es)**	*(insert address)*
**Daytime Telephone Number:**	(*insert number*)
**24-hour Contact Number:**	*(insert number)*

Purpose of the Subject Information and Consent Form

This Subject Information and Consent Form may contain words you do not understand. Please ask the study investigator or the study staff to explain any words or procedures that you do not clearly understand.

The purpose of this form is to give you information about the research study and, if signed, will give your permission to take part in the study. The form describes the purpose, procedures, benefits, risks, discomforts and precautions of the research study. You should take part in the study only if you want to do so. You may refuse to take part or withdraw from this study at any time without penalty or loss of benefits to which you are otherwise entitled. Please read this Subject Information and Consent Form and ask as many questions as needed. You should not sign this form if you have any questions that have not been answered to your satisfaction.

Your study investigator will be paid by the sponsor to conduct this research.

Introduction

You have been asked to take part in a study that is testing a new computer program to help HIV-positive people. This research will use a computer to privately ask you questions. You will be

asked to use the computer a total of 5 times. You will use the computer every three months when you come in for your clinic visits for twelve months

Around 1320 people who are HIV-positive in Washington, D.C. and in the Bronx will participate in the study. You do not have to know how to use a computer or be able to read to be in this study.

What will happen during this study?

If you agree to take part in this study, you will first sign this Subject Information and Consent Form before any study-related procedures are performed.

We will use a computer to talk about what is going on for you with HIV. During your first and 12-month visit, we will also ask you questions about your knowledge of HIV, your knowledge of medications for treating HIV and your feelings about medical care for people with HIV.

You will be asked to answer all of the questions openly and honestly, but you may refuse to answer any of the questions or stop at any time if you feel uncomfortable. You will also be provided with contact and referral information if any of the questions raises issues that you would like to talk about further, at this or some later time.

Computer sessions are anticipated to take you approximately 15 - 40 minutes to complete.

For your time and effort, we will reimburse you $10 per visit. There is no cost to you to participate in this part of the study.

Subjects who choose to join the study will be randomly assigned to a study group. There are only two groups:

- One study group will be asked questions by the computer program.
- The second study group will be asked the same questions and will also be shown some videos. The videos will be short and will include HIV risk reduction topics. After the videos are shown, the computer then will help people create a health plan.

You will have a 50% chance of being in the group that is asked questions by the computer program. You will also have a 50% chance of being in the group that is asked questions and shown videos. The group assignment will be made randomly by the computer program. Staff at the site where you get your HIV care cannot assign you to a group and the staff will not know which group to which you are assigned.

Computer sessions for both study groups are anonymous. No names or identifying information will be attached to the computer. For both study groups, we would like permission to access medical records at the HIV clinic. We will use this information to evaluate HIV Viral Load, CD4 cell count, and other information relevant to your health, at each computer session. We would only like to access your information for as long as you are enrolled in the study. Once you have completed the study we will no longer access your medical records at any HIV clinic. We will not share information with the study clinic staff about your answers to the questions on the computer. If you decide to allow us to access your health information for the study, you will

need to sign an authorization form at the end of this consent form document giving permission to let us see your records.

For the study group that is asked questions and shown videos, an anonymous health plan will print out at the end of the computer session. If you are assigned to this group, you can decide whether to share the health plan print out with your provider. You do not have to show your provider the print out.

For both groups there are questions about depression, suicide and domestic violence. If your answers to these questions show that you may be depressed, suicidal or are currently in an abusive relationship, a health worker here at the clinic will follow-up with you to offer support and/or referral but will not know what you may be having trouble with. For example, the healthcare worker will not know whether you indicated that you are suicidal or whether you indicated that you are in an abusive relationship. We will not share your actual answers with study staff at the clinic. You can decide what information you want to share with the healthcare worker.

What are the possible risks or discomforts?

It is possible that answering the questions on the computer may make you embarrassed or upset. You may refuse to answer any of the questions or stop answering at any time. The greatest risk may involve your privacy. The steps that the study team has taken to protect your privacy are described in this form.

What are the potential benefits?

There may be no direct benefits to you. We hope the information we collect will help us find better ways to provide HIV care in your community. You may feel a benefit from sharing your experiences with someone who is interested in your opinions.

Are there any alternatives to participation?

The study coordinator will explain other programs at this site that can help HIV-positive people change their behavior so that they do not pass HIV on to others.

How will my confidentiality and privacy be protected?

We cannot guarantee absolute confidentiality. However, we will do everything possible to protect your confidentiality if you join this study.

To protect your privacy, you will meet with a healthcare provider in a private area where others cannot overhear conversations with you. While you are participating in the computerized session, you will be given headphones and a place to sit where no one can look over your shoulder to see what you are doing.

Every effort will be made to keep your personal information confidential. Your personal information (name, address, phone number) will be protected by the research clinic. This information will not be used in any publication of information about this study.

In addition to the efforts made by the study staff to help keep your personal information confidential, we have obtained a Certificate of Confidentiality from the U.S. Federal Government. This certificate protects researchers from being forced to tell people who are not connected with this study, such as the court system, about your participation. Any publication of this study will not use your name or identify you personally.

People who may review your records include: the U.S. Food and Drug Administration (FDA), ***(insert name of site IRB***), National Institutes of Health (NIH), study staff, study monitors, and drug companies supporting this study. Also, the Certificate of Confidentiality does not prevent you from releasing information about yourself and your participation in the study. The eCertificate cannot be used to resist a demand for information from personnel of the United States Government that is used for auditing or evaluation of Federally funded projects or for information that must be disclosed in order to meet the requirements of the federal Food and Drug Administration (FDA).

Even with the Certificate of Confidentiality, if the study staff learns of possible child abuse and/or neglect or a risk of harm to you or others, we will tell the proper authorities.

What happens if I am injured by participating in this study?

Because this study only involves answering questions, reading messages and viewing videos, it is very unlikely that you could be injured. However, if you are injured as a result of joining this study, you will be given immediate treatment for your injuries. You may have to pay for this care. There is no program for compensation either through this institution or the United States NIH.

What are my legal rights?

The above section does not restrict your right to seek legal assistance. You will not be giving up any of your legal rights by signing this Subject Information and Consent Form.

Your participation is voluntary.

You are not required to join this study. You do not have to participate in the computer sessions for us. If you decide to participate, you may refuse to answer any of the questions or stop at any time without reducing or affecting any care that you receive at this site. If you do decide to leave the study we will ask you to complete one final computer session, however, you will not be required to do this.

What are some reasons why I may be withdrawn from this activity without my consent?

You may be withdrawn from the study without your consent for the following reasons:

- The research study, or this part of the study, is stopped or canceled.
- The study staff feels that completing the study or this part of the study would be harmful to you or others.

Persons to Contact for Problems or Questions

If you have any questions about your participation in this research study, your rights as a research subject, or if you feel that you have experienced a research-related injury, contact:

- Investigator of Record Name: (*site insert name of the investigator or other study staff)*
- Research Site Address: (*site insert physical address of above)*
- Daytime Telephone Number(s): *(site insert telephone number)*
- 24-hour contact number(s): *(site insert telephone number)*

- If you have any questions or concerns about your rights as a research subject or want to discuss a problem, get information or offer input, you may contact: Institutional Review Board: (*site insert name or title of person on the IRB or other organization appropriate for the site)*
- Address of Institutional Review Board: *(site insert physical address of above)*
- Daytime Telephone Number(s): *(site insert telephone number of above)*

SUBJECT'S STATEMENT OF CONSENT

TLC-Plus: A Study to Evaluate the Feasibility of an Enhanced Test, Link to Care, Plus Treat Approach for HIV Prevention in the United States

- I have been given sufficient opportunity to consider whether to participate in this study.
- My taking part in this research study is voluntary. I may decide not to take part or to withdraw from the research study at any time without penalty or loss of benefits or treatments to which I am entitled.
- The research study may be stopped at any time without my consent.
- I have had an opportunity to ask my study investigator questions about this research study. My questions so far have been answered to my satisfaction.
- I have been told how long I may be in the research study.
- I have been informed of the procedures and tests that may be performed during the research study.
- I have been told what the possible risks and benefits are from taking part in this research study. I may not benefit if I take part in this research study.
- I do not give up my legal rights by signing this form.
- I have been told that prior to any study related procedures being performed, I will be asked to voluntarily sign this Study Information and Consent Form.
- I will receive a signed and dated copy of this Subject Information and Consent Form.

If you have either read or have heard the information in this Subject Information and Consent Form, if all of your questions have been answered, and if you agree to take part in the computer assisted interview and subject survey questionnaire, please sign and print your name on the line below.

I voluntarily agree to take part in this research study.

______________________	______________________
Subject's Name (print)	Subject's Signature and Date

I certify that the information provided was given in a language that was understandable to the subject.

______________________	______________________
Study Staff Conducting Consent Discussion (print)	Study Staff Signature and Date
______________________	______________________
Witness' Name (print) (As appropriate)	Witness' Signature and Date

Authorization to Use and Disclose Personal Health Information for Research

The United States government has issued a privacy rule to protect the privacy rights of patients. This rule was issued under a law called the Health Insurance Portability and Accountability Act of 1996 (HIPAA). The Privacy Rule is designed to protect the confidentiality of your personal health information. The document you are reading, called an "Authorization," describes your rights and explains how your health information will be used and disclosed (shared).

In working with the sponsor, the study investigator, ***(insert the name of site's study investigator)***, will use and share personal health information about you. This is information about your health that includes information in your medical record and information created or collected during the study. This information may include laboratory test results. Some of these tests may have been done as part of your regular care. The study investigator will use this information about you to complete this research.

The study investigator will assign a code number to your information that is shared with the sponsor. The sponsor and its representatives may review or copy your personal health information at the study site. Your IRB, ***(insert name of your IRB)***, may also review or copy your information to make sure that the study is done properly or for other purposes required by law.

By signing this Authorization, you allow the study investigator to use your personal health information to carry out and evaluate this study. You also allow the study investigator to share your personal health information with:

- the sponsor and its representatives
- ***(insert name of your site's IRB)***

Your personal health information may be further shared by the groups above. If shared by them, the information will no longer be covered by the Privacy Rule. However, these groups are committed to keeping your personal health information confidential.

You have the right to see and get a copy of your records related to the study for as long as the study investigator has this information. However, by signing this Authorization you agree that you might not be able to review or receive some of your records related to the study until after the study has been completed.

You may choose to withdraw this Authorization at any time, but you must notify the study investigator in writing. Send your written withdrawal notice to **[*insert study investigator's name & address*]**.

If you withdraw from the study and withdraw your Authorization, no new information will be collected for study purposes unless the information concerns a social harm (a bad effect) related to the study. If a social harm occurs, your entire medical record may be reviewed. All information that has already been collected for study purposes, and any new information about a social harm related to the study, will be sent to the study sponsor.

If you withdraw from the study but do not withdraw your Authorization, new personal health information may be collected until this study ends.

This Authorization does not have an expiration date. If you do not withdraw this Authorization in writing, it will remain in effect indefinitely. Your study investigator will keep this Authorization for at least 6 years.

If you do not sign this Authorization, you cannot participate in this research study. If you withdraw this Authorization in the future, you will no longer be able to participate in this study. Your decision to withdraw your Authorization or not to participate will not involve any penalty or loss of access to treatment or other benefits to which you are entitled.

AUTHORIZATION

I authorize the release of personal health information from my medical records related to this study to the sponsor and its representatives, and *(insert name of site's IRB)*, as described above. I have been told that I will receive a signed and dated copy of this Authorization for my records.

____
Printed Name of Subject

____ _______________
Signature of Subject Date

____
Printed Name of Person Obtaining Authorization

____ _______________
Signature of Person Obtaining Authorization Date

Appendix IIB: Provider Survey Online Informed Consent Text

The NIH-funded HIV Prevention Trials Network (HPTN) is conducting a study to assess the feasibility of increasing HIV testing and facilitating linkage to care for HIV-infected-positive patients with the eventual goal of reducing incident HIV infection in the U.S. For the study to successfully impact HIV testing and treatment, the study team first needs to understand current practices of front-line providers.

You are invited to participate in a brief survey. This usually takes about *_XXX_ minutes to complete. You were selected because of your experience and geographic location. The questionnaire includes questions about your knowledge of HIV, your knowledge of medications for treating HIV, and your feelings/opinions about medical care for people with HIV. No personally identifying information will be collected about you, only some basic demographics. We hope that you will feel comfortable answering all of the questions openly and honestly, but you may refuse to answer any of the questions or stop completing the questionnaire, at any time. For your time and effort in completing this survey, we are providing you with a $50 Amazon.com gift certificate. You may access the certificate by printing the web coupon attached to this survey.

If you have read the information in this consent form, and if you agree to take part in the questionnaire, please CLICK on the link below. This will constitute your informed consent to participate in this survey.

I CONSENT

Appendix IIIA: Adult HIV/AIDS Confidential Case Report

(Currently under revision, Office of Management and Budget expiration February 2010)

I. STATE/LOCAL USE ONLY

Patient's Name: ______ (Last, First, M.I.) Phone No.: ( ) ______

Address: ______ City: ______ County: ______ State: ______ Zip Code: ______

RETURN TO STATE/LOCAL HEALTH DEPARTMENT — *Patient identifier information is not transmitted to CDC!* —

U.S. DEPARTMENT OF HEALTH & HUMAN SERVICES
Centers for Disease Control and Prevention

ADULT HIV/AIDS CONFIDENTIAL CASE REPORT

(Patients ≥13 years of age at time of diagnosis)

CDC
CENTERS FOR DISEASE CONTROL AND PREVENTION

II. HEALTH DEPARTMENT USE ONLY

Form Approved OMB No. 0920-0573 Exp Date 2/28/2010

DATE FORM COMPLETED: Mo. Day Yr.

SOUNDEX CODE:

REPORT STATUS: 1 New Report 2 Update

REPORTING HEALTH DEPARTMENT: State: ______ City/County: ______

State Patient No.:

City/County Patient No.:

REPORT SOURCE:

III. DEMOGRAPHIC INFORMATION

DIAGNOSTIC STATUS AT REPORT (check one): 1 HIV Infection (not AIDS) 2 AIDS

AGE AT DIAGNOSIS: Years / Years

DATE OF BIRTH: Mo. Day Yr.

CURRENT STATUS: Alive 1 Dead 2 Unk. 9

DATE OF DEATH: Mo. Day Yr.

STATE/TERRITORY OF DEATH: ______

SEX: 1 Male 2 Female

ETHNICITY: (select one) 1 Hispanic 9 Unk 2 Not Hispanic or Latino

RACE: (select one or more) American Indian/Alaska Native; Black or African American; Asian; Native Hawaiian or Other Pacific Islander; White; Unk

COUNTRY OF BIRTH: 1 U.S. 7 U.S. Dependencies and Possessions (including Puerto Rico) (specify): ______ 8 Other (specify): ______ 9 Unk

RESIDENCE AT DIAGNOSIS: City: ______ County: ______ State/Country: ______ Zip Code:

IV. FACILITY OF DIAGNOSIS

Facility Name ______

City ______

State/Country ______

FACILITY SETTING (check one): 1 Public 2 Private 3 Federal 9 Unk.

FACILITY TYPE (check one): 01 Physician, HMO 31 Hospital, Inpatient 88 Other (specify): ______

This report to the Centers for Disease Control and Prevention (CDC) is authorized by law (Sections 304 and 306 of the Public Health Service Act, 42 USC 242b and 242k). Response in this case is voluntary for federal government purposes, but may be mandatory under state and local statutes. Your cooperation is necessary for the understanding and control of HIV/AIDS. Information in CDC's HIV/AIDS surveillance system that would permit identification of any individual on whom a record is maintained, is collected with a guarantee that it will be held in confidence, will be used only for the purposes stated in the assurance on file at the local health department, and will not otherwise be disclosed or released without the consent of the individual in accordance with Section 308(d) of the Public Health Service Act (42 USC 242m).

V. PATIENT HISTORY

AFTER 1977 AND PRECEDING THE FIRST POSITIVE HIV ANTIBODY TEST OR AIDS DIAGNOSIS, THIS PATIENT HAD (Respond to **ALL** Categories):

	Yes	No	Unk.
• Sex with male	1	0	9
• Sex with female	1	0	9
• Injected nonprescription drugs	1	0	9
• Received clotting factor for hemophilia/coagulation disorder	1	0	9
Specify disorder: 1 Factor VIII (Hemophilia A) 2 Factor IX (Hemophilia B) 8 Other (specify): ______			
• *HETEROSEXUAL* relations with any of the following:			
• Intravenous/injection drug user	1	0	9
• Bisexual male	1	0	9
• Person with hemophilia/coagulation disorder	1	0	9
• Transfusion recipient with documented HIV infection	1	0	9
• Transplant recipient with documented HIV infection	1	0	9
• Person with AIDS or documented HIV infection, risk not specified	1	0	9
• Received transfusion of blood/blood components (other than clotting factor)	1	0	9
First Mo. Yr. Last Mo. Yr.			
• Received transplant of tissue/organs or artificial insemination	1	0	9
• Worked in a health-care or clinical laboratory setting	1	0	9
(specify occupation): ______			

VI. LABORATORY DATA

1. HIV ANTIBODY TESTS AT DIAGNOSIS: (Indicate first test)

	Pos	Neg	Ind	Not Done	TEST DATE Mo.	Yr.
• HIV–1 EIA	1	0	–	9		
• HIV–1/HIV–2 combination EIA	1	0	–	9		
• HIV–1 Western blot/IFA	1	0	8	9		
• Other HIV antibody test (specify): ______	1	0	8	9		

• Date of last documented negative HIV test (specify type): ______ Mo. Yr.

• If HIV laboratory tests were not documented, is HIV diagnosis documented by a physician? Yes 1 No 0 Unk. 9

If yes, provide date of documentation by physician Mo. Yr.

2. POSITIVE HIV DETECTION TEST: (Record earliest test) Mo. Yr.

culture; antigen; PCR, DNA or RNA probe

• Other (specify): ______

3. DETECTABLE VIRAL LOAD TEST: (Record most recent test)

Test type* COPIES/ML Mo. Yr.

*Type: 11. NASBA (Organon) 12. RT-PCR (Roche) 13. bDNA(Chiron) 18. Other

4. IMMUNOLOGIC LAB TESTS:

AT OR CLOSEST TO CURRENT DIAGNOSTIC STATUS Mo. Yr.

• CD4 Count ___,___ cells/µL

• CD4 Percent ___ %

First <200 µL or <14% Mo. Yr.

• CD4 Count ___,___ cells/µL

• CD4 Percent ___ %

CDC 50.42A REV. 03/2007 (Page 1 of 2) – ADULT HIV/AIDS CONFIDENTIAL CASE REPORT –

VII. STATE/LOCAL USE ONLY

Physician's Name: ______________________ Phone No.: ( ) ____________ Medical Record No. ____________
(Last, First, M.I.)
Hospital/Facility: ______________________ Person Completing Form: ______________ Phone No.: ( ) ____________

– Patient identifier information is not transmitted to CDC! –

VIII. CLINICAL STATUS

CLINICAL RECORD REVIEWED: Yes [1] No [0]

ENTER DATE PATIENT WAS DIAGNOSED AS: Asymptomatic (including acute retroviral syndrome and persistent generalized lymphadenopathy): Mo. ☐☐ Yr. ☐☐ Symptomatic (not AIDS): Mo. ☐☐ Yr. ☐☐

AIDS INDICATOR DISEASES	Initial Diagnosis Def.	Initial Diagnosis Pres.	Initial Date Mo.	Initial Date Yr.	AIDS INDICATOR DISEASES	Initial Diagnosis Def.	Initial Diagnosis Pres.	Initial Date Mo.	Initial Date Yr.
Candidiasis, bronchi, trachea, or lungs	1	NA			Lymphoma, Burkitt's (or equivalent term)	1	NA		
Candidiasis, esophageal	1	2			Lymphoma, immunoblastic (or equivalent term)	1	NA		
Carcinoma, invasive cervical	1	NA			Lymphoma, primary in brain	1	NA		
Coccidioidomycosis, disseminated or extrapulmonary	1	NA			*Mycobacterium avium* complex or *M.kansasii*, disseminated or extrapulmonary	1	2		
Cryptococcosis, extrapulmonary	1	NA			*M. tuberculosis*, pulmonary*	1	2		
Cryptosporidiosis, chronic intestinal (>1 mo. duration)	1	NA			*M. tuberculosis*, disseminated or extrapulmonary*	1	2		
Cytomegalovirus disease (other than in liver, spleen, or nodes)	1	NA			*Mycobacterium*, of other species or unidentified species, disseminated or extrapulmonary	1	2		
Cytomegalovirus retinitis (with loss of vision)	1	2			*Pneumocystis carinii* pneumonia	1	2		
HIV encephalopathy	1	NA			Pneumonia, recurrent, in 12 mo. period	1	2		
Herpes simplex: chronic ulcer(s) (>1 mo. duration); or bronchitis, pneumonitis or esophagitis	1	NA			Progressive multifocal leukoencephalopathy	1	NA		
Histoplasmosis, disseminated or extrapulmonary	1	NA			Salmonella septicemia, recurrent	1	NA		
Isosporiasis, chronic intestinal (>1 mo. duration)	1	NA			Toxoplasmosis of brain	1	2		
Kaposi's sarcoma	1	2			Wasting syndrome due to HIV	1	NA		

Def. = definitive diagnosis Pres. = presumptive diagnosis

* RVCT CASE NO.: ☐☐☐☐☐☐☐☐☐

- **If HIV tests were not positive or were not done, does this patient have an immunodeficiency that would disqualify him/her from the AIDS case definition?** [1] Yes [0] No [9] Unknown

IX. TREATMENT/SERVICES REFERRALS

Has this patient been informed of his/her HIV infection? [1] Yes [0] No [9] Unk.

This patient's partners will be notified about their HIV exposure and counseled by:
[1] Health department [2] Physician/provider [3] Patient [9] Unknown

This patient is receiving or has been referred for:

	Yes	No	NA	Unk.
• HIV related medical services	1	0	–	9
• Substance abuse treatment services	1	0	8	9

This patient received or is receiving:

	Yes	No	Unk.
• Anti-retroviral therapy	1	0	9
• PCP prophylaxis	1	0	9

This patient has been enrolled at:

Clinical Trial	Clinic
[1] NIH-sponsored	[1] HRSA-sponsored
[2] Other	[2] Other
[3] None	[3] None
[9] Unknown	[9] Unknown

This patient's medical treatment is <u>primarily</u> reimbursed by:
[1] Medicaid [2] Private insurance/HMO
[3] No coverage [4] Other Public Funding
[7] Clinical trial/ government program [9] Unknown

FOR WOMEN:
- This patient is receiving or has been referred for gynecological or obstetrical services: .... [1] Yes [0] No [9] Unknown
- Is this patient currently pregnant? .... [1] Yes [0] No [9] Unknown
- Has this patient delivered live-born infants? .... [1] Yes (if delivered after 1977, provide birth information below for the most recent birth) [0] No [9] Unknown

CHILD'S DATE OF BIRTH: Mo. ☐☐ Day ☐☐ Yr. ☐☐

Hospital of Birth: ______________________
City: ______________ State: ______________

Child's Soundex: ☐☐☐☐ ______________

Child's State Patient No. ☐☐☐☐☐☐☐☐☐☐

X. COMMENTS: ____

Public reporting burden of this collection of information is estimated to average 20 minutes per response, including the time for reviewing instructions, searching existing data sources, gathering and maintaining the data needed, and completing and reviewing the collection of information. An agency may not conduct or sponsor, and a person is not required to respond to a collection of information unless it displays a currently valid OMB control number. Send comments regarding this burden estimate or any other aspect of this collection of information, including suggestions for reducing this burden to CDC, Project Clearance Officer, 1600 Clifton Road, MS D-74, Atlanta, GA 30333, ATTN: PRA (0920-0573). **Do not send the completed form to this address.**

CDC 50.42A REV. 03/2007 (Page 2 of 2) – ADULT HIV/AIDS CONFIDENTIAL CASE REPORT –

Appendix IIIB: Behavioral Risk Factor Surveillance System (BRFSS)

(Section 20: HIV/AIDS)

The next few questions are about the national health problem of HIV, the virus that causes AIDS. Please remember that your answers are strictly confidential and that you don't have to answer every question if you do not want to. Although we will ask you about testing, we will not ask you about the results of any test you may have had.

20.1 Have you ever been tested for HIV? Do not count tests you may have had as part of a blood donation. Include testing fluid from your mouth.

1	Yes		7	Don't know / Not sure	**[Go to Q20.5]**
2	No	**[Go to Q20.5]**	9	Refused	**[Go to Q20.5]**

20.2 Not including blood donations, in what month and year was your last HIV test?

NOTE: If response is before January 1985, code "Don't know."

CATI INSTRUCTION: If the respondent remembers the year but cannot remember the month, code the first two digits 77 and the last four digits for the year.

_ _ /_ _ _ _	Code month and year
7 7/ 7 7 7 7	Don't know / Not sure
9 9/ 9 9 9 9	Refused

20.3 Where did you have your last HIV test — at a private doctor or HMO office, at a counseling and testing site, at a hospital, at a clinic, in a jail or prison, at a drug treatment facility, at home, or somewhere else?

0 1	Private doctor or HMO office
0 2	Counseling and testing site
0 3	Hospital
0 4	Clinic
0 5	Jail or prison (or other correctional facility)
0 6	Drug treatment facility
0 7	At home
0 8	Somewhere else
7 7	Don't know / Not sure
9 9	Refused

CATI note: Ask Q20.4; if Q20.2 = within last 12 months. Otherwise, go to Q20.5.

20.4 Was it a rapid test where you could get your results within a couple of hours?

1	Yes	7	Don't know / Not sure
2	No	9	Refused

20.5 I'm going to read you a list. When I'm done, please tell me if any of the situations apply to you. You do not need to tell me which one.

- You have used intravenous drugs in the past year.
- You have been treated for a sexually transmitted or venereal disease in the past year.
- You have given or received money or drugs in exchange for sex in the past year.
- You had anal sex without a condom in the past year.

Do any of these situations apply to you?

1	Yes	7	Don't know / Not sure
2	No	9	Refused

Appendix IIIC: 2008 PEMS Variable Requirements

PEMS Required Variables
Quick Reference Guide
February 8, 2008

This document provides a summary of the variable requirements for the January 1 and July 1, 2008 data collection periods, excluding variable requirements for HIV Testing and Partner Counseling and Referral Services (PCRS). HIV Testing variable requirements are currently specified in the HIV Testing Form and Variables Manual and the CDC HIV Testing Variables Data Dictionary. Requirements for PCRS will be released later in 2008. Since this document only provides a summary of the requirements, please refer to the PEMS DVS for a more detailed description of definitions and value choices.

Variable Number	Variable Name	HD & CBO Reported Required
	**General Agency Information (Table A)**	
A01	Agency Name	Required
A01a	PEMS Agency ID	Required
A02	Community Plan Jurisdiction	Required
A03	Employer Identification Number (EIN)	Required
A04	Street Address 1	Required
A06	City	Required
A08	State	Required
A09	Zip Code	Required
A10	Agency Website	Required
A11	Agency DUNS Number	Required
A12	Agency Type	Required
A13	Faith-based	Required
A14	Race/Ethnicity Minority Focused	Required
A18	Directly Funded Agency	Required
A21	Agency Contact Last Name	Required
A22	Agency Contact First Name	Required
A23	Agency Contact Title	Required
A24	Agency Contact Phone	Required
A25	Agency Contact Fax	Required
A26	Agency Contact Email	Required
	**CDC Program Announcement Award Information (Table B)**	
B01	CDC HIV Prevention PA Number	Required
B02	CDC HIV Prevention PA Budget Start Date	Required
B03	CDC HIV Prevention PA Budget End Date	Required
B04	CDC HIV Prevention PA Award Number	Required
B06	Total CDC HIV Prevention Award Amount	Required
B06a	Annual CDC HIV Prevention Award Amount Expended	Required
B07	Amount Allocated for Community Planning	Required
B08	Amount Allocated for Prevention Services	Required
B09	Amount Allocated for Evaluation	Required
B10	Amount Allocated for Capacity Building	Required

PEMS Required Variables
Quick Reference Guide
February 8, 2008

Variable Number	Variable Name	HD & CBO Reported Required
	**Contractor Information (Table C)**	
C01	Agency Name	Required
C04	City	Required
C06	State	Required
C07	Zip Code	Required
C13	Employer Identification Number (EIN)	Required
C14	DUNS Number	Required
C15	Agency Type	Required
C16	Agency Activities	Required
C17	Faith-based	Required
C18	Race/Ethnicity Minority Focused	Required
C19	Contract Start Date-Month	Required
C20	Contract Start Date-Year	Required
C21	Contract End Date- Month	Required
C22	Contract End Date- Year	Required
C23	Total Contract Amount Awarded	Required
C25	CDC HIV Prevention Program Announcement Number	Required
C26	CDC HIV Prevention PA Budget Start Date	Required
C27	CDC HIV Prevention PA Budget End Date	Required
	**Site Information (Table S)**	
S01	Site ID	Required
S03	Site Name	Required
S04	Site Type	Required
S08	County	Required
S09	State	Required
S10	Zip Code	Required
S16	Use of Mobile Unit	Required
	**Program Name - Planning (Table D)**	
D01	Program Name	Required
D02	Community Planning Jurisdiction	Required
D03	Community Planning Year	Required
	**Program Model and Budget - Planning (Table E1)**	
E101	Program Model Name	Required
E102	Evidence Base	Required
E103	CDC Recommended Guidelines	Required
E104	Other Basis for Program Model	Required
E104-1	Specify Other Basis for Program Model	Required
E105	Target Population	Required
E107	Program Model Start Date	Required
E108	Program Model End Date	Required

PEMS Required Variables
Quick Reference Guide
February 8, 2008

Variable Number	Variable Name	HD & CBO Reported Required
E109	Proposed Annual Budget	Required
	**Intervention Plan Characteristics (Table F)**	
F01	Intervention Type	Required
F02	Intervention ID	Required
F02a	Intervention Name	Required
F03	HIV+ Intervention	Required
F04	Perinatal Intervention	Required
F05	Total Number of Clients	Required
F06	Sub-Total Target Population	Required
F07	Planned Number of Cycles	Required
F08	Number of Sessions	Required
F09	Unit of Delivery	Required
F11	Delivery Method	Required
F14	Level of Data Collection	Required
	**Client Characteristics (Table G)**	
G101	Date Collected	Required
G102	PEMS Client Unique Key	Required
G112	Date of Birth - Year	Required
G113	Calculated Age (System Generated)	Required
G114	Ethnicity	Required
G116	Race	Required
G120	State/Territory of Residence	Required
G123	Assigned Sex at Birth	Required
G124	Current Gender	Required
G200	Date Collected	Required
G204	Previous HIV Test	Required
G205	Self Reported HIV Test Result	Required
G208	In HIV Medical Care/Treatment (only if HIV+)	Required
G209	Pregnant (only if female)	Required
G210	In Prenatal Care (only if pregnant)	Required
G211	Client Risk Factors ***	Required
G212	Additional Client Risk Factors ^^^	Required
G213	Recent STD (Not HIV)	Required

***Note: The recall period for client risk factors is 12 months.
^^^Note: Additional value choices for risk factors added:
Sex without using a condom
Sharing drug injection equipment

PEMS Required Variables
Quick Reference Guide
February 8, 2008

Variable Number	Variable Name	HD & CBO Reported Required
**Client Intervention Charactertics (Table H)**		
H01	Intervention ID	Required
H01a	Intervention Name	Required
H03	Cyce	Required
H04a	Form ID (Counseling & Testing Only)	Required
H05	Session Number	Required
H06	Session Date	Required
H10	Site Name/ID	Required
H13	Recruitment Source	Required
H18	Recruitment Source - Service/Intervention Type	Required
H21	Incentive Provided	Required
H22	Unit of Delivery	Required
H23	Delivery Method	Required
**Referral (Table X7)**		
X702	Referral Date	Required
X702a	Reason Client Not Refrerred to Medical Care	Required
X703	Referral Service Type	Required
X706	Referral Outcome	Required
X710	Referral Close Date	Required
X712	HIV Test Performed	Required
X713	HIV Test Result	Required
X714	Confirmatory Test	Required
X714a	HIV Test Result Provided	Required

PEMS Required Variables
Quick Reference Guide
February 8, 2008

Variable Number	Variable Name	HD & CBO Reported Required
	**Aggregate HE/RR and Outreach (Table AG)**	
AG00	Intervention Name/ID	Required
AG01	Session Number	Required
AG02	Date of Event/Session	Required
AG03	Duration of Event/Session	Required
AG04	Number of Client Contacts	Required
AG05a	Delivery Method	Required
AG05c	Incentive Provided	Required
AG06	Site Name/ID	Required
AG08a	Client Primary Risk - MSM	Required
AG08b	Client Primary Risk - IDU	Required
AG08c	Client Primary Risk - MSM/IDU	Required
AG08d	Client Primary Risk - Sex Involving Transgender	Required
AG08e	Client Primary Risk - Heterosexual Contact	Required
AG08f	Client Primary Risk - Other/Risk Not Identified	Required
AG09a	Client Gender - Male	Required
AG09b	Client Gender - Female	Required
AG09c	Client Gender - Transgender MTF	Required
AG09d	Client Gender - Transgender FTM	Required
AG10a	Client Ethnicity - Hispanic or Latino	Required
AG10b	Client Ethnicity - Not Hispanic or Latino	Required
AG11a	Client Race - American Indian or Alaska Native	Required
AG11b	Client Race - Asian	Required
AG11c	Client Race - Black or African American	Required
AG11d	Client Race - Native Hawaiian or Other Pacific Islander	Required
AG11e	Client Race - White	Required
AG11f	Client Race - Multiracial	Required
AG12a	Client Age - Under 13 years	Required
AG12b	Client Age - 13 - 18 years	Required
AG12c	Client Age - 19-24 years	Required
AG12d	Client Age - 25 - 34 years	Required
AG12e	Client Age - 35 - 44 years	Required
AG12f	Client Age - 45 years and over	Required
AG14a	Materials Distributed - Male Condoms	Required
AG14b	Materials Distributed - Female Condoms	Required
AG14c	Materials Distributed - Bleach or Safer Injection Kits	Required
AG14d	Materials Distributed - Education Materials	Required
AG14e	Materials Distributed - Safe Sex Kits	Required
AG14f	Materials Distributed - Referral list	Required
AG14g	Materials Distributed - Role Model Stories	Required
AG14h	Materials Distributed - Other (specify)	Required
AG15	Aggregate Data Collection Method	Required

PEMS Required Variables
Quick Reference Guide
February 8, 2008

Variable Number	Variable Name	HD & CBO Reported Required
**Health Communication / Public Information (Table HC)**		
HC01	Intervention Name/ID	Required
HC02	HC/PI Delivery Method	Required
HC05	Event Start Date	Required
HC06	Event End Date	Required
HC07	Total Number of Airings	Required
HC08	Estimated total Exposures	Required
HC09	Number of Materials Distributed	Required
HC10	Total Number of Web Hits	Required
HC11	Total Number of Attendees	Required
HC12	Number of Callers	Required
HC13	Number of Callers Referred	Required
HC14	Distribution - Male condoms	Required
HC15	Distribution - Female condoms	Required
HC16	Distribution - Lubricants	Required
HC17	Distribution - Bleach or Safer Injection Kits	Required
HC18	Distribution - Referral Lists	Required
HC19	Distribution - Safe sex kits	Required
HC20	Distribution - Other	Required
HC21	Site Name/ID	Required
**Community Planning Level (Table CP-A/B/C)**		
CP-A01	Name of HIV Prevention CPG	HD only
CP-A02	Community Plan Year	HD only
CP-B01	Priority Population	HD only
CP-B02	Rank	HD only
CP-B03	Age	HD only
CP-B04	Gender	HD only
CP-B05	Ethnicity	HD only
CP-B06	Race	HD only
CP-B07	HIV Status	HD only
CP-B08	Geo Location	HD only
CP-B09	Transmission Risk	HD only
CP-C01	Name of the Prevention Activity/Intervention	HD only
CP-C02	Prevention Activity/Intervention Type	HD only
CP-C04	Evidence Based	HD only
CP-C05	CDC Recommended Guidelines	HD only
CP-C06	Other Basis for Intervention	HD only
CP-C07	Activity	HD only

Version 2.0
Feb 2008

Required Variables for Data Collection 2008
Page 6

Appendix IIID: NYC HIV Surveillance Performance Indicators

New York State requires named reporting of all diagnoses of HIV and AIDS, all HIV-related illness, all positive Western Blot (WB) tests for HIV antibody, all VL and CD4 lymphocyte values, and all HIV genotypes. The NYC HIV/AIDS Reporting System (HARS) is a population-based registry that since 1981 has been continuously updated with new, de-duplicated diagnoses and laboratory results. All incoming provider and laboratory reports that do not match an existing registry record initiate a field investigation with medical record review to confirm the case, date and disposition of diagnosis and collect all other data required for surveillance and partner notification. HARS also obtains data through regular matches with other disease registries, the NYC Death Registry, the National Death Index and the Social Security Death Master File. Because of its comprehensive nature, long history (AIDS reporting since 1981, HIV reporting since 2000) and location (AIDS epicenter, largest in the west), the system can reasonably be characterized as the largest longitudinal community HIV/AIDS database in the world. It is therefore an ideal source of population-level outcome data for communities mounting interventions to improve early diagnosis and uptake of care.

Because of the size, history, continuous feed of new information (>35,000 new laboratory results received per month), and reliance on outside entities (laboratories, medical records) of the surveillance system, control of its quality, completeness, accuracy and timeliness is an ongoing challenge. The program follows a set of monthly performance indicators to track data quality and staff and provider performance, and meets on a quarterly basis to review indicators, identify problem areas and take steps toward corrective action. The following points summarize the volume and periodicity of electronic laboratory reporting, the volume, timeliness and outcome of field investigations, the periodic registry matches, internal matching and deduplication procedures, and the interstate deduplication activities of the program.

NYC HIV Core Surveillance Monthly Performance Indicators July 1, 2008-June 30, 2009

Electronic laboratory reporting from laboratory to NYS and from NYS to NYC in calendar year 2008:

- Western Blot file received weekly (total N = 15,308)
- VL file received every two weeks (total N = 337,418)
- Low CD4 (<200) received every four weeks (total N = 95,479)
- High CD4 (200-499) received every 4-5 weeks (total N = 192,494)
- Very High CD4 (500+) received periodically (total N = 134,019)
- Total laboratory reports excluding genotype received 2008= 640,699
- Nucleotide sequences received monthly (total cumulative N 2008-2009= 71,074)

Total number of laboratory reports received and processed July 1, 2008-June 30, 2009: 453,791

- Mean 37,816 laboratory reports were received per month
- Mean 7,003 unique individuals were represented by these reports
- Mean 1,045 reports were potential new cases and initiated field investigations
- Mean 18,999 reports matched to previous cases

- Range 158-240 reports could not be assigned because of missing name, DOB, provider, or other critical matching data (specific labs flagged by city and state for corrective action)

NYS electronic laboratory reporting lag indicators 2008-9:

- Western Blot
    - Time from draw date to submission by laboratory (varies by lab, negotiated by state, range = 1-30 days, mean = 14 days)
    - Mean time from state to city 7 days
    - Total time to city mean = 21 days
- VL
    - Time from draw date to submission (varies by lab, negotiated by state, range = 1-30 days, mean = 7 days
    - Mean time from state to city = 21 days
    - Mean time from draw date to city = 30 days
- CD4
    - Varies by test result, transmissions every 3 weeks
    - Mean time from draw date to submission to state = 7 days
    - Mean time from state to city = 30 days
- Genotype
    - Mean time from draw date to submission by laboratory 90 days
    - Mean time from state to city = 30 days

Field investigations:

- 12,539 field investigations initiated on potential new cases (non-matches to registry July 1, 2008-June 30, 2009
- 4,472 (35.7%) dispositioned as new diagnoses
    - 20.9% previously reported
    - 40.1% non-cases
    - 2% patient not at site/chart not found
    - 1.5% out of jurisdiction
- Process:
    - 93% of new case investigations returned to surveillance within 2 months of initiation
    - 50% of new diagnoses had provider report form (PRF) submitted as required by NYS Public Health Law Article 21

Vital Status Ascertainment

- Quarterly matches of HIV Registry with NYC vital registry file on HIV-related cause of death
- Semiannual match of HIV Registry with NYC death registry for all causes of death
- Annual match of HIV Registry against Social Security Death Master File
- Annual or biannual match of HIV presumed living cases in HIV Registry against National Death Index

Internal Duplicates and Merges

- Program algorithm ("DupMerge") identified potential duplicate pairs:
    - Same last name, first name, DOB (16 pairs 2008-2009, 130 cumulative)
    - Same SSN (1 pair 2008)
    - Same death certificate and date of death (1 pair 2008, 400 cumulative)
    - Same NYSID number (500 cumulative pairs)
    - Other categories (300 cumulative pairs)
- Corrections
    - Identified, entered, and investigation initiated N=379
    - Investigation completed, fix in progress N=70
    - Investigation completed, fix accomplished N=253
    - Duplicates eliminated: 116 cases deleted
- New matching product in test mode now – IBM Quality Stage(
    - Uses reference dataset to standardize key variables, e.g., first name, last name, date of birth, Social Security number, street address, city and zip
    - Divides matching variables into blocks, sequentially matches and rates blocks in multiple data passes, and creates match weight
    - Test file: 4.6 million records in pre-eHARS laboratory database
        - 672,257 duplicate tests identified and removed
        - 3.7/3.9 million laboratory records rated "high scoring" matches by QS
        - Generated 3,500 cases for manual review
            - 780 duplicate pairs, many of which had already been identified by standard program algorithm and "DupMerge" facility
            - 2,700 cases with mismatched lab documents (represents 30,588 laboratory reports)

Interstate Duplicates – Routine Interstate Duplicate Review

- Backlog of 17,000 cases dating from 2005 was cleared in 2009, entered into Duplicate Review tab in eHARS and submitted to CDC

- Review completed of 2,795 (89%) potential duplicate pairs out of 3,148 potential interstate duplicates assigned for resolution by CDC in 2008.

Completeness and Timeliness of Reporting

- NYC used least squares regression to estimate number of new diagnoses expected in 2007
- Of 3,862 cases expected to be reported in 2007, 3,829 (99.2%) had been reported to the NYCDOHMH by December 31, 2008 (within one calendar year of the date of diagnosis

Appendix IIIE: Washington, D.C. Core HIV Surveillance System

The District has had AIDS reporting since 1985 and transitioned to names based HIV reporting in November, 2006. Since the transition to names based HIV reporting, the District has worked to develop policies and procedures to control the quality, completeness, accuracy and timeliness of HIV reporting data. The District of Columbia requires named reporting of all diagnoses of HIV and AIDS, all HIV-related illness, all positive Western Blot (WB) tests for HIV antibody, all VL and CD4 lymphocyte values, and all HIV genotypes. The District's enhanced HIV/AIDS Reporting System (eHARS) is a population-based registry that since 1981 has been continuously updated with new, de-duplicated diagnoses and laboratory results. All incoming provider and laboratory reports that do not match an existing registry record initiate a field investigation with medical record review to confirm the case, date and disposition of diagnosis and collect all other data required for surveillance and partner notification. The Districts eHARS also obtains data through routine matches with other disease registries; Sexually Transmitted Disease Surveillance system (STD*MIS), the Districts electronic Death Registry (eDeath Registry), electronic Birth Registry (eBirth) and HIV client level data from HRSA and AIDS Drug Assistance Program(ADAP) as well as the Social Security Death Master File. The surveillance program follows a set of monthly performance indicators to track data quality and staff and provider performance, and meets on a monthly basis to review indicators, identify problem areas and take appropriate action steps.

Laboratory Reporting Summary

According to the District's Laboratory Licensing Administration, there are 27 laboratories licensed to do HIV testing in the District. Annually these laboratories are surveyed to assess their testing volume and the types of testing they perform. In the District, positive western blots and all CD4 and VL values are reportable. In 2008, only 2 smaller testing laboratories were found not to be in compliance with HIV laboratory reporting requirements, accounting for less than 1% of testing in the District. Completeness of reporting has improved significantly since the Districts transition to names based HIV reporting in 2006 and has continued to improve annually as more labs in the District begin electronic laboratory reporting (ELR). To improve on timeliness of laboratory reporting, in August, 2009, laboratories were notified of the legal requirements for reporting and received detailed instructions on tests they are required to report including data from 2007-present. Below is a summary of the laboratory reports received during 2008 and 2009.

The following points summarize the volume and periodicity of electronic laboratory reporting, the volume, timeliness and outcome of field investigations, the periodic registry matches, internal matching and deduplication procedures, and the interstate deduplication activities of the program.

Electronic laboratory reporting from laboratory to DC-DOH
Estimated total number of laboratory reports received and processed in the District of Columbia, 2008-2009

Number of Lab Reports Per Year

Lab Test	2008	2009	Total
WBLOT	2,175	1,138	3,028
CD4CNT	5,029	5,532	9,178
CD4PCT	4,870	5,378	8,903
VL	11,634	12,428	20,955

- Western Blot file received monthly (total N = 126)
- VL file received monthly (total N = 873)
- Number and Proportion of CD4, Low CD4 (<200), monthly (total N = 191 (37.1%))
- Number and Proportion of CD4, High CD4 (200-499) monthly (total N = 166 (32.2%))
- Number and Proportion of CD4, Very High CD4 (500+) monthly (total N = 158 (30.7%)
- Number and Proportion of VL, Undetectable (<400 C/ML), monthly (total N = 491 (46.3%))
- Number and Proportion of VL, Detectable (400-10,000 C/ML) monthly (total N = 264 (24.9%))
- Number and Proportion of VL, Very High (>10,000 C/ML) monthly (total N = 305 (28.8%))
- Mean 2,005 laboratory reports were received per month
- Mean 176 reports were potential new cases and initiated field investigations. These reports on average were for 53 unique people
- Mean 1,174 reports matched to previous cases or to 387 unique people

De-duplication of eHARS

All "newly reported" HIV cases must undergo thorough de-duplication within the District and with other jurisdictions. Monthly de-duplication of HIV/AIDS is done prior to sending records to CDC and completed using version SAS 9.0 and Linkplus. The key data elements for matching include the following:

Name
Date of Birth
Gender
Social Security Number

Intrastate/Interstate De-Duplication

If a duplicate exists, the record with the earliest date of diagnosis is retained and any additional information is added to this case record. In addition to reconciling Routine Interstate De-Duplication lists generated by CDC, any cases under investigation that are reported with an indication of being an out of state resident are referred to the appropriate jurisdiction.

Review of other Documents to ensure completeness of reporting:

The District employs the following routine database matches to assess the completeness, timeliness and quality of HIV reporting data.

Vital Status Ascertainment

- Death certificates are received monthly from Vital Records. Quarterly matches with DC vital statistics database
- Annual match of HIV Registry against Social Security Death Master File

.

AIDS Drug Assistance Program (ADAP)

- Quarterly matches conducted to update existing case data and to identify new cases to eHARS surveillance database

Medicaid

- Claims for persons with ICD9/10 codes indicative of HIV/AIDS are reviewed quarterly to update existing case data and to identify new cases

<u>Timeliness</u>

Approximately 85% of District laboratory reports are received within 2 weeks of the test date via electronic reporting. The remaining 15% are reported via the US mail, averaging about a 3 week lag in reporting.

SUMMARY OF CHANGES
INCLUDED IN THE FULL PROTOCOL AMENDMENT OF:

HPTN 065
TLC-Plus: A Study to Evaluate the Feasibility of an Enhanced Test, Link to Care, Plus Treat Approach for HIV Prevention in the United States
Version 1.0 / 01 March 2010

THE AMENDED PROTOCOL IS IDENTIFIED AS:
Version 2.0 / 15 July 2010

Prior to implementation of this amended protocol, HPTN 065 HIV test and care sites will submit this Summary of Changes document, Version 2.0 of the protocol, and the revised site-specific study informed consent forms to either their own Institutional Review Board (IRB) or the central IRB.

The study will begin under Version 2.0 upon receipt of IRB approval and activation by the HPTN CORE.

RATIONALE

HPTN 065 was granted a conditional approval from the central IRB, Copernicus Group Independent Review Board (CGIRB), contingent upon several requested minor modifications to the template Patient Computer-Delivered Intervention and Survey Informed Consent Form. As several other minor revisions were deemed necessary as well, a decision was made by the Protocol Chair to move to Version 2.0 of the protocol so as to incorporate all required modifications. The bulk of these changes are non-substantive and do not affect the study design.

SUMMARY OF REVISIONS

General Updates

- "Regulatory Compliance Center (RCC)" was changed to "Regulatory Support Center (RSC)" per DAIDS memorandum dated 18 May 2010.
- Minor grammatical and typographical errors were fixed throughout the document.

Title Page

- The role of Dr. Bernard Branson was clarified as "Protocol Co-Chair."

List of Abbreviations and Acronyms

- New acronyms were added as needed based on the addition of new text.

Protocol Team Roster

- Dr. Fred Gordin was added to the roster as a protocol team member.
- Dr. Nnemdi Kamanu Elias was added to the roster as a protocol team member (to replace the position of Dr. Shannon Hader at the Washington, D.C. Department of Health).
- Contact information for Dr. Shannon Hader was revised.

Section 3.0: Linkage-to-Care

- Language was included referencing an evaluation of surveillance data for a period of one year after the completion of the financial incentive (FI) intervention. This follow-up time period will provide information on the durability of the effect of FIs (Section 3.2).
- HIV test site selection criteria were revised: previously listed criteria were clarified as being “primary” criteria, and language was added to allow additional site selection criteria to be considered if the required number of HIV test sites cannot be chosen based on the primary criteria (Section 3.4).

Section 4.0: Viral Suppression

- Language was included referencing an evaluation of surveillance data for a period of one year after the completion of the FI intervention. This follow-up time period will provide information on the durability of the effect of FIs (Section 4.2).
- HIV care site selection criteria were revised: previously listed criteria were clarified as being “primary” criteria, and language was added to allow additional site selection criteria to be considered if the required number of HIV care sites cannot be chosen based on the primary criteria (Section 4.4).
- The word “the” was deleted throughout the section headers and sub-headers when it occurred before the words “viral suppression,” for grammatical correctness. (Note: this change also appears in the table of contents).

Section 6.0: Survey of Patients and Providers

- Reference to “paired test statistics” was deleted from the paragraph discussing the analysis of provider surveys of knowledge, attitudes, and practices about the use of ART, as pre- and post-provider surveys will not be linked (Section 6.8).

Section 7.0: HIV Surveillance, Routinely-Collected and Other Survey Data

- Language was included allowing the use of other or site provided data as source data for site selection, process, and outcome measures in certain circumstances.

Section 8.0: Administrative Procedures and Operational Considerations

- New required protocol registration template language was incorporated into existing study activation, protocol compliance, and human subjects/ethical considerations language per 05 April 2010 email from the Division of AIDS, after minor modifications to reflect the unique design of the HPTN 065 study (Sections 8.1, 8.4, and 8.5, respectively).
- Language referencing regular review of the study by an external monitoring committee was added to the Study Monitoring section (Section 8.3) in order to better monitor for

evidence of HIV-test-site and HIV-care-site migration due to the availability of financial incentives for linkage-to-care and viral suppression at some sites. Language was also added to this section to clarify the study elements that will be monitored.

Appendix IIA: Patient Computer-Delivered Intervention and Survey Informed Consent Form

- The Patient Informed Consent Form (ICF) was modified as requested by CGIRB (the Central IRB) and DAIDS regulatory review (Appendix IIA):
    - Formatting of the template ICF was changed slightly in order to conform to CGIRB standard template format.
    - Some information was reorganized or slightly revised to improve clarity.
    - A "Subject's Statement of Consent" summarizing the ICF content was added to the subject's signature page.
    - "Authorization to Use and Disclose Personal Health Information for Research" was added to the end of the ICF.

SUMMARY OF CHANGES
INCLUDED IN THE FULL PROTOCOL AMENDMENT OF:

HPTN 065
TLC-Plus: A Study to Evaluate the Feasibility of an Enhanced Test, Link to Care, Plus Treat Approach for HIV Prevention in the United States
Version 2.0 / 15 July 2010

THE AMENDED PROTOCOL IS IDENTIFIED AS:
Version 3.0 / 14 January 2014

Prior to implementation of this amended protocol, HPTN 065 HIV test and care sites will submit this Summary of Changes document, Version 3.0 of the protocol, and the revised site-specific study informed consent forms to either their own Institutional Review Board (IRB) or the central IRB.

RATIONALE

After V2.0 of the HPTN 065 protocol was finalized in July 2010, two Clarification Memos and four Letters of Amendment were issued to revise the protocol. Currently, there are additional revisions being made to the Prevention for Positives section of the protocol to revise the endpoints, end enrollment after approximately 12 months of recruitment, and eliminate the 18-month visit. The endpoints were revised to more accurately reflect the analysis that will be done with data collected via the CARE+ software. Limiting recruitment to approximately 12 months and elimination of the 18-month visit were made as study implementation and recruitment have taken substantially longer than anticipated and resources are limited. The protocol team has verified that the primary and secondary endpoints can be determined with fewer participants and without the 18-month data point. Normally, these changes would be made via a Letter of Amendment; however, a full protocol amendment is being made to incorporate all previous changes, as well as the current revisions, per study sponsor (DAIDS) request.

SUMMARY OF REVISIONS

<u>Protocol Team Roster</u>

- Carlos Allende, Dr. Robert George Chin, Dr. Margo A. Smith, Dr. Nnemdi Kamanu Elias, Dr. Shannon Hader, Angela Fulwood Wood, and Dr. Fabienne Laraque were removed from the protocol team roster. (LoA #1 and LoA #3)

- Ruth Concepcion, Dr. George Pappas, Nanette Benbow, Dr. Jeffrey Meyer, Dr. Becky Grigg, Dr. Kathleen Brady, June Pollydore, and Dr. Vanessa Elharrar were added to the roster as protocol team members. (LoA #1 and LoA #3)
- Contact information was updated in the protocol roster for Dr. Lisa Fitzpatrick, Dr. Theresa Gamble, and Dr. Nirupama Sista. (LoA #3)
- Michael Kharfen replaced Dr. Gregory Pappas as the protocol team member from the DC Department of Health. (Current revision)

Schema

- The schema was revised to reflect that patients with coupons will be given a $25 gift card upon completion of CD4 and viral load (VL) HIV laboratory tests at the initial HIV care visit. A confirmatory HIV test is not required. (LoA #2)
- The schema was revised to change the potential enrollment into the Prevention for Positives and Patient Survey study components. (Current revision)

Section 3.0: Linkage-to-Care

- The Design for Linkage-to-Care section (Section 3.2) was updated to reflect that the test site surveys will collect data from 2009 through 2013. The purpose of these surveys is to collect information on usual clinic practices for HIV testing and linkage-to-care. Baseline data will be collected for the year 2009, and the same survey data will then be collected annually through 2013. (CM #1)
- Section 3.3 was revised to reflect the inclusion of minors, 12 and older, in the linkage-to-care component of the study, who are permitted to consent to HIV care or can be consented for care by a parent/legal guardian according to the New York State or Washington, DC law. This will enable the study to examine this important subgroup of the target population. (LoA #2)

Section 4.0: Viral Suppression

- The Design for Viral Suppression section (Section 4.2) was updated to reflect that care site surveys will collect data from 2009 through 2013. The purpose of these surveys is to collect information on usual clinic practices for viral suppression. Baseline data will be collected for the year 2009, and the same survey data will then be collected annually through 2013. (CM #1)
- Section 4.3 has been revised to reflect the inclusion of minors, 12 and older, in the viral suppression component of the study, who are permitted to consent to HIV care or can be consented for care by a parent/legal guardian according to the New York State or Washington, DC law. This will enable the study to examine this important subgroup of the target population. (LoA #2)

Section 5.0 Prevention for Positives

- Sections 5.3.1 and 5.3.2 were revised to change the minimum time between the patient's last visit to the clinic and the time when the patient is eligible for PfP from four months to seven months. This allows sites with established, well-controlled patients who are seen at less frequent intervals e.g. every 6 months to be eligible for the study. (LoA #3)
- Section 5.6.1 has been revised to allow sites greater flexibility in recruiting subjects for the Prevention for Positives component. (LoA #4)
- All references to the Month 18 visit were removed. (Current revision)
- The primary and secondary endpoints were revised to more accurately reflect the analysis that will be done with data collected via the CARE+ software. (Current revision)
- Enrollment was limited to approximately 12 months of recruitment. (Current revision)
- CD4 and VL values at ART initiation will no longer be collected. (Current revision)
- Brief, anonymous demographic data will be noted (as opposed to asking the participant) for those individuals refusing study participation, to assess comparability to study participants. (Current revision)
- References to analysis of correct condom use were eliminated, as this is not part of the component’s endpoints. (Current revision)
- Section title, “5.10 Human Subjects/Ethical Considerations” added after the 3rd paragraph of Section 5.9.4. (Current revision)

Section 8.0: Administrative Procedures and Operational Considerations

- In order to keep subject confidentiality, NIAID, OHRP, government or regulatory authorities and/or IRB were added as the authorities that have access to the subject’s records. (Current revision)

Appendix IIA: Patient Computer-Delivered Intervention and Survey Informed Consent Form

- Wording describing the Certificate of Confidentiality was corrected to accurately reflect the previously DAIDS-approved verbiage for all HPTN domestic studies. (LoA #1)
- Text was revised to more clearly indicate that only half of the subjects will receive prevention messaging (the intervention group) and may print out a health plan. (LoA #1)

- The wording for the “Persons to Contact for Problems for Questions” section was revised upon request of the central IRB used the study, Copernicus Group IRB. (LoA#1)
- Anonymous health plans will print out for all subjects randomized to the intervention arm at the end of a CARE+ computer session. (CM #2)
- All subjects enrolled will be assessed for depression, suicide and domestic violence during a CARE+ computer session. (CM #2)
- References to the Month 18 visit were removed. (Current revision)

Statistical Analysis Plan

HPTN 065

TLC-Plus: A Study to Evaluate the Feasibility of an Enhanced Test, Link to Care, Plus Treat Approach for HIV Prevention in the United States

Version 1.0

Date:

13 June 2011

**Protocol Chair**	Wafaa El Sadr<br>Bernie Branson

Plan Prepared by

**Protocol Biostatistician**	Deborah Donnell
**Statistical Research Associates**	Geetha Beauchamp
**CDC**	Irene Hall
**Departments of Health**	Lucia Torian – New York City<br>Tiffany West Ojo – Washington DC

This page left intentionally blank.

Table of Contents

1 Summary of Analysis Plan 1

1.1 Assessing HIV Testing 1

1.2 Analysis of linkage to care 2

1.3 Assessing viral suppression 3

1.4 Assessing prevention for positives 3

1.5 Surveillance case data and jurisdictions 4

2 Definitions for Surveillance Data Components 4

2.1 Measures for HIV testing 4

2.1.1 Description of participating facilities 4
2.1.2 Monitoring HIV testing in Emergency Department 5
2.1.3 Monitoring of HIV testing in Inpatients 5
2.1.4 Enhancements to reporting on HIV testing in Hospitals: as available 5
2.1.5 Indirect measure of enhanced HIV testing from surveillance data 6

2.2 Measures for analysis of linkage to care 7

2.2.1 Definition of HPTN065 diagnosed case eligible for linkage to care 7
2.2.2 Definition of linked to care 7
2.2.3 Definition of HPTN065 diagnosed case eligible for linkage to care from a facility 8
2.2.4 Definition of linked to care by facility 8
2.2.5 Definition of established in care at a facility 8
2.2.6 Measure for linkage to care for a facility 8

2.3 Measures for analysis of viral load suppression. 9

2.3.1 Evaluation of Efficacy of Financial Incentives 9
2.3.2 Definition of HPTN065 care-case 9
2.3.3 Definition of viral suppression in a calendar quarter 10
2.3.4 Definition of care-case in care at a facility 11

2.4 Analysis of migration (change between facilities) in care 12

2.4.1 Baseline analysis of testing at multiple facilities (as an indicator of migration) 13

3 APPENDIX: Shell Tables 14

3.1 HIV Testing 14

3.1.1 Hospital Provided Data 14
3.1.2 Surveillance Data 15

3.2 Monitoring of Linkage to Care 17

3.3 Monitoring of Viral Suppression 19

Tables

TABLE A : HIV TESTING IN THE EMERGENCY DEPARTMENTS 14
TABLE B: HIV TESTING IN INPATIENTS 14
TABLE C: HIV POSITIVE TESTING AND NEWLY DIAGNOSED CASES: BY HOSPITAL, BY QUARTER 15
TABLE D: HIV POSITIVE TESTING AND NEWLY DIAGNOSED CASES: BY JURISDICTION, BY QUARTER 15
TABLE E: CLINICAL CHARACTERISTICS OF NEWLY DIAGNOSED CASES: BY JURISDICTION, BY QUARTER 15
TABLE F: DISEASE PROGRESSION FOR NEWLY DIAGNOSED CASES, BY JURISDICTION 16
TABLE G: DEATHS OF KNOWN HIV CASES: BY JURISDICTION 16
TABLE H: POSITIVE WESTERN BLOT IN JURISDICTION: NUMBER OF DIAGNOSED CASES 17
TABLE I: LINKAGE TO CARE OF DIAGNOSED CASES WITHIN 3 AND 6 MONTHS, BY JURISDICTION 17
TABLE J: LINKAGE TO CARE FOR DIAGNOSED CASES WITHIN 3 AND 6 MONTHS, BY FACILITY IN JURISDICTION 18
TABLE K: ESTABLISHED IN CARE FOR DIAGNOSED CASES. 18
TABLE L HPTN065 CARE CASES: POPULATION ELIGIBLE FOR CARE IN JURISDICTION BY QUARTER 19
TABLE M: PROPORTION OF CARE-CASES IN THE JURISDICTION THAT ARE PERSISTENT BETWEEN CALENDAR QUARTERS, BY JURISDICTION 19
TABLE N: PROPORTION OF CARE-CASES WITH VIRAL LOAD SUPPRESSION (VLS) IN JURISDICTION BY QUARTER. 19
TABLE O IN CARE AT A FACILITY: HPTN065 CARE-CASES AND PATTERN OF CARE ASSESSMENT BY QUARTER. 20
TABLE P: DISTRIBUTION BY FACILITY: HPTN065 CARE-CASES BY IN CARE AT A FACILITY: 20
TABLE Q: PROPORTION OF IN CARE POPULATION AT A FACILITY THAT IS PERSISTENT BETWEEN CALENDAR QUARTERS. 21
TABLE R: PROPORTION OF CARE-CASES IN CARE AT A FACILITY WITH VIRAL LOAD SUPPRESSION (VLS), BY FACILITY AND QUARTER 21
TABLE S ACCESS TO CARE PATTERNS FOR CARE-CASES IN CARE AT A FACILITY. 22
TABLE T: CARE EVALUATIONS AT MULTIPLE FACILITIES AMONGST CARE-CASES WITH CARE EVALUATIONS IN AT LEAST TWO QUARTERS 22
TABLE U: IN INTERVENTION CITIES: CARE EVALUATIONS AT MULTIPLE FACILITIES AMONGST THOSE IN CARE AT HPTN065 FACILITIES. 23

1 Summary of Analysis Plan

The HPTN065 study, Test and Linkage to Care Plus, is evaluating four strategies within the two intervention cities (Bronx, New York and Washington, D.C.)

1. Feasibility component: Expanded testing in the Emergency Departments (ED) and In-patient (IP) population of the participating hospitals
2. Randomized component: Linkage to care of newly diagnosed and out of care HIV cases amongst testing sites randomized to financial incentives or standard of care.
3. Randomized component: Adherence to antiretroviral therapy at HIV care facilities randomized to financial incentives or standard of care.
4. Randomized component: Positive prevention in individuals in care randomized to the two arms of the CARE+ intervention

Evaluation of these components will use Department of Health HIV case surveillance data and study collected data.

Within the four non-intervention cities (Chicago, Illinois; Houston, Texas; Miami, Florida; and Philadelphia, Pennsylvania), surveillance data will provide parallel measures of testing uptake, linkage to care and viral suppression within their jurisdictions.

Study surveys in the intervention cities will monitor change in standard of care for testing, linkage and care, and changes in provider and patient attitudes using three instruments:

1. Provider survey: Attitudes about ART in providers providing care to HIV infected patient
2. Patient survey: Attitudes about positive prevention in HIV infected patients
3. Facility survey: Current standard procedures for linkage to care and adherence to ART

Each of the six Departments of Health will complete an annual survey that gathers information on initiatives operating in the city affecting access to HIV testing and HIV care within the jurisdiction in the previous year.

1. Department of Health survey
2. City testing surveys (e.g. BRFSS)

1.1 Assessing HIV Testing

Monitoring of the feasibility component of enhanced HIV testing is primarily descriptive. The expectation is that enhanced HIV testing in emergency departments and inpatient settings in the hospitals will lead to an increase in proportion of patients tested and an increase in number of newly detected HIV infections in the hospitals. Increased testing throughout a jurisdiction will also lead to an increase in newly detected infections and a decrease in the proportion of newly detected infections detected late in course of disease. The possibility of unexpected findings in the trend of newly detected infection must be acknowledged. The pool of people with undetected HIV infection in the US is a mixture ranging from long-term undetected infections amongst people never tested and people with more recent infection that have not re-tested. An increase in HIV testing could lead to an increase in detection of long-term infections, if

targeted to people not previously tested, or to an increase in detection of recent infections if the testing is focused on more frequent testing amongst people who self-identify as being at risk. .

Four sources of data will be used to monitor trends in HIV testing. In the hospital setting, data about HIV testing uptake will be reported directly from individual hospital data systems. Surveillance data will be used in an indirect assessment of increase in HIV testing. In a subset of non-hospital settings that receive support from the Departments of Health, data on testing volume is currently reported. Annual population based surveys (e.g. BRFSS) will assess the proportion of the population that has received HIV testing in the past year.

Monitoring of HIV testing will include reporting of trends in the following components for intervention and non-intervention cities:

1. HIV testing during HPTN065 in hospitals:
    a. Number and proportion of ED visits with HIV testing
    b. Number and proportion of inpatients with HIV testing
2. Indirect assessment of increased HIV testing effects through surveillance data
    a. Number of newly diagnosed cases, by gender, risk, age, race/ethnicity
    b. CD4 count distribution in newly detected cases
    c. Number of newly diagnosed cases by state of disease (including stage 3, AIDS, at and within 12 months of diagnosis and %BED recent)
    d. Number of previously diagnosed cases re-entering care
3. Volume of testing in testing facilities receiving support for testing from publically funded sources.
4. Estimated proportion of community tested for HIV in the last year (community surveys).

1.2 Analysis of linkage to care

In intervention cities, the efficacy of the financial incentive strategy will be evaluated using a trial design that randomizes strategies by testing facility. In addition to a randomized comparison by arm, rates of linkage to care rates in facilities in the non-intervention cities will be reported.

Reporting for monitoring linkage to care in the intervention cities will include:

1. Number and baseline characteristics of facilities engaged in linkage to care evaluation, including current standard of linkage to care.
2. Randomization of facilities: baseline characteristics of the randomized facilities
3. Number diagnosed and thus eligible for linkage to care, and proportion of diagnosed cases captured within the HPTN065 care facilities.
4. Quality of the data for diagnosed cases and linkage.
5. Number and percentage of linkage to care by facility; randomized vs. non-randomized facilities; by arm (Confidential reporting);
6. Report of financial incentives: number and percent of uptake by testing/care facilities.
7. Social harms: facility migration, individual harms by arm.

In intervention and non-intervention cities:

1. Total number diagnosed and eligible for linkage to care.
2. Quality of the data for diagnosed cases and linkage.
3. Number and percentage of linkage to care within jurisdiction, by facility.

1.3 Assessing viral suppression

In intervention cities, the efficacy of the financial incentive strategy will be evaluated using a trial design that randomizes strategies by medical care facility. In addition to a randomized comparison by arm, rates of linkage to care in facilities in the non-intervention cities will be reported.

Reporting for monitoring viral suppression in the intervention cities will include:
1. Number and baseline characteristics of facilities participating in HPTN065
2. Randomization of care facilities: Baseline characteristics by randomization.
3. Total cases eligible for care; number of cases in care at a facility, proportion captured within the HPTN065 care facilities
4. Quality of the data about VL suppression cases and suppression
5. Number and percentage of viral load suppression by facility; randomized vs. non-randomized facilities; By arm (Confidential reporting)
6. Report of financial incentives: number and percent of uptake by care facilities.
7. Social harms: individual harms by arm
8. Migration to FI vs. non-FI facilities after HPTN065 randomization

For intervention and non-intervention cities:

1. Total cases eligible for care;
2. Quality of the data about cases in care and VL suppression
3. Number of cases in care, number of cases in care by facility and percentage of viral load suppression, by demographic characteristics by facility.

1.4 Assessing prevention for positives

In the medical care facilities where CARE+ is implemented, the efficacy of the financial incentive strategy will be evaluated using an individually randomized design within each facility.

Reporting for monitoring the CARE+ intervention for prevention for HIV positive participants, includes only chosen facilities in the intervention cities. Reporting will include:

1. Number and baseline characteristics of facilities engaged in prevention for positive
2. Randomization of facilities
3. Number enrolled in CARE+ by facility
4. Quality of data
5. Baseline characteristics of participants
6. Outcomes by randomization arm (confidential reporting)

1.5 Surveillance case data and jurisdictions

CDC surveillance case reporting and associated funding is currently tied to the allocation of a case to a jurisdiction. A jurisdiction case is defined by the jurisdiction in which the case was first diagnosed. As HPTN065 is an intervention associated with where a person is living and/or receiving care, we are using a definition different from the CDC jurisdiction case and defining "HPTN065 cases" eligible for analysis of linkage to care and viral suppression based on facility of care and current address of residence.

2 Definitions for Surveillance Data Components

Definition of reporting periods

Each of the components of the monitoring plan based on surveillance data will define measures using calendar reporting periods based on annual quarters.

The annual quarters are defined as

Q1: Jan 1 – Mar 31

Q2: Apr 1 – Jun30

Q3: Jul 1 – Sep 30

Q4: Oct 1 – Dec 31.

Definition of facility

A facility is functionally defined by consideration of

- the data capabilities of the facility/institution and the data entered into the surveillance system
- the function operation of the facility and its parent institution

  To report data by facility using surveillance data, facility must be reliably identified within the surveillance system. This occurs only if the institution can separate lab data by facility and that information is transmitted to surveillance. To randomize different strategies (i.e. financial incentive vs. standard of care) in different facilities, the facilities must function as independent entities (e.g. separate providers and staff). Thus some facilities incorporate multiple care or testing sites operating in several locations, others are single locations operating within a single institution

2.1 Measures for HIV testing

2.1.1 Description of participating facilities

Description of the hospitals and facilities included in the reporting of enhanced HIV testing

1. Number and characteristics of hospitals providing data on HIV testing in Emergency Departments (ED) and inpatient (IP) settings.

2. Data available at each hospital about HIV testing in Emergency Department and inpatient setting; assessment of quality (completeness, timeliness)

2.1.2 Monitoring HIV testing in Emergency Department

Monitoring of eligibility for HIV testing in ED

- Number of visits from patients aged 13-64 attending the ED in a given calendar period
- Number of patients aged 13-64 attending the ED in a given calendar period (Only available for EDs with person level tracking)
- Number of patients with blood drawn in ED
- Exclusions:
  - Not eligible for ED testing as a results of trauma/high triage level
  - Already known HIV+ according to hospital defined standards.
- Number of patients receiving HIV test in ED
- Number testing positive in ED

Calendar reporting period: monthly, quarterly and annually
Reporting lag: 28 days

Refer to Appendix:

- Table A : HIV testing in the Emergency Department

2.1.3 Monitoring of HIV testing in Inpatients

- Number of patients aged 13-64 admitted as inpatients.
- Exclusion/Separation of inpatients admitted through ED who received HIV testing in ED (these cases included in ED monitoring)
- Number of IP admissions through ED: proportion tested in ED vs. IP.
- Number of patients receiving HIV test in IP
- Number testing positive in IP

Calendar reporting period: monthly, quarterly and annually.
Reporting lag: 28 days

Refer to Appendix:

- Table B: HIV Testing in Inpatients

2.1.4 Enhancements to reporting on HIV testing in Hospitals: as available

- Breakdown of eligible for testing (facility data):
  - not offered;
  - offered testing: refused, accepted-completed, accepted-not completed.
- Breakdown by age, gender, ethnicity, (facility data)
- Breakdown of HIV infected by prior case/previous knowledge (in and out of care), newly detected (surveillance data)
- How long since last tested: Never; ever - within last year, last 6 mos, last 3 mos (surveillance/TTH data for positives).

2.1.5 Indirect measure of enhanced HIV testing from surveillance data

1. Trends in number of new cases detected
2. Trends in first CD4 of new cases
3. Trends in proportion of newly diagnosed cases by state of disease (including stage 3, AIDS, at and within 12 months of diagnosis and %BED recent)
4. Trends in proportion of HIV cases dying from AIDS

Calendar reporting period: monthly, quarterly and annually.
Reporting lag for newly diagnosed cases: One calendar quarter
Reporting lag for death: Two calendar quarters

Refer to Appendix:

- Table C: HIV positive testing and Newly Diagnosed Cases: By hospital, by quarter
- Table D: HIV positive testing and Newly Diagnosed Cases: By jurisdiction, by quarter
- Table E: Clinical characteristics of Newly Diagnosed Cases: By jurisdiction, by quarter

- Table F: Disease progression for Newly Diagnosed Cases, by jurisdiction
- Table G: Deaths of Known HIV cases: By Jurisdiction

2.2 Measures for analysis of linkage to care

Linkage is measured using laboratory assessment indicators of access to care amongst newly diagnosed HIV cases in the jurisdiction. We will also assess linkage to care amongst known HIV cases who are out of care.

The evaluation of efficacy of financial incentives for linkage to care will use surveillance data to assess linkage to care from each testing facility: there is no direct measurement of those who received financial incentives.

2.2.1 Definition of HPTN065 diagnosed case eligible for linkage to care

1. Newly detected case during year Y, quarter Q
   a. Positive WB in a newly detected case during the calendar period with residential address in jurisdiction
   b. Positive WB in a newly detected case during the calendar period at a facility within the jurisdiction
2. Out of care case seeking care during year Y, Quarter Q
   a. Known case with positive WB during the calendar period with residential address in jurisdiction and no VL/CD4 evaluation in the year prior to WB
   b. Known case with positive WB during the calendar period at a facility within the jurisdiction and no VL/CD4 evaluation in the year prior to WB

Refer to Appendix:

- Table H: Positive Western Blot in jurisdiction: Number of diagnosed cases

2.2.2 Definition of linked to care

Linked to care is defined as

1) A VL or CD4 evaluation within 3 months of a newly diagnosed case (positive WB)
2) A VL or CD4 evaluation within 3 months of an out of care case seeking care (positive WB)

It is intended that the care evaluation be on a date after the positive WB to indicate initiation of care with a provider, distinct from diagnosis. However, excluding CD4 and VL that have the same date as WB may underestimate entry to care as a person might be screened and referred to care where they receive WB, CD4, and VL as their first care visit. Thus linkage to care will include reporting both including and excluding the day of the WB testing.

We will also report care visits that occur within 6 month of diagnosis.

The three month period is taken from the time of confirmed diagnosis by WB.
A reporting lag of 3 months will be used.

Refer to Appendix:

- Table I: Linkage to care of diagnosed cases within 3 and 6 months, by jurisdiction

2.2.3 Definition of HPTN065 diagnosed case eligible for linkage to care from a facility

A diagnosed case eligible for linkage to care from a facility is a diagnosed case where the WB test defining the diagnosed case was performed at that facility.

2.2.4 Definition of linked to care by facility

A case is linked to care for a facility if a diagnosed case testing at a facility is linked to care within 3 months of testing.

Refer to Appendix:

- Table J: Linkage to care for diagnosed cases within 3 and 6 months, by facility

2.2.5 Definition of established in care at a facility

A diagnosed case is defined as established in care at a facility if a VL or CD4 evaluation occurred in two different quarters in the calendar year following the positive WB defining a diagnosed case.

2.2.6 Measure for linkage to care for a facility

Primary Facility Linkage to Care Endpoint for Efficacy Evaluation

The linkage to care endpoint for a facility is defined as the proportion of diagnosed cases in the ith quarter at facility j, linked to care within 3 months:

$$p_{ij} = \frac{l_{ij}}{m_{ij}}$$

where m_{ij} is the number of diagnosed cases (as defined in 2.2.3) from facility j in the ith quarter and l_{ij} is the number of those cases that are linked to care (as defined in 2.2.4).

Analysis of efficacy of linkage to care by arm will be based on the model

$$E(l_j) = \mu + \alpha l_j^b + \beta X_j$$

Where $\boldsymbol{l_j^b}$ is the baseline rate of linkage to care at facility j, $\boldsymbol{X_j}$ indicates the randomization arm of the jth facility, $\boldsymbol{l_j^b}$ is the proportion of cases linked to care from that facility and includes all quarters after initiation of financial incentives for the randomized facilities. Parameters μ, α, β are estimated using binomial regression.

β is the estimate of effectiveness of financial incentives in increasing linkage to care.

Secondary measures:

- Mean time from time of identification of diagnosed case (positive WB) to first care evaluation (VL or CD4).
- Proportion of diagnosed cases established in care

2.3 Measures for analysis of viral load suppression.

Viral load suppression is the primary measure of a patient adhering to antiretroviral therapy in HPTN065. Viral load suppression is a repeated characteristic for each individual, but we expect that for patients on antiretroviral therapy viral loads will be measured at least q 6 month (Medicare reimburses at most on a q 3 month schedule). We will estimate the proportion of patients with viral load suppression by assessing viral load suppression in the surveillance data amongst all those who are eligible for care in each quarter in a jurisdiction.

2.3.1 Evaluation of Efficacy of Financial Incentives

The financial incentives are intended to improve adherence to antiretroviral therapy, through an incentive for achieving viral load suppression at regular care visits for patients on antiretroviral therapy. Surveillance data will be used to assess viral load suppression at each facility, but there is no direct measurement of those who received financial incentives and no information on who is on antiretroviral therapy. Changes in proportion of patients with viral load suppression will be used as the measure of efficacy of the financial incentives.

We will estimate the proportion of patients with viral load suppression at a facility by assessing viral load suppression in the surveillance data amongst all those who are in care at each facility. In care at a facility is defined by evidence of multiple recent visits at the facility (a similar definition is used before a patient would become eligible for FI at a facility). The estimate of facility viral suppression will be conducted for each calendar quarter over all patients defined as in care for that quarter. Financial incentives can be received at most quarterly. We will also assess the persistence of the patient population in adjacent quarters.

Financial incentives will be introduced over a 3-6 month period, as patients attend regular care visits. As effects are not expected to occur immediately, the intervention effect will be assessed in the period 12-24 months after financial incentives are initiated at the facility.

2.3.2 Definition of HPTN065 care-case

A care-case is defined by calendar quarter. Accessed care in last year is defined as had WB/VL/CD in the any of the last 4 quarters up to and including calendar quarter Q, i.e., to evaluate in care for Q1 2009: consider time period between April 1 2008 through end of Mar 31 2009.

An HPTN065 care case for calendar quarter Q is defined as:

1. For jurisdiction cases
    a. All cases that have accessed care in the last year either within the jurisdiction, within the state or in another state, excluding cases known to have relocated to another jurisdiction or died.
2. For cases not in jurisdiction
    a. All cases who have accessed care in the last year within the jurisdiction
3. For cases newly detected in the last quarter
    a. Residential address in the jurisdiction OR
    b. Confirmed WB at a facility within the jurisdiction.

Living in the jurisdiction is a case known to surveillance whose last known address within the 12 months prior to the assessment year is in the jurisdiction, or who was diagnosed in the jurisdiction and not known to have moved out of jurisdiction or died.

Censoring of care-cases for relocation outside of jurisdiction or death

Conditions where a care-case patient is censored since no longer a case in the jurisdiction:

1. When the patient's current address changes so it is no longer in the jurisdiction and they are not accessing care in the jurisdiction. Define quarter for censoring as quarter when new address recorded or last care visit in jurisdiction, whichever is most recent.
2. Death: Define quarter for censoring as quarter when death occurred or last care visit in jurisdiction, whichever is most recent

Refer to Appendix:

- Table L HPTN065 Care Cases: Population eligible for care in jurisdiction by quarter
- Table M: Proportion of care-cases in the jurisdiction that are persistent between calendar quarters, by jurisdiction

2.3.3 Definition of viral suppression in a calendar quarter

A person is considered to be virally suppressed in a calendar quarter if:

1. At least one viral load is conducted in the calendar quarter and all VLs in the quarter are less than 400; OR
2. Viral load was assessed in the previous quarter, AND in the next quarter (but not in the current quarter) and all were less than 400.

Reporting lag for assessment of the rate of viral suppression is a calendar quarter (i.e. viral load suppression rates for Q1 will occur after the close of Q2).

Schematic of viral load suppression for an individual assessment based on pattern of viral load testing

Viral load suppression in $Q_0$is assessed only in individuals who meet the definition of an HPTN065 care case (and are thus considered eligible for care in a jurisdiction for Q_0)

For each HPTN065 care case, viral load suppression in Q_0 is assessed as follows:

- Let x_i be the maximum of all viral loads in the ith quarter for that individual (irrespective of the facility where the viral load was conducted).

Case	Q_{-2}	Q_{-1}	Q_0 (current quarter)	Q_{+1}	Indicator of viral load suppression in Q_0
If VL in current quarter			x_0		If $x_0 < 400$<br>then $v_0 = 1$ else $v_0 = 0$
If VL in previous quarter		x_{-1}	NR	x_{+1}	If $x_{-1} < 400$ and $x_{+1} < 400$<br>then $v_0 = 1$ else $v_0 =0$
If VL before previous quarter		x_{-1}	NR	NR	$v_0 = 0$
	x_{-2}	NR	NR	x_{+1}	$v_0 = 0$

The viral load suppression, v_0, is a calculated indicator variable for viral suppression in the current quarter, where 1 = viral load suppression, and 0 = viral load not suppressed.

Refer to Appendix:

- Table N: Proportion of care-cases with Viral load suppression (VLS) in jurisdiction by Quarter.

2.3.4 Definition of care-case in care at a facility

A person (care-case) is considered in care at a facility for Quarter X if they have viral load or CD4 assessments conducted at that facility in at least two quarters in the most recent year (including Quarter X). Explicitly, the following patterns would meet the definition of care at a facility.

Definition of in care at a facility: Pattern of lab assessment that would meet the definition for in care at a given facility during Quarter X (Q_X). ♦ denotes assessment of viral load or CD4 at a given facility.

Q_{X-3}	Q_{X-2}	Q_{X-1}	Q_X	In Care
♦	♦	♦	♦	Yes
♦	♦		♦	Yes
♦		♦	♦	Yes
♦	♦		♦	Yes
		♦	♦	Yes
	♦		♦	Yes
♦			♦	Yes
♦	♦	♦		Yes
	♦	♦		Yes
♦		♦		Yes
♦	♦			Yes
♦				No
	♦			No
		♦		No
			♦	No

Patients who meet the definition of HPTN065 care-cases who do not satisfy these conditions will be reported as not in care at a facility.

Patients who are defined as in care at multiple facilities will be reported separately and counted in both.

Refer to Appendix

- Table O In care at a facility: HPTN065 care-cases and pattern of care assessment by quarter
- Table P: Distribution by facility: HPTN065 care-cases by in care at a facility:

- Table Q: Proportion of in care population at a facility that is persistent between calendar quarters.

Primary endpoint measure of viral suppression at a facility

The rate of viral load suppression for the jth facility in the ith quarter is defined as:

$$r_{ij} = \frac{1}{N_j} \sum_{k=1}^{N_j} v_{ik}$$

for the Nj patients in care at the facility during the ith quarter, where v_{ik} = 1 if the kth patient is virally suppressed in the quarter, 0 if not.

Analysis for evaluation of efficacy of financial incentives for viral load suppression

Comparison between facilities randomized to FI vs. no-FI will use the following model:

$$E(r_{ij}) = \mu + \alpha r_j^b + \beta X_j$$

Where $\boldsymbol{r_j^b}$ is the baseline rate of viral load suppression at facility j, $\boldsymbol{X_j}$ indicates the randomization arm of the jth facility, i includes quarters 12 to 24 mos after initiation of the FI. Parameters μ, α, β are estimated using generalized estimating equations to account for the correlation between estimates from the same facility. Weighting that appropriately accounts for the precision of the estimates will be used.
β is the estimate of effectiveness of financial incentives for increasing viral load suppression for patients in care.

Refer to Appendix

- Table R: Proportion of care-cases in care at a facility with Viral load suppression (VLS), by facility and quarter

2.4 Analysis of migration (change between facilities) in care

Migration between facilities may occur as a result of financial incentives. We will monitor for change in care facility between FI and no-FI facilities using the lab testing data during the study.

Lab testing patterns are likely to be complex, as HIV infected patients currently access care from different providers, e.g. through hospitals and new care providers, as a result of medical care seeking behaviors independent of the financial incentives. Analysis of lab testing patterns based on cross sectional data from the patients in care at each facility using baseline data (i.e. prior to implementation of financial incentives) will provide a basis for comparison of post FI patterns.

2.4.1 Baseline analysis of testing at multiple facilities (as an indicator of migration)

Hypotheses:

- *Migration to financial incentive facilities will cause an increase in cases in care at a non-FI facility having care evaluations at multiple facilities, compared to baseline levels of care evaluations at multiple facilities.*

- *Migration to financial inventive facilities will cause an increase in the number of patients in care at facilities randomized to financial incentives*

- *Financial incentives will lead to patients seeking care at more than one facility.*

Changes in the proportion of patients accessing care at multiple facilities will be able to be detected earlier than increases in cases with established care or detection of increase in cases with care established at multiple facilities.

Description of access to care patterns for cases eligible for care

For all the HPTN065 care-cases (defined above), report the proportion of care-cases for a given quarter, are: not in care at a facility; in care at a single facility; in care at multiple facilities. For each HPTN065 facility, we will report the proportion of cases with single and multiple facilities of care.

Refer to Appendix

Table S Access to care patterns for care-cases in care at a facility

Baseline assessment of proportion of cases in care at a facility in Quarter X, who have had care assessments at multiple facilities.

The basis of the metric for monitoring migration of care is the number of people in care at a facility who have care evaluations at another facility. The assessment is restricted to those with care evaluations in at least two quarters of the year, and thus could meet the definition of in care at a facility. The reporting is further refined by the number of quarters where reporting occurs, since the probability of assessment at multiple facilities is higher with more quarters with assessments.

Refer to Appendix:

- Table T: Care evaluations at multiple facilities amongst care-cases with care evaluations in at least two quarters
- Table U: In Intervention cities: Care evaluations at multiple facilities amongst those in care at HPTN065 facilities.

There may be a need for more detailed reporting by type of facilities where multiple facilities are being reported: for example, it is possible that hospitals are the major contributor to multiple facilities reporting, whereas it may be uncommon for patients to report care from multiple non-hospital providers.

3 APPENDIX: Shell Tables

3.1 HIV Testing

3.1.1 Hospital Provided Data

Table A : HIV testing in the Emergency Departments

Hospital Name	Number of ED visits	Number of ED visits where blood is drawn	Number of ED visits resulting in hospital admission	# of unique persons tested in ED	Number of HIV tests in ED	Number of HIV-positive results in ED	Number not tested in ED b/c previously diagnosed HIV
	**Required?**	**Required?**	**Required?**	**Required?**	**Required?**	**Required?**	**Required?**
	Y	Y	Y	N	Y	Y	Y
	**Frequency**	**Frequency**	**Frequency**	**Frequency**	**Frequency**	**Frequency**	**Frequency**
	Monthly	Monthly	Monthly	Annual	Monthly	Monthly	Monthly

Table B: HIV Testing in Inpatients

Hospital Name	Number of hospital admissions	# of unique persons tested in hospital admissions	Number of HIV tests in admissions	Number not tested in ED b/c previously diagnosed HIV
	**Required?**	**Required?**	**Required?**	**Required?**
	Y	Protocol Team/SCHARP to decide	Y	Y
	**Frequency**	**Frequency**	**Frequency**	**Frequency**
	Monthly	Monthly	Monthly	Monthly

3.1.2 Surveillance Data

Table C: HIV positive testing and Newly Diagnosed Cases: By hospital, by quarter

Hospital	Time period	Number Testing Positive	Number newly diagnosed	Number previously diagnosed but out of care
Hospital 1	Y1, Q1			
	Y1, Q2			
	Y1, Q3			
	Y1, Q4			

Table D: HIV positive testing and Newly Diagnosed Cases: By jurisdiction, by quarter

Jurisdiction	Time period	Number Testing Positive	Number newly diagnosed	Number previously diagnosed but out of care
Jurisdiction 1	Y1, Q1			
	Y1, Q2			
	Y1, Q3			
	Y1, Q4			

Table E: Clinical characteristics of Newly Diagnosed Cases: By jurisdiction, by quarter

Jurisdiction	Y1, Q1	Y1, Q2	Y1, Q3	Y1, Q4
Number of newly diagnosed cases				
First CD4<br>None<br>Within 3 months<br>3-6 months<br>More than 6 months				
Distribution of CD4 within 3 months<br>N<br>Median (IQR)<br><50<br>51-200<br>201-350<br>>350				
BED<br>Not available<br>Recent<br>Not recent				

Table F: Disease progression for Newly Diagnosed Cases, by jurisdiction

Jurisdiction	Y1, Q1	Y1, Q2	Y1, Q3	Y1, Q4
Number of newly diagnosed cases				
Disease Stage at diagnosis<br>No evaluation<br>< Stage 3<br>Stage 3<br>AIDS				
AIDS within 12 months<br>Not evaluated<br>Not AIDS<br>AIDS				

Table G: Deaths of Known HIV cases: By Jurisdiction

Jurisdiction	Y1, Q1	Y1, Q2	Y1, Q3	Y1, Q4
Number of jurisdiction cases				
Number of deaths				
Cause of death<br>Unknown<br>AIDS related<br>No AIDS related				

3.2 Monitoring of Linkage to Care

Table H: Positive Western Blot in jurisdiction: Number of diagnosed cases

Jurisdiction	Y1, Q1	Y1, Q2	Y1, Q3	Y1, Q4
**Positive WB reported within jurisdiction**				
Number of Positive WB				
Case finding not complete				
Newly detected case				
Known case in jurisdiction<br>In care<br>Out of care				
Known case in state<br>In care<br>Out of care				
Known case out of state<br>In care<br>Out of care				
**Resident jurisdiction cases with Positive WB reported from another jurisdiction**				
Number of positive WB				
Case finding not complete				
Newly detected case				
Known case in jurisdiction<br>In care<br>Out of care				
Known case in state<br>In care<br>Out of care				
Known case out of state<br>In care<br>Out of care				

Table I: Linkage to care of diagnosed cases within 3 and 6 months, by jurisdiction

Jurisdiction	Y1, Q1	Y1, Q2	Y1, Q3	Y1, Q4
Number of diagnosed cases				
No known linkage				
First VL/CD4 within 3 months				
First VL/CD4 3-6 months				
First VL/CD4 after 6 months				

Table J: Linkage to care for diagnosed cases within 3 and 6 months, by facility in jurisdiction

Facility	Y1, Q1	Y1, Q2	Y1, Q3	Y1, Q4
Number of diagnosed cases				
No known linkage				
First VL/CD4 within 3 months				
First VL/CD4 3-6 months				
First VL/CD4 after 6 months				

Reporting of linkage will be stratified by data source as we anticipate different levels of completeness based on whether lab data is directly available to the Department of Health or reported through National Surveillance.

Table K: Established in care for diagnosed cases.

Facility	Y1, Q1	Y1, Q2	Y1, Q3	Y1, Q4
Number of diagnosed cases				
Number diagnosed cases-known 12 mos				
No known CD4/VL evaluations<br>CD4/VL in one quarter<br>CD4/VL in at least 2 quarters				

3.3 Monitoring of Viral Suppression

Table L HPTN065 Care Cases: Population eligible for care in jurisdiction by quarter

Quarter	Jurisdiction case				Other jurisdiction case		Newly detected case in jurisdiction
Resident	In jurisdiction				In jurisdiction	Out of jurisdiction	In jurisdiction
Accessed care in last year:	In jurisdiction	Only outside of jurisdiction, same state	Only in another state	No known access to care	In jurisdiction		
Y1, Q1							
Y1, Q2							
Y1, Q3							
Y1, Q4							

Table M: Proportion of care-cases in the jurisdiction that are persistent between calendar quarters, by jurisdiction

Jurisdiction	Q1 care cases	Q2 care cases	Q3 care cases	Q4 care cases
Total<br>Included in previous Q<br>Not included in previous Q<br>Eval at site in previous year<br>Not eval at site in prev year, not new case<br>New case	N1<br>N2(N2/N1%)<br>N3(N3/N1)<br>N4(N4/N3)<br>N5(N5/N3)<br>N6(N6/N3)			

Table N: Proportion of care-cases with Viral load suppression (VLS) in jurisdiction by Quarter.

Jurisdiction	Jurisdiction case				Not jurisdiction case:
Accessed care in last year	Accessed care in last year in jurisdiction	Only access to care outside of jurisdiction, same state	Only accessed care in another state	No known access to care	Accessed care in last year in jurisdiction
Q1 2009<br>Total number<br>Number with VL<br>Proportion VLS	N1<br>N2(N2/N1%)<br>N3(N3/N2%)				
Q2 2009<br>Total number<br>Number with VL<br>Proportion VLS	N1<br>N2(N2/N1%)<br>N3(N3/N2%)				
Q3 2009					

Table O In care at a facility: HPTN065 care-cases and pattern of care assessment by quarter.

	Q1	Q2	Q3	Q4
Total Eligible for Care	N			
Not in care at a facility	N1 (N1/N%)			
No lab reports	N2(N2/N1)			
Single quarter lab rept	N3(N3/N1)			
Two quarter lab repts	N4(N4/N1)			
In care at a single facility	N5(N5/N%)			
Two quarter lab repts	N6(N6/N5%)			
Three quarter lab repts	N7(N7/N5%)			
Four quarter lab repts	N8(N8/N5%)			
In care at multiple facilities	N9(N9/N)			
Two quarter lab repts				
Three quarter lab repts				
Four quarter lab repts				

Table P: Distribution by facility: HPTN065 care-cases by in care at a facility:

**Facility**	Q1	Q2	Q3	Q4
**Not in care at a facility**	N			
**Facility 1**	N	N		
**Facility 2**				
**Facility 3**				

Table Q: Proportion of in care population at a facility that is persistent between calendar quarters.

Each quarter, the care population for a facility will change (as it is based on having care evaluations in two calendar quarters of the previous year). For each facility, we will monitor the proportion of the population that remains in care (migrates in/out) at that facility.

Facility	Q1 care cases	Q2 care cases	Q3 care cases	Q4 care cases
Facility 1:<br>Total<br>Included in previous Q<br>Not included in previous Q<br>Eval at site in previous year<br>Not eval at site in prev year, not new case<br>New case	 N1<br>N2(N2/N1%)<br>N3(N3/N1)<br>N4(N4/N3)<br>N5(N5/N3)<br><br>N6(N6/N3)			

Table R: Proportion of care-cases in care at a facility with Viral load suppression (VLS), by facility and quarter

	Q1	Q2	Q3	Q4
Facility 1<br>Number in care<br>Number with VLS	 N1<br>N2 (N2/N1%)			

Table S Access to care patterns for care-cases in care at a facility.

Facility	Number of Quarters in previous year with evaluations	All at one facility	More than one care facility
Facility 1	1	N(% of those in sole care at this facility))	N(% of those in care at multiple facilities at this facility)
	2	N	N
	3	N	N
	4	N	N
	Total	N(% of total in care at this facility)	N(% of total in care at this facility)
Facility 2	1`		
	2		
	3		
	4		
Total across facilities	Total	N(% of total in care at this facility)	N(% of total in care at this facility)
	1`		
	2		
	3		

Table T: Care evaluations at multiple facilities amongst care-cases with care evaluations in at least two quarters

	Care assessment in the past year			
	All at one facility	At 2 facilities	At 3 facilities	At more than 3 facilities
Not in care at a facility<br>Multiple lab reports				
In care at a single facility<br>Reports in 2 quarters<br>Reports in 3 quarters<br>Reports in 4 quarters				

In care at multiple facilities<br>Reports in 2 quarters<br>Reports in 3 quarters<br>Reports in 4 quarters				

Table U: In Intervention cities: Care evaluations at multiple facilities amongst those in care at HPTN065 facilities.

	All at one HPTN065 facility	At 2 facilities: both HPTN065	At 2 facilities: one HPTN065, one non-HPTN065	At more than 2 facilities. All HPTN065	At more than 2 facilities. Not all HPTN065
Not in care at an HPTN065 facility<br>Multiple lab reports					
In care at a single HPTN065 facility<br>Reports in 2 quarters<br>Reports in 3 quarters<br>Reports in 4 quarters					
In care at multiple HPTN065 facilities<br>Reports in 2 quarters<br>Reports in 3 quarters<br>Reports in 4 quarters					

Statistical Analysis Plan

HPTN 065

TLC-Plus: A Study to Evaluate the Feasibility of an Enhanced Test, Link to Care, Plus Treat Approach for HIV Prevention in the United States

Version 2.0

Date: **9 September 2014**

Protocol Chair	Wafaa El Sadr<br>Bernie Branson	Plan Prepared by	
		Protocol Biostatistician	Deborah Donnell
		Statistical Research Associates	Geetha Beauchamp
		CDC	Irene Hall<br>Kirsten Mahle Grey<br>Jianmin Li
		Departments of Health	Lucia Torian – New York City<br>Garrett Lum – Washington DC

This page left intentionally blank.

Table of Contents

1 Summary of Analysis Plan ____ 1
1.1 Assessing HIV Testing ____ 1
1.2 Assessing effect of financial incentives for linkage to care ____ 2
1.3 Assessing effect of financial incentives for viral suppression ____ 3
1.4 Assessing the CARE+ intervention for prevention for positives ____ 3
2 Analysis plan for HIV testing ____ 4
2.1 Description of participating facilities ____ 4
2.2 HIV testing self-reported by hospitals ____ 4
2.2.1 HIV testing in Emergency Department ____ 4
2.3 Statistical Analysis ____ 4
2.3.1 HIV testing in Inpatient Setting ____ 5
2.3.2 Enhancements to reporting on HIV testing in Hospitals: as available ____ 6
2.4 Indirect measure of enhanced HIV testing from surveillance data ____ 6
3 Definitions for Surveillance Data ____ 6
3.1 Reporting periods ____ 6
3.2 Definition of facility ____ 6
3.3 Surveillance case data and jurisdictions ____ 7
4 Assessment of Effectiveness of Financial Incentives for Linkage to Care in newly tested HIV-infected patients ____ 7
4.1 Baseline and Intervention Periods ____ 7
4.1.1 Reporting Time Lag ____ 7
4.2 Definition of Linkage to Care Endpoints ____ 8
4.2.1 In Newly DIagnosed ____ 8
4.2.2 In PLWH Re-testers ____ 8
4.3 Statistical analysis for evaluation of Linkage to Care ____ 9
4.3.1 Definition of population (Denominator): Newly diagnosed or retested at randomized facility, resident or non-resident ____ 9
4.3.2 Definition of linkage outcomes (Numerators) ____ 10
4.4 Primary Linkage to Care Endpoint for Efficacy Evaluation ____ 10
4.5 Subgroup Analysis ____ 10
4.6 Modifications arising from Study Execution ____ 11
4.7 Data Tables: Refer to Appendix – Linkage to Care: ____ 11
5 Assessment of effectiveness of financial Incentives for Viral Load Suppression for HIV-infected patients in care. ____ 11
5.1 Assessment periods ____ 12
5.1.1 Viral Suppression ____ 12

5.1.2 Continuity of Care ____ 12
5.1.3 Reporting lag: ____ 12

5.2 Definition of Concepts ____ 13

5.3 Definition of Endpoints ____ 13
5.3.1 Definition of **Viral Suppression Outcome** (Numerator) ____ 13
5.3.2 Definition of Continuity of care outcome (Numerator) ____ 14
5.3.3 Definition of In care at a Facility Definition (Denominator) ____ 14
5.3.4 Primary endpoint measure of viral suppression at a facility ____ 15
5.3.5 Endpoint measure of Continuity of Care at a facility: ____ 15

5.4 Statistical Analysis for evaluation of efficacy of financial incentives for viral load suppression and continuity of care ____ 15
5.4.1 Model for viral suppression ____ 15
5.4.2 Model for Continuity of Care ____ 15

5.5 Subgroup and Sensitivity Analysis ____ 16

5.6 Modification as a result of Study Implementation ____ 16

5.7 Data Tables: Refer to Appendix for Viral Suppression and Continuity of Care ____ 17

6 Assessment of migration (change between facilities) in care ____ 17

6.1 Baseline analysis of testing at multiple facilities (as an indicator of migration) ____ 17

6.2 Definition of Concepts ____ 17
6.2.1 Facility-level Migration ____ 17
6.2.2 Individual-level Migration ____ 18
6.2.3 HIV Care Site Utilization: single vs. multiple facilities ____ 18
6.2.4 HIV Care Site Utilization: number of facilities ____ 19

6.3 Migration Data Tables: Refer to Appendix – Migration ____ 20

7 Assessment of the Effectiveness of Care+ ____ 20

8 Modifications to the Statistical Analysis Plan ____ 20

8.1 Version 1.0 to Version 2.0 ____ 20

1 Summary of Analysis Plan

The HPTN065 study, Test and Linkage to Care Plus, is evaluating four strategies within the two intervention cities (Bronx, New York and Washington, D.C.)

1. Feasibility component: Expanded testing in the Emergency Departments (ED) and In-patient (IP) population of the participating hospitals
2. Randomized component: Linkage to care of newly diagnosed and out of care HIV cases amongst testing sites randomized to financial incentives or standard of care.
3. Randomized component: Adherence to antiretroviral therapy at HIV care facilities randomized to financial incentives or standard of care.
4. Randomized component: Positive prevention in individuals in care randomized to the two arms of the CARE+ intervention

Evaluation of these components will use Department of Health HIV case surveillance data and study collected data.

Within the four non-intervention cities (Chicago, Illinois; Houston, Texas; Miami, Florida; and Philadelphia, Pennsylvania), surveillance data will provide parallel measures of testing uptake, linkage to care and viral suppression within their jurisdictions.

Study surveys in the intervention cities will monitor change in standard of care for testing, linkage and care, and changes in provider and patient attitudes using three instruments:

1. Provider survey: Attitudes about ART in providers providing care to HIV infected patient
2. Patient survey: Attitudes about positive prevention in HIV infected patients
3. Facility survey: Current standard procedures for linkage to care and adherence to ART

Each of the six Departments of Health will complete an annual survey that gathers information on initiatives operating in the city affecting access to HIV testing and HIV care within the jurisdiction in the previous year.

1. Department of Health survey
2. City testing surveys (e.g. BRFSS)

1.1 Assessing HIV Testing

The feasibility component of enhanced HIV testing is primarily descriptive. The expectation is that enhanced HIV testing in emergency departments and inpatient settings in the hospitals will lead to an increase in proportion of patients tested and an increase in number of newly detected HIV infections in the hospitals. Increased testing throughout a jurisdiction will also lead to an increase in newly detected infections and a decrease in the proportion of newly detected infections detected late in course of disease. The possibility of unexpected findings in the trend of newly detected infection must be acknowledged. The pool of people with undetected HIV infection in the US is a mixture ranging from long-term undetected infections amongst people never tested and people with more recent infection that have not re-tested. An increase in HIV testing could lead to an increase in detection of long-term infections, if

targeted to people not previously tested, or to an increase in detection of recent infections if the testing is focused on more frequent testing amongst people who self-identify as being at risk. .

Four sources of data will be used to monitor trends in HIV testing. In the hospital setting, data about HIV testing uptake will be reported directly from individual hospital data systems. Surveillance data will be used in an indirect assessment of increase in HIV testing. In a subset of non-hospital settings that receive support from the Departments of Health, data on testing volume is currently reported. Annual population based surveys (e.g. BRFSS) will assess the proportion of the population that has received HIV testing in the past year.

Assessment of HIV testing will include reporting of trends in the following components for intervention and non-intervention cities:

1. HIV testing during HPTN065 in hospitals:
   a. Number and proportion of ED visits with HIV testing
   b. Number and proportion of inpatients with HIV testing
2. Indirect assessment of increased HIV testing effects through surveillance data:
   a. Number of newly diagnosed cases, by gender, risk, age, race/ethnicity
   b. CD4 count distribution in newly detected cases
   c. Number of newly diagnosed cases by state of disease (including stage 3, AIDS, at and within 12 months of diagnosis and %BED recent)
   d. Number of previously diagnosed cases re-entering care
3. Volume of testing in testing facilities receiving support for testing from publically funded sources.
4. Estimated proportion of community tested for HIV in the last year (community surveys).

1.2 Assessing effect of financial incentives for linkage to care

In intervention cities, the efficacy of the financial incentive strategy will be evaluated using a trial design that randomizes strategies by testing facility. In addition to a randomized comparison by arm, rates of linkage to care rates in facilities in the non-intervention cities will be reported.

Data for linkage to care in the intervention cities will include:

- Number and baseline characteristics of facilities engaged in linkage to care evaluation, including current standard of linkage to care.
- Randomization of facilities: baseline characteristics of the randomized facilities
- Number diagnosed and thus eligible for linkage to care, and proportion of diagnosed cases captured within the HPTN065 care facilities.
- Quality of the data for diagnosed cases and linkage.
- Number and percentage of linkage to care by facility; randomized vs. non-randomized facilities; by arm (Confidential reporting);
- Report of financial incentives: number and percent of uptake by testing/care facilities.

In intervention and non-intervention cities:

- Total number diagnosed and eligible for linkage to care.
- Quality of the data for diagnosed cases and linkage.
- Number and percentage of linkage to care within jurisdiction, by facility.

1.3 Assessing effect of financial incentives for viral suppression

In intervention cities, the efficacy of the financial incentive strategy will be evaluated using a trial design that randomizes strategies by medical care facility. In addition to a randomized comparison by arm, rates of viral suppression in the non-intervention cities will be reported.

Data for monitoring viral suppression in the intervention cities will include:

- Number and baseline characteristics of facilities participating in HPTN065
- Randomization of care facilities: Baseline characteristics by randomization.
- Total cases eligible for care; number of cases in care at a facility, proportion captured within the HPTN065 care facilities
- Quality of the data about VL suppression cases and suppression
- Number and percentage of viral load suppression by facility; randomized vs. non-randomized facilities; By arm (Confidential reporting)
- Report of financial incentives: number and percent of uptake by care facilities.
- Continuity of care ny facility: Randomized facilities by arm (confidential)
- Migration to FI vs. non-FI facilities after HPTN065 randomization

For intervention and non-intervention cities:

- Total cases eligible for care;
- Quality of the data about cases in care and VL suppression
- Number of cases in care, number of cases in care in area facilities, and percentage of viral load suppression overall and by demographic characteristics and facility.

1.4 Assessing the CARE+ intervention for prevention for positives

In the medical care facilities where CARE+ is implemented, the efficacy of the financial incentive strategy will be evaluated using an individually randomized design within each facility.

The CARE+ intervention for prevention for HIV positive participants in conducted in a subset of chosen facilities in the intervention cities. Data collected will include:

- Number and baseline characteristics of facilities engaged in prevention for positive
- Randomization of facilities
- Number enrolled in CARE+ by facility
- Quality of data
- Baseline characteristics of participants
- Outcomes by randomization arm (confidential reporting)

2 Analysis plan for HIV testing

2.1 Description of participating facilities

Description of the hospitals and facilities included in the reporting of enhanced HIV testing

1. Number and characteristics of hospitals providing data on HIV testing in Emergency Departments (ED) and inpatient (IP) settings.
2. Data reported by each hospital about HIV testing in Emergency Department and inpatient setting; assessment of quality (completeness, timeliness)

2.2 HIV testing self-reported by hospitals

2.2.1 HIV testing in Emergency Department

Data for HIV testing in ED

- Number of visits from patients aged 13-64 attending the ED in a given calendar period
- Number of patients aged 13-64 attending the ED in a given calendar period (Only available for EDs with person level tracking)
- Number of patients with blood drawn in ED

Exclusions:

- Not eligible for ED testing as a results of trauma/high triage level
- Already known HIV+ according to hospital defined standards.
- Number of patients receiving HIV test in ED
- Number testing positive in ED

Calendar reporting period: monthly, quarterly and annually

Reporting lag: 28 days

2.3 Statistical Analysis

A. Trend in HIV testing over time

Hospital model: Assess the change in average performance of the hospitals during this period.

$p_{ij} = n_{ij}/N$ are the proportion of visits with tests at the ith hospital in the jth month. Assume a Gaussian distribution for proportion of visits with HIV tests in the quarter, and assume a mixed effects model with a random intercept for each hospital, a fixed effect for each hospital and time as the covariate of interest.. The coefficient of time is the increase in proportion tested at a hospital per month.

Model to test for linear increase over period of intervention:

Fit separate models for DC and NY

$p_{ij} \sim mu + a_i + U_i + \beta\, t_j$ where $U_i \sim N(0, \tau^2)$

The analysis assesses hospitals: the model assesses whether there is an increase in the probability of these hospitals delivering an HIV test.

B. Baseline factors predicting higher HIV testing

- Specific hospital characteristics

The approaches parallel the development above. Assume the covariate X_i is the baseline covariate of interest. An interaction with intercept is added to the model to evaluate whether the level of testing is different for different levels of X_i. Explicitly:

$$logit\ Bin(n_{ij}, N_{ij}) \sim \mu + a_i + U_i + \beta_1 t_j + a_2 X_i \text{ where } U_i \sim N(0, \tau^2).$$

The coefficient a is the increase in rate of testing associated with X_i. Testing $H_0: a_2 = 0$ vs. $H_0: a_2 \neq 0$ corresponds to testing whether X_i is associated with an increase in HIV testing.

C. Baseline factors predicting increase in HIV testing over time
   - Specific hospital characteristics

The approaches parallel the development above. Assume the covariate X_i is the baseline covariate of interest. An interaction with time is added to the model to evaluate whether the rate of increase in testing is different for different levels of X_i. Explicitly:

$$logit\ Bin(n_{ij}, N_{ij}) \sim \mu + a_i + U_i + \beta_1 t_j + \beta_2 X_i t_j \text{ where } U_i \sim N(0, \tau^2)$$

The coefficient β_2 is the monthly increase in rate of testing associated with X_i. Testing $H_0: \beta_2 = 0$ vs. $H_0: \beta_2 \neq 0$ corresponds to testing whether X_i is associated with a change in the increase of HIV tests.

Note: B and C could be combined to simultaneously test for change in level and trend in HIV testing for any given covariate.

D. Time dependent initiation of a factors predicting increase in HIV testing
   - Beginning of new testing strategy
   - Following an implementation intervention by the HPTN065 team

The approaches parallel the development above. Assume the covariate X_{ij} is the time dependent covariate of interest. An interaction with time since initiation is added to the model to evaluate whether there is an increase in testing after initiation of X_i. Explicitly: If t_{i_0} is the time when the initiation began in the ith hospital and $[.]^+$ denotes the positive part.

$$logit\ Bin(n_{ij}, N_{ij}) \sim \mu + a_i + U_i + \beta_1 t_j + a_2 X_{ij} [t_j - t_{i_0}]^+ \text{ where } U_i \sim N(0, \tau^2)$$

The coefficient a_2 is the change in proportion of tests for every month following initiation associated with X_i. Testing $H_0: a_2 = 0$ vs. $H_0: a \neq 0$ corresponds to testing whether X_i is associated with an increase in HIV testing.

2.3.1 *HIV testing in Inpatient Setting*

Data for HIV testing in IP

- Number of patients aged 13-64 admitted as inpatients.
- Exclusion/Separation of inpatients admitted through ED who received HIV testing in ED (these cases included in ED monitoring)
- Number of IP admissions through ED: proportion tested in ED vs. IP.
- Number of patients receiving HIV test in IP
- Number testing positive in IP

Calendar reporting period: monthly, quarterly and annually.

Reporting lag: 28 days

Statistical Analysis

The analytic approach exactly parallels the ED testing and is not repeated here.

2.3.2 Enhancements to reporting on HIV testing in Hospitals: as available

Breakdown of eligible for testing (facility data):
not offered;
offered testing: refused, accepted-completed, accepted-not completed.
Breakdown by age, gender, ethnicity, (facility data)
Breakdown of HIV infected by prior case/previous knowledge (in and out of care), newly detected (surveillance data)
How long since last tested: Never; ever - within last year, last 6 mos, last 3 mos (surveillance/TTH data for positives).

2.4 Indirect measure of enhanced HIV testing from surveillance data

- Trends in number of new cases detected
- Trends in first CD4 of new cases
- Trends in proportion of newly diagnosed cases by state of disease (including stage 3, AIDS, at and within 12 months of diagnosis and %BED recent)
- Trends in proportion of HIV cases dying from AIDS

Calendar reporting period: monthly, quarterly and annually.

- Reporting lag for newly diagnosed cases: One calendar quarter
- Reporting lag for death: Two calendar quarters

3 Definitions for Surveillance Data

3.1 Reporting periods

Each of the components of the monitoring plan based on surveillance data will define measures using calendar reporting periods based on annual quarters.

The annual quarters for a calendar year are defined as:

Q1: Jan 1 – Mar 31

Q2: Apr 1 – Jun30

Q3: Jul 1 – Sep 30

Q4: Oct 1 – Dec 31.

3.2 Definition of facility

A facility is functionally defined by consideration of

- the data capabilities of the facility/institution and the data entered into the surveillance system

- the function operation of the facility and its parent institution

To report data by facility using surveillance data, facility must be reliably identified within the surveillance system. This occurs only if the institution can separate lab data by facility and that information is transmitted to surveillance. To randomize different strategies (i.e. financial incentive vs. standard of care) in different facilities, the facilities must function as independent entities (e.g. separate providers and staff). Thus some facilities incorporate multiple care or testing sites operating in several locations, others are single locations operating within a single institution

3.3 Surveillance case data and jurisdictions

CDC surveillance case reporting and associated funding is currently tied to the allocation of a case to a jurisdiction. A jurisdiction case is defined by the jurisdiction in which the case was first diagnosed. As HPTN065 is an intervention associated with where a person is living and/or receiving care, we are using a definition different from the CDC jurisdiction case and defining “HPTN065 cases” eligible for analysis of linkage to care and viral suppression based on facility of care and current address of residence.

4 Assessment of Effectiveness of Financial Incentives for Linkage to Care in newly tested HIV-infected patients

Linkage to care is measured using laboratory assessment indicators of access to care amongst newly diagnosed HIV cases in the jurisdiction. We will also assess linkage to care amongst known HIV cases who are out of care and retest.

The evaluation of efficacy of financial incentives for linkage to care will use surveillance data to assess linkage to care from each testing facility: there is no direct measurement of linkage amongst those who received financial incentives.

4.1 Baseline and Intervention Periods

Most sites began the Linkage-to-Care component of the study in April 2011; one financial incentive test site did not begin this component of the study until July 12, 2011. Sites had until December 31, 2012, to hand out a coupon for L2C.

Linkage to care data is reported by quarter from surveillance. The study time periods are defined as:

- Baseline: Q2, 2010 to Q1, 2011
- Ramp-up: Q2, 2011 to Q3, 2011
- Intervention period: Q4, 2011 to Q4, 2012

4.1.1 Reporting Time Lag

- For newly diagnosed: Data reported within 6 months from diagnosis (i.e., data available after 2 quarters of the quarter of interest)
- For retesters: Data reported within 6 months from re-test

4.2 Definition of Linkage to Care Endpoints

4.2.1 In Newly DIagnosed

- Initiation of care within 3 months of HIV diagnosis, measured by: >=1 CD4 and/or VL test within (<=) 3 months of diagnosis
- Date of diagnosis defined as earliest diagnosis date known to surveillance
- Cases included are based on WB or other confirmatory test (IFA, p24 Antigen test, HIV isolation test (culture), NAAT [DNA/RNA based]), includes cases with confirmatory test <=3 months of diagnosis date
- Newly diagnosed cases:
  - Bronx: city population diagnosed at Bronx testing facilities
  - DC: city population diagnosed at DC testing facilitiesNote: earliest diagnosis may be based on screening or confirmatory test.

For quality assurance:

- Assess number/percentage of earlier diagnosis dates based on screening tests (cases with confirmatory test <=3 months of diagnosis date)
- Assess time from date of diagnosis to confirmatory test for randomized sites

Note: for cases without WB, VL confirmation may equal entry to care: conduct assessment of the number/percentage of diagnoses not based on WB

- Includes date of diagnosis = date of first CD4 or VL test
- Entry into to care will also be assessed within 6 and 12 months

4.2.2 In PLWH Re-testers

Randomized Re-testers eligible for Linkage to Care

- Initiation of care within 3 months of HIV diagnosis, measured by: >=1 CD4 and/or VL test within (<=) 3 months of diagnosis
- Retester: Had WB or screening test (for sites not conducting WB testing) at randomized testing facility during DATE1 and DATE2 (quarter)
- Exclusion: Death within 3 months of WB
- Retest cases:
  - Bronx: city population diagnosed at Bronx testing facilities
  - DC: city population diagnosed at DC testing facilitiesNote: earliest diagnosis may be based on screening or confirmatory test.

Note: for testing sites with rapid testing only (no WB testing), assure screening tests are entered into surveillance database. Assess WB related to repeat screening tests.

4.3 Statistical analysis for evaluation of Linkage to Care

4.3.1 Definition of population (Denominator): Newly diagnosed or retested at randomized facility, resident or non-resident

Newly Diagnosed: Residents and Non-residents

1. Residents diagnosed at randomized facility during DATE1—DATE2 (quarter)
   - Residence at diagnosis
   - Age >=13
2. Non-residents diagnosed in randomized facility during DATE1—DATE2 (quarter)
   - Not a resident at diagnosis
   - Resident of adjacent areas (MSA for all except NYC; for NYC other 4 NYC boroughs)
   - Age >=13

   Exclusions
   - Death within 3 months of dx

PLWH Re-testers: Residents and Non-residents

TLC Re-testers Linkage to Care Eligible

- Had WB or screening test (for sites not conducting WB testing) at randomized testing facility during DATE1 and DATE2
- Exclusion
  a. Death within 3 months of WB

Note: for testing sites with rapid testing only (no WB testing), assure screening tests are entered into surveillance database. Assess WB related to repeat screening tests.

Resident

1. Resident at time of diagnosis
2. Living with HIV at time of DATE1
3. Did not have a care visit in 12 months prior to DATE1, defined as no CD4 or VL

Exclusions

a. Death during observation period

Non-residents

1. Residents of adjacent areas
2. Did not have a care visit in 12 months prior to DATE1, defined as no CD4 or VL (need to define evidence base)
3. Other conditions:
   a. Evidence of prior lab test in area (prior to DATE1 minus 12 months)

4.3.2 Definition of linkage outcomes (Numerators)

Linked to Care

- CD4 and/or VL test within 3 months of date of diagnosis or repeat test

4.4 Primary Linkage to Care Endpoint for Efficacy Evaluation

The linkage to care endpoint for a facility is defined as the proportion of diagnosed cases during the assessment period at facility *j*, linked to care within 3 months:

$$l_j = \frac{\sum_i n_{ij}}{\sum_i N_{ij}}$$

where N_{ij} is the number of diagnosed cases from facility *j* in the ith quarter and n_{ij} is the number of those cases that are linked to care.

Analysis of efficacy of linkage to care by arm will be based on the model

$$E\left(\log l_j\right) = \mu + \alpha \log \boldsymbol{l_j^b} + \beta X_j$$

Assume that $E\left(\log l_j \mid l_i^b, T_i\right)$ $is\ N(a_i + \beta, W\ \sigma^2)$

Where $\boldsymbol{l_j^b}$ is the baseline rate of linkage to care at facility *j*, $\boldsymbol{X_j}$ indicates the randomization arm of the jth facility, $\boldsymbol{l_j}$ is the proportion of cases linked to care from that during the assessment period. Parameters μ, α, β are estimated using logistic regression, with GEE methods used to account for within site correlation assuming an exchangeable correlation structure.

The statistical test for the effectiveness of the intervention is $H_0: \beta = 0$ vs $H_1: \beta \neq 0$. β is the estimate of effectiveness of financial incentives in increasing log odds of linkage to care.

4.5 Subgroup Analysis

The following groups of participants will be compared

- All sites: residents and non-residents
- All sites: residents only
- All sites: Newly diagnosed and retesters

The following site subgroups will be assessed

- Type of testing provider: Community based vs not community based
- Hospital vs non-hospital
- Site population is primarily MSM vs not primarily MSM

4.6 Modifications arising from Study Execution

- Several sites had 0% and 100% estimate of baseline linkage, often as a result of a low number of cases. The Median Unbiased Estimator was used to estimate the baseline p, which has the property of not estimating a probability of 0% or 100% as the baseline estimate. In simulated power scenarios, the MUE estimate was consistently more powerful than other maximum likelihood estimates. A logistic transformation was used for the baseline linkage covariate.
- Sites were excluded if they had zero cases for linkage during the intervention period in the surveillance data. Excluded sites were:

Site 204	FI	Bronx
Site 215	Non-FI	Bronx
Site 114	Non-FI	DC

- Sites 212 and 214 were randomized together.
- The final analysis of the FI for linkage to care is a cluster randomized analysis of 34 sites (16 in the Bronx and 18 in DC), 18 in the FI arm (8 from the Bronx, 10 from DC and) and 16 in the non-FI arm (8 from Bronx, 8 from DC)

4.7 Data Tables: Refer to Appendix – Linkage to Care:

5 Assessment of effectiveness of financial Incentives for Viral Load Suppression for HIV-infected patients in care.

Viral load suppression is the primary measure of a patient adhering to antiretroviral therapy in HPTN065. Viral load suppression is a repeated characteristic for each individual, but we expect that for patients on antiretroviral therapy viral loads will be measured at least q 6 month (Medicare reimburses at most on a q 3 month schedule). We will estimate the proportion of patients with viral load suppression by assessing viral load suppression in the surveillance data amongst all those who are eligible for care in each quarter in a jurisdiction.

The financial incentives are intended to improve adherence to antiretroviral therapy, through an incentive for achieving viral load suppression at regular care visits for patients on antiretroviral therapy. Surveillance data will be used to assess viral load suppression at each facility, but there is no direct measurement of those who received financial incentives and no information on who is on antiretroviral therapy. Changes in proportion of patients with viral load suppression will be used as the measure of efficacy of the financial incentives.

We will estimate the proportion of patients with viral load suppression at a facility by assessing viral load suppression in the surveillance data amongst all those who are in care at each facility. In care at a facility is defined by evidence of multiple recent visits at the facility (a similar definition is used before a patient would become eligible for FI at a facility). The estimate of facility viral suppression will be conducted for each calendar quarter over all patients defined as in care for that quarter. Financial incentives can be received at most quarterly. We will also assess the persistence of the patient population in adjacent quarters.

The use of financial incentives for viral suppression may also increase regularity of care visits, referred to as "Continuity of care." The distribution of quarterly gift cards for suppressed viral load may incentivize participants in clinics distributing financial incentives vs clinics without FI to maintain regular quarterly visits. The effect of financial incentives on this endpoint will also be evaluated

Financial incentives will be introduced over a 3-6 month period, as patients attend regular care visits. As effects are not expected to occur immediately, the intervention effect will be assessed in the period 12-24 months after financial incentives are initiated at the facility.

5.1 Assessment periods

5.1.1 Viral Suppression

The Viral Suppression component began at 10 DC care sites on February 1, 2011; on March 1, 2011 at one Bronx care site; and on April 1, 2011 at the remaining 28 DC and Bronx care sites (total participating care sites = 39). The last gift card for VS could have been given for a VL done up until Jan 31, 2013 (5 sites), Feb 28, 2013 (1 site), March 31, 2013 (13 sites).

Rationale: three quarters are allowed for ramp up of the intervention effect, following the last sites to initiate financial incentives.

Viral load data is reported by quarter from surveillance. The study periods are defined as:

a. Baseline: Calendar Q1, 2010–Q1, 2011.
b. Ramp-up: Q2, 2011 to Q4 2011
c. Intervention: Q1 2012 to Q1 2013
d. Peak Intervention: Q4 2012.

5.1.2 Continuity of Care

Continuity of care is reported by quarter from surveillance. Because continuity of care involved behaviors over the prior 5 quarters, evaluation begins in the 5th quarter after the intervention. The study periods are defined as:

a. Baseline: Calendar Q1, 2010 –Q1, 2011.
b. Ramp-up: Q2, 2011 to Q2, 2012
c. Intervention: Q3 2012 to Q1 2013 (3 quarters)

5.1.3 Reporting lag:

Two calendar quarter (i.e. viral load suppression rates for Q0 will occur after the close of Q2) Baseline data can be provided starting with Y10,Q1 (jurisdictions to have lab data available from at least January 1, 2009; this allows the required 15 months to determine the in-care population at end of Y10,Q1). Therefore, there will be at least one year (4 quarters) of baseline data before study start quarter Y11,Q1.

5.2 Definition of Concepts

In intervention cities, the efficacy of the financial incentive strategy will be evaluated using a trial design that randomizes strategies by medical care facility. In addition to a randomized comparison by arm, the percentage of persons with viral suppression in the non-intervention cities will be reported.

Reporting for monitoring viral suppression in the intervention cities using surveillance data will include:

- Total cases eligible for care; number of cases in care at a facility, proportion captured within the HPTN065 care facilities
- Quality of the data about VL suppression cases and suppression
- Number and percentage of viral load suppression by facility; randomized vs. non-randomized facilities; By arm (Confidential reporting)

For intervention and non-intervention cities:

- Total cases eligible for care;
- Quality of the data about cases in care and VL suppression
- Number of cases in care, number of cases in care by facility (if available) and percentage of viral load suppression, by demographic characteristics by facility.

5.3 Definition of Endpoints

5.3.1 *Definition of **Viral Suppression Outcome** (Numerator)*

- At least one VL result in quarter and all VL in quarter are <400, or
- Viral load was assessed in the previous quarter and d all were less than 400

Viral load suppression in Q_0 is assessed as follows:

- Let x_i be the maximum of all viral loads in the ith quarter for that individual (irrespective of the facility where the viral load was conducted).

Viral Suppression Definition Table (for cases in care)

Case	Q_{-2}	Q_{-1}	Q_0 (current quarter)	Indicator of viral load suppression in Q_0
If VL in current quarter			x_0	If $x_0 < 400$<br>then $v_0 = 1$ else $v_0 = 0$
If VL in previous quarter		x_{-1}	NR	If $x_{-1} < 400$<br>then $v_0 = 1$ else $v_0 =0$
If VL before previous quarter	x_{-2}	NR	NR	$v_0 = 0$

The viral load suppression, v0, is a calculated indicator variable for viral suppression in the current quarter, where 1 = viral load suppression, and 0 = viral load not suppressed.

Measure of site level viral load suppression: $V_{it} = v_{it} / N_{it}$; where N_{it} = number of facility's patients engaged in care, in the ith facility and the tth calendar quarter, and v_{it} is the number of those patients who are currently virally suppressed.

Baseline viral load suppression: The median unbiased estimate of the proportion of quarterly visits virally suppressed during the baseline period.

5.3.2 Definition of Continuity of care outcome (Numerator)

Continuity of Care in a facility

- At least four care visits at the facility in the previous five quarters amongst those in care at that facility
- Care visits are visits with a CD4 or VL

An Alternative definition for continuity of care was also analysed:

- At least three care visits at the facility in the previous five quarters amongst those in care at that facility

5.3.3 Definition of In care at a Facility Definition (Denominator)

A person (care-case) is considered in care at a facility for Quarter X if they have viral load or CD4 assessments conducted at that facility in at least two quarters in the most recent year (including Quarter X). Explicitly, the following patterns would meet the definition of care at a facility.

Definition of in care at a facility: Pattern of lab assessment that would meet the definition for in care at a given facility during Quarter i (Qi). ♦ denotes assessment of viral load or CD4 at a given facility.

Q_{i-3}	Q_{i-2}	Q_{i-1}	Q_i	In Care
♦	♦	♦	♦	Yes
♦	♦		♦	Yes
♦		♦	♦	Yes
♦	♦		♦	Yes
		♦	♦	Yes
	♦		♦	Yes
♦			♦	Yes
♦	♦	♦		Yes
	♦	♦		Yes
♦		♦		Yes
♦	♦			Yes
♦				No
	♦			No
		♦		No
			♦	No

Patients who meet the definition of HPTN065 care-cases who do not satisfy these conditions will be reported as not in care at a facility.

Patients who are defined as in care at multiple facilities will be reported separately and counted in both.

5.3.4 *Primary endpoint measure of viral suppression at a facility*

The proportion of participants with viral load suppression for the jth facility in the ith quarter is defined as:

$$r_{ij} = \frac{1}{N_j} \sum_{k=1}^{N_j} v_{ik}$$

for the Nj patients in care at the facility during the ith quarter, where v_{ik} = 1 if the kth patient is virally suppressed in the quarter, 0 if not.

5.3.5 *Endpoint measure of Continuity of Care at a facility:*

The proportion of participants with continuity of care for the jth facility in the ith quarter is defined as $C_{it} = y_{it} / N_{it}$,; where N_{it} = number of facility's patients engaged in care, in the ith facility and the tth calendar quarter, using the same definition of patients in care as for the viral load suppression endpoint, and y_{it} is the number of those patients who have had had lab evaluations in at least 4 of the last 5 quarters.

Covariate for Baseline continuity of care is the median unbiased estimate of the proportion of quarterly visits with person having care continuity during the baseline period.

5.4 Statistical Analysis for evaluation of efficacy of financial incentives for viral load suppression and continuity of care

5.4.1 *Model for viral suppression*

Comparison between facilities randomized to FI vs. no-FI will use the following model:

$$E(r_{ij}) = \mu + \alpha r_j^b + \beta X_j$$

Where $\boldsymbol{r_j^b}$ is the baseline rate of viral load suppression at facility j, $\boldsymbol{X_j}$ indicates the randomization arm of the jth facility, i includes quarters In the intervention assessment period. Parameters μ, α, β are estimated using generalized estimating equations to account for the correlation between observations from the same facility in different quarters. Weighting that appropriately accounts for the precision of the estimates will be used: explicitly, estimation is weighted by the mean number of patients at a site during the assessment period.

β is the estimate of effectiveness of financial incentives: the increase in the proportion of patients in care with viral load suppression, adjusted for baseline proportion with viral load suppression.

5.4.2 *Model for Continuity of Care*

The continuity in care model is the same as for viral load suppression, with a shorter evaluation period, and the addition of an alternate definition.

5.5 Subgroup and Sensitivity Analysis

The primary endpoint for viral load suppression is proportion of resident and non-resident cases in care at each TLC facility who are virally suppressed during the intervention assessment period. Secondary sensitivity analyses considered data that was likely to be of slightly higher completeness because of the surveillance data standards, in which resident cases receive a greater level of scrutiny by the DoH. An analysis that considered only the final quarter in which the assessment was in place throughout the entire quarter at all sites will assess the effect of the intervention at the peak of the implementation

1. Primary: Residents and non-residents, entire intervention assessment period
    a. Sensitivity 1: Residents, entire intervention assessment period
    b. Sensitivity 2: Residents and non-residents, final full intervention assessment quarter (Q4 2011)

Subgroup of participants:

Residents and non-residents who were not fully virally suppressed during 2010, the year before the intervention. Cases were excluded from this analysis if they were suppressed in all four quarters of 2010.

Site subgroups

1. By City
2. By baseline clinic size (<= median size in Q1 2011). The median size was 196 patients
3. By clinic type (Hospital vs. community)
4. By baseline viral load suppression (<=median site VLS in Baseline data). The median viral load suppression was 66%

Supportive analyses:

1. Examine trend over time of the effect of the intervention during intervention period
2. Examine trend over time of the effect of the intervention during ramp up and intervention periods

5.6 Modification as a result of Study Implementation

- Sites 155 and 156 were combined as the data in eHARS from these two sites at Children’s Hospital in DC could not be separated
- Site 153 was omitted because the mechanisms through which viral loads were entered into eHARS appeared strongly biased toward patients who had viral loads assessed during the intervention period.

5.7 Data Tables: Refer to Appendix for Viral Suppression and Continuity of Care

6 Assessment of migration (change between facilities) in care

Migration between facilities may occur as a result of financial incentives. We will monitor for change in care facility between FI and no-FI facilities using the lab testing data during the study.

Lab testing patterns are likely to be complex, as HIV infected patients currently access care from different providers, e.g. through hospitals and new care providers, as a result of medical care seeking behaviors independent of the financial incentives. Analysis of lab testing patterns based on cross sectional data from the patients in care at each facility using baseline data (i.e. prior to implementation of financial incentives) will provide a basis for comparison of post FI patterns.

6.1 Baseline analysis of testing at multiple facilities (as an indicator of migration)

Hypotheses:

- *Migration to financial incentive facilities will cause an increase in cases in care at a non-FI facility having care evaluations at multiple facilities, compared to baseline levels of care evaluations at multiple facilities.*

- *Migration to financial incentive facilities will cause an increase in the number of patients in care at facilities randomized to financial incentives*

- *Financial incentives will lead to patients seeking care at more than one facility.*

Changes in the proportion of patients accessing care at multiple facilities will be able to be detected earlier than increases in cases with established care or detection of increase in cases with care established at multiple facilities.

6.2 Definition of Concepts

Patient migration has a complex, two-part definition. Migration is measured at the facility ("health care site migration") and individual level ("health care site utilization"), with different definitions and indicators for each.

Baseline migration analysis will establish baseline patterns of variation in health care site census and health care site utilization by individuals and to use this baseline to aid in detecting significant changes citywide, within arms, and by facility.

6.2.1 Facility-level Migration

Analyses:

- Aggregate citywide census variation
- Aggregate randomized facility census variation
- Aggregate non-randomized facility census variation
- Aggregate randomized facility FI
- Aggregate non-FI facility variation

- randomized single facility variation

Population:

Citywide analysis: all standardized facilities with mean quarterly census of >=50 patients in year preceding Yj,Qi, but including all randomized care sites regardless of patient census

Analysis: All participating HIV care sites

Indicator: Number of unique individuals with ≥1 VL or CD4 per time period (quarter)

Measurement interval: first report two quarters after beginning of randomization, report quarterly thereafter

6.2.2 Individual-level Migration

Census of patients in active care

Analyses:

- Aggregate citywide census variation
- Aggregate randomized facility census variation
- Aggregate non-randomized facility census variation
- Aggregate randomized facility FI
- Aggregate non-FI facility variation
- randomized single facility variation

Population:

Persons who had at least two care visits at least 3 months apart in the 12 months preceding and including Yj,Qi (15 months period)
Facilities within jurisdiction

Indicator: Number of individuals engaged in care in Yj,Qi: >=1 CD4 or VL in Yj,Qi

Definitions:

Single facility utilization = all CD4 and VL in the 15 month period were ordered from one facility

6.2.3 HIV Care Site Utilization: single vs. multiple facilities

Analyses: Aggregate citywide variation

Aggregate randomized facility variation

Aggregate non-randomized facility variation

Aggregate randomized facility FI

Aggregate non-FI facility variation

randomized single facility variation

Population:

- Persons who had at least two care visits at least 3 months apart in the 12 months preceding and including Yj,Qi (15 months period)

- Alive at end of Yj,Qi
- No evidence of moved out of jurisdiction by end of Yj,Qi (based on RIDR or current address)

Indicator:

Number of individuals with 2, 3, or 4 or more evaluations (number of CD4 and VL reports) in 15 months period (12 months preceding and including Yj,Qi)

Definitions:

Single facility = all VLs and CD4s in time period ordered by same facility/HIV care site
Multiple facilities = VLs and CD4s in time period ordered by >1 HIV care site

6.2.4 HIV Care Site Utilization: number of facilities

Analyses: Citywide variation

randomized facilities variation

Combination randomized and non-randomized facilities

Population:

Persons who had at least two care visits at least 3 months apart in the 12 months preceding and including Yj,Qi (15 months period)

Alive at end of Yj,Qi

No evidence of moved out of jurisdiction by end of Yj,Qi (based on RIDR or current address)

Indicator:

Number of individuals with 2, 3, or 4 or more evaluations (number of CD4 and VL reports) in 15 months period (12 months preceding and including Yj,Qi)

Definitions:

Single facility = all VLs and CD4s in time period ordered by same facility/HIV care site

Justification:

Change in clinical dialogue with patient re: initiation of ART (patient who can receive payment may want to change ART strategy or change previous decision to delay initiation of ART so that he can be reimbursed for undetectable VL)

Potential negative effect on good patient management if patient seeing multiple uncoordinated health care sites who do not have access to each other's VL and CD4 results or knowledge of the ART regimen prescribed

Known reasons for multiple HIV Care Site Utilization:

- Jail during intervention = reasonable narrative re: disrupted care (ID via NYSID, corrections match)
- Pregnancy and/or delivery during study = reasonable narrative (match to EPS)
- Hospitalizations and ER visits during study (how to distinguish these from regular HIV clinic visits if medical home is hospital based)
- Relocation (definition = address at last lab different from address at diagnosis) is legitimate reason for movement to new health care site, but is it a reasonable explanation for double utilization?HMO requirement for regular HMO visit for referral

Data and Coding

- Data used with one quarter reporting lag (e.g., for assessing Y1,Q1, use data reported through Y1,Q2)
- For dates missing day, use month and year; exclude if month is missing.

6.3 Migration Data Tables: Refer to Appendix – Migration

7 Assessment of the Effectiveness of Care+

The statistical analysis plan for this component of the HPTN 065 study is in a separate document.

8 Modifications to the Statistical Analysis Plan

8.1 Version 1.0 to Version 2.0

Summary of Changes

1. Updated Personnel: New Head of Surveillance
2. Revision/reorganization of sections: Version 2.0 has separate sections for each of the study component endpoints: 1) HIV testing, 2) Linkage to Care, 3) Viral suppression and Continuity of Care, 4) Migration and 5) Positive Prevention (Care+)
3. Shell Table replaced by separate Appendices for Data Table for each of the Endpoints
4. Simplification of assessment of the viral suppression endpoint: remove requirement for viral suppression in the "future" quarter for those with no viral load testing in the current quarter. Viral load assessed and suppressed in the current or prior quarter is evaluated as suppressed.
5. Addition of new endpoint: Continuity of Care
6. Determination of the baseline and assessment periods for Linkage to Care, Viral Suppression and Continuity of Acre

7. Addition of site subgroup and participant subgroup analyses for Linkage to Care and Viral Suppression
8. Modifications in the sites included in final analysis, resulting from study conduct and data access, as described.
