# Supplementary material for: Financial Incentives for Linkage to Care and Viral Suppression Among HIV-Positive Patients: A Randomized Clinical Trial (HPTN 065)
Source: JAMA Intern Med. 2017 Aug 7;177(8):1083–92. doi: 10.1001/jamainternmed.2017.2158 (PMC5604092; doi:10.1001/jamainternmed.2017.2158)
Supplement: Supplement 2. — eTable 1. Baseline Characteristics of HIV Test and Care Sites, Bronx, NY and Washington, DC, 2010 and 2011 eTable 2. Baseline Characteristics of HIV-positive Patients at HIV Test Sites by Study Arm and Overall eTable 3. Baseline Characteristics of Patients at HIV Care Sites by Study Arm and Overall eTable 4. Baseline HIV Care Site Level Characteristics for Viral Suppression (VS) and Continuity in Care (CC) for Patients in Care eTable 5. Effect of Financial Incentives (FI) and Standard-of-Care (SOC) on Viral Load Suppression (VS) for Patients at Peak Intervention Period eFigure 1. Change in Proportion Virally Suppressed over Time by Study Arm [file jamainternmed-177-1083-s002.pdf]

Supplementary Online Content

El-Sadr WM, Donnell D, Beauchamp G, et al. Financial incentives for linkage to care and viral suppression among HIV-positive patients: a randomized clinical trial (HPTN 065) [published online June 19, 2017]. *JAMA Intern Med*. doi:10.1001/jamainternmed.2017.2158

eTable 1: Baseline Characteristics of HIV Test and Care Sites, Bronx, NY and Washington, DC, 2010 and 2011

eTable 2: Baseline Characteristics of HIV-positive Patients at HIV Test Sites by Study Arm and Overall

eTable 3: Baseline Characteristics of Patients at HIV Care Sites by Study Arm and Overall

eTable 4: Baseline HIV Care Site Level Characteristics for Viral Suppression (VS) and Continuity in Care (CC) for Patients in Care

eTable 5: Effect of Financial Incentives (FI) and Standard-of-Care (SOC) on Viral Load Suppression (VS) for Patients at Peak Intervention Period

eFigure 1: Change in Proportion Virally Suppressed over Time by Study Arm

This supplementary material has been provided by the authors to give readers additional information about their work.

Copyright 2017 El-Sadr WM et al. JAMA Internal Medicine.

eTable 1: Baseline characteristics of HIV Test and Care Sites, Bronx, NY and Washington, DC, 2010 and 2011

	Number or Site mean (SD)		
	**Financial Incentives**	**Standard of Care**	**Overall**
**HIV Test sites**[a]	**18**	**16**	**34**
Bronx, NY	8	8	16
Washington, DC	10	8	18
Hospital-based sites	8	7	15
Community-based sites	10	9	19
Baseline number of HIV-positive cases	39 (58)	32 (25)	35 (45)
Proportion linked to care	75% (30%)	72% (27%)	74% (29%)
**HIV Care sites**[b]	**17**	**20**	**37**
Bronx, NY	10	10	20
Washington, DC	7	10	17
Hospital-based sites	7	7	14
Community-based sites	10	13	23
Quarterly average number of HIV-positive in care at baseline	410 (543)	344 (427)	374 (478)
Baseline viral load assessment in previous 6 months	84% (15%)	92% (4%)	88% (11)
Baseline viral load suppression	58% (18%)	66% (13%)	62% (16%)
Baseline continuity-of-care	41% (21%)	48% (17%)	45% (19%)

[a] For linkage to care, baseline was assessed among the linkage-to-care cases in surveillance during April 1, 2010 to March 31, 2011. HIV-positive persons included if either newly-detected HIV infection (based on new report to surveillance system of a positive Western blot) or had previously diagnosed infection, but were out of care (evidenced by no reported HIV-related laboratory tests) in prior year.
[b] For viral suppression and continuity-in-care, baseline was assessed in HIV-positive patients in care during Jan 1 2010 to March 31, 2011 according to surveillance system.

Copyright 2017 El-Sadr WM et al. JAMA Internal Medicine.

eTable 2: Baseline Characteristics of HIV-positive Patients at HIV Test Sites by Study Arm and Overall

	Mean (SD)		
	**Financial Incentives**	**Standard of Care**	**Overall**
**HIV Test Sites**	18	16	34
**Age (years)**			
13-24	25% (25)	25% (25)	25% (25)
25-34	27% (15)	29% (24)	28% (19)
35-44	15% (11)	20% (14)	17% (13)
45-54	24% (19)	21% (15)	22% (17)
≥55	10% (15)	6% (7)	8% (12)
**Gender**			
Men	71% (29)	76% (20)	73% (25)
Women	29% (29)	24% (20)	27% (25)
**Race**			
Black/African American	68% (31)	60% (33)	65% (32)
Hispanic/Latino	25% (32)	35% (35)	30% (33)
White	6% (9)	3% (5)	5% (7)
Multi races/Other	1% (2)	1% (2)	1% (2)
**Male Transmission Mode Category**			
Male-to-Male Sexual contact	42% (28)	39% (28)	41% (28)
Male Injection Drug Use	3% (5)	11% (18)	6% (13)
Male Heterosexual contact	10% (17)	7% (6)	9% (13)
Male-to-Male Sexual contact and Injection Drug Use	1% (2)	5% (12)	3% (9)
Male Other	15% (17)	14% (13)	15% (15)
**Female Transmission Mode Category**			
Female Heterosexual contact	5% (7)	7% (11)	6% (9)
Female Injection Drug Use	3% (8)	2% (3)	3% (6)
Female Other	21% (27)	15% (13)	18% (22)
**Stage of Disease**			
Stage 1 (CD4 > 500 copies/mL)	17% (13)	11% (10)	14% (12)
Stage 2(CD4 > 350-499 copies/mL)	12% (11)	15% (11)	14% (11)
Stage 3(CD4 > 200-349 copies/mL)	16% (14)	19% (23)	18% (19)
Stage 4(CD4 < 200 cp/mL or OI)	25% (19)	27% (16)	26% (17)
Stage Unknown	30% (35)	27% (27)	29% (31)

Copyright 2017 El-Sadr WM et al. JAMA Internal Medicine.

eTable 3: Baseline Characteristics of Patients at HIV Care Sites by Study Arm and Overall

	Mean (SD)		
	Financial Incentives	Standard of Care	Overall
**HIV Care Sites**	17	20	37
**Age (Years)**			
13-24	9% (23)	10% (22)	10% (23)
25-34	12% (8)	15% (8)	14% (8)
35-44	26% (10)	27% (8)	26% (9)
45-54	35% (12)	32% (11)	33% (11)
≥55	18% (11)	17% (8)	17% (9)
**Gender**			
Men	63% (19)	64% (17)	64% (18)
Women	37% (19)	36% (17)	36% (18)
**Race**			
Black/African American	57% (23)	58% (26)	57% (24)
Hispanic/Latino	33% (24)	31% (26)	32% (25)
White	8% (17)	9% (18)	9% (17)
Multi races/Other	2% (1)	2% (2)	2% (2)
**Male Transmission Mode Category**			
Male-to-Male Sexual contact	24% (22)	29% (22)	27% (22)
Male Injection Drug Use	13% (11)	11% (10)	12% (10)
Male Heterosexual Contact	7% (4)	8% (6)	7% (5)
Male-to-Male Sexual Contact and Injection Drug Use	3% (3)	4% (3)	4% (3)
Male Other	16% (11)	12% (7)	14% (9)
**Female Transmission Mode Category**			
Female Heterosexual Contact	14% (11)	14% (6)	14% (8)
Female Injection Drug Use	9% (7)	8% (8)	8% (8)
Female Other	14% (10)	14% (12)	14% (11)

Copyright 2017 El-Sadr WM et al. JAMA Internal Medicine.

eTable 4: Baseline HIV Care Site Level Characteristics for Viral Suppression (VS) and Continuity in Care (CC) for Patients in Care

		Site mean (SD)					
	**N Sites**	**Patients in Care with VS**		**Patients in Care Not Consistently Suppressed at Baseline**		**Patients in Care with CC**	
		**N[1]**	**VS**	**N[2]**	**VS**	**N[1]**	**CC**
**Overall**	37	374 (478)	62% (16)	222 (269)	46% (12)	374 (478)	45% (19)
**Subgroups**							
Bronx, NY	20	425 (557)	62% (13)	243 (302)	42% (8)	425 (557)	56% (16%)
Washington, DC	17	314 (372)	63% (19)	198 (232)	49% (15)	314 (372)	32% (15)
Hospital-based	14	579 (611)	60% (20)	331(328)	43% (14%)	579 (611)	46% (24)
Community-based	23	250 (332)	64% (13)	157 (207)	47% (11)	250 (332)	44% (16)
Smaller (≤196 at baseline)	19	96 (56)	56% (19)	68 (46)	41% (14)	96 (56)	41% (23)
Larger (>196 at baseline)	18	668 (550)	69% (8)	385 (311)	50% (9)	668 (550)	48% (14)
Lower base VS (Baseline≤66%)	20	230 (300)	53% (16)	163 (203)	39% (11)	230 (300)	39% (20%)
Higher base VS (Baseline>66%)	17	544 (593)	73% (6)	293 (323)	53% (10)	544 (593)	52% (16)

N[1] = Average number of person in care per quarter at a site during the baseline period.

N[2] = Average number of person in care per quarter at a site not consistently virally suppressed during the baseline period.

VS = Average proportion of patients in care with viral suppression per quarter during the baseline period.

CC = Average proportion of patients with continuity of care during the baseline period.

Copyright 2017 El-Sadr WM et al. JAMA Internal Medicine.

eTable 5: Effect of Financial Incentives (FI) and Standard-of-Care (SOC) on Viral Load Suppression (VS) for Patients at Peak Intervention Period

	Mean (SD)			
	**Patients in care at peak intervention**	**Proportion with VS during intervention**	**Change in proportion with VS from baseline**	**Effect of FI: Increase in proportion with VS (95% CI), p value**
**Overall**				
FI (N=17)	529 (646)	72% (12)	14% (11)	4.6% (0.4%, 8.8%), **p=0.031**
SOC (N=20)	404 (465)	70% (15)	4% (8)	
		**Subgroups**		
Bronx, NY				
FI (N=10)	554 (714)	74% (7)	11% (8)	3.5% (0.1%, 7.0%), **p=0.047**
SOC (N=10)	385 (543)	67% (19)	6% (5)	
Washington, DC				
FI (N=7)	493 (588)	69% (17)	19% (14)	6.6% (-2.3%, 15.6%), p=0.14
SOC (N=10)	424 (402)	74% (11)	3% (10)	
Community-based				
FI (N=10)	360 (499)	75% (8)	13% (6)	2.2% (-1.0%, 5.4%), p=0.16
SOC (N=13)	295 (389)	72% (18)	6% (5)	
Hospital-based				
FI (N=7)	770 (790)	68% (16)	15% (17)	6.1% (-2.0%, 14.2%), p=0.13
SOC (N=7)	607 (556)	68% (9)	1% (11)	
Smaller (≤196 at baseline)				
FI (N=9)	169 (203)	68% (15)	20% (12)	13.6% (0.5%, 26.7%), **p=0.043**
SOC (N=10)	121 (67)	68% (19)	5% (5)	
Larger (>196 at baseline)				
FI (N=8)	933 (745)	76% (5)	7.5% (5)	3.9% (-1.3%, 9.2%), p=0.13
SOC (N=10)	688 (523)	73% (10)	3% (8)	
Lower base VS (Baseline≤66%)				
FI (N=11)	240 (253)	69% (14)	18% (11)	6.8% (-1.1%, 14.8%), p=0.088
SOC (N=9)	360 (473)	63% (18)	6% (4)	
Higher base VS (Baseline>66%)				
FI (N=6)	1057 (830)	78% (3)	6% (4)	4.7% (-1.3%, 10.6%), p=0.12
SOC (N=11)	441 (478)	76% (10)	3% (9)	

Copyright 2017 El-Sadr WM et al. JAMA Internal Medicine.

eFigure 1: Change in Proportion of Patients with Viral Suppression over Time by Study Arm

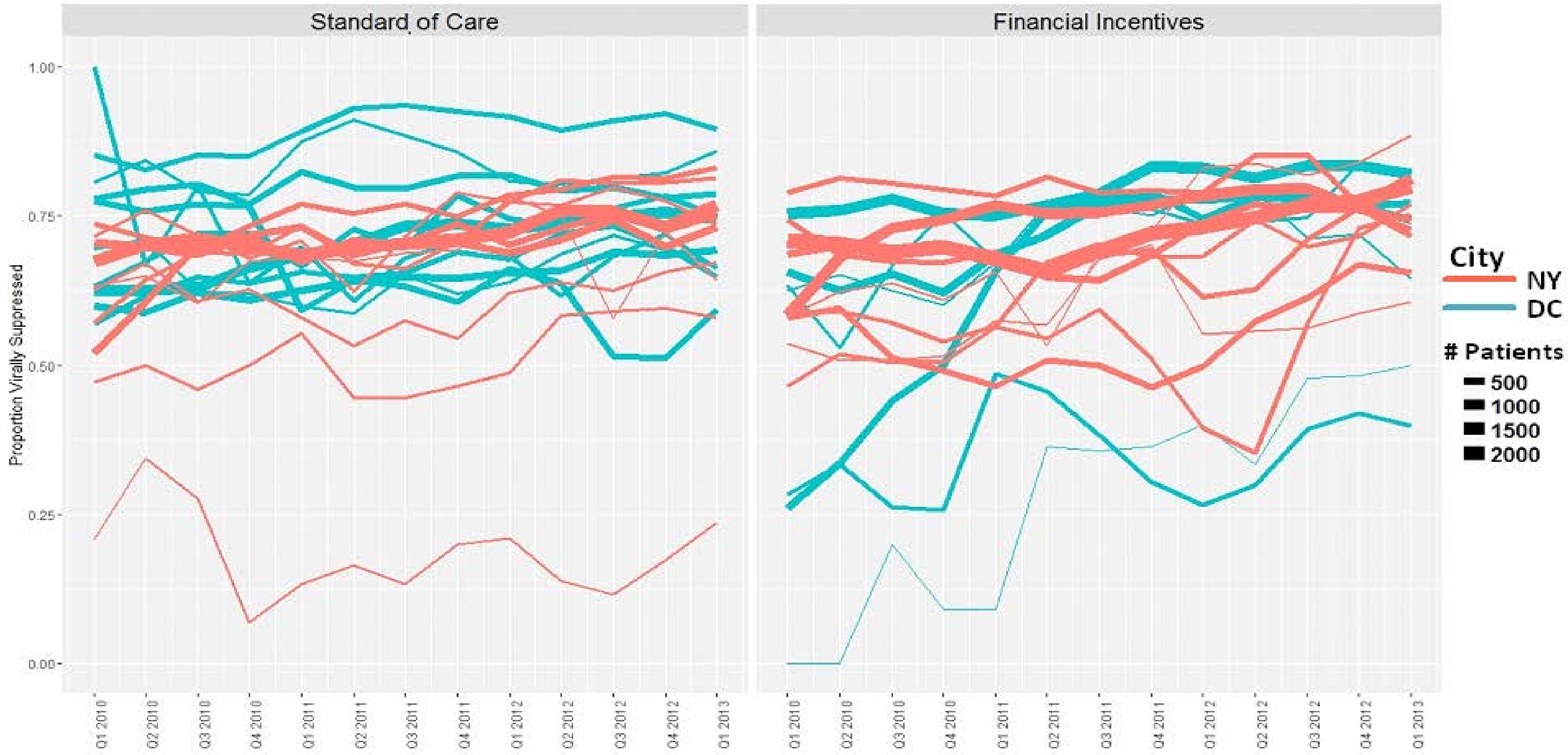

Copyright 2017 El-Sadr WM et al. JAMA Internal Medicine.

Copyright 2017 El-Sadr WM et al. JAMA Internal Medicine.
